# Supplementary material for: Involvement of casein kinase 1 epsilon/delta (Csnk1e/d) in the pathogenesis of familial Parkinson's disease caused by CHCHD2
Source: EMBO Mol Med. 2023 Aug 14;15(9):e17451. doi: 10.15252/emmm.202317451 (PMC10493588; doi:10.15252/emmm.202317451)
Supplement: Supplementary file 1 — Appendix [file EMMM-15-e17451-s006.pdf]

Torii et al.

Involvement of casein kinase 1 epsilon/delta (Csnk1e/d) in the pathogenesis of familial Parkinson’ s disease caused by CHCHD2 gene mutations

Table of contents

Appendix Figure S1..2
Appendix Figure S2..4
Appendix Figure S3..6
Appendix Figure S4..8
Appendix Figure S5..10
Appendix Figure S6..12
Appendix Figure S7..13
Appendix Figure S8..15
Appendix Figure S9..16
Appendix Figure S10..18
Appendix Figure S11..19
Appendix Figure S12..21
Appendix Figure S13..22
Appendix Figure S14..24
Appendix Figure S15..25
Appendix Figure S16..27
Appendix Figure S17..28
Appendix Figure S18..30

Appendix Table S1...32
Appendix Table S2...34

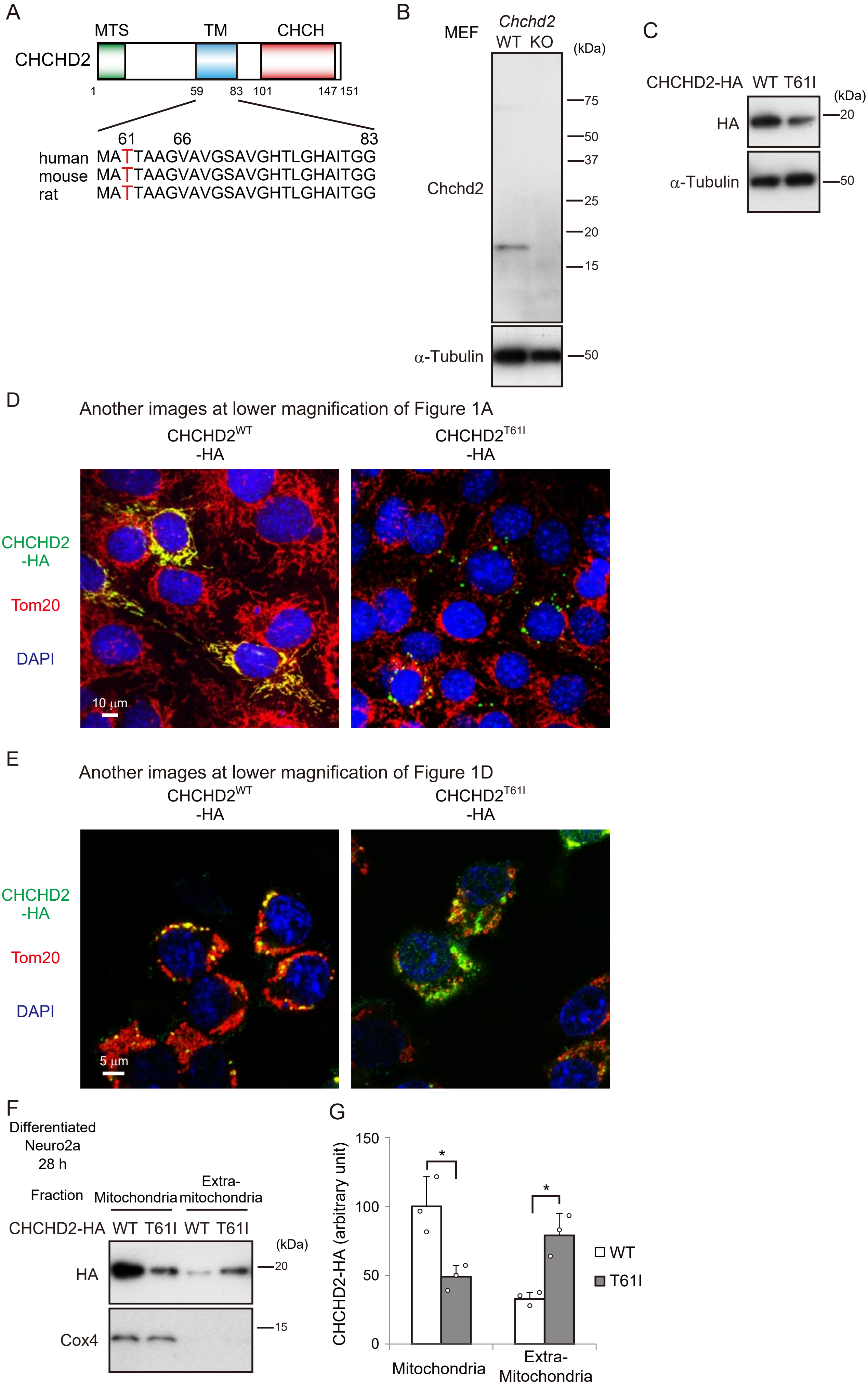
A
MTS
TM
CHCH
CHCHD2
1
59
83
101
147
151
61
66
83
human MATTAAGVAVGSAVGHTLGHAITGG
mouse MATTAAGVAVGSAVGHTLGHAITGG
rat MATTAAGVAVGSAVGHTLGHAITGG
B
MEF
Chchd2
WT KO
(kDa)
75
50
37
25
20
15
Chchd2
α-Tubulin
50
C
CHCHD2-HA WT T61I
(kDa)
HA
20
α-Tubulin
50
D
Another images at lower magnification of Figure 1A
CHCHD2WT -HA
CHCHD2T61I -HA
CHCHD2 -HA
Tom20
DAPI
10 μm
E
Another images at lower magnification of Figure 1D
CHCHD2WT -HA
CHCHD2T61I -HA
CHCHD2 -HA
Tom20
DAPI
5 μm
F
Differentiated Neuro2a 28 h
Fraction
Mitochondria
Extra-mitochondria
CHCHD2-HA WT T61I WT T61I
(kDa)
HA
20
Cox4
15
G
CHCHD2-HA (arbitrary unit)
150
100
50
0
*
*
Mitochondria
Extra-Mitochondria
WT
T61I

Appendix Fig. S1. Structure and expression of CHCHD2

(A) Structure of CHCHD2. MTS: mitochondrial targeting signal, TM: central α-helix (putative transmembrane domain), CHCH: coiled-coil-helix-coiled-coil-helix domain. Alignment of amino acid sequences of the TM of mammalian CHCHD2 (human, mouse, and rat). Thr^{61} is marked in red. **(B)** Lack of endogenous Chchd2 in *Chchd2*^{KO} MEFs. Cell lysates from *Chchd2*^{WT} and *Chchd2*^{KO} MEFs were subjected to western blotting to verify the expression of endogenous Chchd2. **(C)** Expression of Chchd2 and its mutant protein. *Chchd2*^{KO} cells were transfected with the *CHCHD2*^{WT}*-HA* and *CHCHD2*^{T61I}*-HA* plasmids. At 48 hr after transfection, cell lysates were subjected to western blotting. **(D)** Another representative images at lower magnification in Fig. 1A are shown. **(E)** Additional representative low-magnification images of the images in Fig. 1D are shown. **(F)** Neuro2a cells were transfected with the *CHCHD2*^{WT}*-HA* and *CHCHD2*^{T61I}*-HA* plasmids for 4 hr, and then cultured in medium containing 2% FBS and 10 µM retinoic acid. At 28 hr after transfection, cells were fractionated into mitochondria and non-mitochondria, and the expression of each protein was analyzed by western blotting. **(G)** A semiquantitative analysis of protein expression in **(F)** is shown. Data are shown as the mean ± SD ($n = 3$). Comparisons were performed using one-way ANOVA followed by the Tukey *post-hoc* test. *$p < 0.05$

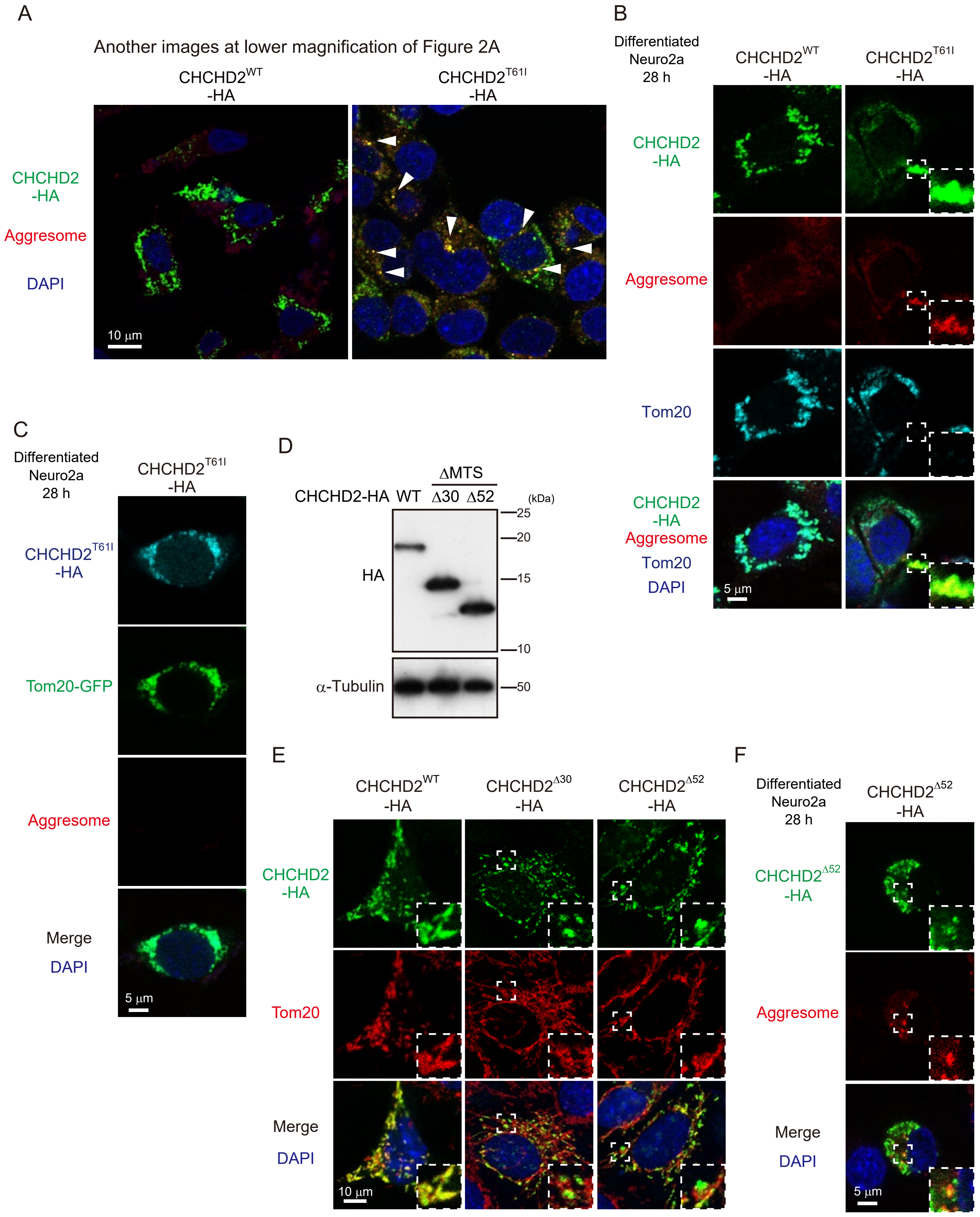
A
Another images at lower magnification of Figure 2A
CHCHD2WT -HA
CHCHD2T61I -HA
CHCHD2 -HA
Aggresome
DAPI
10 μm
B
Differentiated Neuro2a 28 h
CHCHD2WT -HA
CHCHD2T61I -HA
CHCHD2 -HA
Aggresome
Tom20
CHCHD2 -HA Aggresome Tom20 DAPI
5 μm
C
Differentiated Neuro2a 28 h
CHCHD2T61I -HA
CHCHD2T61I -HA
Tom20-GFP
Aggresome
Merge DAPI
5 μm
D
ΔMTS
CHCHD2-HA WT Δ30 Δ52
(kDa)
25
20
15
10
50
HA
α-Tubulin
E
CHCHD2WT -HA
CHCHD2Δ30 -HA
CHCHD2Δ52 -HA
CHCHD2 -HA
Tom20
Merge DAPI
10 μm
F
Differentiated Neuro2a 28 h
CHCHD2Δ52 -HA
CHCHD2Δ52 -HA
Aggresome
Merge DAPI
5 μm

Appendix Fig. S2. Formation of aggresomes by extra-mitochondrial CHCHD2 mutant proteins

(A) Additional representative images at lower magnification of those in Fig. 2A are shown. Arrowheads indicate aggresomes containing CHCHD2^{T61I}-HA. **(B)** Neuro2a cells were transfected with *CHCHD*^{IWT}*-HA* and *CHCHD2*^{T61I}*-HA* plasmids for 4 hr, and then cultured in medium containing 2% FBS and 10 µM retinoic acid. At 28 hr after transfection, cells were stained with anti-HA and anti-Tom20 antibodies, and ProteoStat aggresome dye. Representative images are shown. The region of interest (ROI) indicates extramitochondrial aggresomes containing CHCHD2^{T61I}-HA, but not mitochondria (dashed squares). Magnified images of the areas are shown in the insets. **(C)** Neuro2a cells were transfected with *CHCHD2*^{T61I}*-HA* and *Tom20-GFP* plasmids for 4 hr, and then cultured in medium containing 2% FBS and 10 µM retinoic acid. At 28 hr after transfection, cells were stained with an anti-HA and anti-GFP antibodies, and ProteoStat aggresome dye. Representative images are shown. Intramitochondrial CHCHD2^{T61I}-HA did not generate aggresomes. **(D, E)** Extra-mitochondrial expression of CHCHD2$^{\Delta 30}$-HA and CHCHD2$^{\Delta 52}$-HA. The indicated CHCHD2 mutant proteins were expressed in *Chchd2*^{KO} cells by plasmid transfection. At 48 hr after transfection, cells were lysed and subjected to western blotting **(D)**, and were stained with anti-HA and anti-Tom20 antibodies and observed by confocal microscopy **(E)**. Magnified images of the areas within the dashed squares are shown in the insets. **(F)** Formation of aggresomes in Neuro2a cells expressing extra-mitochondrial CHCHD2$^{\Delta 52}$-HA. Neuro2a cells were transfected with the *CHCHD2*^{T61I}*-HA* plasmid for 4 hr, and then cultured in medium containing 2% FBS and 10 µM retinoic acid. At 28 hr after transfection, cells were stained with an anti-HA antibody and ProteoStat aggresome dye. Representative images are shown. Magnified images of the areas within the dashed squares are shown in the insets.

A

Another images at lower magnification of Figure 2C

CHCHD2^{WT}-HA CHCHD2^{T61I}-HA

CHCHD2-HA
Nefl
DAPI

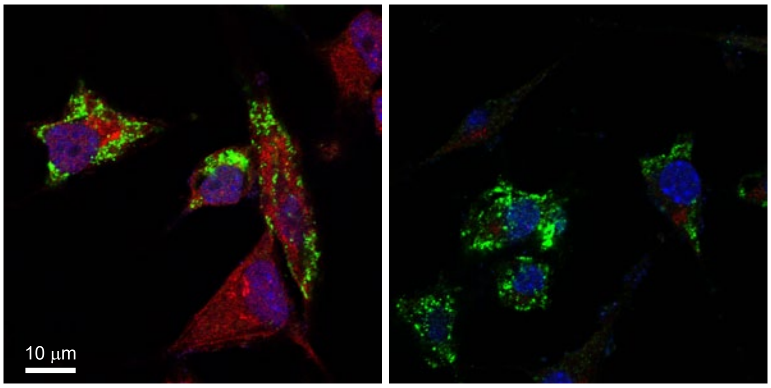

B

Another images at lower magnification of Figure 2E

CHCHD2^{WT}-HA CHCHD2^{T61I}-HA

CHCHD2-HA
p-Nefl^{473}
DAPI

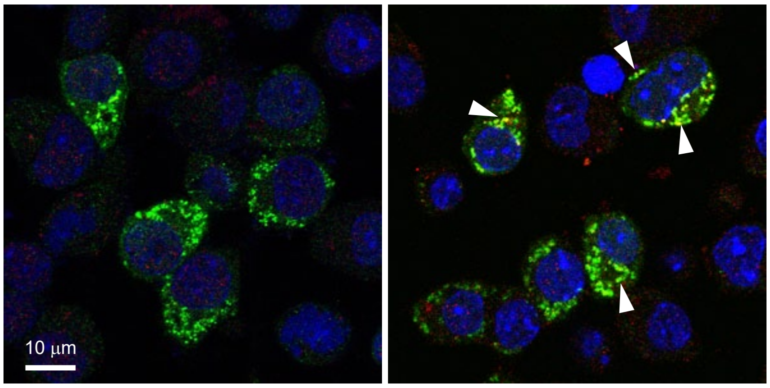

C

Another iamges at lower magnification of Figure 2G

CHCHD2^{WT}-HA CHCHD2^{T61I}-HA

CHCHD2-HA
p-α-Syn^{129}
DAPI

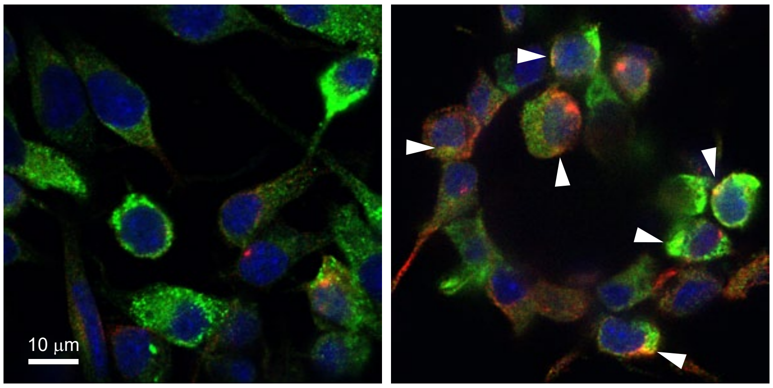

D

Differentiated Neuro2a 28 h

CHCHD2^{WT}-HA CHCHD2^{T61I}-HA

Tom20 (pseudocolor)

p-Nefl^{473}

Tom20
p-Nefl^{473}
DAPI

GFP

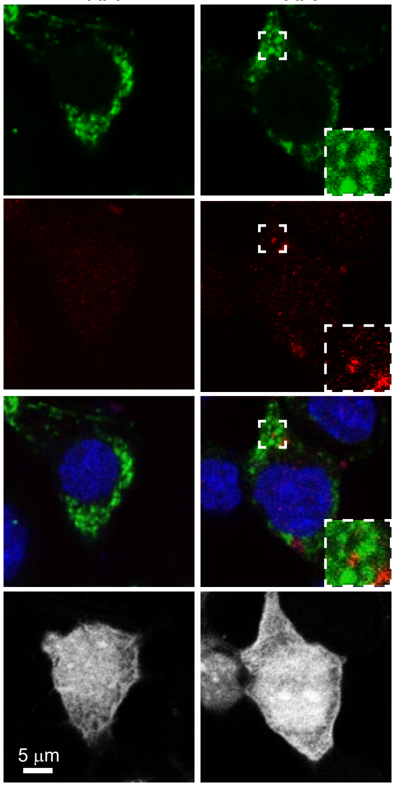

E

Differentiated Neuro2a 28 h

CHCHD2^{WT}-HA CHCHD2^{T61I}-HA

Tom20 (pseudocolor)

p-α-Syn^{129}

Tom20
p-α-Syn^{129}
DAPI

GFP

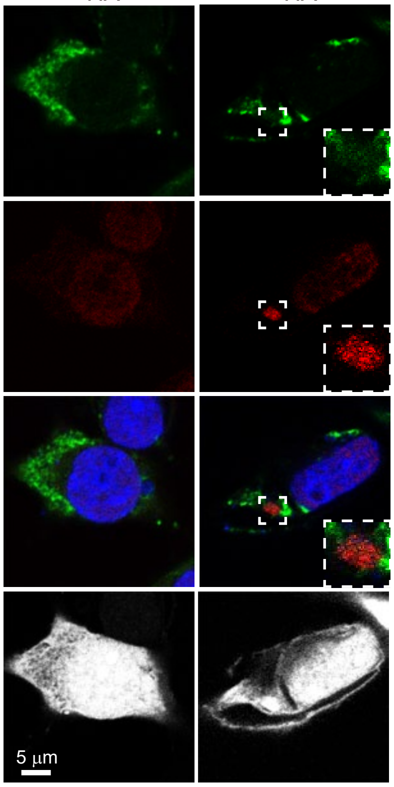

Appendix Fig. S3. Generation of p-Nefl^{473} and p-α-Syn^{129} puncta in cells expressing extra-mitochondrial CHCHD2^{T61I}

(**A-C**) Additional representative lower magnification images of the images in Fig. 2C, E, and G are shown. **(D, E)** Neuro2a cells were transfected with *CHCHD^{WT}-HA*, *CHCHD2^{T61I}-HA*, and *pmax-GFP* plasmids for 4 hr, and then cultured in medium containing 2% FBS and 10 µM retinoic acid. At 28 hr after transfection, cells were stained with anti-Tom20 and anti-p-Nefl^{473} **(D)** or anti-p-α-Syn^{129} **(E)** antibodies. Extramitochondrial p-Nefl^{473} and p-α-Syn^{129} puncta (dashed squares) were observed only in *CHCHD2^{T61I}-HA* expressing cells. Magnified images of the areas are shown in the insets. GFP is a marker of successful transfection.

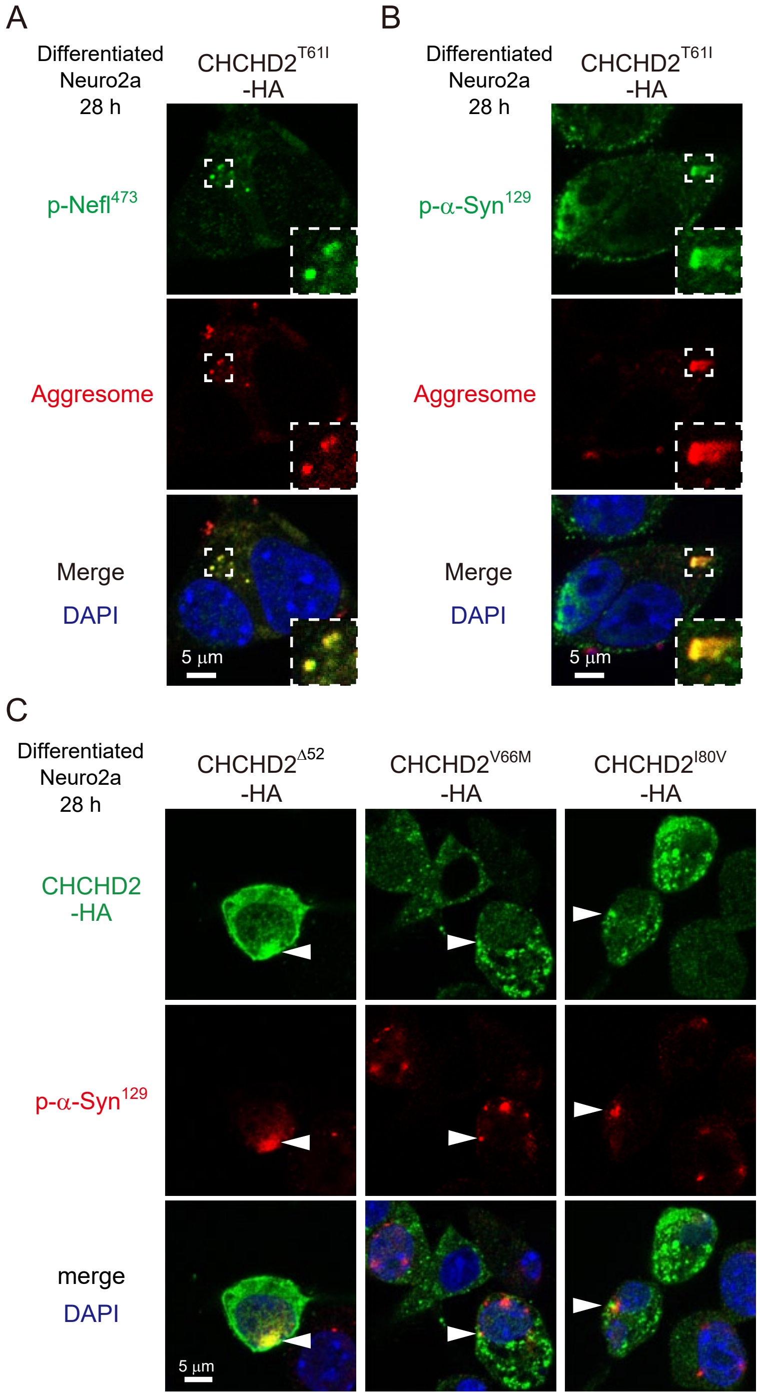
A
Differentiated Neuro2a 28 h
CHCHD2T61I -HA
p-Nefl473
Aggresome
Merge
DAPI
5 µm
B
Differentiated Neuro2a 28 h
CHCHD2T61I -HA
p-α-Syn129
Aggresome
Merge
DAPI
5 µm
C
Differentiated Neuro2a 28 h
CHCHD2Δ52 -HA
CHCHD2V66M -HA
CHCHD2I80V -HA
CHCHD2 -HA
p-α-Syn129
merge
DAPI
5 µm

Appendix Fig. S4. Generation of aggresome and p-α-Syn^{129} puncta in cells expressing extra-mitochondrial CHCHD2 mutant proteins

(A, B) Localization of p-Nefl^{473} and p-α-Syn^{129} in aggresomes formed in cells expressing CHCHD2^{T61I}-HA. Neuro2a cells were transfected with the *CHCHD2^{T61I}-HA* plasmid for 4 hr. At 28 hr after transfection, cells were stained with anti-p-Nefl^{473} **(A)** and anti-p-α-Syn^{129} **(B)** antibodies, and ProteoStat aggresome dye. Representative images are shown. Magnified images of the areas within the dashed squares are shown in the insets. **(C)** Neuro2a cells were transfected with the *CHCHD2$^{\Delta 52}$-HA*, *CHCHD2^{V66M}-HA*, and *CHCHD2^{I80V}-HA* plasmids for 4 hr, and then cultured in medium containing 2% FBS and 10 µM retinoic acid for neuronal differentiation. At 28 hr after transfection, cells were stained with anti-HA and anti-p-α-Syn^{129} antibodies. Arrowheads indicate colocalizing puncta.

Appendix Figure S5

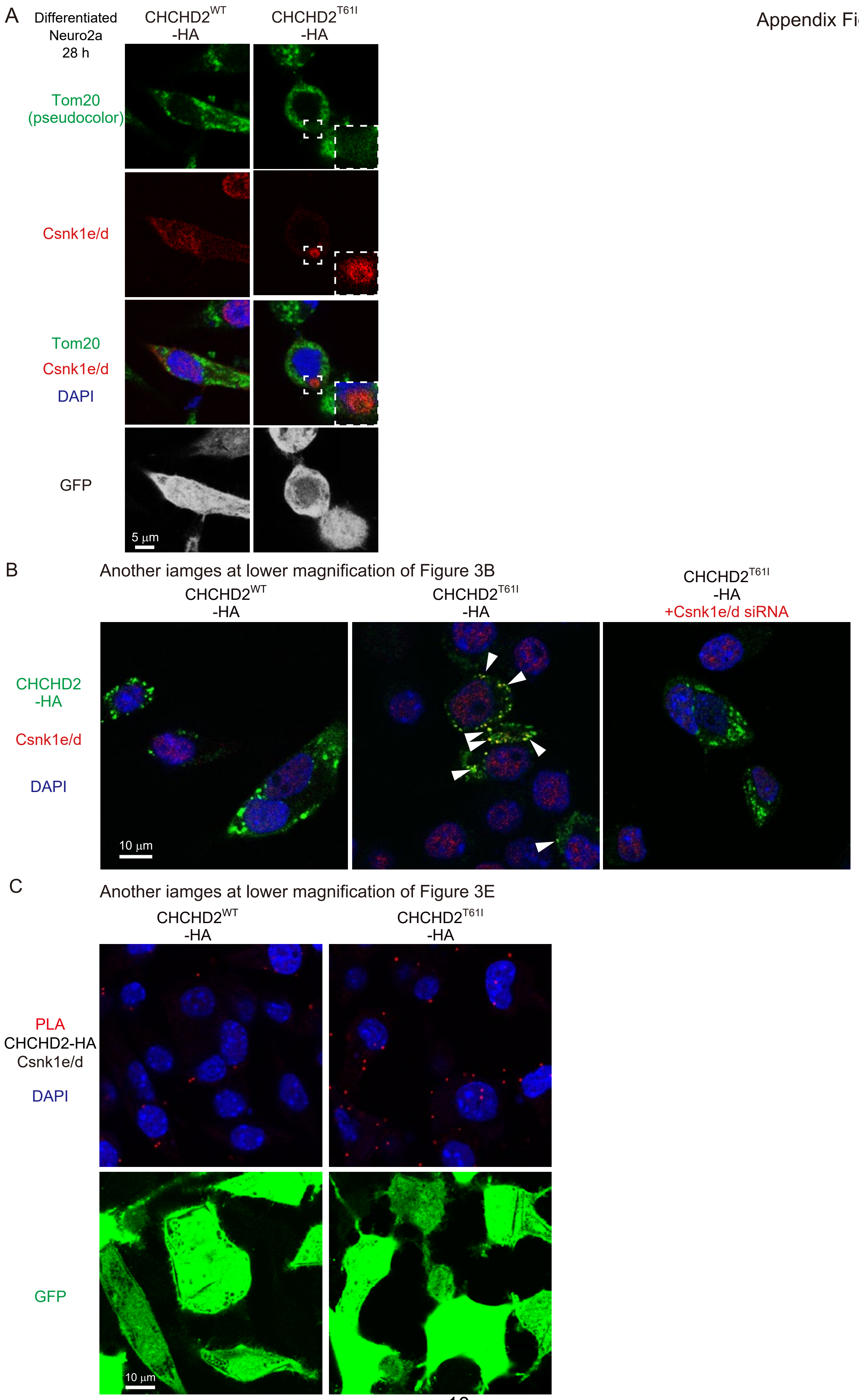

Appendix Fig. S5 Interaction between CHCHD2^{T61I} and Csnk1e/d in outside of mitochondria

(A) Neuro2a cells were transfected with the *CHCHD2^{WT}-HA*, *CHCHD2^{T61I}-HA*, and *pmax-GFP* plasmids (to detect transfected cells) for 4 hr. At 28 hr after transfection, cells were stained with anti-Tom20 and anti-Csnk1e/d antibodies. Representative images are shown. Magnified images of the areas within the dashed squares are shown in the insets. **(B, C)** Additional representative lower magnification images of the images in Fig. 3B and E are shown. Arrows indicate colocalized puncta of CHCHD2^{T61I}-HA and Csnk1e/d.

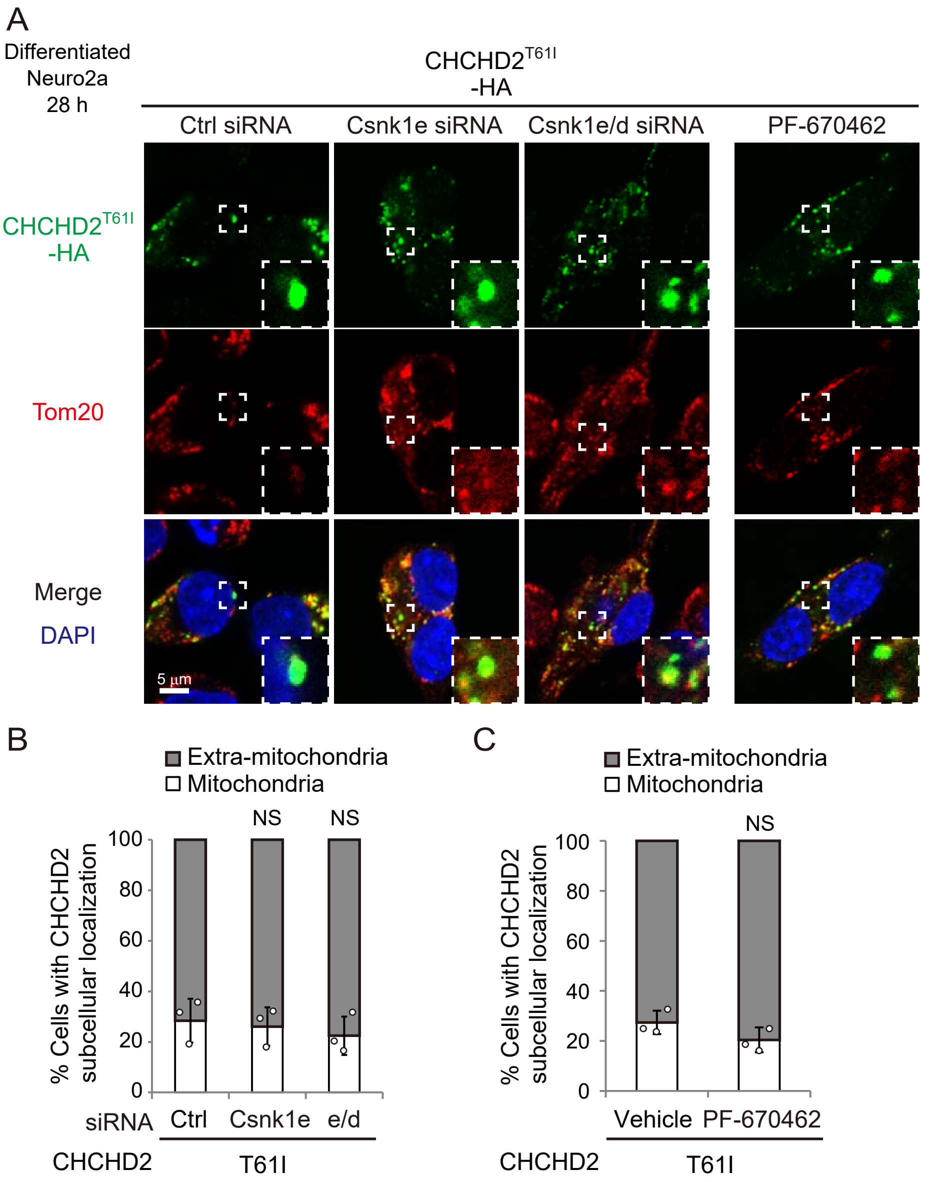

Appendix Fig. S6. No involvement of Csnk1e/d in the extra-mitochondrial localization of CHCHD2^{T61I}

Neuro2a cells were transfected with the *CHCHD2^{T61I}-HA* plasmid together with the indicated siRNAs, and were differentiated after 4 hr. At 28 hr after transfection, cells were fixed and stained with anti-HA and anti-Tom20 antibodies. Similar experiments were performed by the addition of PF-670462 (10 µM) instead of gene silencing. In **(A)**, representative images are shown. Magnified images of the areas within the dashed squares are shown in the insets. In **(B, C)**, the number of cells displaying mitochondrial CHCHD2^{T61I} and extra-mitochondrial CHCHD2^{T61I} puncta were quantified ($n \geq 100$ cells in each experiment). Data are shown as the mean ± SD ($n = 3$). Comparisons were performed using one-way ANOVA followed by the Tukey *post-hoc* test and the unpaired two-tailed Student *t*-test. NS: not significant

Appendix Figure S7

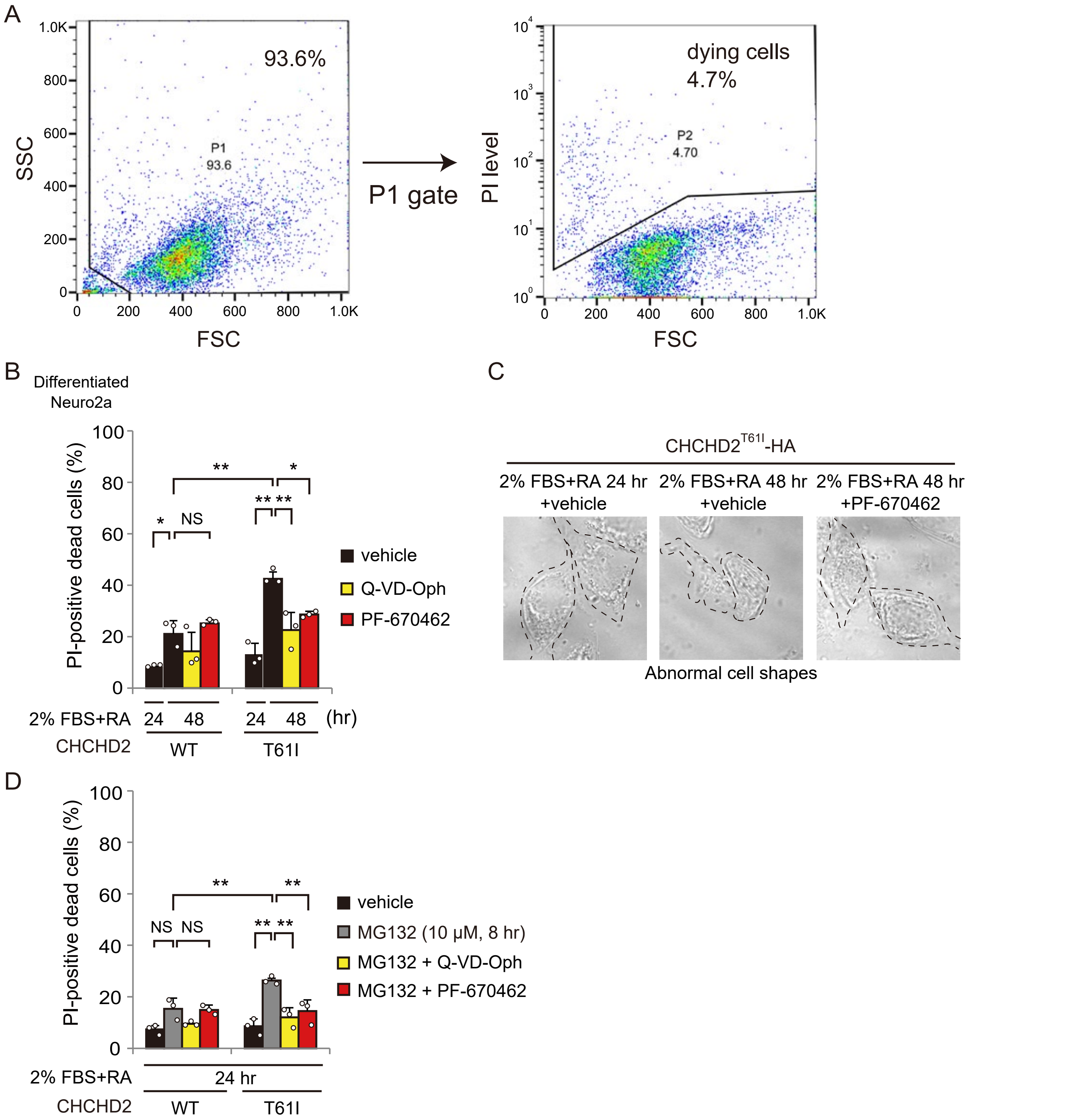

Appendix Fig. S7. Induction of cell death in cells expressing CHCHD2^{T61I} and its inhibition by Q-VD-Oph and PF-670462

(A) Flow cytometry gating strategy. For the cell viability assay, cellular debris was removed using FSC/SSC (left panel). Then, PI-positive dead cells were detected using FSC/PE (PI level) (right panel). **(B, C)** Neuro2a cells stably expressing CHCHD2^{WT} or CHCHD2^{T61I} were seeded onto 12-well plates. After 12 hr, the culture medium was changed to medium containing 2% FBS and 10 µM retinoic acid for neuronal differentiation in the presence or absence of Q-VD-Oph (25 µM) or PF-670462 (10 µM). At the indicated times, cell death was analyzed by the PI uptake assay **(B)**. Cell morphology was also observed by differential interference contrast microscopy **(C)**. Dashed lines indicate cell shapes. **(D)** Neuro2a cells stably expressing CHCHD2^{WT} or CHCHD2^{T61I} were seeded onto 12-well plates, and the culture medium was changed to medium containing 2% FBS and 10 µM retinoic acid for neuronal differentiation. After 16 hr, MG132 (10 µM) was added together with or without Q-VD-Oph (25 µM) or PF-670462 (10 µM) for 8 hr, and cell death was analyzed by the PI uptake assay. Comparisons were performed using one-way ANOVA followed by the Tukey *post-hoc* test. NS: not significant; $*p < 0.05$; $**p < 0.01$

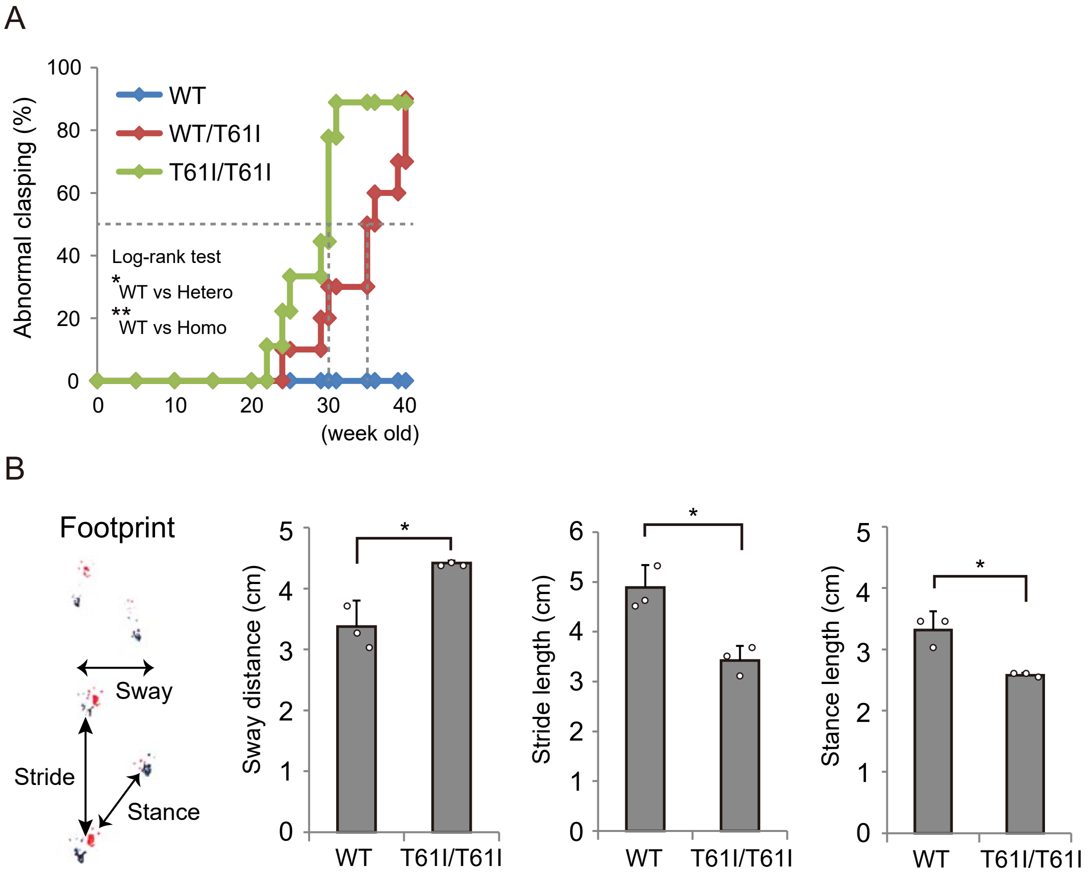

Appendix Fig. S8. Abnormal motor performance in Chchd2^{T61I} knock-in mice

(A) Quantitative analyses of the data in Fig. 5A are shown. Kaplan-Meier analysis of abnormal clasping of mice with the indicated genotypes. Comparisons were performed by the log-rank test (WT, n = 9; WT/T61I, n = 10; T61I/T61I, n = 9). **(B)** Sway, stride and stance length measured from the footprint analysis are shown with arrows (left). Quantitative analyses of the footprints are shown (right). Longer sway, shorter stride, and shorter stance indicate lower motor performance. Comparisons were performed using the unpaired two-tailed Student t-test. *$p < 0.05$; **$p < 0.01$

Appendix Figure S9

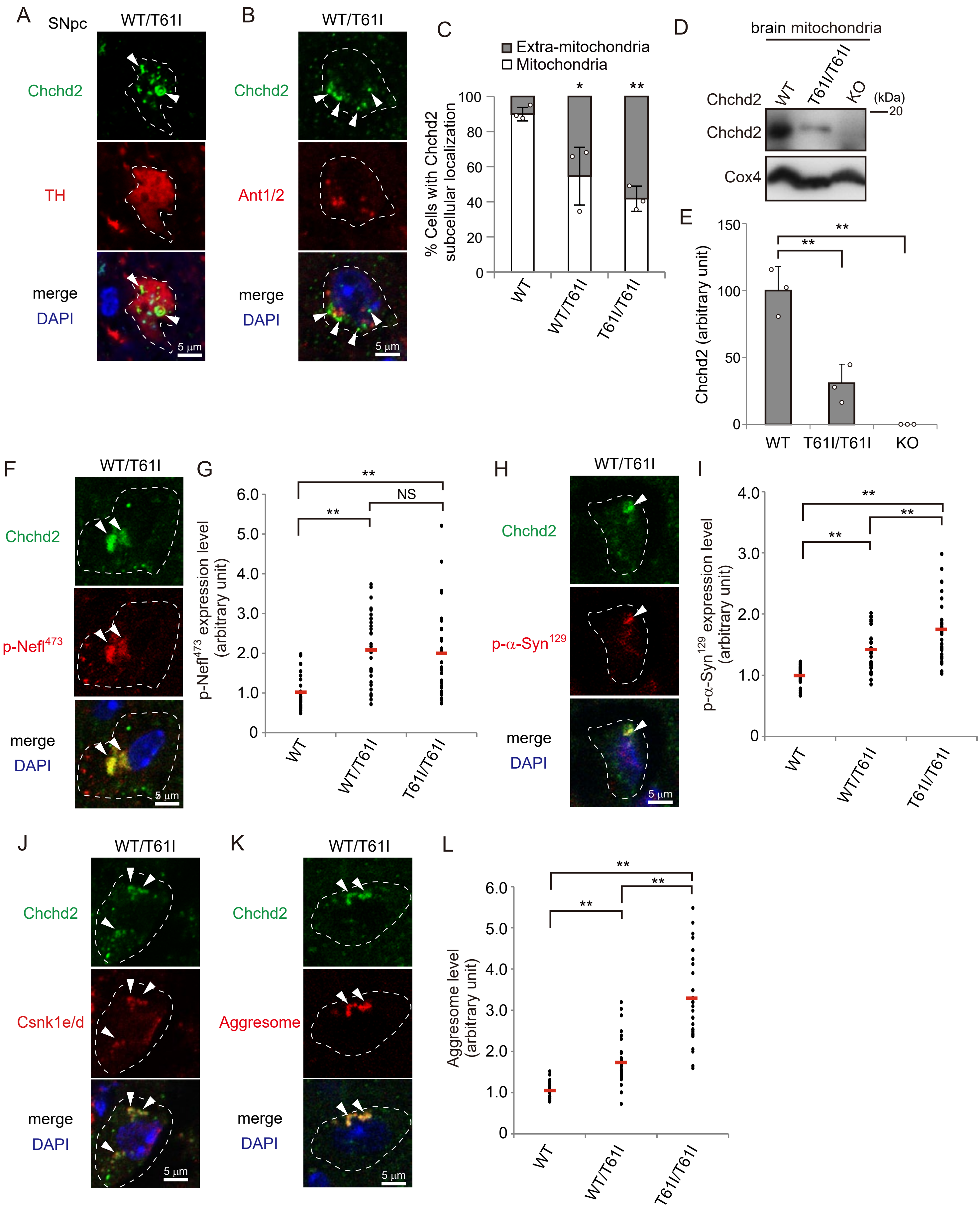

Appendix Fig. S9. Generation of p-Nefl^{473} and p-α-Syn^{129} puncta, and aggresomes in Chchd2^{T61I} knock-in mice

(A–C) Cryosections of the midbrain of the indicated Chchd2^{T61I} knock-in mice were immunostained using antibodies for the indicated proteins. Dashed lines indicate cell shapes. Arrowheads indicate Chchd2 puncta in a TH-positive DA **(A)** and an extra-mitochondrial Chchd2$^{WT/T61I}$ punctum **(B)**. **(C)** Population of cells displaying mitochondrial CHCHD2 puncta and extra-mitochondrial CHCHD2 puncta in the SNpc of each mouse ($n \geq 100$ cells in each experiment). Data are shown as the mean ± SD ($n = 3$). **(D, E)** Isolated mitochondrial lysates from the whole brain of WT, *Chchd2$^{T61I/T61I}$*, and *Chchd2^{KO}* mice were subjected to western blotting. **(E)** A semiquantitative analysis of the protein expression in **(D)** is shown. Data are shown as the mean ± SD ($n = 3$). **(F-L)** The levels of p-Nefl^{473} **(F, G)**, p-α-Syn^{129} **(H, I)**, Csnk1e/d **(J)** puncta, and aggresomes **(K, L)** were analyzed by the average fluorescence intensity per cell ($n = 30$ cells in each experiment). Representative images are shown in **(F, H, J, K)**. Arrowheads indicate CHCHD2 puncta colocalizing with p-Nefl^{473} **(F)**, p-α-Syn^{129} **(H)**, Csnk1e/d **(J)**, and aggresomes **(K)**. In **(G, I, L)**, red bars indicate mean values. Comparisons were performed using one-way ANOVA followed by the Tukey post-hoc test. NS: not significant, $^*p < 0.05$; $^{**}p < 0.01$

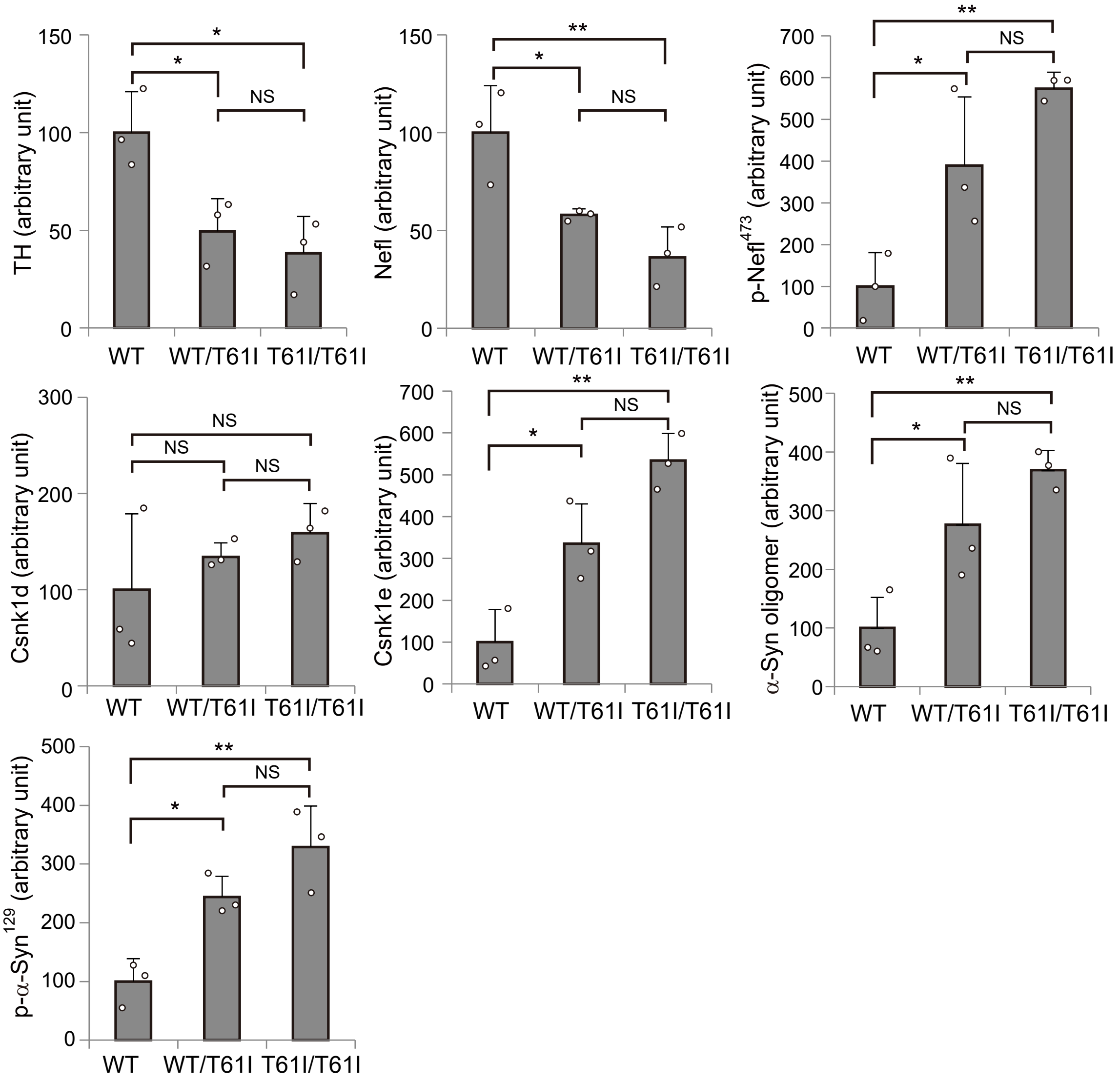

Appendix Fig. S10. Semiquantitative analyses of protein expression in the isolated midbrain and diencephalon

Expression levels of the indicated proteins from Fig. 5L are shown as the mean ± SD ($n = 3$). Comparisons were performed using one-way ANOVA followed by the Tukey post-hoc test. NS: not significant, *$p < 0.05$; **$p < 0.01$

Appendix Figure S11

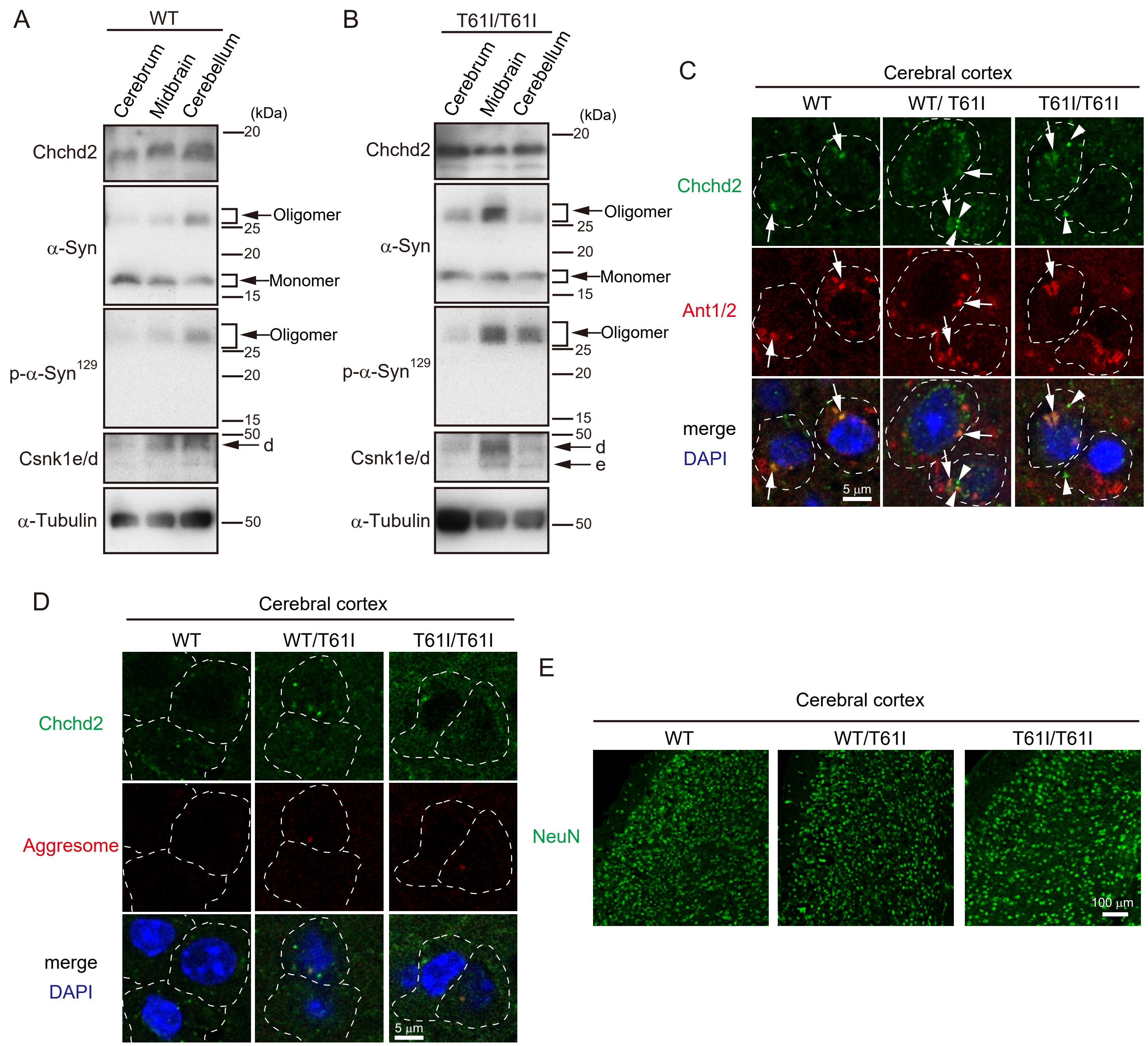

Appendix Fig. S11. Low level of p-α-Syn^{129} and absence of aggresome formation in the cerebral cortex and cerebellum of *CHCHD2^{T61I}* knock-in mice

(A, B) Lysates of the indicated brain regions from WT mice **(A)** and CHCHD2$^{T61I/T61I}$ knock-in mice **(B)** were prepared and subjected to western blotting. **(C)** Cryosections of the cerebral cortex of each mouse were immunostained with anti-Chchd2 and anti-Ant1/2 antibodies. Dashed lines indicate cell shape. Arrows and arrowheads indicate mitochondrial Chchd2 puncta and extra-mitochondrial Chchd2 puncta, respectively. **(D)** Cryosections of the cerebral cortex were immunostained with an anti-Chchd2 antibody and ProteoStat aggresome dye. Dashed lines indicate cell shapes. Aggresomes were not observed in these cells. **(E)** Cryosections of the cerebral cortex were immunostained with an anti-NeuN antibody to detect neuronal injury. No clear neuronal injury was detected in CHCHD2$^{T61I/T61I}$ and CHCHD2$^{WT/T61I}$ knock-in mice.

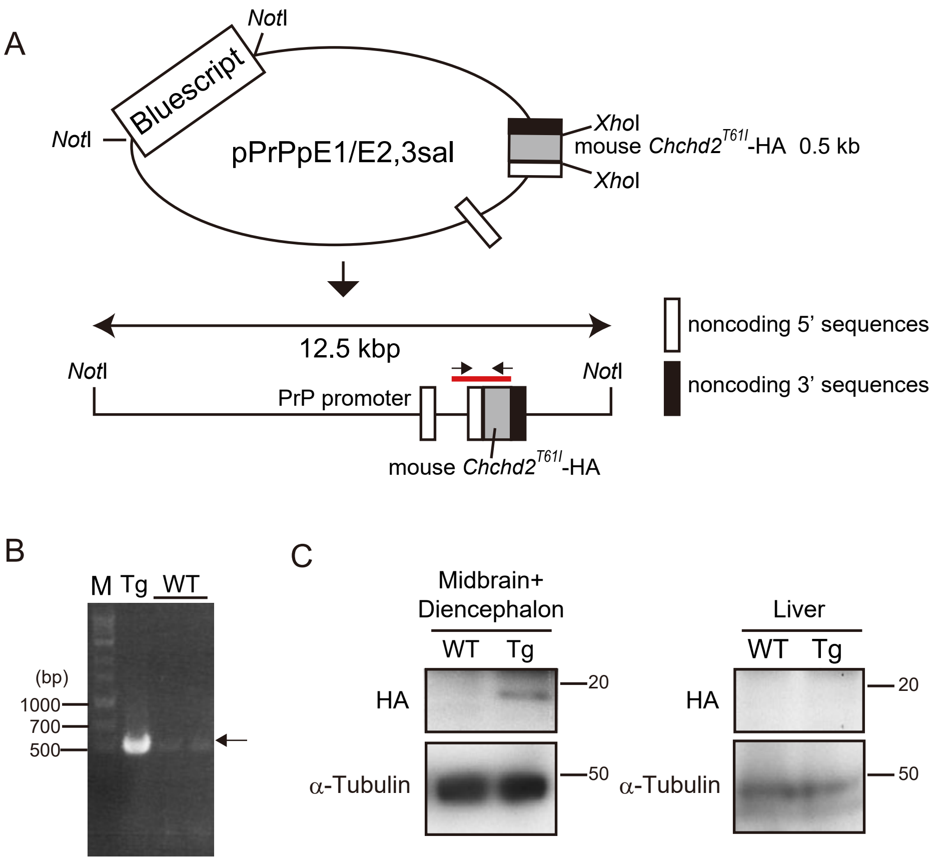

Appendix Fig. S12. Generation of Chchd2^{T61I}-HA Tg mice

(A) Schematic representation of the vector used to generate Chchcd2^{T61I}-HA Tg mice. The *Chchd2* ^{T61I}-HA fragment was inserted into the *Xho*I site in the pPrPpE1/E2,3sal vector. The pBluescript vector sequence was removed by digestion with *Not*I, resulting in a 12.5-kbp fragment (bottom). Small arrows indicate the position of the primers used for genotyping PCR and the red line indicates the PCR product (about 600 bp). **(B)** Genotyping was performed by genome amplification by PCR [product about 600 bp, a red line in **(A)**] from tail genomic DNA. The primers used are listed in Appendix Table S1. M indicates the lane of DNA markers. **(C)** Lysates from the indicated tissues were obtained from Chchcd2^{T61I}-HA Tg mice and their littermate WT mice, and were subjected to western blotting. Chchcd2^{T61I}-HA was expressed in the midbrain and diencephalon, but not in the liver.

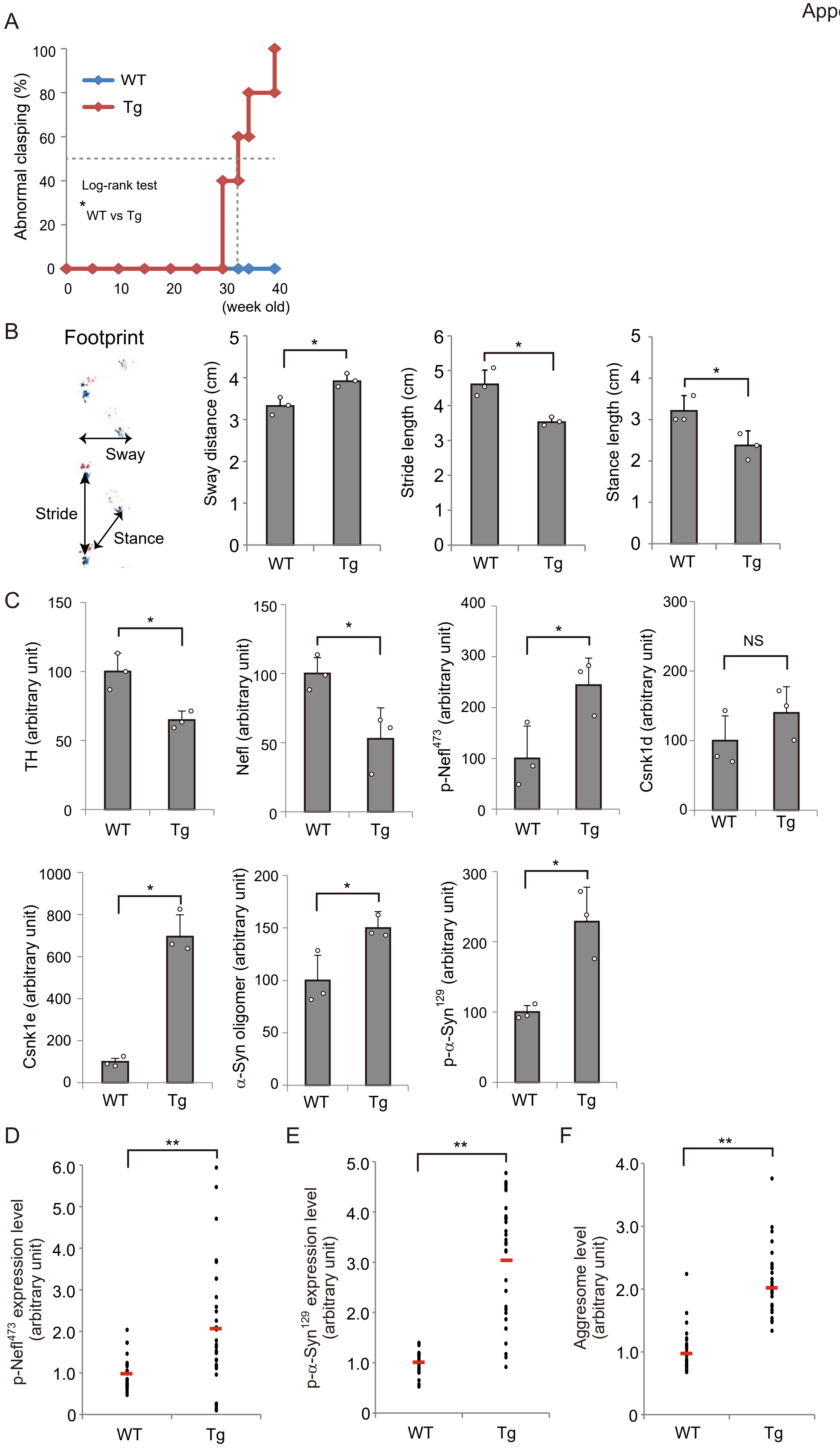
A
Abnormal clasping (%)
WT
Tg
Log-rank test
* WT vs Tg
(week old)
B
Footprint
Sway
Stride
Stance
Sway distance (cm)
Stride length (cm)
Stance length (cm)
C
TH (arbitrary unit)
Nefl (arbitrary unit)
p-Nefl473 (arbitrary unit)
Csnk1d (arbitrary unit)
NS
Csnk1e (arbitrary unit)
α-Syn oligomer (arbitrary unit)
p-α-Syn129 (arbitrary unit)
D
p-Nefl473 expression level (arbitrary unit)
E
p-α-Syn129 expression level (arbitrary unit)
F
Aggresome level (arbitrary unit)

Appendix Fig. S13. Reduced motor performance and upregulation of p-Nefl^{473}, p-α-Syn^{129}, and aggresomes in the SNpc of Chchd2^{T61I}-HA Tg mice

(A) Quantitative analyses of the data in Fig. EV4A are shown. Kaplan-Meier analysis of the abnormal clasping of mice with the indicated genotypes. Comparisons were performed by the log-rank test (WT, $n = 5$; Chchd2^{T61I}-HA Tg, $n = 5$). **(B)** Sway, stride and stance length measured from footprint analysis are shown with arrows (left). Quantitative analyses of the footprints in Fig. EV4C are shown (right). Longer sway, shorter stride, and shorter stance indicate lower motor performance. **(C)** Semiquantitative analyses of protein expression in Fig. EV4B. **(D-F)** The amount of p-Nefl^{473} **(D)**, p-α-Syn^{129} **(E)**, and aggresomes **(F)** in the SNpc of Chchd2^{T61I}-HA Tg mice was quantified by the average fluorescence intensity per cell ($n = 30$ cells in each experiment). Representative images are shown in Fig. EV4I–K. Red bars indicate mean values. In **(B-F)**, comparisons were performed by the unpaired two-tailed Student *t*-test. $*p < 0.05$; $**p < 0.01$. NS: not significant

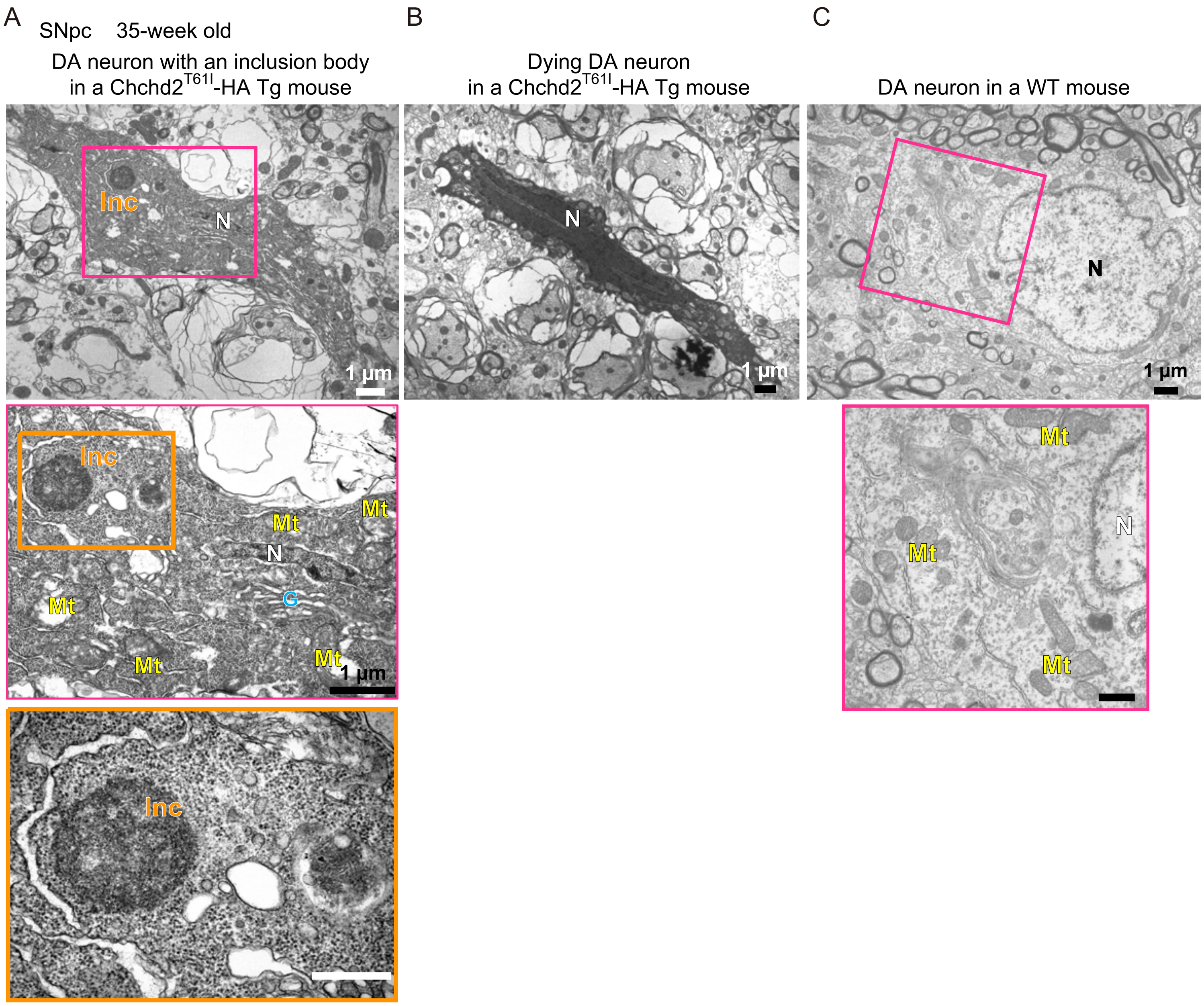

Appendix Fig. S14. Abnormal inclusion bodies and cell death in DA neurons of Chchd2^{T61I}-HA Tg mice observed by EM

(A, B) Sections of DA neurons in the SNpc of CHCHD2^{T61I}-HA Tg mice. **(A)** The DA neuron contained an inclusion body (Inc), and showed an abnormal nuclear shape (N), swollen mitochondria (Mt), and swollen Golgi (G). A magnified image of the area within the pink square of the top panel is shown in the middle panel, and that of the orange square in the middle panel is shown in the bottom panel. The inclusion body did not have a clear membrane. A low magnification image is shown in Fig. EV4M. **(B)** A dying DA neuron is shown. **(C)** A normal DA neuron in the SNpc of a WT mouse. A magnified image of the area within the pink square of the top panel is shown in the bottom panel.

Appendix Figure S15

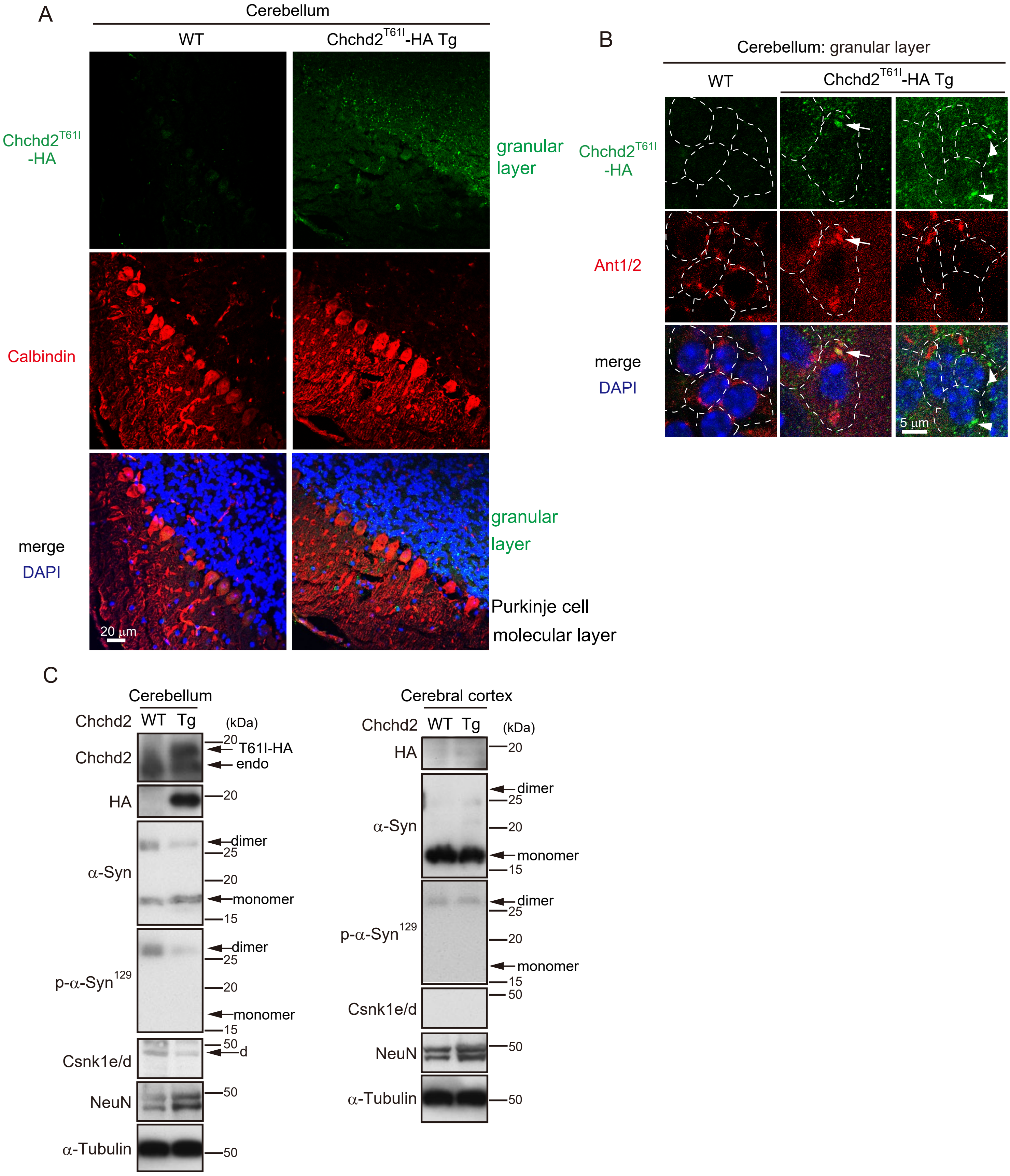

Appendix Fig. S15. No upregulation of p-α-Syn^{129} in the cerebellum and cerebral cortex of Chchd2^{T61I}-HA Tg mice

(A) Cryosections of the cerebellum of WT and Chchd2^{T61I}-HA Tg mice were immunostained with anti-HA and anti-calbindin antibodies. Chchd2^{T61I}-HA was expressed in the granular layer of the cerebellum. **(B)** Cryosections of the granular layer of the cerebellum in WT and Chchd2^{T61I}-HA Tg mice were immunostained with anti-HA and anti-Ant1/2 antibodies. Dashed lines indicate the cell shapes. Arrows and arrowheads indicate mitochondrial Chchd2^{T61I}-HA and extra-mitochondrial Chchd2^{T61I}-HA, respectively. **(C)** Lysates of the cerebellum (left) and cerebral cortex (right) were subjected to western blotting for the indicated proteins.

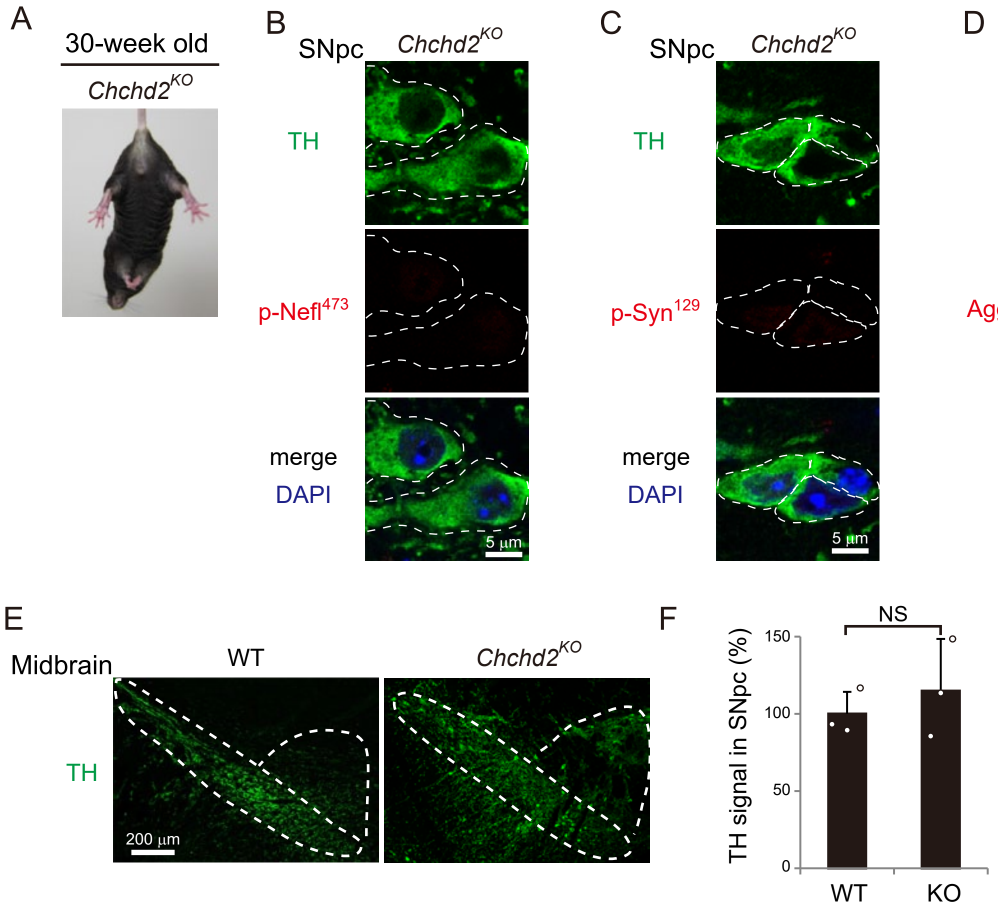

Appendix Fig. S16. Different neuropathology of dopaminergic neurons between *Chchd2^{KO}* mice and *CHCHD2^{T61I}* knock-in mice

(A) The limb-clasping reflex was not observed in *Chchd2^{KO}* mice at 30 weeks of age. **(B–D)** Cryosections of the SNpc in *Chchd2^{KO}* mice at 40 weeks of age were immunostained with anti-TH and anti-p-Nefl^{473} antibodies (**B**), the anti-p-α-Syn^{129} antibody **(C)**, and ProteoStat aggresome dye **(D)**. **(E, F)** Nine sections each from the brains of 40-weeks-old mice were immunostained for TH. In **(E)**, representative images of TH staining of the SNpc and VTA are shown. Dashed lines indicate the SNpc and VTA regions. In **(F)**, TH signals in the SNpc were counted. Data are shown as the mean ± SD (n = 3). Comparisons were performed using the unpaired two-tailed Student *t*-test. NS: not significant

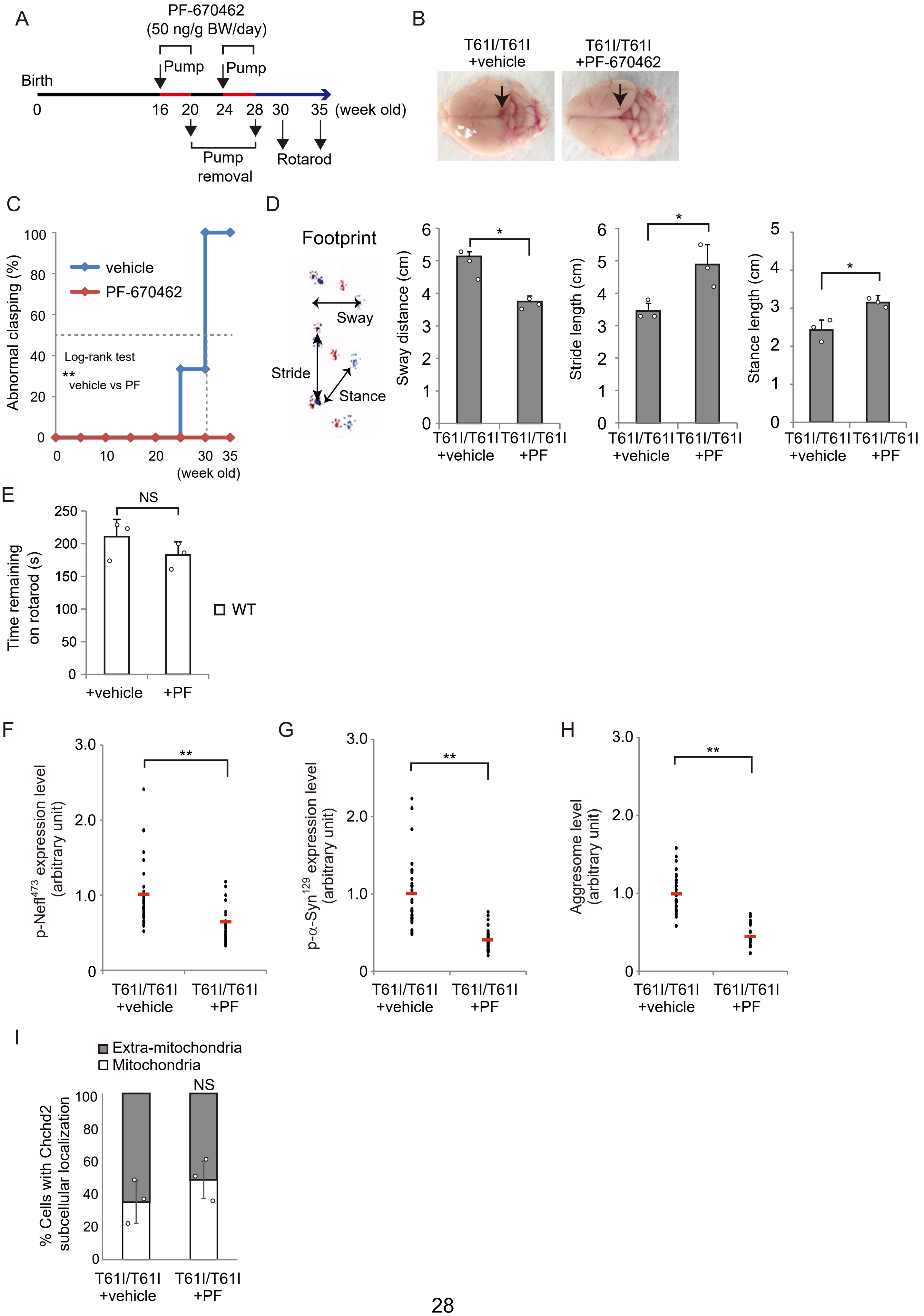
A
PF-670462
(50 ng/g BW/day)
Pump
Pump
Birth
0
16 20 24 28 30 35 (week old)
Pump removal
Rotarod
B
T61I/T61I +vehicle
T61I/T61I +PF-670462
C
Abnormal clasping (%)
vehicle
PF-670462
Log-rank test
** vehicle vs PF
(week old)
D
Footprint
Sway
Stride
Stance
Sway distance (cm)
Stride length (cm)
Stance length (cm)
T61I/T61I +vehicle
T61I/T61I +PF
E
NS
Time remaining on rotarod (s)
WT
+vehicle
+PF
F
p-Nefl473 expression level (arbitrary unit)
G
p-α-Syn129 expression level (arbitrary unit)
H
Aggresome level (arbitrary unit)
I
Extra-mitochondria
Mitochondria
NS
% Cells with Chchd2 subcellular localization

Appendix Fig. S17. Effects of PF-670462 on Chchd2$^{T61I/T61I}$ mice

(A) Schematic diagram of the time course of the osmotic pump experiment. PF-670462 or vehicle was continuously infused into the cerebral cortex just above the SNpc of Chchd2$^{T61I/T61I}$ mice from 16 weeks of age. Mice were analyzed at 30 and 35 weeks of age. **(B)** Representative brains after removal of the olfactory bulbs are shown. Arrows indicate the stereotaxic injection sites (see Material and Methods). **(C)** Quantitative analyses of the data in Fig. 6A are shown. Kaplan-Meier analysis of the abnormal clasping of mice with the indicated genotypes. Comparisons were performed by the log-rank test (vehicle, $n = 3$; PF-670462, $n = 4$). **(D)** Sway, stride, and stance length measured from the footprint analysis are indicated by arrows (left). Quantitative analyses of the footprint analysis data in Fig. 6B are shown (right). Longer sway, shorter stride, and shorter stance indicate lower motor performance. **(E)** No effect of PF-670462 on the motor performance of WT mice. PF-670462 (50 ng/g BW/day) or 5% DMSO was continuously infused into the cerebral cortex of WT mice from 16 weeks of age for 4 weeks. Then, the time that the indicated mice remained on the rotarod was measured. Data are shown as the mean ± SD ($n = 3$ mice). **(F-H)** Quantitative data of Fig. 6F-H ($n = 30$ cells in each experiment). Red bars indicate mean values. **(I)** Quantitative data of Fig. 6I. Population of cells displaying mitochondrial CHCHD2 puncta and extra-mitochondrial CHCHD2 puncta in the SNpc of each mouse ($n \geq 100$ cells in each experiment). Data are shown as the mean ± SD ($n = 3$). In **(D-I)**, comparisons were performed using the unpaired two-tailed Student *t*-tests. $*p < 0.05$; $**p < 0.01$, NS: not significant

Appendix Figure S18

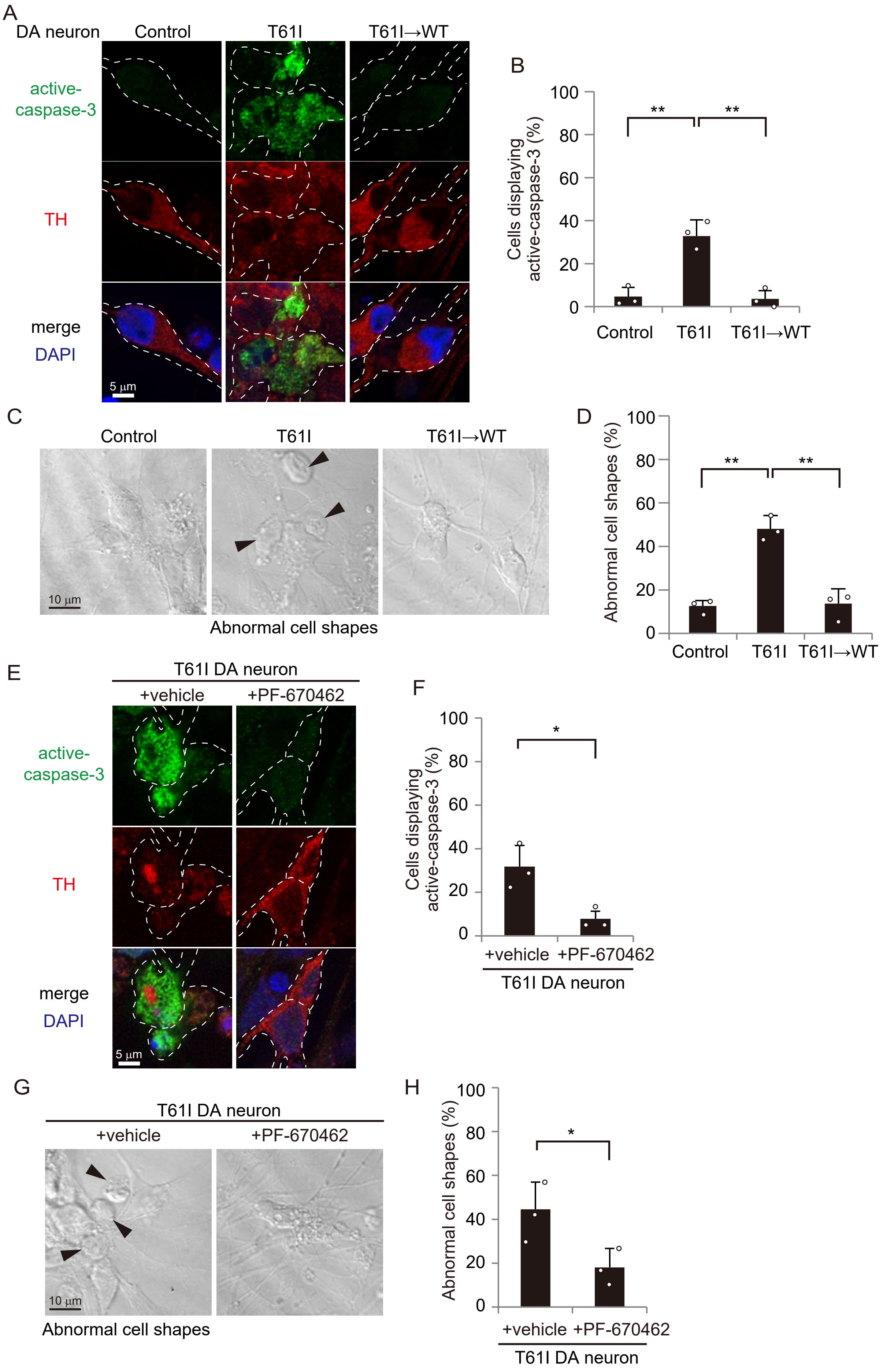

Appendix Fig. S18. Presence of dying dopaminergic neurons generated from patient-derived iPSCs

(A, B) The various types of DA neurons generated from iPSCs were cultured for a few days after differentiation. Then, cells were fixed and stained with anti-active caspase-3 and anti-TH antibodies. In **(A)**, representative images are shown. Dotted lines indicate the cell shape. In **(B)**, quantification of cells with active caspase-3 signals is shown ($n \geq 100$ cells in each experiment). Data are shown as the mean ± SD ($n = 3$ experiments). **(C, D)** The various types of DA neurons generated from iPSCs were cultured for a few days. Then, cells were fixed and observed by differential interference contrast microscopy. Representative images are shown in **(C)**. Arrowheads indicate cells with abnormal round shapes and shortened neurites. In **(D)**, the population of cells with abnormal round shapes was calculated ($n \geq 100$ cells in each experiment). Data are shown as the mean ± SD ($n = 3$). **(E–H)** Similar experiments to **(A–D)** were performed in the presence or absence of PF-670462 for 20 hr. Comparisons were performed using one-way ANOVA followed by the Tukey post-hoc test **(B, D)** and the unpaired two-tailed Student *t*-test **(F, H)**. $^{*}p < 0.05$; $^{**}p < 0.01$

Appendix Table S1. Used primers

Names	Sequences
CHCHD2-P2L-Fw	ATTCGCCACCATGCTGCGTGGAAGCCGAAG
CHCHD2-P2L-Rv	CTTCGGCTTCCACGCAGCATGGTGGCGAAT
CHCHD2-S5R-Fw	ATGCCGCGTGGAAGGCGAAGCCGCACCTCC
CHCHD2-S5R-Rv	GGAGGTGCGGCTTCGCCTTCCACGCGGCAT
CHCHD2-A32T-Fw	GGCCAGCACCAGTCACTCAGCCACCAGCAG
CHCHD2-A32T-Rv	CTGCTGGTGGCTGAGTGACTGGTGCTGGCC
CHCHD2-V66M-Fw	CCACTGCAGCTGGCATGGCTGTGGGCTCTG
CHCHD2-V66M-Rv	CAGAGCCCACAGCCATGCCAGCTGCAGTGG
CHCHD2-I80V-Fw	CATTGGGTCACGCCGTTACTGGGGGCTTCA
CHCHD2-I80V-Rv	TGAAGCCCCCAGTAACGGCGTGACCCAATG
CHCHD2-S85R-Fw	ACTGGGGGCTTCAGAGGAGGAAGTAATGCT
CHCHD2-S85R-Rv	AGCATTACTTCCTCCTCTGAAGCCCCCAGT
human CHCHD2-delta30-Fw	CGCGAATTCGCCACCATGGTCGCTCAGCCAC CA
human CHCHD2-delta52-Fw	CGCGAATTCGCCACCATGCCAGGTCTGATGG CC
human CHCHD2-Rv	CAGCCTCGAGGGCCAATCCGTTTGCAAGTC
CHCHD2 (T61I) transgenic XhoI PCR-Fw	CAGCCTCGAGATGCCGCGTGGAAGCCGAAG
CHCHD2 (T61I) transgenic XhoI PCR-Rv	CAGCCTCGAGTTACATTAAACCATTTGCAA
mouse CHCHD2-T61I-Fw	TGATGGCCCAGATGGCTATCACCGCGGCCG
mouse CHCHD2-T61I-Rw	CGGCCGCGGTGATAGCCATCTGGGCCATCA
pCMV6-cHA-Fw	GGCCGTACCCCTACGACGTGCCCGACTACGC CT
pCMV6-cHA-Rv	AGGCGTAGTCGGGCACGTCGTAGGGGTAC
CHCHD2 (T61I) transgenic Genotyping 1	TGCTCCATTTTGCGTGACTC
CHCHD2 (T61I) transgenic Genotyping 2 (pCMV6-cHA-Rv)	AGGCGTAGTCGGGCACGTCGTAGGGGTAC
CHCHD2 (T61I) Knock-in 5' arm cloning-Fw	GAAGACGAGCCTTGGTAAATATGAGC
CHCHD2 (T61I) Knock-in 5' arm cloning-Rv	ACAAGGGACTATCTTGATGACAGATG
CHCHD2 (T61I) Knock-in 5' arm Hind III-Fw	GATCAAGCTTACAGAATTTTCTTTCTCATA
CHCHD2 (T61I) Knock-in 5' arm Sal I-Fw	GATCGTCGACAAATGGTAGACATCTTGGAC
CHCHD2 (T61I) Knock-in exon2-4 arm cloning-Fw	TGTGGTAAACGATCTATTGAGCTACC
CHCHD2 (T61I) Knock-in exon2-4 arm cloning-Rv	AGGATAGGCCATTTACACAGATCTGG
CHCHD2 (T61I) Knock-in exon2-4 arm Sal I-Fw	GATCGTCGACTGTGGTAAACGATCTATTGAG

CHCHD2 (T61I) Knock-in exon2-4 arm Mlu I-Rv	GATCACGCGTGAATACTAGACAAGCCTGCTG
CHCHD2 (T61I) Knock-in 3' arm cloning- Xba I-Mlu I-Fw	GATCTCTAGAACGCGTACCAGGCTTGAGTCCT GTC
CHCHD2 (T61I) Knock-in 3' arm cloning-Xba I-Rv	GATCTCTAGAGGTAGATCTTTTGAGTTTGAGT G
CHCHD2 (T61I) Knock-in Genotyping for 9,000 bp-Fw	GTGGGCATCTCTGCATTTGAGCAG
CHCHD2 (T61I) Knock-in Genotyping for 9,000 bp-Rw	CAAATGGCTCAGAGGAGACAAAGC
CHCHD2 (T61I) Knock-in Genotyping 5' long-Fw	GCATCTCTGCATTTGAGCAG
CHCHD2 (T61I) Knock-in Genotyping 5' long-Rv	GGTGATATTAGGAATGCAGTCC
CHCHD2 (T61I) Knock-in Genotyping 3' long-Fw	GCCACCACACTAGCTTTGTTG
CHCHD2 (T61I) Knock-in Genotyping 3' long-Rv	GAACTACATCAGGGGTGACAC

Appendix Table S2. Antibody list

name	clone	Campany	Cat. Number	method	dilution
HA-Tag	F-7	Santa Cruz	sc-7392	WB, IF	1:200, 1:100
Anti-HA-tag pAb		MBL	561	IF	1:100
COX IV 3E11		Cell Signaling	4850	WB	1:500
α-Tubulin	DM1A	Sigma-Aldrich	T9026	WB	1:3,000
Tom20		Santa Cruz	sc-11415	IF	1:200
Anti-Tyrosine Hydroxylase Antibody		Merck Millipore	AB152	WB, IF	1:500, 1:100
Anti-Tyrosine Hydroxylase Antibody	LNC1	Merck Millipore	AB318	IF	1:100
CHCHD2 Antibody		Proteintech	19424-1-AP	WB, IF	1:200, 1:100
Anti-ANT1/2	5F51B B5AG7	Abcam	Ab110322	IF	1:50
Anti-68kDa		Abcam	ab24520	IF	1:100
Neurofilament-L	C28E1	Cell Signaling Tec.	2837T	WB	1:200
Anti-phospho-NFL (NEFL) (Ser473) Antibody mouse	4F8	Merck Millipore	MABN2431	WB, IF	1:200, 1:200
Synuclein	AB_39 8107	BD	610787	WB	1:500
Anti-phosphorylated a-Synuclein pSyn	#64	Wako FUJFILM	015-25191	IF	1:200
casein kinase I epsilon	A-2	Santa Cruz	sc-373912	WB, IF	1:200, 1:100
Anti-Caspase-3,Cleaved(Asp175)	# 269518	R&D systems	MAB835	IF	1:200
Anti-Calbindin D-28k	300	Swant	CB300	IF	1:400
Anti-Fox3 (NeuN)	1B7	BioLegend	834501	IF	1:100
