# Supplementary material for: Involvement of casein kinase 1 epsilon/delta (Csnk1e/d) in the pathogenesis of familial Parkinson's disease caused by CHCHD2
Source: EMBO Mol Med. 2023 Aug 14;15(9):e17451. doi: 10.15252/emmm.202317451 (PMC10493588; doi:10.15252/emmm.202317451)
Supplement: Supplementary file 2 — Expanded View Figures PDF [file EMMM-15-e17451-s009.pdf]

Expanded View Figures

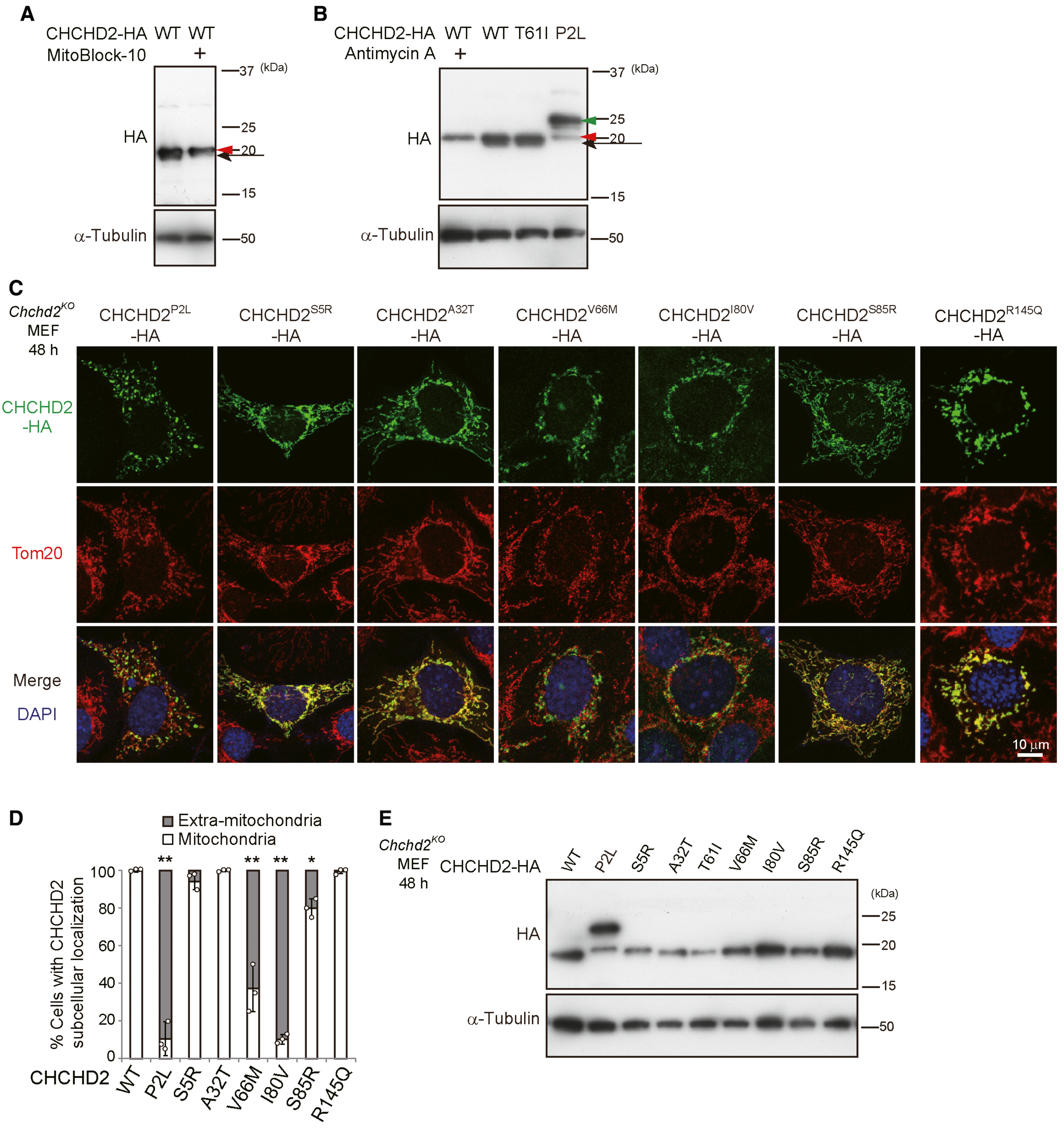

Figure EV1.

◀ **Figure EV1. Expression of pathogenic mutant CHCHD2 proteins.**

A, B Effects of mitochondrial protein import inhibitors on Western blot band sizes of CHCHD2 mutant proteins. *Chchd2^{KO}* cells were transfected with the *CHCHD2^{WT}-HA*, *CHCHD2^{T61I}-HA*, and *CHCHD2^{P2L}-HA* plasmids, and treated with compounds that inhibit mitochondrial protein import (MitoBlock-10 [20 μM] and antimycin A [10 μM]) for 16 h. At 48 h after transfection, cell lysates were subjected to Western blotting. Addition of these compounds altered the band size of CHCHD2^{WT} from 18 kD (black arrows) to 20 kD (red arrowheads). The band size of CHCHD2^{T61I} and CHCHD2^{P2L} is 18 kD (black arrows) and 20 kD (red arrowheads) plus 23 kD (green arrowhead), respectively.

C–E The indicated pathogenic mutant CHCHD2 proteins were expressed in *Chchd2^{KO}* MEFs by gene transfection. After 48 h, cells were permeabilized and stained with anti-HA and anti-Tom20 antibodies (C, D), or cells were collected and lysates were subjected to Western blotting using an anti-HA antibody (E). In (C), representative images are shown. In (D), the number of cells displaying mitochondrial CHCHD2 and extra-mitochondrial CHCHD2 puncta were quantified ($n \geq 100$ cells in each experiment). Data are shown as the mean ± SD ($n = 3$).

 © 2023 The Authors

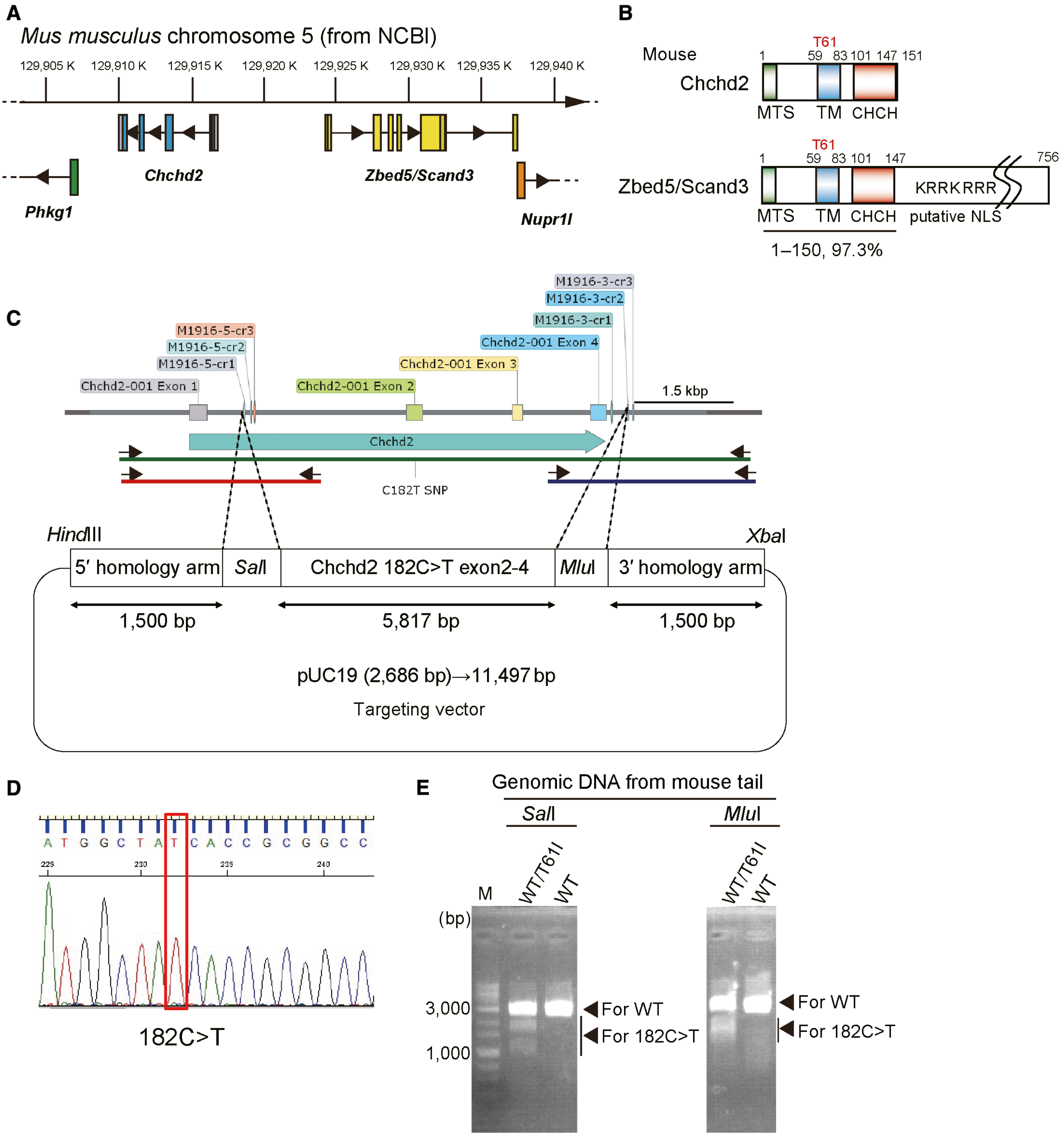
A
Mus musculus chromosome 5 (from NCBI)
129,905 K
129,910 K
129,915 K
129,920 K
129,925 K
129,930 K
129,935 K
129,940 K
Chchd2
Zbed5/Scand3
Phkg1
Nupr1l
B
Mouse
Chchd2
T61
1
59
83
101
147
151
MTS
TM
CHCH
Zbed5/Scand3
T61
1
59
83
101
147
756
KRRKRRR
MTS
TM
CHCH
putative NLS
1–150, 97.3%
C
M1916-3-cr3
M1916-3-cr2
M1916-3-cr1
Chchd2-001 Exon 4
M1916-5-cr3
M1916-5-cr2
M1916-5-cr1
Chchd2-001 Exon 3
Chchd2-001 Exon 1
Chchd2-001 Exon 2
1.5 kbp
Chchd2
C182T SNP
HindIII
XbaI
5′ homology arm
SalI
Chchd2 182C>T exon2-4
MluI
3′ homology arm
1,500 bp
5,817 bp
1,500 bp
pUC19 (2,686 bp)→11,497 bp
Targeting vector
D
A T G G C T A T C A C C G C G G C C
225
230
235
240
182C>T
E
Genomic DNA from mouse tail
SalI
MluI
M
WT/T61I
WT
WT/T61I
WT
(bp)
3,000
1,000
For WT
For 182C>T
For WT
For 182C>T

Figure EV2.

Figure EV2. Generation of Chchd2^{T61I} knock-in mice.

A Schematic representation of the gene map of *Mus musculus* chromosome 5.
B Structures of the Chchd2 and Zbed5/Scand3 proteins. The protein sequence of Chchd2 is very similar (97.3%) to that of the N-terminal domain of Zbed5/Scand3.
C Schematic representation of the targeting vector and targeted allele of the *Chchd2* gene. The 5.8-kb region of the mouse *Chchd2* gene, including exons 2–4, was recombined with the C182T SNP-mutated sequence. A 1.5-kb 5′ fragment and a 1.5-kb 3′ fragment were used as the homologous arms. Small arrows indicate the position of the primers used for genotyping PCR. The green line indicates the PCR product used for sequencing. Red and blue lines indicate PCR products for *Sal* I and *Mlu* I, respectively.
D Genotyping was performed by genome amplification of by PCR (green line in (C), about 9,000 bp including a modified genome sequence) from tail genomic DNA followed by sequencing.
E Genotyping was also performed by genome amplification by PCR (red and blue lines in (C), about 3,000 bp) followed by digestion with the respective restriction enzymes. M indicates a lane of DNA markers. The primers used are listed in Appendix Table S1.

Figure EV3. Observation of inclusion bodies and cell death in DA neurons of Chchd2$^{T61I/T61I}$ knock-in mice by EM.

A–C Sections of DA neurons in the SNpc of CHCHD2$^{T61I/T61I}$ knock-in mice. Magnified images of the area within the pink square in the top panels are shown in the middle panels, and those of the orange squares in the middle panels are shown in the bottom panels. (A, B) DA neurons contained an inclusion body (Inc). The nucleus (N), mitochondria (Mt), and Golgi (G) appeared almost normal. (C) A dying neuron containing an inclusion body (Inc) is shown. The nucleus appears shrunken, and mitochondria and Golgi are abnormally swollen. A low-magnified image is shown in Fig 5M.
D A normal DA neuron in the SNpc of a littermate WT littermate mouse is shown. A magnified image of the area within the pink square is shown in the bottom panel.

 © 2023 The Authors

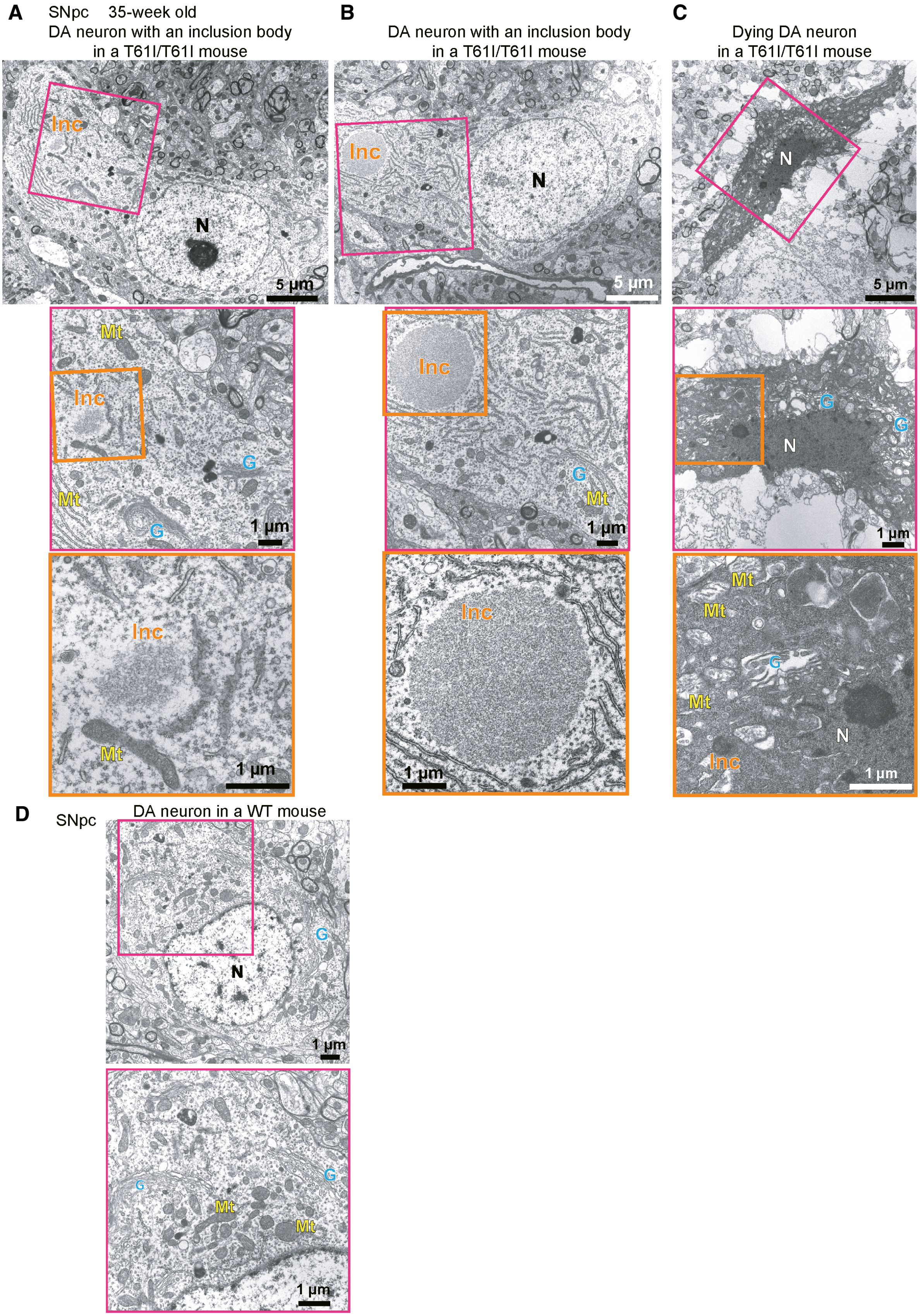
A
SNpc 35-week old
DA neuron with an inclusion body
in a T61I/T61I mouse
Inc
N
5 μm
Mt
Inc
G
Mt
G
1 μm
Inc
Mt
1 μm
B
DA neuron with an inclusion body
in a T61I/T61I mouse
Inc
N
5 μm
Inc
G
Mt
1 μm
Inc
1 μm
C
Dying DA neuron
in a T61I/T61I mouse
N
5 μm
G
G
N
1 μm
Mt
Mt
G
Mt
N
Inc
1 μm
D
SNpc
DA neuron in a WT mouse
G
N
1 μm
G
G
Mt
Mt
1 μm

Figure EV3.

Figure EV4. Reduced motor performance and dopaminergic neuronal loss in Chchd2^{T61I} Tg mice.

A The limb-clasping reflex was observed in Chchd2^{T61I} Tg mice at 30 weeks of age. Quantitative analysis is shown in Appendix Fig S13A.

B Midbrain and diencephalon brain lysates were obtained from WT and Tg mice, and expression of the indicated proteins was analyzed by Western blotting. Quantitative analysis of protein expression is shown in Appendix Fig S13C.

C, D Abnormal motor performance in Chchd2^{T61I} Tg mice. In (C), the footprint assay indicated motor deficits at 35 weeks. Quantitative analysis is shown in Appendix Fig S13B. In (D), the time that the indicated mice remained on the rotarod was measured. Data are shown as the mean ± SD (n = 4).

E–G Reduction in TH signals in the SNpc of Tg mice. In (E), brain cryosections were immunostained with the dopaminergic cell marker TH. Representative images of the SNpc and VTA are shown. Dashed lines indicate the SNpc and VTA regions. Bars = 200 μm. In (F), TH signals in the SNpc (average fluorescence intensity per region) are shown as the mean ± SD (n = 3). In (G), cryosections of the SNpc were immunostained with anti-HA and anti-TH antibodies. Arrowheads indicate Chchd2^{T61I}-HA puncta in TH-positive cells. Dashed lines indicate cell shapes.

H–L Extra-mitochondrial aggresome formation by p-Nefl^{473}, p-α-Syn^{129}, and Csnk1e/d in the SNpc of Tg mice. Cryosections of the SNpc were immunostained with anti-HA and anti-Ant1/2 (H), anti-p-Nefl^{473} (I), and anti-p-α-Syn^{129} antibodies (J), ProteoStat dye (K), and anti-Csnk1e/d antibodies (L). Arrowheads indicate extra-mitochondrial Chchd2^{T61I}-HA (H). In (I–L), arrowheads indicated the colocalization of puncta with Chchd2^{T61I}-HA and the indicated proteins. Dashed lines indicate cell shapes. Quantitative data are shown in Appendix Fig S13D–F.

M EM analysis of dopaminergic neurons in the SNpc of Chchd2^{T61I}-HA Tg mice. A dopaminergic neuron with an abnormal nuclear/cell shape and an inclusion body is shown. The red arrowhead indicates an inclusion body. N: nucleus. A magnified image of this cell is shown in Appendix Fig S14. In (D, F), comparisons were performed using unpaired two-tailed Student t-test. *$P < 0.05$; **$P < 0.01$.

© 2023 The Authors

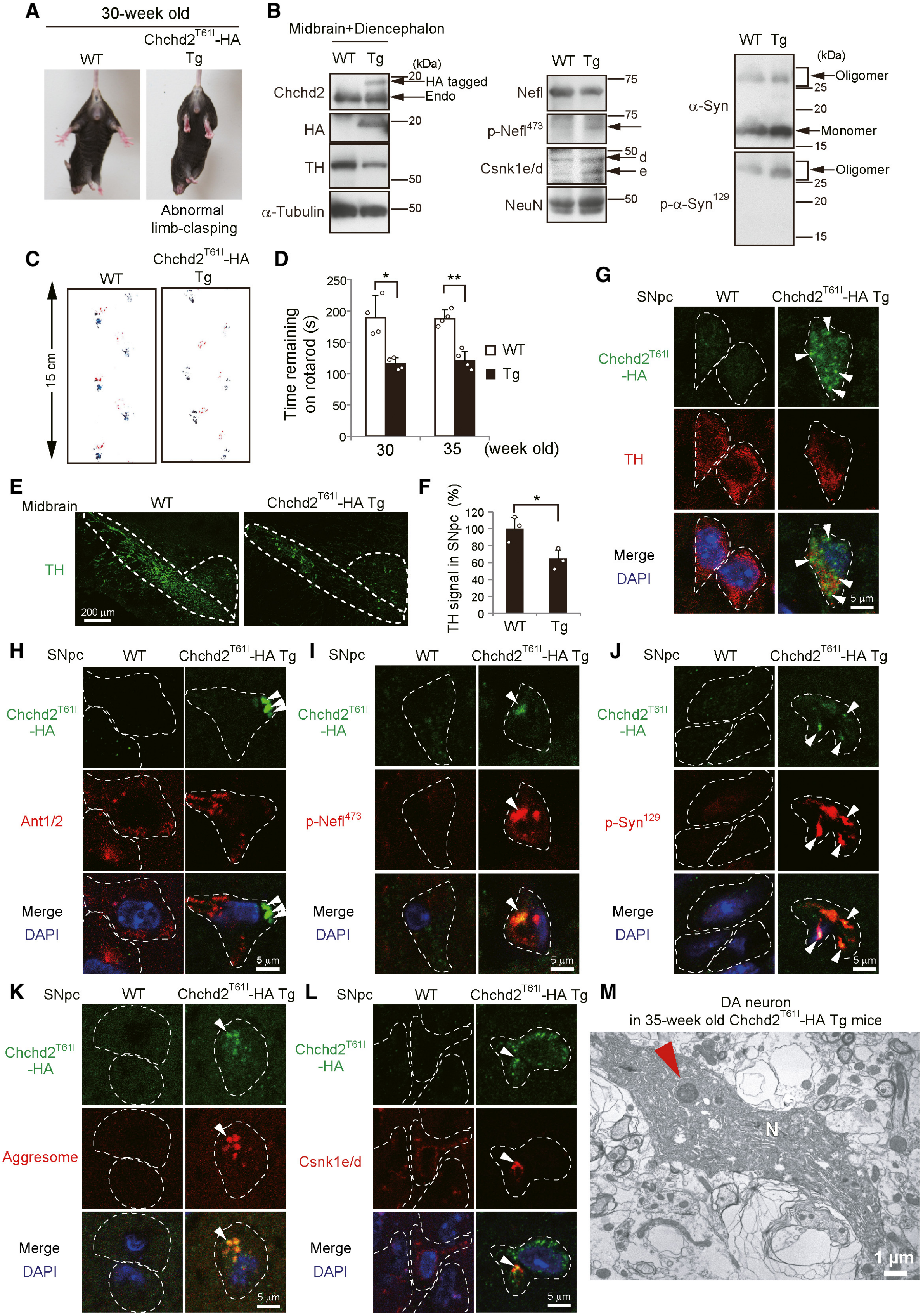
A
30-week old
Chchd2T61I-HA
WT
Tg
Abnormal limb-clasping
B
Midbrain+Diencephalon
WT Tg
(kDa)
Chchd2
HA tagged
Endo
HA
TH
α-Tubulin
Nefl
p-Nefl473
Csnk1e/d
NeuN
α-Syn
Oligomer
Monomer
p-α-Syn129
C
15 cm
D
Time remaining on rotarod (s)
WT
Tg
30
35
(week old)
E
Midbrain
Chchd2T61I-HA Tg
TH
200 µm
F
TH signal in SNpc (%)
G
SNpc
Chchd2T61I -HA
Merge
DAPI
5 µm
H
Ant1/2
I
p-Nefl473
J
p-Syn129
K
Aggresome
L
Csnk1e/d
M
DA neuron in 35-week old Chchd2T61I-HA Tg mice
N
1 µm

Figure EV4.

Figure EV5. Analysis of dopaminergic neurons generated from patient-derived iPSCs.

A Schematic design of the guide RNA (gRNA) and the oligo donor for making isogenic control iPSC lines from patient-derived iPSCs. A mutation of thymine in both the edited allele and gRNA and the recovered cytosine in the oligo donor are shown in red. The two additional synonymous mutations that were used to introduce a *de novo* SacII site into the oligo donor are shown in green.

B Cultured DA neurons from iPSCs were observed by differential interference contrast microscopy. DA neurons from non-patient iPSCs (Control) and gene-corrected CHCHD2^{T61I} iPSCs (T61I-WT) looked healthy with neurite elongation, whereas those from CHCHD2^{T61I} iPSCs (T61I) were shrunken and without neurite elongation.

C DA neurons of each type were fixed and stained with anti-CHCHD2 and anti-TH antibodies. Dashed lines indicate cell shapes, and arrowheads indicate abnormal CHCHD2^{T61I} puncta.

D Quantification of cells displaying mitochondrial CHCHD2 puncta and extra-mitochondrial CHCHD2 puncta ($n \geq 100$ cells in each experiment). Data are shown as the mean ± SD ($n = 3$).

E–G Quantitative data of Fig 8C–E ($n = 30$ cells in each experiment). Red bars indicate mean values.

H–J Quantitative data of Fig 8F–H ($n = 30$ cells in each experiment). Red bars indicate mean values.

K, L PF-670462 has no effect on the extra-mitochondrial localization of CHCHD2^{T61I}. DA neurons from CHCHD2^{T61I} iPSCs were treated with PF-670462 for 20 h. Then, cells were fixed and stained with an anti-CHCHD2 and anti-ANT1/2 antibodies. In (K), representative images are shown. Dashed lines indicate cell shapes, and arrowheads indicate extra-mitochondrial CHCHD2^{T61I} puncta. (L) Quantification of cells displaying mitochondrial CHCHD2 and extra-mitochondrial CHCHD2 puncta ($n \geq 100$ cells in each experiment). Data are shown as the mean ± SD ($n = 3$). Comparisons were performed using one-way ANOVA followed by the Tukey *post hoc* test (D–G) or unpaired two-tailed Student *t*-test (H–J, L). $^*P < 0.05$; $^{**}P < 0.01$; NS: not significant.

 © 2023 The Authors

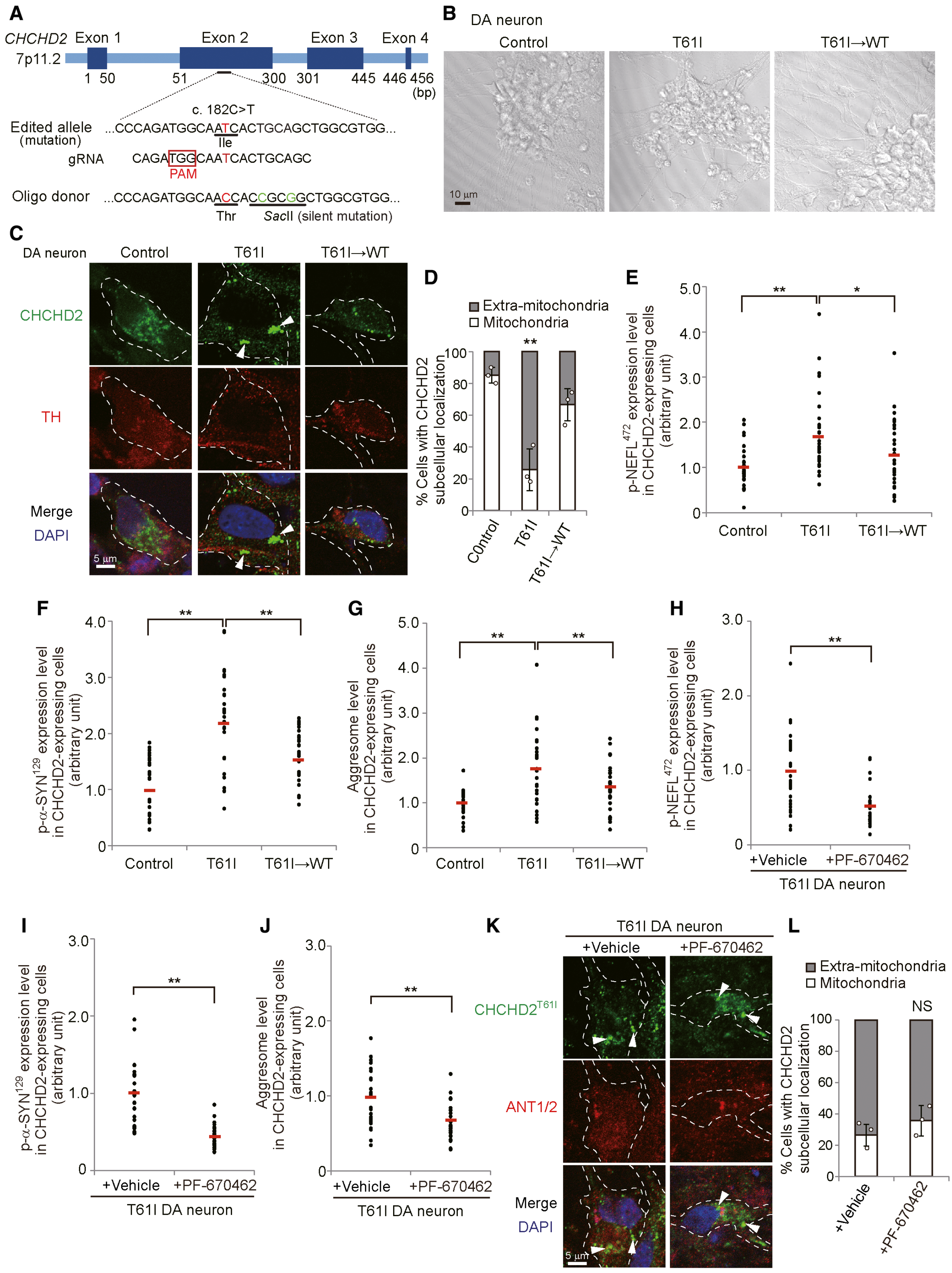
A
CHCHD2 Exon 1 Exon 2 Exon 3 Exon 4
7p11.2
1 50 51 300 301 445 446 456 (bp)
c. 182C>T
Edited allele (mutation) ...CCCAGATGGCAATCACTGCAGCTGGCGTGG...
Ile
gRNA CAGATGGCAATCACTGCAGC
PAM
Oligo donor ...CCCAGATGGCAACCACCGCGGCTGGCGTGG...
Thr SacII (silent mutation)
B
DA neuron
Control T61I T61I→WT
10 μm
C
DA neuron Control T61I T61I→WT
CHCHD2
TH
Merge
DAPI
5 μm
D
Extra-mitochondria
Mitochondria
% Cells with CHCHD2 subcellular localization
Control T61I T61I→WT
E
p-NEFL472 expression level in CHCHD2-expressing cells (arbitrary unit)
Control T61I T61I→WT
F
p-α-SYN129 expression level in CHCHD2-expressing cells (arbitrary unit)
Control T61I T61I→WT
G
Aggresome level in CHCHD2-expressing cells (arbitrary unit)
Control T61I T61I→WT
H
p-NEFL472 expression level in CHCHD2-expressing cells (arbitrary unit)
+Vehicle +PF-670462
T61I DA neuron
I
p-α-SYN129 expression level in CHCHD2-expressing cells (arbitrary unit)
+Vehicle +PF-670462
T61I DA neuron
J
Aggresome level in CHCHD2-expressing cells (arbitrary unit)
+Vehicle +PF-670462
T61I DA neuron
K
T61I DA neuron
+Vehicle +PF-670462
CHCHD2T61I
ANT1/2
Merge
DAPI
5 μm
L
Extra-mitochondria
Mitochondria
NS
% Cells with CHCHD2 subcellular localization
+Vehicle +PF-670462

Figure EV5.
