# Supplementary material for: Involvement of casein kinase 1 epsilon/delta (Csnk1e/d) in the pathogenesis of familial Parkinson's disease caused by CHCHD2
Source: EMBO Mol Med. 2023 Aug 14;15(9):e17451. doi: 10.15252/emmm.202317451 (PMC10493588; doi:10.15252/emmm.202317451)
Supplement: Supplementary file 3 — PDF+ [file EMMM-15-e17451-s001.pdf]

Article

SOURCE DATA | TRANSPARENT PROCESS | OPEN ACCESS

EMBO Molecular Medicine

Involvement of casein kinase 1 epsilon/delta (Csnk1e/d) in the pathogenesis of familial Parkinson's disease caused by CHCHD2

Satoru Torii[1,*], Satoko Arakawa[1], Shigeto Sato[2], Kei-ichi Ishikawa[2,3], Daisuke Taniguchi[2], Hajime Tajima Sakurai[1], Shinya Honda[1], Yuuichi Hiraoka[4,5], Masaya Ono[6], Wado Akamatsu[3], Nobutaka Hattori[2] & Shigeomi Shimizu[1,**]

Abstract

Parkinson's disease (PD) is a common neurodegenerative disorder that results from the loss of dopaminergic neurons. Mutations in coiled-coil-helix-coiled-coil-helix domain containing 2 (CHCHD2) gene cause a familial form of PD with α-Synuclein aggregation, and we here identified the pathogenesis of the T61I mutation, the most common disease-causing mutation of CHCHD2. In Neuro2a cells, CHCHD2 is in mitochondria, whereas the T61I mutant ($CHCHD2^{T61I}$) is mislocalized in the cytosol. $CHCHD2^{T61I}$ then recruits casein kinase 1 epsilon/delta (Csnk1e/d), which phosphorylates neurofilament and α-Synuclein, forming cytosolic aggresomes. *In vivo*, both $Chchd2^{T61I}$ knock-in and transgenic mice display neurodegenerative phenotypes and aggresomes containing $Chchd2^{T61I}$, Csnk1e/d, phospho-α-Synuclein, and phospho-neurofilament in their dopaminergic neurons. Similar aggresomes were observed in a postmortem PD patient brain and dopaminergic neurons generated from patient-derived iPS cells. Importantly, a Csnk1e/d inhibitor substantially suppressed the phosphorylation of neurofilament and α-Synuclein. The Csnk1e/d inhibitor also suppressed the cellular damage in $CHCHD2^{T61I}$-expressing Neuro2a cells and dopaminergic neurons generated from patient-derived iPS cells and improved the neurodegenerative phenotypes of $Chchd2^{T61I}$ mutant mice. These results indicate that Csnk1e/d is involved in the pathogenesis of PD caused by the $CHCHD2^{T61I}$ mutation.

Keywords CHCHD2; Csnk1e/d; α-Synuclein
Subject Categories Genetics, Gene Therapy & Genetic Disease; Molecular Biology of Disease; Neuroscience
DOI 10.15252/emmm.202317451 | Received 18 January 2023 | Revised 4 July 2023 | Accepted 5 July 2023 | Published online 14 August 2023
EMBO Mol Med (2023) 15: e17451

Introduction

Parkinson's disease (PD) is the second most common neurodegenerative disorder and is characterized by progressive resting tremors, rigidity, bradykinesia, gait disturbances, postural instability, and dementia (Exner *et al*, 2012; Imai *et al*, 2019; Torii *et al*, 2020a). The symptoms of PD are strongly linked to the degeneration of dopaminergic neurons in the substantia nigra pars compacta (SNpc). Approximately 10% of PD patients have familial forms of the disease, and about 20 genomic regions (called *PARK*) have been identified as loci containing disease-associated mutations. Among them, the gene encoding coiled-coil-helix-coiled-coil-helix (CHCH) domain containing 2 (CHCHD2) was identified as the gene responsible for PARK22 (Funayama *et al*, 2015).

CHCHD2 is a protein containing a mitochondrial-targeting sequence (MTS) in the N-terminus, followed by a putative transmembrane domain, and a conserved CHCH domain at the C-terminus (Funayama *et al*, 2015; Imai *et al*, 2019; Torii *et al*, 2020a; Kee *et al*, 2021). The CHCH domain consists of two CX(9)C motifs and two disulfide bonds that stabilize the CHCH fold. CHCHD2 is localized in the intermembrane space of mitochondria, and interacts with cytochrome *c* and MICS1, a member of the Bax inhibitor-1 superfamily, to regulate oxidative phosphorylation (Meng *et al*, 2017). It was also reported to interact with Bcl-x_L to suppress apoptosis (Liu *et al*, 2015; Cornelissen *et al*, 2020). However, the effects of *CHCHD2* deletion on oxidative phosphorylation and apoptosis are marginal, and hence its precise biological roles have remained unclear.

Various mutations in the *CHCHD2* gene have been linked to various neuronal diseases, i.e., 182C>T (T61I), 434G>A (R145Q), and 300+5G>A in familial PD, P2L, R8H, and A71P in sporadic PD, V66M in multiple system atrophy, and P2L, S5R, A32T, and S85R in

1 Department of Pathological Cell Biology, Medical Research Institute, Tokyo Medical and Dental University (TMDU), Tokyo, Japan
2 Department of Neurology, School of Medicine, Juntendo University, Tokyo, Japan
3 Center for Genomic and Regenerative Medicine, School of Medicine, Juntendo University, Tokyo, Japan
4 Laboratory of Molecular Neuroscience, Medical Research Institute, Tokyo Medical and Dental University (TMDU), Tokyo, Japan
5 Laboratory of Genome Editing for Biomedical Research, Medical Research Institute, Tokyo Medical and Dental University (TMDU), Tokyo, Japan
6 Department of Clinical Proteomics, National Cancer Center Research Institute, Tokyo, Japan
*Corresponding author. Tel: +81 3 5803 4797; Fax: +81 3 5803 4821; E-mail: toripcb@tmd.ac.jp
**Corresponding author. Tel: +81 3 5803 4797; Fax: +81 3 5803 4821; E-mail: shimizu.pcb@mri.tmd.ac.jp

© 2023 The Authors. Published under the terms of the CC BY 4.0 license

Alzheimer's disease and frontotemporal dementia (Imai *et al*, 2019; Kee *et al*, 2021). Among these mutations, evidence for pathogenicity is the strongest for the T61I missense mutation, which was identified in three independent Japanese and Chinese families with dominant PD. Patients with this mutation exhibit symptoms such as resting tremor and bradykinesia (Imai *et al*, 2019; Kee *et al*, 2021). Regarding pathogenesis, biochemical analysis demonstrated the instability of the T61I mutant protein, leading to its localization in the insoluble fraction (Meng *et al*, 2017; Huang *et al*, 2018; Ikeda *et al*, 2019; Cornelissen *et al*, 2020). This mutant protein was also reported to increase the production of mitochondrial reactive oxygen species and susceptibility to apoptosis (Cornelissen *et al*, 2020). Analysis of autopsied brains of patients, dopaminergic neurons generated from patient-derived iPS cells (iPSC), and PD model flies suggested the intracellular mislocalization of CHCHD2^{T61I} and the increased phosphorylation of α-Synuclein (Ikeda *et al*, 2019), although its pathogenesis remains unclear.

To investigate the pathogenesis of CHCHD2^{T61I}-induced PD, we performed cell biological analyses as well as genetic analyses by generating *Chchd2^{T61I}* knock-in mice and transgenic (Tg) mice. These mice demonstrated abnormalities in motor performance and dopaminergic neuronal loss in the SNpc. We found that Chchd2^{T61I} is mislocalized to outside of mitochondria, where casein kinase 1 epsilon (Csnk1e) and its paralogue casein kinase 1 delta (Csnk1d) were recruited. The mislocalized Csnk1e/d phosphorylates neurofilament and α-Synuclein, resulting in the formation of aggresomes in dopaminergic neurons of the SNpc. Consistent pathology was observed in both the postmortem brain of a CHCHD2^{T61I} PD patient and iPSC-derived dopaminergic neurons with the *CHCHD2^{T61I}* mutation. Importantly, pharmacological inhibition of Csnk1e/d substantially improved the neurodegenerative phenotypes and neuronal pathology of *Chchd2^{T61I}* mutant mice and also suppressed cellular damage in iPSC-derived dopaminergic neurons with the *CHCHD2^{T61I}* mutation. These data demonstrate the involvement of Csnk1e/d in the pathogenesis of CHCHD2^{T61I}-induced PD.

Results

In this study, we investigated the pathogenesis of the T61I mutation of CHCHD2, which is a mutation that causes familial PD (Funayama *et al*, 2015). For this purpose, we first expressed wild-type human CHCHD2 (CHCHD2^{WT}) and the T61I mutant (CHCHD2^{T61I}) in mouse embryonic fibroblasts (MEFs) isolated from *Chchd2*-deficient mice (*Chchd2^{KO}*) (Appendix Fig S1A–C). As expected from the presence of a MTS, CHCHD2^{WT} was almost completely localized in mitochondria, as judged from its colocalization with Tom20, an outer mitochondrial membrane protein (Fig 1A and B, Appendix Fig S1D). This localization of CHCHD2 is consistent with previous reports (Funayama *et al*, 2015; Imai *et al*, 2019; Kee *et al*, 2021). In contrast, for CHCHD2^{T61I}, we observed two types of cells; i.e., cells with mitochondria-localized CHCHD2^{T61I} and cells with extra-mitochondrial CHCHD2^{T61I} (Fig 1A and B, Appendix Fig S1D), and the population of the latter increased in a time-dependent manner after gene transfection (Fig 1C). There are two possible mechanisms that may result in increased extra-mitochondrial localization of CHCHD2^{T61I} despite the presence of an N-terminal MTS: it can either enter the mitochondria and then be released into the cytoplasm, or it can be localized in the cytoplasm without entering the mitochondria. The height of the protein band on Western blotting would be able to determine which is correct. In fact, when cells expressing CHCHD2^{WT} were treated with compounds that inhibit mitochondrial import (antimycin A and MitoBlock-10), CHCHD2^{WT} did not enter the mitochondria and an abnormal 20-kD band appeared instead of the normal 18-kD band (Fig EV1A and B). On the other hand, for CHCHD2^{T61I} we observed an 18-kD band (Fig EV1B), indicating that CHCHD2^{T61I} is first imported into mitochondria and then is subsequently removed from mitochondria. Among the various disease-associated mutants of CHCHD2, the P2L, V66M, and I80V mutants, and a fraction of the S85R mutant demonstrated a similar extra-mitochondrial localization (Fig EV1C–E). Note that P2L, which is a mutation in the MTS region, was detected mainly as a 23-kD protein, as well as a 20-kD proteins (Fig EV1B and E).

Localization analysis was also performed using Neuro2a cells that were differentiated into neurons by 2% FBS plus retinoic acid. As indicated in Fig 1D and E, Appendix Fig S1E, we observed that CHCHD2^{T61I} was expressed outside of the mitochondria in many cells at 28 h after transfection. Consistently, cell fractionation analysis showed that most of the CHCHD2^{WT} was recovered in the mitochondria-containing organellar fraction, whereas the amount of CHCHD2^{T61I} in this fraction was greatly reduced and was mostly recovered in the insoluble fraction (Fig 1F and G). Similar results were obtained when a different fractionation procedure was applied (Appendix Fig S1F and G). These results indicate that CHCHD2^{T61I} is prone to removal from mitochondria.

We next analyzed whether aggregates of misfolded proteins with a beta-sheet structure are generated in differentiated Neuro2a cells expressing CHCHD2^{T61I}, because aggresomes are frequently observed in the neurons of PD patients harboring the CHCHD2^{T61I} mutation. As expected, multiple aggresomes were detected in differentiated Neuro2a cells expressing extra-mitochondrial CHCHD2^{T61I}, but not those expressing CHCHD2^{WT}, as assessed by ProteoStat protein aggregation dye (Fig 2A and B, Appendix Fig S2A). Notably, aggresome signals were almost completely colocalized with CHCHD2^{T61I} (Fig 2A, Appendix Fig S2A), but not mitochondria (Appendix Fig S2B). Furthermore, aggresomes were not generated in cells with mitochondria-localized CHCHD2^{T61I} (Appendix Fig S2C). However, when we analyzed the CHCHD2$^{\Delta52}$ mutant lacking the MTS comprising the N-terminal 52 amino acids, it was localized completely outside of the mitochondria, formed puncta, and generated aggresomes (Appendix Fig S2D–F), suggesting that the extra-mitochondrial localization of CHCHD2 is crucial for the generation of aggresomes.

Phosphorylation of Nefl and α-Synuclein in cells expressing CHCHD2^{T61I}

Neurofilament light chain (Nefl, also called Nfl/Nf68) is a subunit of neurofilament, which is an abundant cytoskeletal protein in axons. Because decreased Nefl expression and its excessive phosphorylation (at Ser^{473} in mouse Nefl, which corresponds to Ser^{472} in human NEFL; Hill *et al*, 1991; Basso *et al*, 2004) are biomarkers of axonal damage in PD patients, we analyzed these molecular alterations. As expected, we observed these changes in Neuro2a cells expressing CHCHD2^{T61I}, but not those expressing CHCHD2^{WT} (Fig 2C and D, Appendix Fig S3A (Nefl), Fig 2E and F and Appendix Fig S3B (p-Nefl)). *Snca*

 © 2023 The Authors

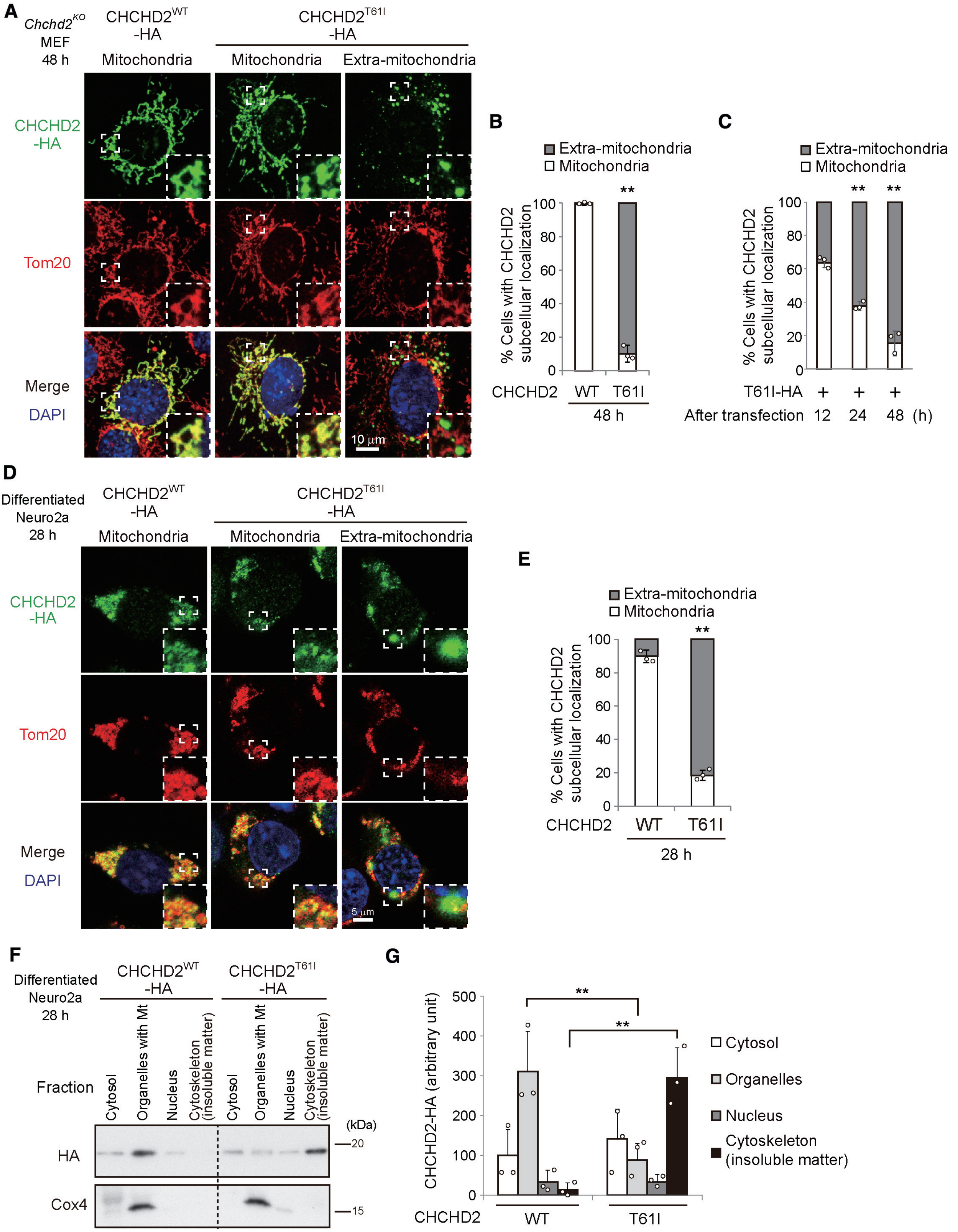
A
Chchd2KO MEF 48 h
CHCHD2WT -HA
CHCHD2T61I -HA
Mitochondria
Extra-mitochondria
CHCHD2 -HA
Tom20
Merge
DAPI
10 μm
B
Extra-mitochondria
Mitochondria
% Cells with CHCHD2 subcellular localization
CHCHD2 WT T61I
48 h
C
T61I-HA + + +
After transfection 12 24 48 (h)
D
Differentiated Neuro2a 28 h
5 μm
E
28 h
F
Fraction
Cytosol
Organelles with Mt
Nucleus
Cytoskeleton (insoluble matter)
(kDa)
HA
Cox4
20
15
G
CHCHD2-HA (arbitrary unit)
Cytosol
Organelles
Nucleus
Cytoskeleton (insoluble matter)
CHCHD2 WT T61I

Figure 1.

Figure 1. Extra-mitochondrial puncta formation of the CHCHD2^{T61I} protein.

A–C Extra-mitochondrial puncta formation of CHCHD2^{T61I} in MEFs. *Chchd2^{KO}* cells were transfected with the *CHCHD2^{WT}-HA* and *CHCHD2^{T61I}-HA* plasmids. At the indicated times, cells were fixed and stained with anti-HA and anti-Tom20 antibodies, and observed by confocal microscopy. In (A), representative images are shown. Magnified images of the areas within the dashed squares are shown in the insets. Representative images at lower magnification are shown in Appendix Fig S1D. (B, C) Quantification of cells displaying mitochondrial CHCHD2 and extra-mitochondrial CHCHD2 puncta ($n \geq 100$ cells in each experiment).

D, E Extra-mitochondrial puncta formation of CHCHD2^{T61I} in Neuro2a cells. Neuro2a cells were transfected with the *CHCHD2^{WT}-HA* and *CHCHD2^{T61I}-HA* plasmids for 4 h, then cultured with medium containing 2% FBS and 10 μM retinoic acid for neuronal differentiation. At 28 h after transfection, cells were fixed and stained with anti-HA and anti-Tom20 antibodies. In (D), representative images are shown. Magnified images of the areas within the dashed squares are shown in the insets. Representative images at lower magnification are shown in Appendix Fig S1E. (E) Quantification of cells displaying mitochondrial CHCHD2 and extra-mitochondrial CHCHD2 puncta ($n \geq 100$ cells in each experiment).

F Neuro2a cells were treated as described in (D), and cell lysates were fractionated into cytosol, organelles (including mitochondria), nuclei, and cytoskeleton (including insoluble matter). The expression of each protein was analyzed by Western blotting using an anti-HA antibody. Cox4 was used as a control.

G A semiquantitative analysis of protein expression in (F) is shown. In (B, C, E, G), data are shown as the mean ± SD ($n = 3$). Comparisons were performed using unpaired two-tailed Student *t*-tests and one-way ANOVA followed by the Tukey *post hoc* tests. $^{**}P < 0.01$.

Source data are available online for this figure.

codes for α-Synuclein and is known as a causal gene of PD, and its phosphorylation (Ser^{129}) is also associated with PD (Ikeda *et al*, 2019). Consistent with Nefl, phospho-α-Synuclein signals are increased in cells expressing CHCHD2^{T61I}, but not in those expressing CHCHD2^{WT} (Fig 2G and H, Appendix Fig S3C). Furthermore, both phospho-Nefl and phospho-α-Synuclein showed substantial colocalization with CHCHD2^{T61I} (Fig 2E and G), but not mitochondria (Appendix Fig S3D and E), suggesting that CHCHD2^{T61I}, phospho-Nefl, and phospho-α-Synuclein gather together and form aggresomes. This was confirmed by the colocalization of an aggresome marker with phospho-Nefl and phospho-α-Synuclein (Appendix Fig S4A and B). A decrease in the level of total Nefl and increase in the level of - phospho-Nefl, total α-Synuclein, phospho-α-Synuclein, and toxic α-Synuclein oligomers was further confirmed by Western blot analysis (Fig 2I and J). Phospho-α-Synuclein signals were also observed in cells expressing extra-mitochondrial CHCHD2$^{\Delta 52}$, CHCHD2^{V66M}, and CHCHD2^{I80V} (Appendix Fig S4C).

How are Nefl and α-Synuclein phosphorylated? Because these phosphorylated proteins colocalized with CHCHD2^{T61I}, we suspected the possible involvement of unidentified kinases interacting with CHCHD2^{T61I}. To identify such kinases, we immunoprecipitated CHCHD2^{T61I}-HA and CHCHD2^{WT}-HA proteins using an anti-HA antibody from Neuro2a cells expressing these proteins and analyzed them by LC–MS/MS (Fig 3A). Csnk1e was identified as the only kinase that more preferentially interacts, with CHCHD2^{T61I} than with CHCHD2^{WT} (Fig 3A). Csnk1d is a close paralogue of Csnk1e, and they share substrates and perform the same biological roles in some cases (Bibian *et al*, 2013; Hanna-Addams *et al*, 2020). Csnk1d was also reported to be a candidate kinase for α-Synuclein (Okochi *et al*, 2000; Dzamko *et al*, 2014; Tenreiro *et al*, 2014). Therefore, we here analyzed Csnk1e and Csnk1d in an integrated manner. As expected, immunostaining of Csnk1e/d demonstrated their colocalization with extra-mitochondrial CHCHD2^{T61I}, but not CHCHD2^{WT} (Fig 3B). Csnk1e/d did not colocalize with mitochondria (Appendix Fig S5A). The immunofluorescent Csnk1e/d signals were validated by their disappearance using siRNA (Fig 3B–D, Appendix Fig S5B). The physical interaction of Csnk1e/d with CHCHD2^{T61I}, but not with CHCHD2^{WT}, was also shown by the close proximity assay (Fig 3E and F, Appendix Fig S5C).

We next addressed whether Csnk1e/d are responsible for the phosphorylation of Nefl and α-Synuclein. These two molecules contain consensus phosphorylation sequences for casein kinase (Fig 4A) (Okochi *et al*, 2000; Dzamko *et al*, 2014; Tenreiro *et al*, 2014; Rutherford *et al*, 2016), and the *in vitro* kinase assay demonstrated that α-Synuclein^{129} is phosphorylated by recombinant auto-active CSNK1E (R178C mutant), but not by non-active WT CSNK1E (Fig 4B and C) (Gietzen & Virshup, 1999; Guo *et al*, 2019). When we silenced Csnk1e in CHCHD2^{T61I}-expressing cells (Fig 3D), the amount of phospho-Nefl was significantly reduced (Fig 4D and E), and the reduction was more efficient upon the concomitant silencing of Csnk1e and Csnk1d (Fig 4D and E). Similar results were obtained when phospho-α-Synuclein expression (Fig 4F and G) and aggresome formation (Fig 4H and I) were analyzed, and when the Csnk1e/d inhibitor PF-670462 (Badura *et al*, 2007) was added to CHCHD2^{T61I}-expressing cells (Fig 4D–I). Note that Csnk1e/d silencing and PF-670462 treatment did not affect T61I mislocalization (Appendix Fig S6A–C). Taken together, CHCHD2^{T61I} mislocalization is the initial event, and subsequently CHCHD2^{T61I}

Figure 2. Generation of aggresomes and phosphorylation of both Nefl and α-Synuclein in cells expressing CHCHD2^{T61I}.

A–I Neuro2a cells were transfected with the *CHCHD2^{WT}-HA* and *CHCHD2^{T61I}-HA* plasmids for 4 h and then cultured in medium containing 2% FBS and 10 μM retinoic acid to induce neuronal differentiation. At 28 h after transfection, cells were fixed and stained with an anti-HA antibody and ProteoStat protein aggregation dye (A, B), anti-HA and anti-Nefl antibodies (C, D), anti-HA and anti-p-Nefl^{473} antibodies (E, F), and anti-HA and anti-p-α-Synuclein^{129} (p-α-Syn^{129}) antibodies (G, H). In (A, C, E, G), representative images are shown. Magnified images of the areas within the dashed squares are shown in the inset. Arrowheads indicate colocalized puncta between CHCHD2^{T61I}-HA and aggresomes or the indicated proteins. Representative images at lower magnification are shown in Appendix Figs S2A and S3A–C. (B, D, F, H) The amount of aggresomes (B), Nefl (D), p-Nefl^{473} (F), and p-α-Syn^{129} (H) was measured as the fluorescence intensity per cell ($n = 30$ cells in each experiment). Red bars indicate mean values. In (I), cell lysates were harvested at 28 h, and the expression of each protein was analyzed by Western blotting.

J A semiquantitative analysis of protein expression in (I) is shown. Data are shown as the mean ± SD ($n = 3$). In (B, D, F, H, J), comparisons were performed using unpaired two-tailed Student *t*-tests. $^{*}P < 0.05$. $^{**}P < 0.01$.

Source data are available online for this figure.

 © 2023 The Authors

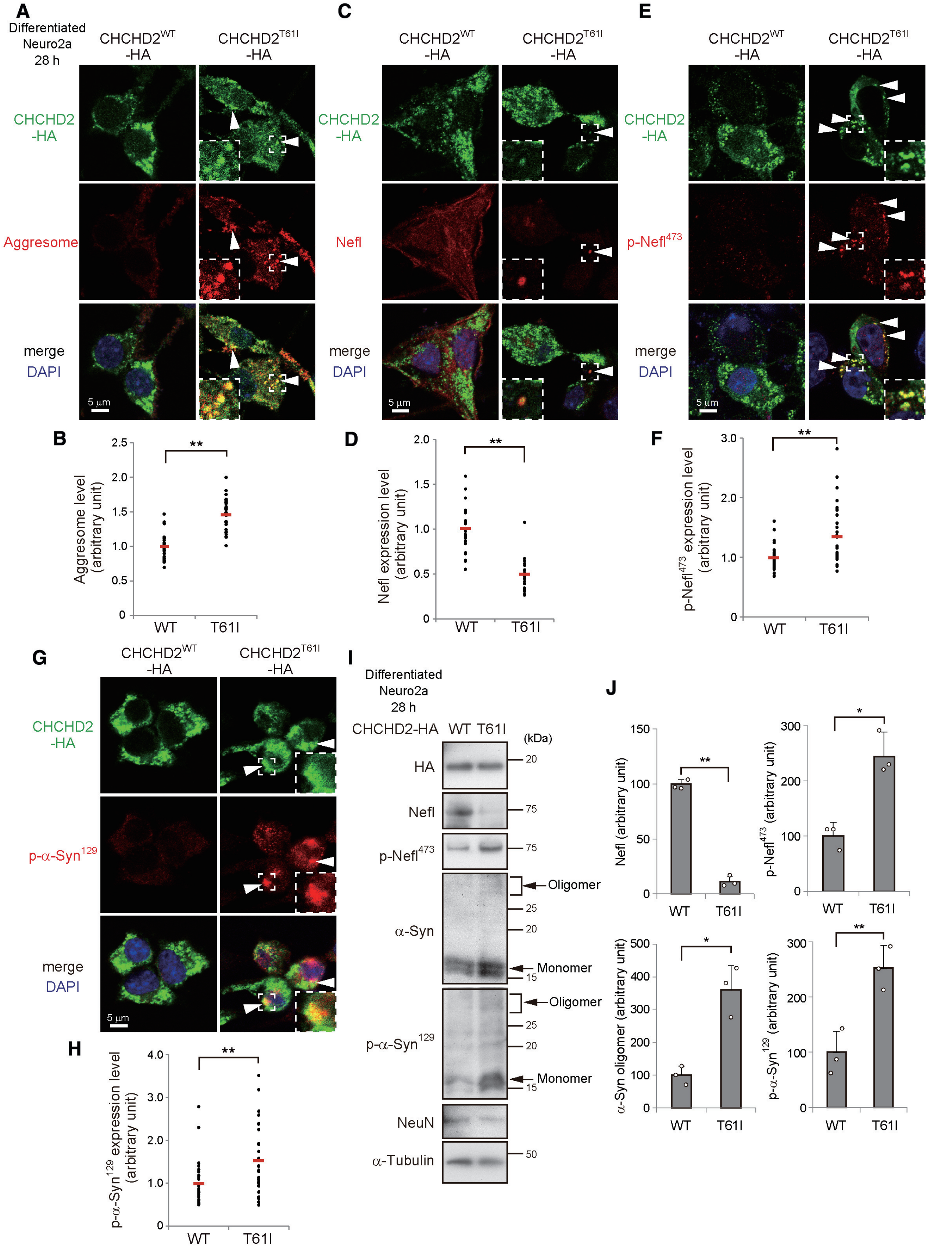
A
Differentiated Neuro2a 28 h
CHCHD2WT -HA
CHCHD2T61I -HA
CHCHD2 -HA
Aggresome
merge
DAPI
5 μm
B
Aggresome level (arbitrary unit)
WT
T61I
C
Nefl
D
Nefl expression level (arbitrary unit)
E
p-Nefl473
F
p-Nefl473 expression level (arbitrary unit)
G
p-α-Syn129
H
p-α-Syn129 expression level (arbitrary unit)
I
CHCHD2-HA WT T61I
(kDa)
HA
Nefl
α-Syn
Oligomer
Monomer
NeuN
α-Tubulin
J
Nefl (arbitrary unit)
p-Nefl473 (arbitrary unit)
α-Syn oligomer (arbitrary unit)
p-α-Syn129 (arbitrary unit)

Figure 2.

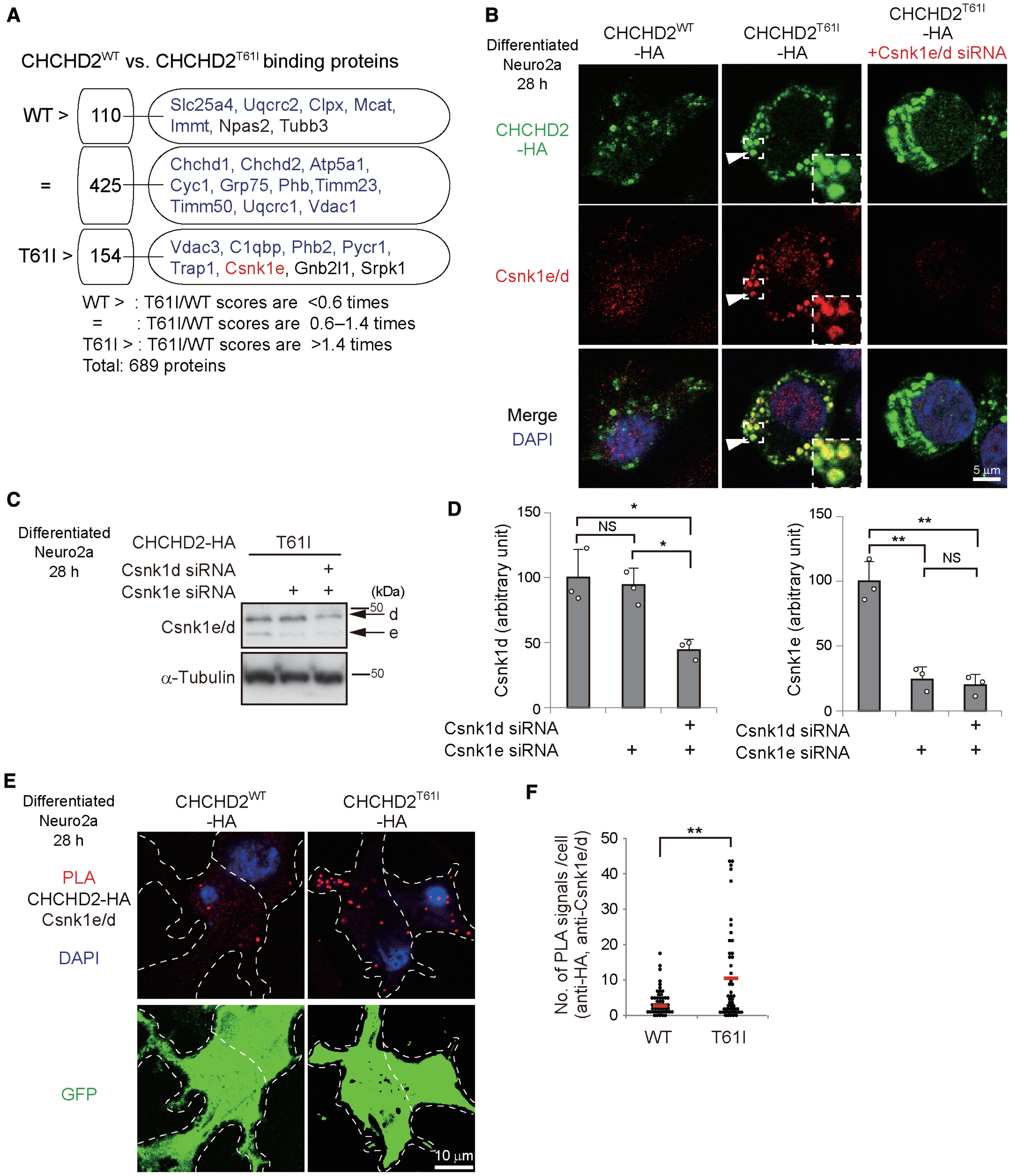

Figure 3.

© 2023 The Authors

Figure 3. Interaction between CHCHD2^{T61I} and Csnk1e/d.

A Comparison of binding proteins of CHCHD2^{WT} with those of CHCHD2^{T61I} by shotgun mass spectrometry analysis. Immunoprecipitants using the anti-HA antibody were obtained from Neuro2a cells expressing CHCHD2^{WT}-HA or CHCHD2^{T61I}-HA and were analyzed by mass spectrometry. The identified proteins (689 proteins) were classified into three groups according to their binding efficiency with CHCHD2^{WT} and CHCHD2^{T61I}. The number indicates the number of identified proteins, and representative proteins are shown. Mitochondrial proteins are colored blue, and Csnk1e is colored red.

B Accumulation of Csnk1e/d on CHCHD2^{T61I} puncta. Similar experiments to Fig 2 were performed with or without Csnk1e/d silencing. Cells were fixed and stained with an anti-HA and anti-Csnk1e/d antibodies. Representative images are shown. Magnified images of the areas within the dashed squares are shown in the inset. Arrowheads indicate colocalization between CHCHD2^{T61I}-HA and Csnk1e/d. Representative images at lower magnification are shown in Appendix Fig S5B.

C Efficiency of Csnk1e/d silencing. Neuro2a cells were transfected with the *CHCHD2^{T61I}-HA* plasmid together with Csnk1e and Csnk1d siRNAs, and were differentiated after 4 h. At 28 h after transfection, cell lysates were harvested, and the expression of each protein was analyzed by Western blotting using an anti-Csnk1e/d antibody. "d" and "e" indicate Csnk1d and Csnk1e, respectively. Tubulin was used as a control.

D A semiquantitative analysis of protein expression in (C) is shown. Data are shown as the mean ± SD (n = 3).

E, F Interaction between CHCHD2^{T61I} and Csnk1e/d. Neuro2a cells were transfected with the *CHCHD2^{WT}-HA* and *CHCHD2^{T61I}-HA* plasmids together with *pmax-GFP* (to detect transfected cells), and were differentiated into neuronal cells at 4 h after transfection. At 28 h after transfection, cells were fixed and the PLA was performed using anti-HA and anti-Csnk1e/d antibodies and Duolink PLA reagents. In (E), PLA signals were clearly observed in cells expressing CHCHD2^{T61I}-HA, but were faint and localized in the cytosol in cells expressing CHCHD2^{WT}-HA. Representative images at lower magnification are shown in Appendix Fig S5C. In (F), the number of PLA signals was counted (n = 50 cells in each experiment). Red bars indicate mean values. In (D, F), comparisons were performed using the unpaired two-tailed Student *t*-test and one-way ANOVA followed by the Tukey *post hoc* test. *P < 0.05. **P < 0.01. NS: not significant.

Source data are available online for this figure.

recruits Csnk1e/d, resulting in the phosphorylation of Nefl and α-Synuclein, and the formation of aggresomes.

Note that CHCHD2^{T61I}-expressing Neuro2a cells were more vulnerable than CHCHD2^{WT}-expressing Neuro2a cells to long culture times after differentiation, as assessed by the propidium iodide (PI) assay (Appendix Fig S7A and B) and by abnormal cell shape with shortened neurites (Appendix Fig S7C). Cell death was suppressed not only by the apoptosis inhibitor Q-VD-Oph, but also by PF-670462, indicating that Csnk1e/d-induces the phosphorylation of Nefl, and α-Synuclein causes toxicity. Similar results were obtained when cells were incubated with the proteasome inhibitor MG132 (Appendix Fig S7D).

Chchd2^{T61I} knock-in mice demonstrate low motor performance and dopaminergic neuronal loss

To analyze the pathology of autosomal dominant PD caused by the CHCHD2^{T61I} mutation, we generated Chchd2^{T61I} knock-in mice as a mouse model that mimics human disease. Rodents have the *Zbed5* (*Scand3*) gene, which has almost the same N-terminal amino acid sequence as the Chchd2 sequence (97.3% identity in amino acids 1–150), and is located next to the *Chchd2* gene (Fig EV2A and B). To avoid affecting *Zbed5*, we designed a target vector to insert the *C182T* mutation in the *Chchd2* gene, but not in the *Zbed5* gene (Fig EV2C), and generated Chchd2^{T61I} knock-in mice using the CRISPR/Cas9 system (Figs 5A and EV2D and E). We obtained two mouse lines and their phenotypes were almost the same. Both heterozygous and homozygous knock-in mice were born at a normal Mendelian ratio and were fully viable and fertile. However, these mice demonstrated abnormal motor performance from about 30 weeks of age, including abnormal limb-clasping reflexes (Fig 5A, Appendix Fig S8A), abnormal footprint patterns (Fig 5B, Appendix Fig S8B), and lower rotarod performance than littermate WT mice (Fig 5C). Their disease severity was gene dosage-dependent. Consistently, tyrosine hydroxylase (TH) staining was decreased in heterozygous knock-in mice, as observed in human CHCHD2^{T61I} PD patients, and more so in homozygous knock-in mice (Fig 5D and E). The TH-positive neurons in Chchd2^{T61I} knock-in mice showed abnormal cell shapes with weak TH staining and condensed Chchd2^{T61I} puncta (Fig 5F, Appendix Fig S9A). These puncta were localized outside of the mitochondria (Fig 5G, Appendix Fig S9B and C). Loss of Chchd2^{T61I} from mitochondria was confirmed in mitochondria purified from Chchd2^{T61I} knock-in mouse brains (Appendix Fig S9D and E). Furthermore, as with CHCHD2^{T61I}-expressing Neuro2a cells, Chchd2^{T61I} puncta colocalized with phospho-Nefl^{473} (Fig 5H, Appendix Fig S9F and G), phospho-α-Synuclein (Fig 5I, Appendix Fig S9H and I), and Csnk1e/d (Fig 5J, Appendix Fig S9J), and

Figure 4. Involvement of Csnk1e/d in Nefl and α-Synuclein phosphorylation and aggresome formation in cells expressing CHCHD2^{T61I}.

A Alignment of amino acid sequences containing the phosphorylation sites of *Mus musculus* (mouse) Snca (α-Synuclein, α-Syn) and Nefl, and Homo sapiens (human) SNCA (α-Synuclein, α-SYN) and NEFL. The phosphorylation sites (Ser) are indicated in red. The amino acids three residues before the phosphorylation sites are glutamic acids (boxed in red), forming consensus phosphorylation sequences for casein kinase.

B *In vitro* kinase assay using recombinant human α-Synuclein and Csnk1e. GST-tagged α-SYN and inactive CSNK1E^{WT} or an active mutant (CSNK1E^{R178C}) were incubated in kinase assay buffer for 30 min at 30°C. Phosphorylation levels were analyzed by Western blotting.

C A semiquantitative analysis of protein expression in (B) is shown. Data are shown as the mean ± SD (n = 3).

D–I Neuro2a cells were transfected with the *CHCHD2^{T61I}-HA* plasmid together with the indicated siRNAs and were differentiated into neuronal cells after 4 h. At 28 h after transfection, cells were fixed and stained with anti-HA, anti-p-Nefl^{473}, and anti-p-α-Syn^{129} antibodies, or ProteoStat aggresome detection dye. Similar experiments were performed by the addition of PF-670462 (10 μM) after 4 h of transfection instead of gene silencing. In (D, F, H), representative images are shown. Magnified images of the areas within the dashed squares are shown in the insets. Arrowheads indicate colocalized puncta between CHCHD2^{T61I}-HA and the indicated phosphorylated proteins or aggresomes. In (E, G, I), the amount of p-Nefl^{473} (E), p-α-Syn^{129} (G), and aggresomes (I) was measured as the fluorescence intensity per cell (n = 30 cells in each experiment). Red bars indicate mean values. In (C, E, G, I), comparisons were performed using one-way ANOVA followed by the Tukey *post hoc* tests or unpaired two-tailed Student *t*-test. *P < 0.05; **P < 0.01. NS: not significant.

Source data are available online for this figure.

© 2023 The Authors

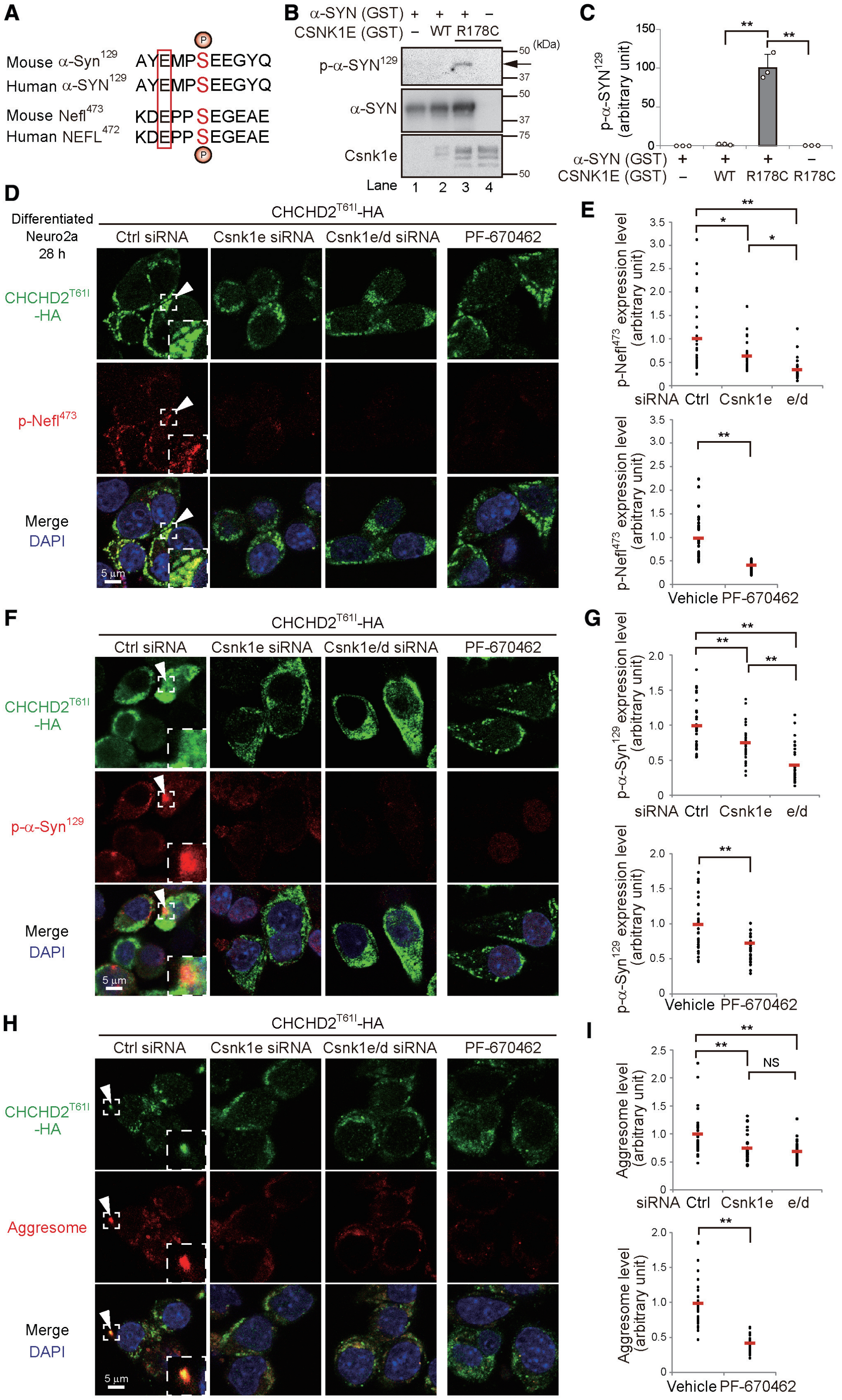
A
Mouse α-Syn129 AYEMPSEEGYQ
Human α-SYN129 AYEMPSEEGYQ
Mouse Nefl473 KDEPPSEGEAE
Human NEFL472 KDEPPSEGEAE
B
α-SYN (GST) + + + –
CSNK1E (GST) – WT R178C
p-α-SYN129
α-SYN
Csnk1e
Lane 1 2 3 4
(kDa)
C
p-α-SYN129 (arbitrary unit)
α-SYN (GST) + + + –
CSNK1E (GST) – WT R178C R178C
D
Differentiated Neuro2a 28 h
CHCHD2T61I-HA
Ctrl siRNA Csnk1e siRNA Csnk1e/d siRNA PF-670462
p-Nefl473
Merge DAPI
5 μm
E
p-Nefl473 expression level (arbitrary unit)
siRNA Ctrl Csnk1e e/d
Vehicle PF-670462
F
p-α-Syn129
G
p-α-Syn129 expression level (arbitrary unit)
H
Aggresome
I
Aggresome level (arbitrary unit)
NS

Figure 4.

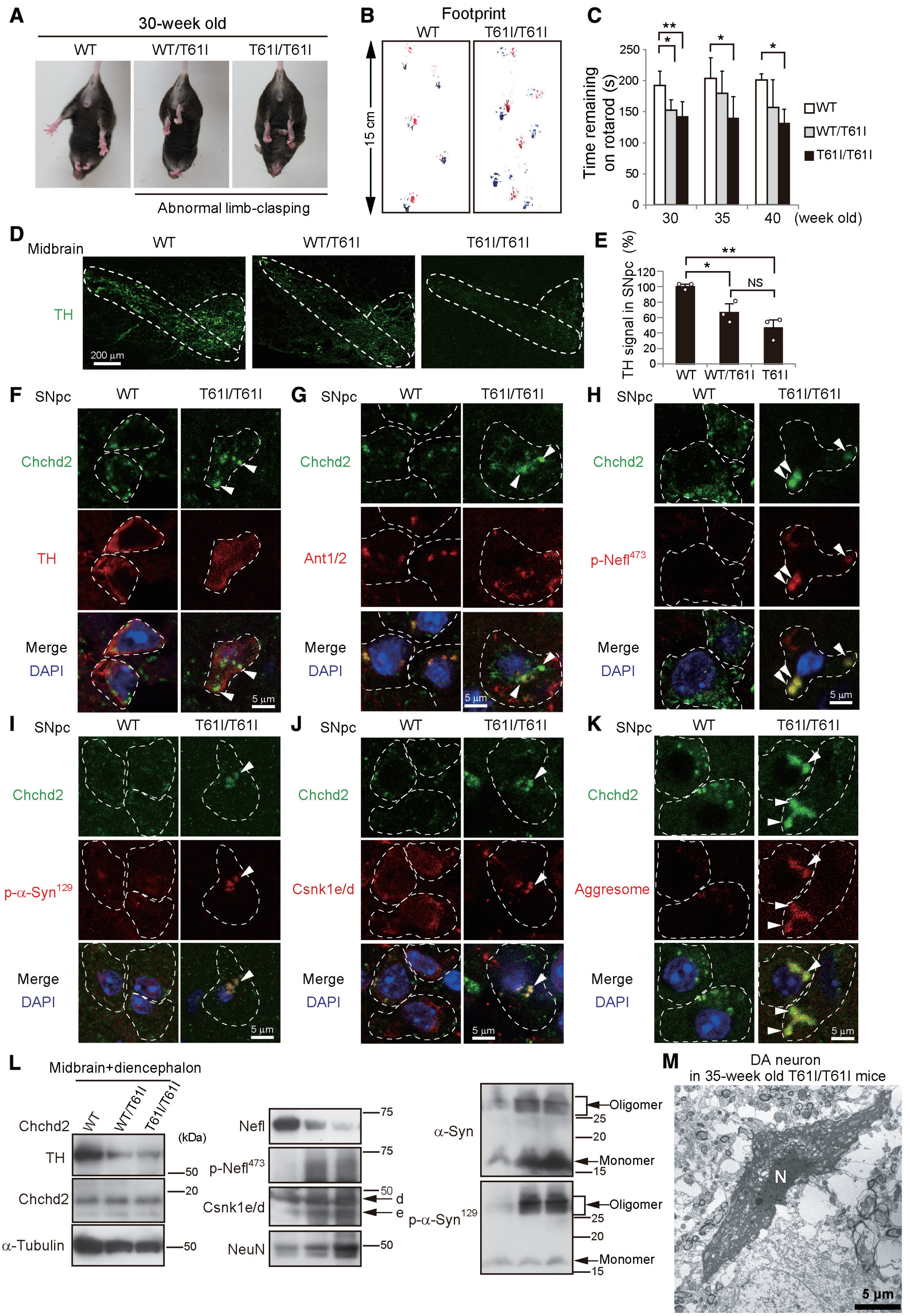
A
30-week old
WT
WT/T61I
T61I/T61I
Abnormal limb-clasping
B
Footprint
WT
T61I/T61I
15 cm
C
Time remaining on rotarod (s)
WT
WT/T61I
T61I/T61I
30
35
40
(week old)
D
Midbrain
TH
200 µm
E
TH signal in SNpc (%)
NS
F SNpc
G SNpc
H SNpc
Chchd2
TH
Ant1/2
p-Nefl473
Merge
DAPI
5 µm
I SNpc
J SNpc
K SNpc
p-α-Syn129
Csnk1e/d
Aggresome
L
Midbrain+diencephalon
(kDa)
α-Tubulin
Nefl
NeuN
α-Syn
p-α-Syn129
Oligomer
Monomer
M
DA neuron
in 35-week old T61I/T61I mice
N
5 µm

Figure 5.

Figure 5. Reduced motor performance and dopaminergic neuronal loss in Chchd2^{T61I} knock-in mice.

A–C Abnormal motor performance in Chchd2^{T61I} hetero (WT/T61I) and homo (T61I/T61I) knock-in mice. In (A), the limb-clasping reflex was observed at 30 weeks of age. Quantitative analyses are shown in Appendix Fig S8A. In (B), the footprint assay indicated a motor deficit at 40 weeks of age. Quantitative analyses are shown in Appendix Fig S8B. In (C), the time that the indicated mice remained on the rotarod was measured. Data are shown as the mean ± SD (n = 4–8 mice).

D–F Reduction in TH signals in the SNpc of knock-in mice. In (D), brain cryosections were immunostained with the dopaminergic cell marker TH. Representative images of the SNpc and ventral tegmental area (VTA) are shown. Dashed lines indicate the SNpc and VTA regions. Bars = 200 μm. In (E), TH signals in the SNpc (average fluorescence intensity per region) are shown as the mean ± SD (n = 3). In (F), cryosections of the SNpc were immunostained with anti-Chchd2 and anti-TH antibodies. Arrowheads indicate Chchd2^{T61I} puncta in TH-positive cells. Dashed lines indicate cell shapes.

G–K Extra-mitochondrial aggresome formation by p-Nefl^{473}, p-α-Syn^{129}, and Csnk1e/d in the SNpc of knock-in mice. Cryosections of the SNpc were immunostained with anti-Chchd2 and anti-Ant1/2 (G), anti-p-Nefl^{473} (H), anti-p-α-Syn^{129} (I), and anti-Csnk1e/d antibodies (J), and with ProteoStat dye (K). Ant1/2 are mitochondrial membrane proteins and arrowheads indicate extra-mitochondrial Chchd2^{T61I} (G). In (H–K), arrowheads indicate the colocalization of puncta with Chchd2^{T61I} and the indicated proteins or aggresomes. Dashed lines indicate cell shapes. Quantitative analyses are shown in Appendix Fig S9G, I and L. In addition, the results of heterozygous mice are shown in Appendix Fig S9.

L Isolated midbrain and diencephalon lysates were subjected to Western blotting. A semiquantitative analysis of protein expression is shown in Appendix Fig S10.

M EM analysis of dopaminergic neurons in the SNpc of CHCHD2^{T61I} knock-in mice. A dying dopaminergic neuron with small inclusion bodies is shown. N: nucleus. A magnified image of the same cell is shown in Fig EV3C. In (C, E), comparisons were performed using one-way ANOVA followed by the Tukey *post hoc* tests. $*P < 0.05$; $**P < 0.01$. NS: not significant.

Source data are available online for this figure.

formed aggresomes (Fig 5K, Appendix Fig S9K and L). Western blot analysis confirmed the low expression of TH and Nefl, and high expression of phospho-Nefl, monomeric α-Synuclein, toxic α-Synuclein oligomers, phospho-α-Synuclein, and Csnk1e/d (Fig 5L, Appendix Fig S10), consistent with Chchd2^{T61I}-expressing Neuro2a cells (Figs 1–4). Electron microscopy (EM) analysis confirmed the existence of electron dense inclusion bodies in some dopaminergic neurons (Figs 5M and EV3A–D), and some of them were shrinking and dying (Figs 5M and EV3C). These results suggested that the same pathogenesis as CHCHD2^{T61I}-expressing Neuro2a cells occurred in the SNpc midbrain of Chchd2^{T61I} knock-in mice, in which extra-mitochondrial Chchd2^{T61I} recruits Csnk1e/d and thereby phosphorylates Nefl and α-Synuclein.

We observed equivalent Chchd2^{T61I} expression levels in the cerebral cortex and cerebellum as in the midbrain, as for endogenous Chchd2 in WT mice (Appendix Fig S11A and B). However, less Chchd2^{T61I} mislocalization from mitochondria was observed in the cerebral cortex than in the midbrain (Appendix Fig S11C). Therefore, although we observed the formation of toxic α-Synuclein oligomers and phospho-α-Synuclein, and the expression of Csnk1e/d even in the cerebral cortex and cerebellum, the extent was much less than in the SNpc (Appendix Fig S11B). Furthermore, neurons of cerebral cortex and cerebellum did not contain aggresomes (Appendix Fig S11D), and were not injured as assessed by immunostaining with the neuronal marker NeuN (Appendix Fig S11E). Again, the reason for the different phenotypes as a result of extra-mitochondrial Chchd2^{T61I} between dopaminergic neurons and other neurons remains unclear. Taken together, dopaminergic neurons are sensitive to extra-mitochondrial Chchd2^{T61I}, possibly via the Csnk1e/d-induced phosphorylation of Nefl and α-Synuclein.

Chchd2^{T61I} transgenic mice also demonstrate low motor performance and dopaminergic neuronal loss

We also created Chchd2^{T61I}-HA Tg mice expressing an HA-tagged gene at the C-terminus of Chchd2^{T61I} under the prion promoter, by which mouse Chchd2^{T61I} was expected to be specifically expressed in the brain (Fig EV4A, Appendix Fig S12A). The generation of Chchd2^{T61I}-HA Tg mice was confirmed by PCR and Western blotting, demonstrating that Chchd2^{T61I}-HA was expressed in the midbrain/diencephalon, but not in the liver (Fig EV4B, Appendix Fig S12B and C). Chchd2^{T61I}-HA Tg mice were born at the normal Mendelian ratio. Although the expression level of exogenous Chchd2^{T61I} was lower than that of endogenous Chchd2 in the midbrain/diencephalon (Fig EV4B), these mice demonstrated the same abnormal phenotype with Chchd2^{T61I} knock-in mice, i.e., motor defects from about the age of 30 weeks, including abnormal limb-clasping reflexes (Fig EV4A, Appendix Fig S13A), abnormal footprint patterns (Fig EV4C, Appendix Fig S13B), and abnormalities on the rotarod test (Fig EV4D). Both Western blotting and immunostaining showed low expression of TH (Fig EV4B and E–G, Appendix Fig S13C), demonstrating severely damaged dopaminergic neurons in Tg mice, as observed in Chchd2^{T61I} knock-in mice. Furthermore, as with Chchd2^{T61I} knock-in mice, Chchd2^{T61I}-HA Tg mice showed a decrease in the level of Nefl and an increase in levels of phospho-

Figure 6. Effect of PF-670462 in Chchd2^{T61I} knock-in mice.

A–C Improvement of motor performance in Chchd2^{T61I} knock-in mice by PF-670462. PF-670462 (50 ng/g BW/day) or 5% DMSO was continuously infused into the brain of Chchd2^{T61I} knock-in mice from 16 weeks of age. Improvement of the limb-clasping reflex (A) and abnormal footprint patterns (B) was observed. Quantitative analyses are shown in Appendix Fig S17C and D. In (C), the time the indicated mice remained on the rotarod was measured. Data are shown as the mean ± SD (vehicle, n = 3; PF-670462, n = 4).

D, E The same experiments as Fig 5D and E were performed using PF-670462-infused and vehicle-infused *Chchd2^{T61I}* knock-in mice.

F–J The same experiments as Fig 5G–K were performed using PF-670462-infused and vehicle-infused *Chchd2^{T61I}* knock-in mice. Quantitative analyses are shown in Appendix Fig S17F–I. In (C, E), comparisons were performed using one-way ANOVA followed by the Tukey *post hoc* test and an unpaired two-tailed Student t-test. $**P < 0.01$.

Source data are available online for this figure.

© 2023 The Authors

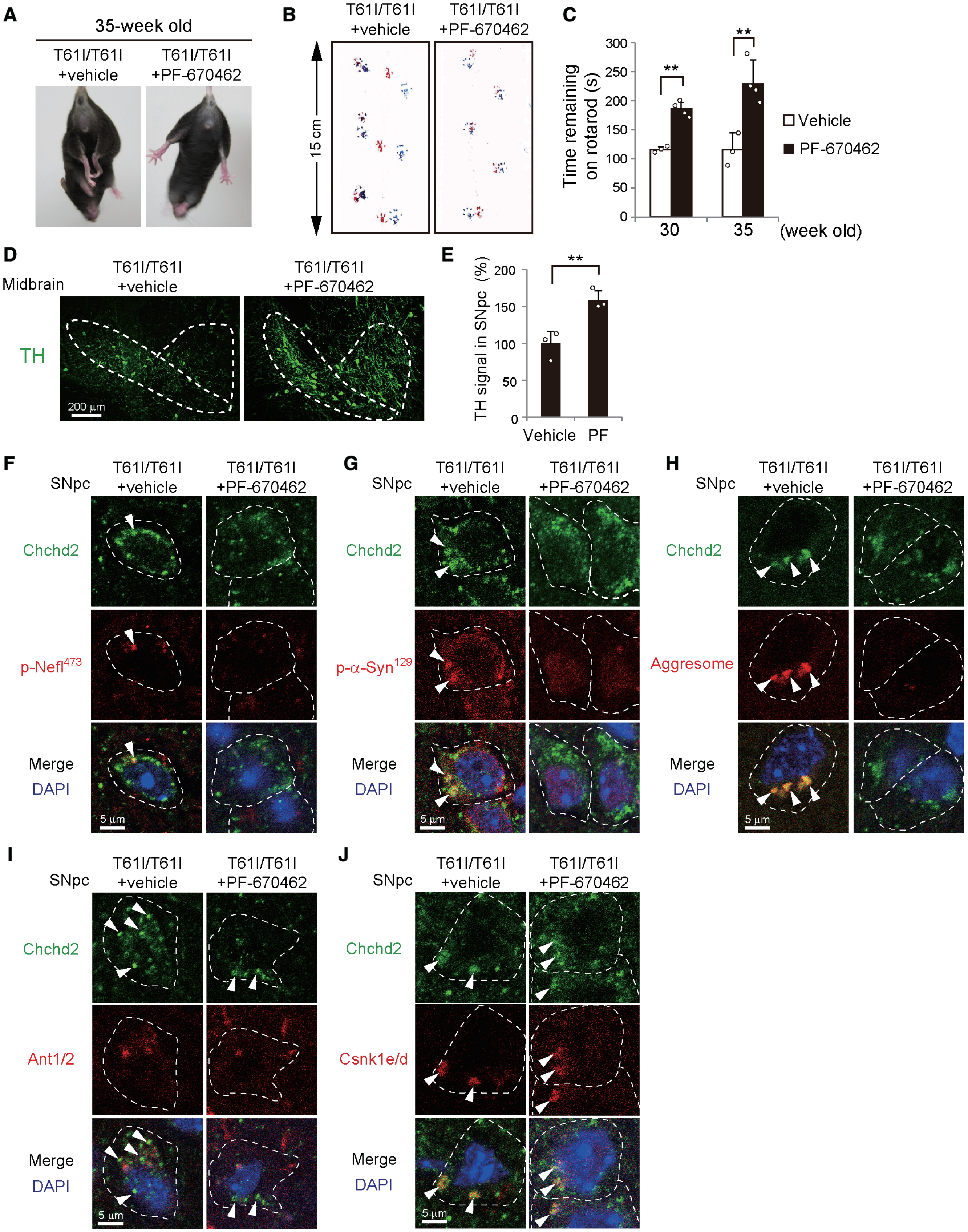
A
35-week old
T61I/T61I +vehicle
T61I/T61I +PF-670462
B
T61I/T61I +vehicle
T61I/T61I +PF-670462
15 cm
C
Time remaining on rotarod (s)
**
**
Vehicle
PF-670462
30
35
(week old)
D
Midbrain
T61I/T61I +vehicle
T61I/T61I +PF-670462
TH
200 µm
E
TH signal in SNpc (%)
**
Vehicle
PF
F
SNpc
T61I/T61I +vehicle
T61I/T61I +PF-670462
Chchd2
p-Nefl473
Merge
DAPI
5 µm
G
SNpc
T61I/T61I +vehicle
T61I/T61I +PF-670462
Chchd2
p-α-Syn129
Merge
DAPI
5 µm
H
SNpc
T61I/T61I +vehicle
T61I/T61I +PF-670462
Chchd2
Aggresome
Merge
DAPI
5 µm
I
SNpc
T61I/T61I +vehicle
T61I/T61I +PF-670462
Chchd2
Ant1/2
Merge
DAPI
5 µm
J
SNpc
T61I/T61I +vehicle
T61I/T61I +PF-670462
Chchd2
Csnk1e/d
Merge
DAPI
5 µm

Figure 6.

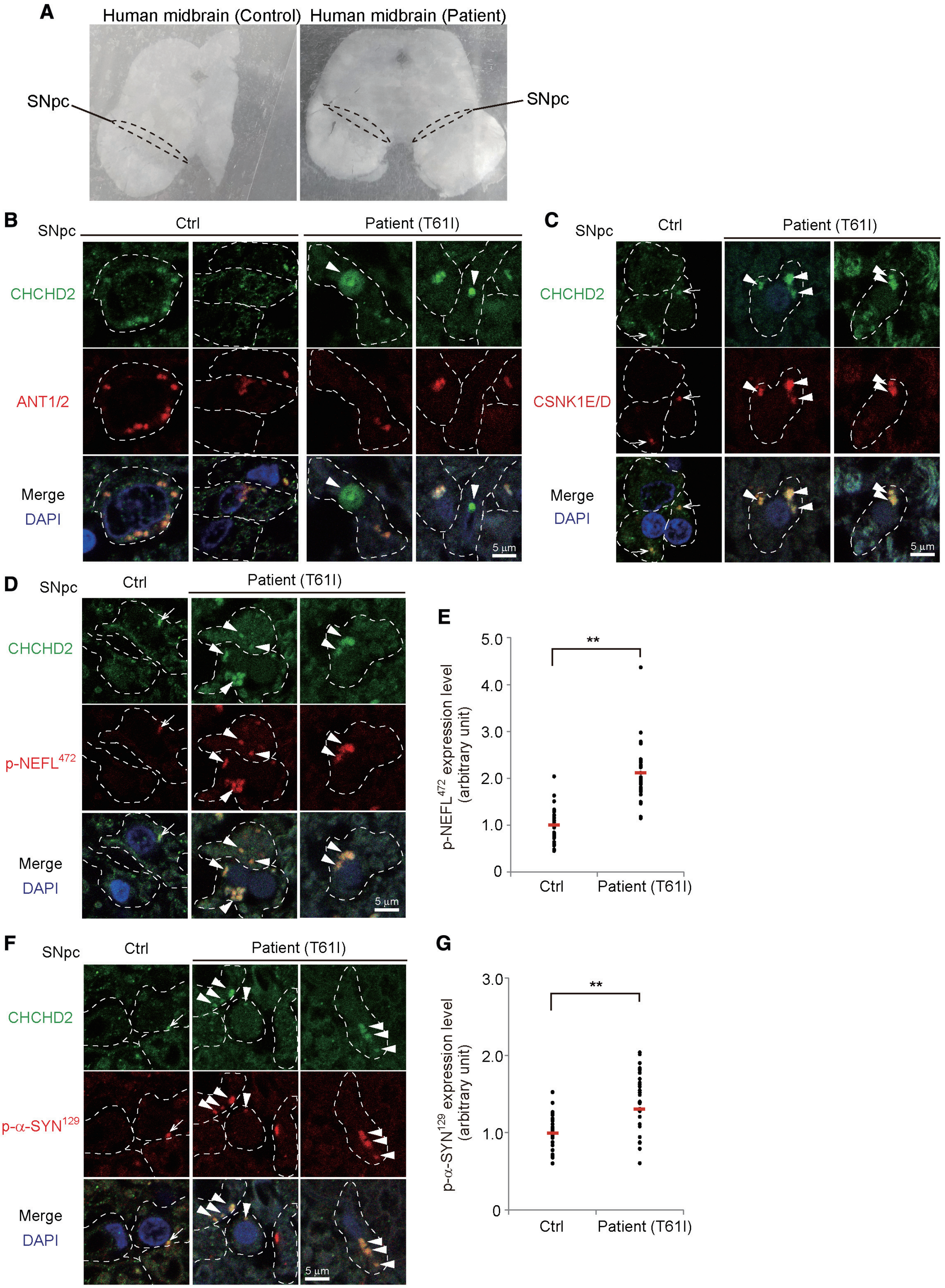

Figure 7.

 © 2023 The Authors

Figure 7. Analysis of an autopsied brain of a PD patient harboring the CHCHD2^{T61I} mutation.

A Photos of the midbrain of a control subject (left) and PD patient harboring the CHCHD2^{T61I} mutation (right). The dashed lines indicate the SNpc. A portion of the midbrain of the control subject was lost when slicing.

B–G Colocalization of extra-mitochondrial CHCHD2^{T61I} with CSNK1E/D, p-NEFL^{472}, and p-α-SYN^{129} in the brain of a PD patient harboring the CHCHD2^{T61I} mutation. Each brain section was deparaffinized and immunostained with anti-CHCHD2 and anti-ANT1/2 (B), anti-CSNK1E/D (C), anti-p-NEFL^{472} (D, E), or anti-p-α-SYN^{129} antibodies (F, G). Arrowheads indicate extra-mitochondrial CHCHD2^{T61I} puncta (B), and puncta showing colocalization of CHCHD2^{T61I} with CSNK1E/D (C), p-NEFL^{472} (D), and p-α-SYN^{129} (F). Arrows indicate weaker colocalization between CHCHD2^{T61I} and the indicated proteins in the control brain (C, D, F). Dashed lines indicate cell shapes. In (E, G), the amount of p-NEFL^{472} (E) and p-α-SYN^{129} (G) was measured by the average fluorescence intensity per cell (n = 30 cells in each experiment). Red bars indicate mean values. Comparisons were performed using the unpaired two-tailed Student t-tests. $^{**}P < 0.01$.

Source data are available online for this figure.

Nefl, monomeric α-Synuclein, toxic α-Synuclein oligomers, phospho-α-Synuclein, and Csnk1e/d (Fig EV4B, Appendix Fig S13C). Immunostaining analysis confirmed the colocalization of extra-mitochondrial Chchd2^{T61I} puncta (Fig EV4H) with phospho-Nefl (Fig EV4I), phospho-α-Synuclein (Fig EV4J), aggresome puncta (Fig EV4K), and Csnk1e/d (Fig EV4L) in the SNpc neurons of Tg mice (Appendix Fig S13D–F). Dopaminergic neurons can be identified by EM, and we found electron dense inclusion bodies in these neurons, and some of the neurons were dying with shrinking (Fig EV4M, Appendix Fig S14A–C).

Chchd2^{T61I}-HA was also expressed in granular cells of the cerebellum, but not in the cortex (Appendix Fig S15A–C). However, unlike dopaminergic neurons, granular cells were not damaged (Appendix Fig S15A). Furthermore, the upregulation of phospho-α-Synuclein and Csnk1e/d was not observed (Appendix Fig S15C), despite the existence of several extra-mitochondrial Chchd2^{T61I}-HA puncta (Appendix Fig S15B). The reason underlying the different phenotypes observed between dopaminergic neurons and cerebellar neurons remains unclear at present.

Importantly, unlike Chchd2^{T61I} knock-in mice and Chchd2^{T61I} Tg mice, Chchd2-deficient mice develop symptoms only after 110 weeks of age (Sato *et al*, 2021). These Chchd2-deficient mice do not express phospho-Nefl and phospho-α-Synuclein and do not form aggresomes at the age of 30 weeks (Appendix Fig S16A–D). TH staining was also not altered in these mice at the age of 30 weeks (Appendix Fig S16E and F). Therefore, the pathogenesis of CHCHD2^{T61I} patients appears not to be the loss of function of CHCHD2, but the gain of function of mutant CHCHD2^{T61I}, which is consistent with the autosomal dominant inheritance of this disease.

Protective effects of PF-670462 in Chchd2^{T61I} knock-in mice

Because the Csnk1e/d inhibitor PF-670462 inhibited the phosphorylation of Nefl and α-Synuclein, and aggresomes were observed in CHCHD2^{T61I}-expressing Neuro2a cells (Fig 4D–I), we administered PF-670462 into the brain of Chchd2^{T61I} homozygous knock-in mice using an osmotic pump from 16 weeks of age (Appendix Fig S17A and B). At 30 weeks of age, abnormal neurological phenotypes, including the limb-clasping reflex (Fig 6A, Appendix Fig S17C), and abnormalities in the footprint test (Fig 6B, Appendix Fig S17D) and rotarod analyses (Fig 6C) were substantially improved by PF-670462. There was no effect of PF-670462 on WT mice (Appendix Fig S17E). In addition, PF-670462 improved the loss of dopaminergic neurons in the SNpc (Fig 6D and E), suppressed the puncta formation of phospho-Nefl (Fig 6F, Appendix Fig S17F) and phospho-α-Synuclein (Fig 6G, Appendix Fig S17G), and the formation of aggresomes (Fig 6H, Appendix Fig S17H), although it did not suppress the formation of extra-mitochondrial Chchd2^{T61I} puncta (Fig 6I, Appendix Fig S17I) and its partial colocalization with Csnk1e/d (Fig 6J). Taken together, our results demonstrate that the pathology of Chchd2^{T61I}-induced neurodegeneration is caused by the mislocalization of Chchd2^{T61I}, followed by the recruitment of Csnk1e/d, resulting in the accumulation of phospho-Nefl and phospho-α-Synuclein, and the formation of aggresomes.

Involvement of Csnk1e/d in human patients with the CHCHD2^{T61I} mutation

To investigate whether the pathology observed in CHCHD2^{T61I} PD model mice is also observed in human patients, we analyzed an autopsied brain of a PD patient with the CHCHD2^{T61I} mutation (Fig 7A). Immunofluorescence analysis demonstrated that CHCHD2^{T61I} proteins were mislocalized from mitochondria (Fig 7B), and colocalized with CSNK1E/D (Fig 7C). Furthermore, phospho-NEFL^{472} (corresponding to phospho-Nefl^{473} in mice) (Fig 7D and E) and phospho-α-Synuclein^{129} (Fig 7F and G) were generated and also colocalized with CHCHD2^{T61I} puncta, suggesting that the same pathogenesis occurs in human PD patients harboring the CHCHD2^{T61I} mutation.

Figure 8. Analysis of iPSC-derived dopaminergic neurons from a patient with the CHCHD2^{T61I} mutation.

A–E iPSCs were prepared from a healthy control and a PD patient harboring the CHCHD2^{T61I} mutation. An isogenic control iPSC line was generated by the correction of the gene mutation (see Fig EV5A and B). Then, these iPSC cells were differentiated into dopaminergic (DA) neurons, and analyzed by immunofluorescence using anti-CHCHD2 (A–E), anti-ANT1/2 (A), anti-CSNK1E/D (B), anti-p-NEFL^{472} (C), and anti-p-α-SYN^{129} antibodies (D), and ProteoStat aggresome dye (E).

F–H CHCHD2^{T61I} iPSC-derived DA neurons were treated with PF-670462 (10 μM) for 20 h and analyzed by immunofluorescence using anti-CHCHD2 (F–H), anti-p-NEFL^{472} (F), and anti-p-α-SYN^{129} antibodies (G), and ProteoStat aggresome dye (H). Arrowheads indicate extra-mitochondrial CHCHD2^{T61I} (A), puncta showing colocalization of CHCHD2^{T61I} with CSNK1E/D (B), p-NEFL^{472} (C, F), and p-α-SYN^{129} (D, G), or aggresomes (E, H). Dashed lines indicate cell shapes. Quantitative analyses are shown in Fig EV5.

Source data are available online for this figure.

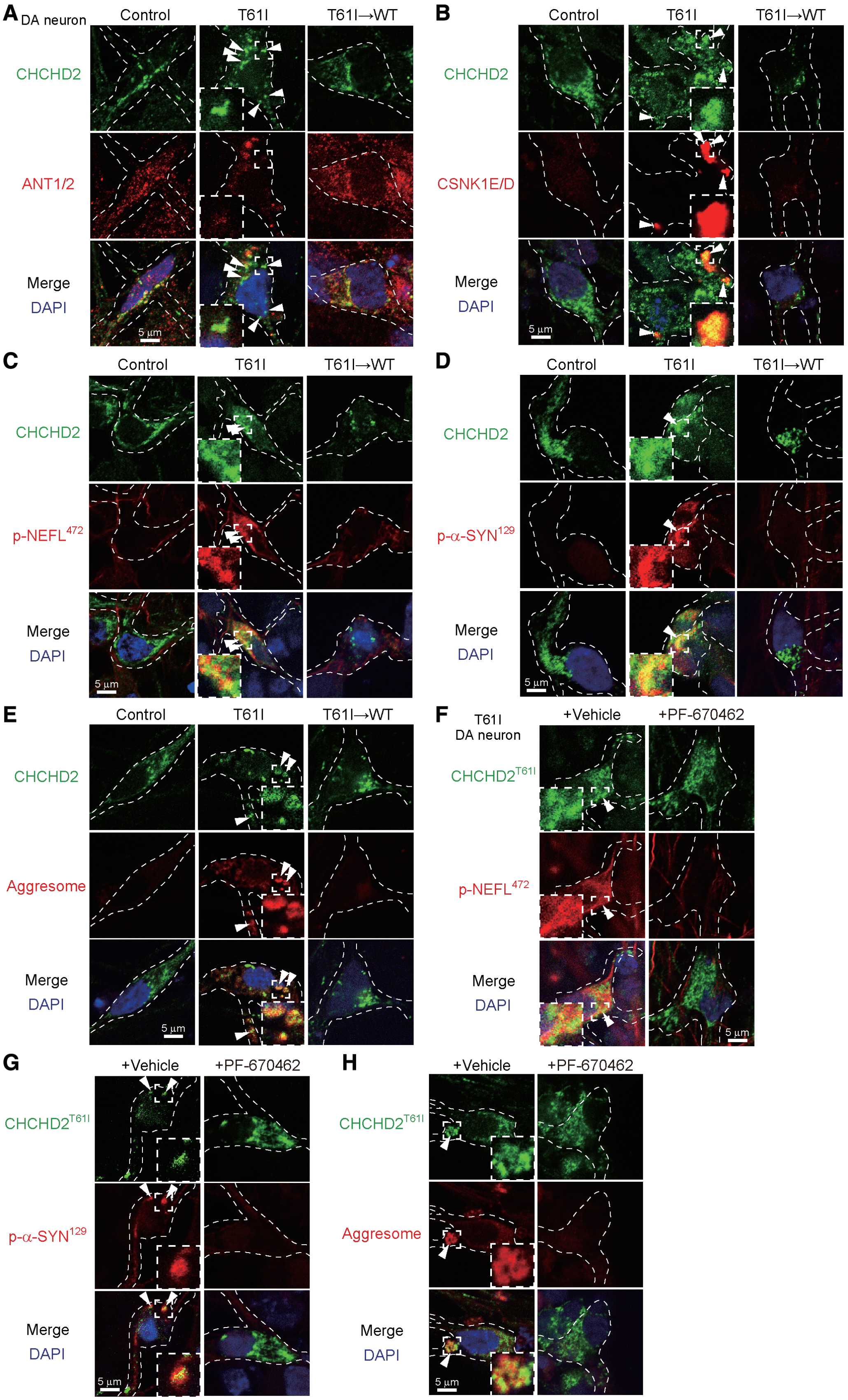

Figure 8.

 © 2023 The Authors

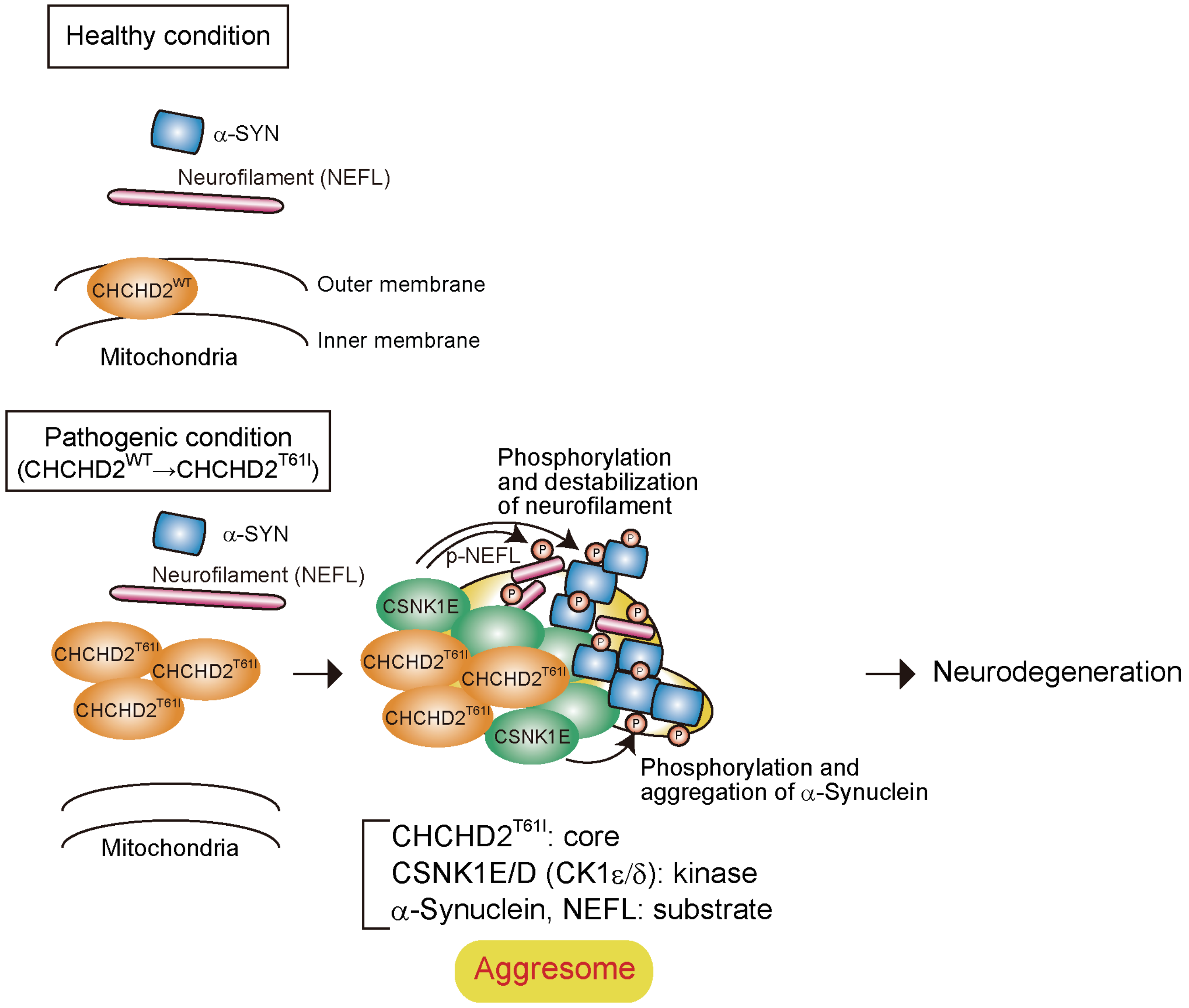

Figure 9. Schematic model of the pathogenic mechanism of CHCHD2^{T61I}-induced PD.

CHCHD2^{WT} localizes in mitochondria, whereas CHCHD2^{T61I} mislocalizes to the cytosol. Extra-mitochondrial CHCHD2^{T61I} recruits CSNK1E/D, which phosphorylates NEFL and α-Synuclein to generate aggresomes, subsequently resulting in neurodegeneration.

These results were confirmed using CHCHD2^{T61I} patient-derived iPS cells and their isogenic control cells, in which the T61I mutation was corrected by CRISPR/Cas9 (Fig EV5A). We generated TH-expressing dopaminergic neurons from these iPS cells (Fig EV5B and C) according to the established procedure described previously (Ikeda *et al*, 2019), by which these neurons are equivalently differentiated as assessed using the neuronal markers βIII-tubulin and TH (Ikeda *et al*, 2019). In CHCHD2^{T61I} dopaminergic neurons, we observed CHCHD2^{T61I} puncta in the cytoplasm (Figs 8A and EV5D), and their colocalization with CDNK1E/D (Fig 8B), phospho-NEFL^{472} (Figs 8C and EV5E), phospho-α-Synuclein^{129} (Figs 8D and EV5F), and aggresomes (Figs 8E and EV5G). These abnormalities were not observed in control iPS-derived neurons or mutation-corrected neurons (Figs 8A–E and EV5D–G). Treatment with PF-670462 also substantially suppressed the puncta formation of phospho-NEFL^{472} (Figs 8F and EV5H) and phospho-α-Synuclein^{129} (Figs 8G and EV5I), and the formation of aggresomes (Figs 8H and EV5J), although it did not suppress the mislocalization of CHCHD2^{T61I} (Fig EV5K and L). Finally, we analyzed the vulnerability of patient iPS-derived dopaminergic neurons. Abnormal cells appeared 15–20 days after the induction of differentiation only in patient-derived dopaminergic neurons. Analysis of active caspase-3, a marker of apoptosis, demonstrated that a larger number of CHCHD2^{T61I} iPS-derived dopaminergic neurons expressed active caspase-3 than WT and isogenic dopaminergic neurons (Appendix Fig S18A and B). CHCHD2^{T61I} iPS-derived neurons had abnormal cell shapes with shortened neurites (Appendix Fig S18C and D). Furthermore, PF-670462 substantially suppressed caspase-3 activation and the cell shape abnormalities of these cells (Appendix Fig S18E–H), indicating the involvement of CSNK1E/D in the pathogenesis of PD caused by CHCHD2^{T61I} (Fig 9).

© 2023 The Authors

Discussion

The mechanism by which CHCHD2^{T61I} causes neurodegenerative diseases has not been fully elucidated. In this study, we showed that CHCHD2^{T61I} localizes outside of mitochondria, and recruits Csnk1e/d, which phosphorylates Nefl and α-Synuclein to form aggregates, resulting in damage to dopaminergic neurons. This mechanism was observed in CHCHD2^{T61I} PD models of neuron-differentiated Neuro2a cells, dopaminergic neurons from patient-derived iPS cells, and disease model knock-in mice and Tg mice, as well as in the postmortem brain of patient. We thus believe that this is the actual pathogenesis of PD caused by the CHCHD2^{T61I} mutation (Fig 9).

CHCHD2 has an N-terminal MTS, which is cleaved upon entry into mitochondria, resulting in its 18-kD mature form. The T61I mutant is also detected at the 18-kD height, even though it is mainly localized in the cytoplasm. The mechanism by which the mutant CHCHD2^{T61I} is released from mitochondria is unclear, but the involvement of mitochondrial membrane pores formed during apoptosis, and mitochondrial permeability transition pores has been ruled out. This is because T61I localizes to the cytoplasm even in *Bax/Bak* double-deficient cells, which do not form apoptotic pores (Tsujimoto & Shimizu, 2007), and in cyclosporin A-treated cells, which do not form permeability transition pores (Tsujimoto & Shimizu, 2007). The other mutants, V66M and I80V, which are both mutations in the same central transmembrane region of T61I, were also detected as 18-kD proteins localized outside of the mitochondria, and phosphorylated α-Synuclein, suggesting the same pathogenesis as T61I. This suggests that mutations in the central transmembrane region of CHCHD2 may be important for its release from mitochondria and its binding to Csnk1e/d.

In this study, Csnk1e was isolated as a protein that specifically binds to CHCHD2^{T61I}. Csnk1e and Csnk1d are highly homologous, and hence they are expected to share common substrates. In fact, the phosphorylation of Nefl and α-Synuclein was substantially reduced by Csnk1e knockdown and was further reduced by the double knockdown of Csnk1e and Csnk1d. We here first showed the involvement of Csnk1e/d in the pathogenesis of CHCHD2^{T61I}-induced PD. Regarding other types of PD, although Csnk1d and Csnk2 have been reported to be able to phosphorylate Nefl and α-Synuclein (Okochi *et al*, 2000; Dzamko *et al*, 2014; Tenreiro *et al*, 2014; Rutherford *et al*, 2016), the involvement of Csnk1e has not been reported. Furthermore, to our knowledge, there have been no reports to date indicating a direct link between Csnk1e/d and the pathogenesis of PD. Therefore, the extent to which Csnk1e/d is involved in PD other than PD induced by CHCHD2^{T61I} is currently unknown and needs further investigation.

In this study, we found that the CHCHD2^{T61I} mutation has a greater effect in the SNpc than in the cortex or cerebellum. mRNA expression levels did not differ significantly among these regions according to the database (Wilhelm *et al*, 2014; Uhlen *et al*, 2015), and indeed no differences in protein expression levels were observed in CHCHD2^{T61I} knock-in mice (Appendix Fig S11B). One reason for this specificity is that CHCHD2^{T61I} tends to mislocalize more specifically to the cytoplasm in neurons of the SNpc (Figs 5G and EV4H, Appendix Figs S11C and S15B). This might be because the amount and type of proteins that bind and keep CHCHD2^{T61I} in the mitochondria may differ among brain regions. In addition, Csnk1e might be specifically upregulated by extramitochondrial CHCHD2^{T61I} in the SNpc (Figs 5L and EV4B, Appendix Figs S11B and S15C). This increase in Csnk1e is expected to accelerate the recruitment of phospho-NEFL^{472} and phospho-α-Synuclein^{129}, rendering dopaminergic neurons to be more prone to abnormalities. These two factors may be responsible for the specific damage to dopaminergic neurons by CHCHD2^{T61I}.

Materials and Methods

Mice

CHCHD2^{KO} mice have been described previously (Sato *et al*, 2021). Mice were housed in a 12 h light /12 h dark cycle at approximately 23°C and 40% relative humidity at the Laboratory for Recombinant Animals of Tokyo Medical and Dental University, Tokyo, Japan. The Tokyo Medical and Dental University Ethics Committee for Animal Experiments approved all experiments in this study, and all experiments were performed according to their regulations.

Generation of Chchd2^{T61I} knock-in mice

For the generation of a targeting vector for Chchd2^{T61I} knock-in mice, DNA fragments were amplified by PCR from tail genomic DNA and introduced into the pUC19 vector (Fig EV2C), and their sequences were confirmed by sequencing. One-cell stage zygotes were obtained by mating C57BL6/J males and females. CrRNA, namely 5′-cr1 (5′-ATAGA TCGTT TACCA CAGTC GACAA ATGG-3′) and 3′-cr2 (5′-TCCGA ACAGA TCATA AGGAG CTCTG GTCC-3′) were designed, chemically synthesized, and purified by polyacrylamide gel electrophoresis. A mixture of recombinant Cas9 proteins (NEB; Thousand Oaks, CA, USA), crRNA, and the *Chchd2^{T61I}* targeting vector were injected into pronuclei of one-cell stage zygotes using a micromanipulator/microscope (Leica, Wetzlar, Germany) and injector (Eppendorf, Hauppauge, NY, USA), and transferred into pseudopregnant ICR female mice (CLEA Japan) (Takao *et al*, 2020). We obtained two lines of *Chchd2^{T61I}/Zbed5^{WT}* mice, and the phenotypes of both lines were almost the same. The phenotypes were observed in both male and female mice. We used male mice in the experiments. Genotyping was performed using by amplification of a PCR product (9,227 bp including the modified genome sequence) from tail genomic DNA followed by insertion into a vector and sequencing. Genotyping was also performed by amplification of a PCR product (each arm, about 3,000 bp) followed by digestion with restriction enzymes. The primers used were purchased from Eurofins and are listed in Appendix Table S1.

Generation of neuron-specific Chchd2^{T61I} Tg mice

For the generation of a Chchd2^{T61I} Tg mouse line carrying the mouse *Chchd2^{T61I}* gene driven by the prion promoter (PrP), mouse *Chchd2* cDNA was purchased from Origene (MR223513). The introduction of point mutations into mouse *Chchd2* was performed using PCR with Pfu Turbo (Agilent Technologies). The introduction of an HA-tag sequence into the vector was performed using primers including the HA sequence. Mouse *Chchd2^{T61I}* cDNA was introduced into the pPrPpE1/E2,3sal vector. All constructs were confirmed by sequence analysis. The vector was digested by NotI to remove an

 © 2023 The Authors

unnecessary vector sequence (Appendix Fig S12A). The vector was then injected into pronuclei of one-cell stage zygotes using a micromanipulator/microscope and injector. Genotyping was performed by genome amplification by PCR (product about 600 bp) from tail genomic DNA. The expression of Chchd2^{T61I}-HA was confirmed by Western blotting. The phenotypes were observed in both male and female mice. We used male mice in these results. The primers used were purchased from Eurofins and are listed in Appendix Table S1.

Mouse rotarod and footprint analysis

Male mice were trained on a rotarod twice daily for 1 week before the analysis and then analyzed on an accelerating rotarod apparatus (Ugo Basile) set to accelerate from 4 to 40 rpm within a period of 300 s. Latency to fall was recorded for four trials, with an intermission of at least 5 min between each trial. Measurements from mice within the same treatment cohorts were averaged and expressed as ± standard deviation (SD). For footprint analysis, mice were trained to run in a straight line down a runway of white paper before the test. On the test day, forepaws and hindpaws were dipped in red and blue (non-toxic acrylic paint), respectively. Mice were then made to walk down the enclosed runway lined with white paper. Three trials each were performed on 3 consecutive days. We calculated the average sway length (3 position), stride length (8 position), and stance length (6 position) of each mouse and evaluated the averages and standard deviations of WT, knock-in, and Tg mice.

Immunohistochemistry

Frozen mouse brain sections were blocked with 5% goat serum in PBS, and cells were then stained with the indicated primary antibodies for 1 h at room temperature. After washing, the cells were stained with secondary antibodies for 1 h at room temperature, then mounted in Prolong Diamond Antifade reagent with DAPI (Thermo Fisher Scientific), and observed using a laser-scanning confocal microscope (LSM710, Zeiss). Paraffin sections of a patient's brain were deparaffinized in xylene, rehydrated through a graded series of ethanol, and then treated as described above for mouse brain sections.

Electron microscopy

Cells were fixed with 1.5% paraformaldehyde/3% glutaraldehyde in 0.1 M phosphate buffer (pH 7.2) followed by fixation in 1% OsO_4. After dehydration, ultrathin sections were stained with uranyl acetate and lead citrate and observed using a JEM-1010 electron microscope (JEOL Ltd.) at 80 kV.

Brain infusion

An Alzet osmotic pump (Model 2004, Alzet, USA) filled with a 0.5 mM PF-670462 or 5% DMSO in artificial sterile cerebrospinal fluid (CSF) was placed in a subcutaneous area of the back of mice at 16 weeks of age. The CSF solution from the osmotic pump was infused through a catheter (BRAIN INFUSION KIT3 1–3 mm, Alzet, USA) implanted in the right frontal region of the brain (~1.5 mm lateral, and 3.0–4.0 mm posterior from the bregma) at a rate of 0.25 µl/h. The osmotic pump was replaced every 28 days (50 ng/g body weight/day) and removed before the rotarod test.

Antibodies and chemicals

The antibodies used in this study are listed in Appendix Table S2. PF-670462 was purchased from Sigma-Aldrich. All other chemicals were purchased from Nacalai Tesque.

Plasmid construction

The HA-tagged human *CHCHD2* plasmid was previously reported (Funayama *et al*, 2015). The HA-tagged mouse *Chchd2* plasmid was purchased from Origene (MR223513). The introduction of point mutations into human *CHCHD2* was performed using PCR with Pfu Turbo. The primers used are listed in Appendix Table S1.

Cell culture and DNA/siRNA transfection

Mouse embryonic fibroblasts were generated from WT and *Chchd2*^{KO} embryos as previously reported (Sato *et al*, 2021). MEFs and Neuro2a cells were cultured in Dulbecco's modified Eagle's medium supplemented with 2 mM L-glutamine, 1 mM sodium pyruvate, 0.1 mM nonessential amino acids, 10 mM HEPES/Na^+ (pH 7.4), 0.05 mM 2-mercaptoethanol, 100 U/ml penicillin, 100 µg/ml streptomycin, and 10% FBS, and incubated as previously reported (Torii *et al*, 2020b). MEFs (1×10^6) were transfected with the Amaxa electroporation system (Lonza) according to the manufacturer's instructions. Neuro2a cells were transfected with Lipofectamine 2000 (Thermo Fisher Scientific) according to the manufacturer's instructions. For DNA transfection, the human *CHCHD2*-HA plasmid was transfected into Neuro2a cells with Lipofectamine 2000 and selected using G418. The siRNAs were purchased from Horizon/Dharmacon (SMARTpool) and transfected using Lipofectamine 2000.

Immunoprecipitation

Cells were harvested and lysed using cell lysis buffer containing 20 mM HEPES (pH 7.5), 100 mM NaCl, 1.5 mM $MgCl_2$, 1 mM EGTA, 10 mM $Na_2P_2O_7$, 10% glycerol, 1% Nonidet P-40, 1 mM dithiothreitol, 1 mM Na_3VO_4, and 1% protease inhibitor cocktail. Immunoprecipitation was performed using the indicated antibody in the presence of protein-G Sepharose (GE Healthcare) for 2 h at 4°C. The beads were then washed two times with PBS.

Mass-spectrometry analysis

Mass-spectrometry analysis was performed as follows. Anti-HA immunoprecipitates from CHCHD2^{WT} or CHCHD2^{T61I}-expressing Neuro2a cells were eluted with 200 µl of 2% sodium deoxycholate. Samples were mixed with 80 µl of 5 M urea, 20 µl of 1 M NH_4HCO_3, 95 µl of water, and 2 µg trypsin (Promega, V5280, Madison, WI, USA) and incubated at 37°C on a rotating incubator for 20 h. Samples were mixed with 80 µl of 5% formic acid and centrifuged at 15,000 *g* at room temperature for 10 min. Supernatants were then mixed with 480 µl of ethyl acetate. After vortexing, samples were centrifuged at 15,000 *g* for 10 min, and the top ethyl acetate layer was removed. The peptide samples were dried using an evaporator (GeneVac) at 40°C and then suspended in 20 µl of 0.1% formic acid. The peptides were passed through C18 stage tips (Thermo Fisher)

© 2023 The Authors

and washed twice with 20 μl of 2% acetonitrile and 0.1% formic acid. Bound peptides were eluted from the C18 column twice with 20 μl of 80% acetonitrile and 0.1% formic acid. The samples were dried up at room temperature overnight and then resuspended in 20 μl of 0.1% formic acid. Shotgun mass-spectrometry analysis was performed as described previously (Hu *et al*, 2021).

Immunofluorescence analysis

Cells were fixed in 4% paraformaldehyde containing 8 mM EGTA for 10 min and then permeabilized using 0.5% Triton X-100 for 5 min. Cells were then stained with the indicated primary antibodies for 1 h at room temperature. After washing, the cells were stained with secondary antibodies, mounted in Prolong Diamond Antifade reagent with DAPI, and observed using a laser-scanning confocal microscope (LSM710, Zeiss). Data analysis was performed using Zen software 2012 (Zeiss), Adobe Photoshop CS5.1, Illustrator CS5.1, and Image J software.

Duolink *in situ* proximity ligation assay (PLA)

Cells were fixed in 4% paraformaldehyde containing 8 mM EGTA for 10 min and then permeabilized using 0.5% Triton X-100 for 5 min. Cells were then stained with the indicated primary antibodies overnight at 4°C. After washing, the cells were assayed with Duolink *in situ* PLA reagents according to the manufacturer's instructions (Sigma-Aldrich), mounted in Prolong Diamond Antifade reagent with DAPI, and observed using a laser-scanning confocal microscope (LSM710, Zeiss). Data analysis was performed using Zen, Adobe Photoshop CS5.1, and Illustrator CS5.1 software.

Cell fractionation assay

Neuro2a cells were transfected with the indicated plasmids. At 24 h after transfection, cells were collected by a scraper in 300 μl PBS buffer. Cell lysates were centrifuged at 500 *g* for 10 min at 4°C. After aspiration of the supernatant, cells were treated by one freeze–thaw cycle and assayed with ProteoExtract Subcellular Proteome Extraction kit (Millipore, 549790) according to the manufacturer's protocol. Cell lysates were fractionated into cytosol, organelles (including mitochondria), nuclei, and cytoskeleton (including insoluble matter).

Mitochondrial isolation

Mitochondria were isolated from the cells and the brains of mice by differential centrifugation. Briefly, cells and brains were washed and homogenized in a sufficient volume of 0.3 M mannitol solution containing 10 mM HEPES, 0.2 mM EDTA and 0.1% BSA (pH 7.4), and centrifuged at 740 *g* for 10 min. Pellets were harvested as non-mitochondrial insoluble material. Supernatants were centrifuged at 3,000 *g* for 8 min followed by 7,600 *g* for 5 min. Pellets were suspended in 0.3 M mannitol solution containing 10 mM HEPES, 3 mM EGTA and 0.1% BSA (pH 7.4), and centrifuged at 740 *g* for 10 min. Supernatants were centrifuged at 6,700 *g* for 10 min. Finally, pellets were resuspended in 0.3 M mannitol solution containing 10 mM HEPES, 3 mM EGTA, and 0.1% BSA (pH 7.4).

Immunoblot analysis

Cells and mouse tissues were lysed in cell lysis buffer containing 20 mM HEPES (pH 7.5), 100 mM NaCl, 1.5 mM $MgCl_2$, 1 mM EGTA, 10 mM $Na_2P_2O_7$, 10% glycerol, 1% Nonidet P-40, 1 mM dithiothreitol, 1 mM Na_3VO_4, and 1% protease inhibitor cocktail. After vortexing for 15 s, insoluble material of mouse tissues was removed by centrifugation. Supernatants were loaded onto 5–20%, or 15% SDS-polyacrylamide gels. After electrophoresis, the proteins were blotted onto PVDF membranes. The membranes were blocked with 5% skim milk in TBS containing 0.05% Tween-20 (TBS-T), and incubated with a primary antibody overnight at 4°C. After washing with TBS-T, the membranes were incubated with a horseradish peroxidase-labeled secondary antibody and visualized with Chemi-Lumi One Super reagent. All experiments were conducted at least in duplicate.

Brain autopsy, culture of iPSCs, and their differentiation into dopaminergic neurons

Brain tissues from a neurologically normal older subject as a control and a previously reported PD patient harboring the CHCHD2 p.T61I mutation (Ikeda *et al*, 2019) were analyzed. Control (EKA4) and CHCHD2^{T61I} iPSCs (CHA11) were generated from dermal fibroblasts using episomal vectors as reported previously (Matsumoto *et al*, 2016; Ikeda *et al*, 2019). An isogenic control iPSC line (CHAI-191) was generated from CHA11 by the CRISPR/Cas9 system. A specific guide RNA (gRNA) for the c.182C>T mutation site at exon 2 in the *CHCHD2* gene (hCHCHD2_ex2_crRNA: CAGATGGCAATCACTGCAGC) was designed using CRISPRdirect (http://crispr.dbcls.jp/) and CRISPOR (http://crispor.tefor.net/). The donor DNA was designed using the WT allele and two additional synonymous mutations to introduce a *de novo* SacII site to help with the screening. The donor DNA, gRNA, and Cas9 nuclease protein (Nippon Gene) were coeletroporated into CHA11 iPSCs using 4D-Nucleofector (Lonza). Single colonies were selected by PCR followed by SacII digestion. Clone that were positive by SacII digestion were sequenced by Sanger sequencing and showed correction of the mutation.

Human iPSCs were cultured dish coated with the lamin 511 E8 fragment (iMatrix-511, Nippi) with StemfitAK02N media (Ajinomoto) and then differentiated into midbrain dopaminergic neurons was performed basically according to a previously reported method (Ikeda *et al*, 2019), with slight modifications. iPSCs were cultured in StemfitAK02 medium supplemented with 3 μM SB431542 (TOCRIS), 3 μM dorsomorphin (Sigma-Aldrich), and 3 μM CHIR99021 (Nacalai Tesque) for 5 days. To make primary neurospheres, iPSC colonies were dissociated into single cells by 0.5 × TrypLE Select (Life Technologies) and cultured at a density of 1 × 10^4 cells/ml in KBM Neural Stem Cell medium (KOHJIN BIO) supplemented with B27 (Life Technologies), 5 μM Y27632 (Wako), 20 ng/ml basic fibroblast growth factor (bFGF, Pepro Tech), and 2 μM SB431542 in 4% O^2, and 3 μM CHIR99021, and then 2 μM purmorphamine (Millipore) was added to the culture medium after 3 days. After 7–10 days of primary neurosphere generation, cells were dissociated by TrypLE Select, and cultured at a density of 4 × 10^4 cells/ml in KBM Neural Stem Cell medium with B27, 5 μM Y27632, 20 ng/ml bFGF, 2 μM SB431542, 3 μM CHIR99021, and 2 μM purmorphamine to making secondary neurospheres. After 7 days of secondary neurosphere

© 2023 The Authors

culture, the neurospheres were dissociated and seeded onto poly-L-ornithine (Sigma-Aldrich) and fibronectin (Corning)-coated coverslips in 48-well culture plates in Neurobasal plus medium (Life Technologies) supplemented with B27 plus (Life Technologies), Culture One (Life Technologies), 20 ng/ml brain-derived neurotrophic factor (BioLegend), glial cell-derived neurotrophic factor (Pepro Tech), 200 μM ascorbic acid (Sigma-Aldrich), 0.5 mM dibutyryl-cAMP (Nakalai Tesque), 1 ng/ml TGF-β (Biolegend), and 10 μM DAPT (Sigma), and cultured for 14 days before analysis. For the cell population assay, the numbers of Hoechst-positive total nuclei, βIII-tubulin-positive cells, and TH-positive cells among DA neurons were analyzed.

The Tokyo Medical and Dental University and Juntendo University Ethics Committees for Human Experiments approved all experiments in this study, and all experiments were performed according to their regulations and adhered to the World Medical Association (WMA) Declaration of Helsinki and to the Department of Health and Human Services Belmont Report. Informed consent was obtained from all participants.

Statistical analysis

Results are expressed as the mean ± SD. Statistical analyses were performed using Excel and Prism 5 and 8 (GraphPad) software. Comparisons of two datasets were performed using unpaired two-tailed Student *t*-tests. All other comparisons of multiple datasets were performed using one-way ANOVA followed by the Tukey *post hoc* test. A *P*-value of less than 0.05 was considered to indicate a statistically significant difference between two groups. Details on the *P*-values of significant differences for figures are summarized in the source data.

Data availability

The data of identified proteins from mass spectrometry (Fig 3A) is included within the source data.

Expanded View for this article is available online.

Acknowledgements

This study was supported by JSPS KAKENHI Grant-in-Aid for Scientific Research (S) (23H05480), (A) (20H00467), and by MEXT KAKENHI Grant-in-Aid for Scientific Research on Innovative Areas (17H06414, 22H04639, 23H04773) to SS. This study was also supported by AMED-CREST (JP23gm1410012) and the Project for Psychiatric and Neurological Disorders (JP22wm0525028) from the Japan Agency for Medical Research and development, AMED, and by the Joint Usage/Research Program of Medical Research Institute, Tokyo Medical and Dental University to SS. This study was also supported by grants from the Japan Agency for Medical Research and Development (GAPFREE, JP19ak0101112/JP19ak0101125) to WA and NH and the Acceleration Program for Intractable Disease Research Utilizing Disease-specific iPS Cells (JP21bm0804003) to WA and NH, as well as by a grant of Strategic Research Foundation Grant-aided Project for Private Universities from MEXT.

Author contributions

Shigeomi Shimizu: Conceptualization; supervision; funding acquisition; writing – review and editing. **Satoru Torii:** Conceptualization; investigation; visualization; writing – original draft. **Kei-ichi Ishikawa:** Resources; methodology. **Shigeto Sato:** Resources; methodology. **Hajime Tajima Sakurai:** Investigation. **Shinya Honda:** Investigation. **Masaya Ono:** Methodology. **Wado Akamatsu:** Resources; methodology. **Nobutaka Hattori:** Resources; supervision. **Satoko Arakawa:** Investigation. **Daisuke Taniguchi:** Resources; investigation. **Yuuichi Hiraoka:** Resources; investigation.

The paper explained

Problem
Parkinson's disease (PD) is the second most common neurodegenerative disease, characterized by progressive resting tremors, rigidity, bradykinesia, gait disturbances, postural instability, and dementia. Although most PD cases are sporadic, approximately 10% are familial. The pathogenesis of PD is yet to be established, but accumulation of α-Synuclein is likely involved in progression of this disease.

Results
We investigated one of causative factors for familial PD, coiled-coil-helix-coiled-coil-helix domain containing 2 (CHCHD2/PARK22) mutations. We found the CHCHD2^{T61I} mutant recruited casein kinase and induced phosphorylation and accumulation of α-Synuclein, resulting dopaminergic neuronal loss in the mouse midbrain. Pharmacological inhibition of casein kinase 1 epsilon and delta (Csnk1e/d) improved dopaminergic neuron damage.

Impact
These results reveal the pathogenesis of familial PD caused by CHCHD2 mutations. Furthermore, these data provide a foundation to test whether Csnk1e/d could be a pharmacological biomarker of other PD types, and whether its inhibitor could be a candidate for PD therapy.

Disclosure and competing interests statement

The authors declare that they have no conflict of interest.

References

Badura L, Swanson T, Adamowicz W, Adams J, Cianfrogna J, Fisher K, Holland J, Kleiman R, Nelson F, Reynolds L *et al* (2007) An inhibitor of casein kinase I epsilon induces phase delays in circadian rhythms under free-running and entrained conditions. *J Pharmacol Exp Ther* 322: 730 – 738

Basso M, Giraudo S, Corpillo D, Bergamasco B, Lopiano L, Fasano M (2004) Proteome analysis of human substantia nigra in Parkinson's disease. *Proteomics* 4: 3943 – 3952

Bibian M, Rahaim RJ, Choi JY, Noguchi Y, Schurer S, Chen W, Nakanishi S, Licht K, Rosenberg LH, Li L *et al* (2013) Development of highly selective casein kinase 1delta/1epsilon (CK1delta/epsilon) inhibitors with potent antiproliferative properties. *Bioorg Med Chem Lett* 23: 4374 – 4380

Cornelissen T, Spinazzi M, Martin S, Imberechts D, Vangheluwe P, Bird M, De Strooper B, Vandenberghe W (2020) CHCHD2 harboring Parkinson's disease-linked T61I mutation precipitates inside mitochondria and induces precipitation of wild-type CHCHD2. *Hum Mol Genet* 29: 1096 – 1106

Dzamko N, Zhou J, Huang Y, Halliday GM (2014) Parkinson's disease-implicated kinases in the brain; insights into disease pathogenesis. *Front Mol Neurosci* 7: 57

Exner N, Lutz AK, Haass C, Winklhofer KF (2012) Mitochondrial dysfunction in Parkinson's disease: molecular mechanisms and pathophysiological consequences. *EMBO J* 31: 3038 – 3062

Funayama M, Ohe K, Amo T, Furuya N, Yamaguchi J, Saiki S, Li Y, Ogaki K, Ando M, Yoshino H *et al* (2015) CHCHD2 mutations in autosomal dominant late-onset Parkinson's disease: a genome-wide linkage and sequencing study. *Lancet Neurol* 14: 274 – 282

Gietzen KF, Virshup DM (1999) Identification of inhibitory autophosphorylation sites in casein kinase I epsilon. *J Biol Chem* 274: 32063 – 32070

Guo G, Wang K, Hu SS, Tian T, Liu P, Mori T, Chen P, Johnson CH, Qin X (2019) Autokinase activity of casein kinase 1 delta/epsilon governs the period of mammalian circadian rhythms. *J Biol Rhythms* 34: 482 – 496

Hanna-Addams S, Liu S, Liu H, Chen S, Wang Z (2020) CK1alpha, CK1delta, and CK1epsilon are necrosome components which phosphorylate serine 227 of human RIPK3 to activate necroptosis. *Proc Natl Acad Sci USA* 117: 1962 – 1197

Hill WD, Lee VM, Hurtig HI, Murray JM, Trojanowski JQ (1991) Epitopes located in spatially separate domains of each neurofilament subunit are present in Parkinson's disease Lewy bodies. *J Comp Neurol* 309: 150 – 160

Hu X, Ono M, Chimge NO, Chosa K, Nguyen C, Melendez E, Lou CH, Lim P, Termini J, Lai KKY *et al* (2021) Differential Kat3 usage orchestrates the integration of cellular metabolism with differentiation. *Cancers (Basel)* 13: 5884

Huang X, Wu BP, Nguyen D, Liu YT, Marani M, Hench J, Benit P, Kozjak-Pavlovic V, Rustin P, Frank S *et al* (2018) CHCHD2 accumulates in distressed mitochondria and facilitates oligomerization of CHCHD10. *Hum Mol Genet* 27: 3881 – 3900

Ikeda A, Nishioka K, Meng H, Takanashi M, Hasegawa I, Inoshita T, Shiba-Fukushima K, Li Y, Yoshino H, Mori A *et al* (2019) Mutations in CHCHD2 cause alpha-synuclein aggregation. *Hum Mol Genet* 28: 3895 – 3911

Imai Y, Meng H, Shiba-Fukushima K, Hattori N (2019) Twin CHCH proteins, CHCHD2, and CHCHD10: key molecules of Parkinson's disease, amyotrophic lateral sclerosis, and frontotemporal dementia. *Int J Mol Sci* 20: 908

Kee TR, Espinoza Gonzalez P, Wehinger JL, Bukhari MZ, Ermekbaeva A, Sista A, Kotsiviras P, Liu T, Kang DE, Woo JA (2021) Mitochondrial CHCHD2: disease-associated mutations, physiological functions, and current animal models. *Front Aging Neurosci* 13: 660843

Liu Y, Clegg HV, Leslie PL, Di J, Tollini LA, He Y, Kim TH, Jin A, Graves LM, Zheng J *et al* (2015) CHCHD2 inhibits apoptosis by interacting with Bcl-x L to regulate Bax activation. *Cell Death Differ* 2: 1035 – 1046

Matsumoto T, Fujimori K, Andoh-Noda T, Ando T, Kuzumaki N, Toyoshima M, Tada H, Imaizumi K, Ishikawa M, Yamaguchi R *et al* (2016) Functional neurons generated from T cell-derived induced pluripotent stem cells for neurological disease modeling. *Stem Cell Reports* 6: 422 – 435

Meng H, Yamashita C, Shiba-Fukushima K, Inoshita T, Funayama M, Sato S, Hatta T, Natsume T, Umitsu M, Takagi J *et al* (2017) Loss of Parkinson's disease-associated protein CHCHD2 affects mitochondrial crista structure and destabilizes cytochrome c. *Nat Commun* 8: 15500

Okochi M, Walter J, Koyama A, Nakajo S, Baba M, Iwatsubo T, Meijer L, Kahle PJ, Haass C (2000) Constitutive phosphorylation of the Parkinson's disease associated alpha-synuclein. *J Biol Chem* 275: 390 – 397

Rutherford NJ, Brooks M, Giasson BI (2016) Novel antibodies to phosphorylated alpha-synuclein serine 129 and NFL serine 473 demonstrate the close molecular homology of these epitopes. *Acta Neuropathol Commun* 4: 80

Sato S, Noda S, Torii S, Amo T, Ikeda A, Funayama M, Yamaguchi J, Fukuda T, Kondo H, Tada N *et al* (2021) Homeostatic p62 levels and inclusion body formation in CHCHD2 knockout mice. *Hum Mol Genet* 30: 443 – 453

Takao T, Hiraoka Y, Kawabe K, Yamada D, Ming L, Tanaka K, Sato M, Takarada T (2020) Establishment of a tTA-dependent photoactivatable Cre recombinase knock-in mouse model for optogenetic genome engineering. *Biochem Biophys Res Commun* 526: 213 – 217

Tenreiro S, Eckermann K, Outeiro TF (2014) Protein phosphorylation in neurodegeneration: friend or foe? *Front Mol Neurosci* 7: 42

Torii S, Kasai S, Yoshida T, Yasumoto KI, Shimizu S (2020a) Mitochondrial E3 ubiquitin ligase Parkin: relationships with other causal proteins in familial Parkinson's disease and its substrate-involved mouse experimental models. *Int J Mol Sci* 21: 1202

Torii S, Yamaguchi H, Nakanishi A, Arakawa S, Honda S, Moriwaki K, Nakano H, Shimizu S (2020b) Identification of a phosphorylation site on Ulk1 required for genotoxic stress-induced alternative autophagy. *Nat Commun* 11: 1754

Tsujimoto Y, Shimizu S (2007) Role of the mitochondrial membrane permeability transition in cell death. *Apoptosis* 12: 835 – 840

Uhlen M, Fagerberg L, Hallstrom BM, Lindskog C, Oksvold P, Mardinoglu A, Sivertsson A, Kampf C, Sjostedt E, Asplund A *et al* (2015) Proteomics. Tissue-based map of the human proteome. *Science* 347: 1260419

Wilhelm M, Schlegl J, Hahne H, Gholami AM, Lieberenz M, Savitski MM, Ziegler E, Butzmann L, Gessulat S, Marx H *et al* (2014) Mass-spectrometry-based draft of the human proteome. *Nature* 509: 582 – 587

License: This is an open access article under the terms of the Creative Commons Attribution License, which permits use, distribution and reproduction in any medium, provided the original work is properly cited.

Expanded View Figures

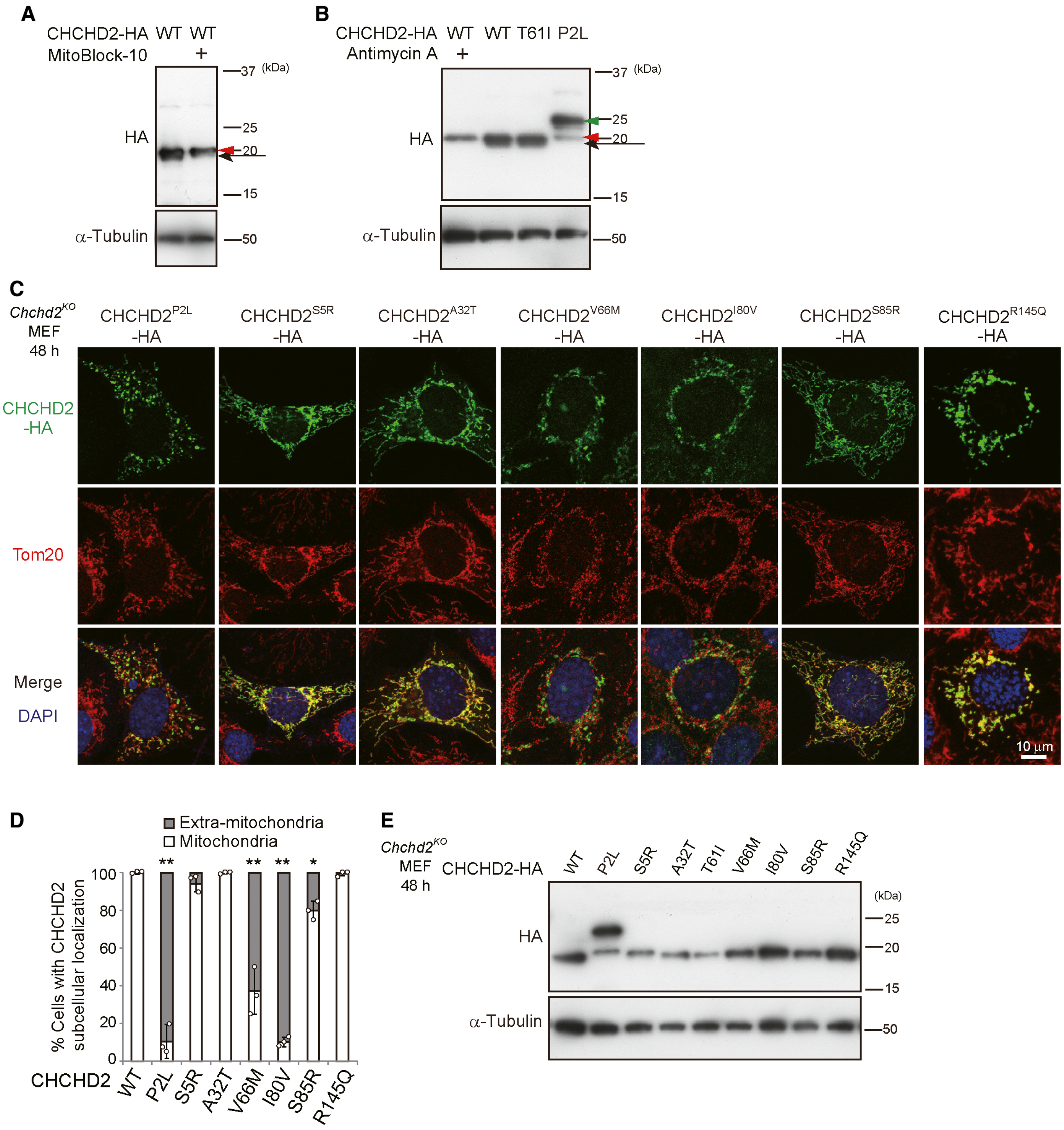

Figure EV1.

◀

Figure EV1. Expression of pathogenic mutant CHCHD2 proteins.

A, B Effects of mitochondrial protein import inhibitors on Western blot band sizes of CHCHD2 mutant proteins. *Chchd2*^{KO} cells were transfected with the *CHCHD2*^{WT}*-HA*, *CHCHD2*^{T61I}*-HA*, and *CHCHD2*^{P2L}*-HA* plasmids, and treated with compounds that inhibit mitochondrial protein import (MitoBlock-10 [20 μM] and antimycin A [10 μM]) for 16 h. At 48 h after transfection, cell lysates were subjected to Western blotting. Addition of these compounds altered the band size of CHCHD2^{WT} from 18 kD (black arrows) to 20 kD (red arrowheads). The band size of CHCHD2^{T61I} and CHCHD2^{P2L} is 18 kD (black arrows) and 20 kD (red arrowheads) plus 23 kD (green arrowhead), respectively.

C–E The indicated pathogenic mutant CHCHD2 proteins were expressed in *Chchd2*^{KO} MEFs by gene transfection. After 48 h, cells were permeabilized and stained with anti-HA and anti-Tom20 antibodies (C, D), or cells were collected and lysates were subjected to Western blotting using an anti-HA antibody (E). In (C), representative images are shown. In (D), the number of cells displaying mitochondrial CHCHD2 and extra-mitochondrial CHCHD2 puncta were quantified ($n \geq 100$ cells in each experiment). Data are shown as the mean ± SD ($n = 3$).

 © 2023 The Authors

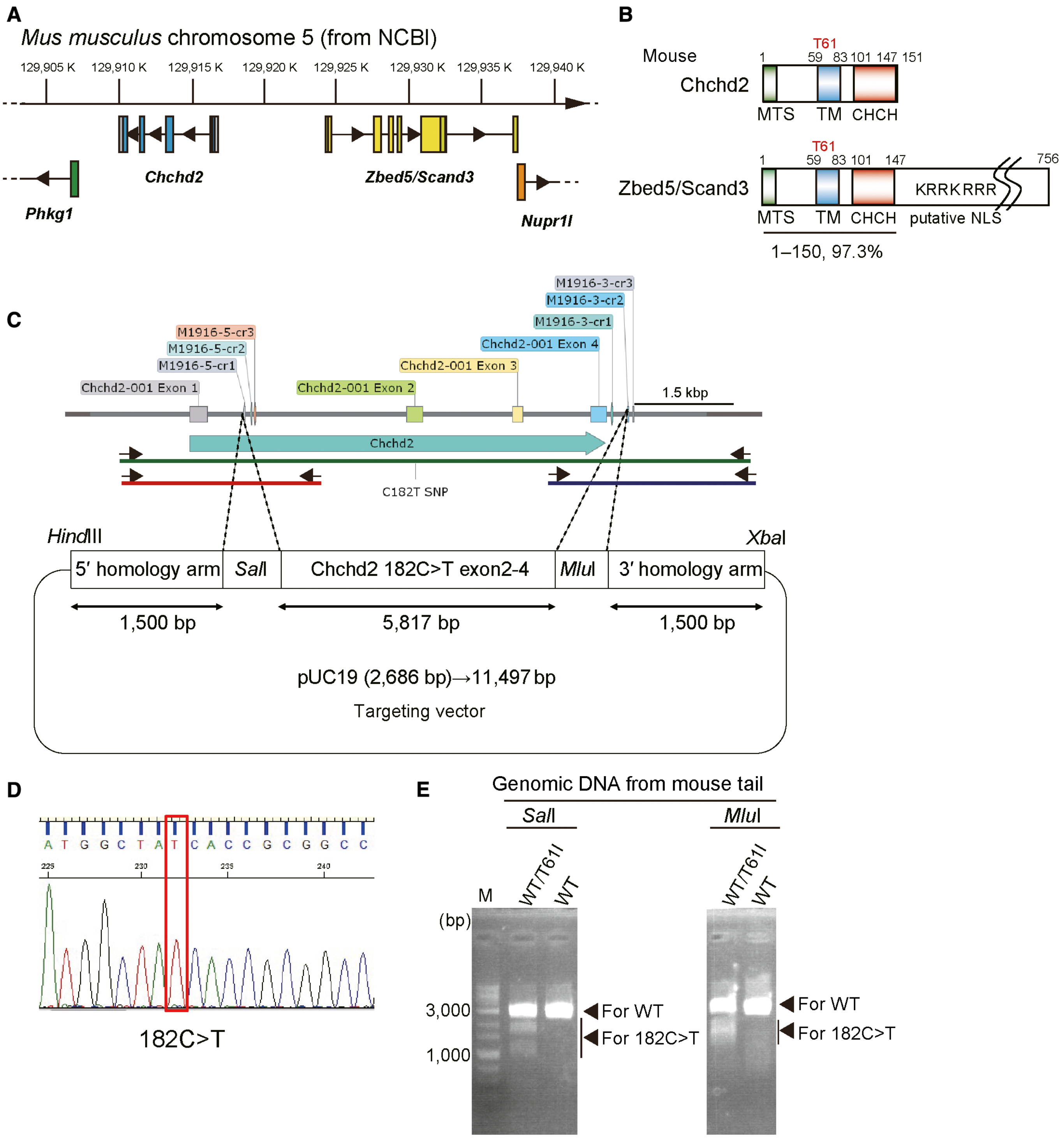

A
Mus musculus chromosome 5 (from NCBI)
129,905 K 129,910 K 129,915 K 129,920 K 129,925 K 129,930 K 129,935 K 129,940 K
Chchd2
Zbed5/Scand3
Phkg1
Nupr1l
B
Mouse
T61
1 59 83 101 147 151
Chchd2
MTS TM CHCH
T61
1 59 83 101 147 756
Zbed5/Scand3
KRRKRRR
MTS TM CHCH putative NLS
1–150, 97.3%
C
M1916-3-cr3
M1916-3-cr2
M1916-3-cr1
Chchd2-001 Exon 4
M1916-5-cr3
M1916-5-cr2
Chchd2-001 Exon 3
M1916-5-cr1
Chchd2-001 Exon 1
Chchd2-001 Exon 2
1.5 kbp
Chchd2
C182T SNP
HindIII
XbaI
5′ homology arm
SalI
Chchd2 182C>T exon2-4
MluI
3′ homology arm
1,500 bp
5,817 bp
1,500 bp
pUC19 (2,686 bp)→11,497 bp
Targeting vector
D
A T G G C T A T C A C C G C G G C C
225 230 235 240
182C>T
E
Genomic DNA from mouse tail
SalI
MluI
M
WT/T61I
WT
WT/T61I
WT
(bp)
3,000
1,000
For WT
For 182C>T
For WT
For 182C>T

Figure EV2.

Figure EV2. Generation of Chchd2^{T61I} knock-in mice.

A Schematic representation of the gene map of *Mus musculus* chromosome 5.
B Structures of the Chchd2 and Zbed5/Scand3 proteins. The protein sequence of Chchd2 is very similar (97.3%) to that of the N-terminal domain of Zbed5/Scand3.
C Schematic representation of the targeting vector and targeted allele of the *Chchd2* gene. The 5.8-kb region of the mouse *Chchd2* gene, including exons 2–4, was recombined with the C182T SNP-mutated sequence. A 1.5-kb 5′ fragment and a 1.5-kb 3′ fragment were used as the homologous arms. Small arrows indicate the position of the primers used for genotyping PCR. The green line indicates the PCR product used for sequencing. Red and blue lines indicate PCR products for *Sal* I and *Mlu* I, respectively.
D Genotyping was performed by genome amplification of by PCR (green line in (C), about 9,000 bp including a modified genome sequence) from tail genomic DNA followed by sequencing.
E Genotyping was also performed by genome amplification by PCR (red and blue lines in (C), about 3,000 bp) followed by digestion with the respective restriction enzymes. M indicates a lane of DNA markers. The primers used are listed in Appendix Table S1.

Figure EV3. Observation of inclusion bodies and cell death in DA neurons of Chchd2$^{T61I/T61I}$ knock-in mice by EM.

A–C Sections of DA neurons in the SNpc of CHCHD2$^{T61I/T61I}$ knock-in mice. Magnified images of the area within the pink square in the top panels are shown in the middle panels, and those of the orange squares in the middle panels are shown in the bottom panels. (A, B) DA neurons contained an inclusion body (Inc). The nucleus (N), mitochondria (Mt), and Golgi (G) appeared almost normal. (C) A dying neuron containing an inclusion body (Inc) is shown. The nucleus appears shrunken, and mitochondria and Golgi are abnormally swollen. A low-magnified image is shown in Fig 5M.
D A normal DA neuron in the SNpc of a littermate WT littermate mouse is shown. A magnified image of the area within the pink square is shown in the bottom panel.

© 2023 The Authors

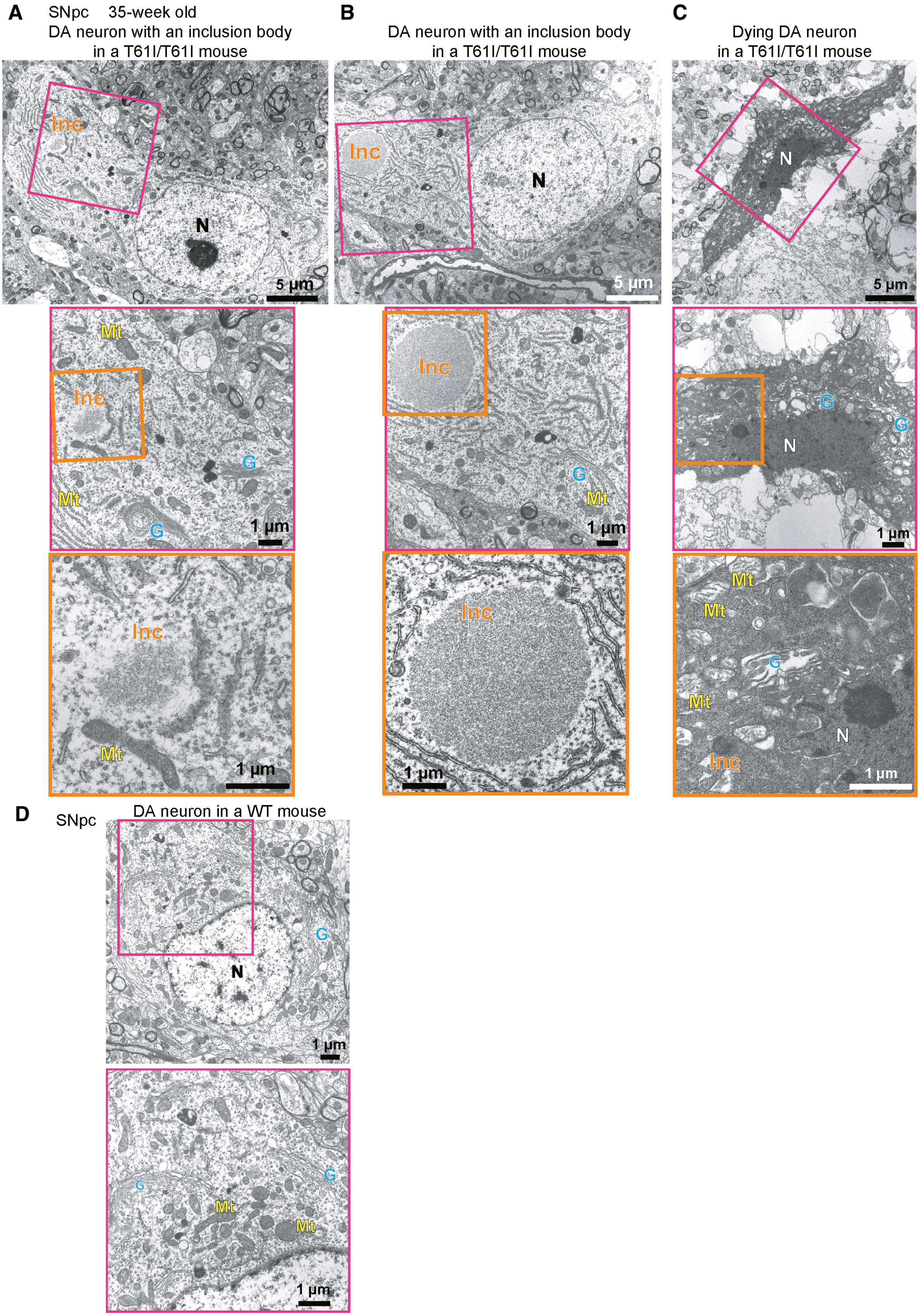
A
SNpc 35-week old
DA neuron with an inclusion body
in a T61I/T61I mouse
Inc
N
5 μm
Mt
Inc
G
Mt
G
1 μm
Inc
Mt
1 μm
B
DA neuron with an inclusion body
in a T61I/T61I mouse
Inc
N
5 μm
Inc
G
Mt
1 μm
Inc
1 μm
C
Dying DA neuron
in a T61I/T61I mouse
N
5 μm
G
G
N
1 μm
Mt
Mt
G
Mt
N
Inc
1 μm
D
SNpc
DA neuron in a WT mouse
G
N
1 μm
G
G
Mt
Mt
1 μm

Figure EV3.

Figure EV4. Reduced motor performance and dopaminergic neuronal loss in Chchd2^{T61I} Tg mice.

A The limb-clasping reflex was observed in Chchd2^{T61I} Tg mice at 30 weeks of age. Quantitative analysis is shown in Appendix Fig S13A.

B Midbrain and diencephalon brain lysates were obtained from WT and Tg mice, and expression of the indicated proteins was analyzed by Western blotting. Quantitative analysis of protein expression is shown in Appendix Fig S13C.

C, D Abnormal motor performance in Chchd2^{T61I} Tg mice. In (C), the footprint assay indicated motor deficits at 35 weeks. Quantitative analysis is shown in Appendix Fig S13B. In (D), the time that the indicated mice remained on the rotarod was measured. Data are shown as the mean ± SD ($n = 4$).

E–G Reduction in TH signals in the SNpc of Tg mice. In (E), brain cryosections were immunostained with the dopaminergic cell marker TH. Representative images of the SNpc and VTA are shown. Dashed lines indicate the SNpc and VTA regions. Bars = 200 μm. In (F), TH signals in the SNpc (average fluorescence intensity per region) are shown as the mean ± SD (n = 3). In (G), cryosections of the SNpc were immunostained with anti-HA and anti-TH antibodies. Arrowheads indicate Chchd2^{T61I}-HA puncta in TH-positive cells. Dashed lines indicate cell shapes.

H–L Extra-mitochondrial aggresome formation by p-Nefl^{473}, p-α-Syn^{129}, and Csnk1e/d in the SNpc of Tg mice. Cryosections of the SNpc were immunostained with anti-HA and anti-Ant1/2 (H), anti-p-Nefl^{473} (I), and anti-p-α-Syn^{129} antibodies (J), ProteoStat dye (K), and anti-Csnk1e/d antibodies (L). Arrowheads indicate extra-mitochondrial Chchd2^{T61I}-HA (H). In (I–L), arrowheads indicated the colocalization of puncta with Chchd2^{T61I}-HA and the indicated proteins. Dashed lines indicate cell shapes. Quantitative data are shown in Appendix Fig S13D–F.

M EM analysis of dopaminergic neurons in the SNpc of Chchd2^{T61I}-HA Tg mice. A dopaminergic neuron with an abnormal nuclear/cell shape and an inclusion body is shown. The red arrowhead indicates an inclusion body. N: nucleus. A magnified image of this cell is shown in Appendix Fig S14. In (D, F), comparisons were performed using unpaired two-tailed Student *t*-test. *$P < 0.05$; **$P < 0.01$.

© 2023 The Authors

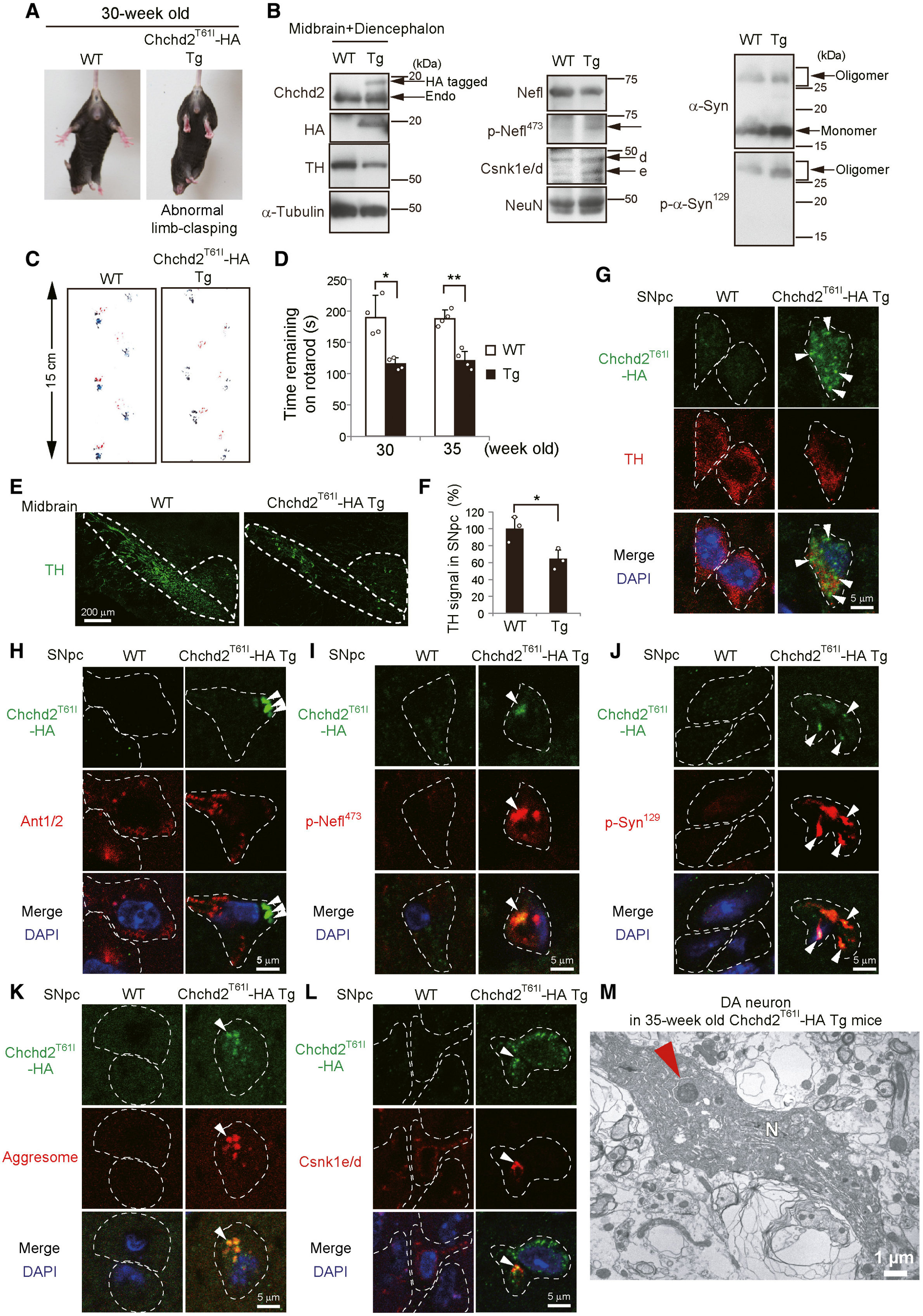
A
30-week old
Chchd2T61I-HA
WT
Tg
Abnormal limb-clasping
B
Midbrain+Diencephalon
WT Tg
(kDa)
Chchd2
HA tagged
Endo
HA
TH
α-Tubulin
Nefl
p-Nefl473
Csnk1e/d
NeuN
α-Syn
p-α-Syn129
Oligomer
Monomer
C
15 cm
D
Time remaining on rotarod (s)
WT
Tg
(week old)
E
Midbrain
TH
200 μm
F
TH signal in SNpc (%)
G
SNpc
Chchd2T61I-HA Tg
Merge
DAPI
5 μm
H
Ant1/2
I
p-Nefl473
J
p-Syn129
K
Aggresome
L
Csnk1e/d
M
DA neuron in 35-week old Chchd2T61I-HA Tg mice
N
1 μm

Figure EV4.

Figure EV5. Analysis of dopaminergic neurons generated from patient-derived iPSCs.

A Schematic design of the guide RNA (gRNA) and the oligo donor for making isogenic control iPSC lines from patient-derived iPSCs. A mutation of thymine in both the edited allele and gRNA and the recovered cytosine in the oligo donor are shown in red. The two additional synonymous mutations that were used to introduce a *de novo* SacII site into the oligo donor are shown in green.

B Cultured DA neurons from iPSCs were observed by differential interference contrast microscopy. DA neurons from non-patient iPSCs (Control) and gene-corrected CHCHD2^{T61I} iPSCs (T61I-WT) looked healthy with neurite elongation, whereas those from CHCHD2^{T61I} iPSCs (T61I) were shrunken and without neurite elongation.

C DA neurons of each type were fixed and stained with anti-CHCHD2 and anti-TH antibodies. Dashed lines indicate cell shapes, and arrowheads indicate abnormal CHCHD2^{T61I} puncta.

D Quantification of cells displaying mitochondrial CHCHD2 puncta and extra-mitochondrial CHCHD2 puncta ($n \geq 100$ cells in each experiment). Data are shown as the mean ± SD ($n = 3$).

E–G Quantitative data of Fig 8C–E ($n = 30$ cells in each experiment). Red bars indicate mean values.

H–J Quantitative data of Fig 8F–H ($n = 30$ cells in each experiment). Red bars indicate mean values.

K, L PF-670462 has no effect on the extra-mitochondrial localization of CHCHD2^{T61I}. DA neurons from CHCHD2^{T61I} iPSCs were treated with PF-670462 for 20 h. Then, cells were fixed and stained with an anti-CHCHD2 and anti-ANT1/2 antibodies. In (K), representative images are shown. Dashed lines indicate cell shapes, and arrowheads indicate extra-mitochondrial CHCHD2^{T61I} puncta. (L) Quantification of cells displaying mitochondrial CHCHD2 and extra-mitochondrial CHCHD2 puncta ($n \geq 100$ cells in each experiment). Data are shown as the mean ± SD ($n = 3$). Comparisons were performed using one-way ANOVA followed by the Tukey *post hoc* test (D–G) or unpaired two-tailed Student *t*-test (H–J, L). $^{*}P < 0.05$; $^{**}P < 0.01$; NS: not significant.

 © 2023 The Authors

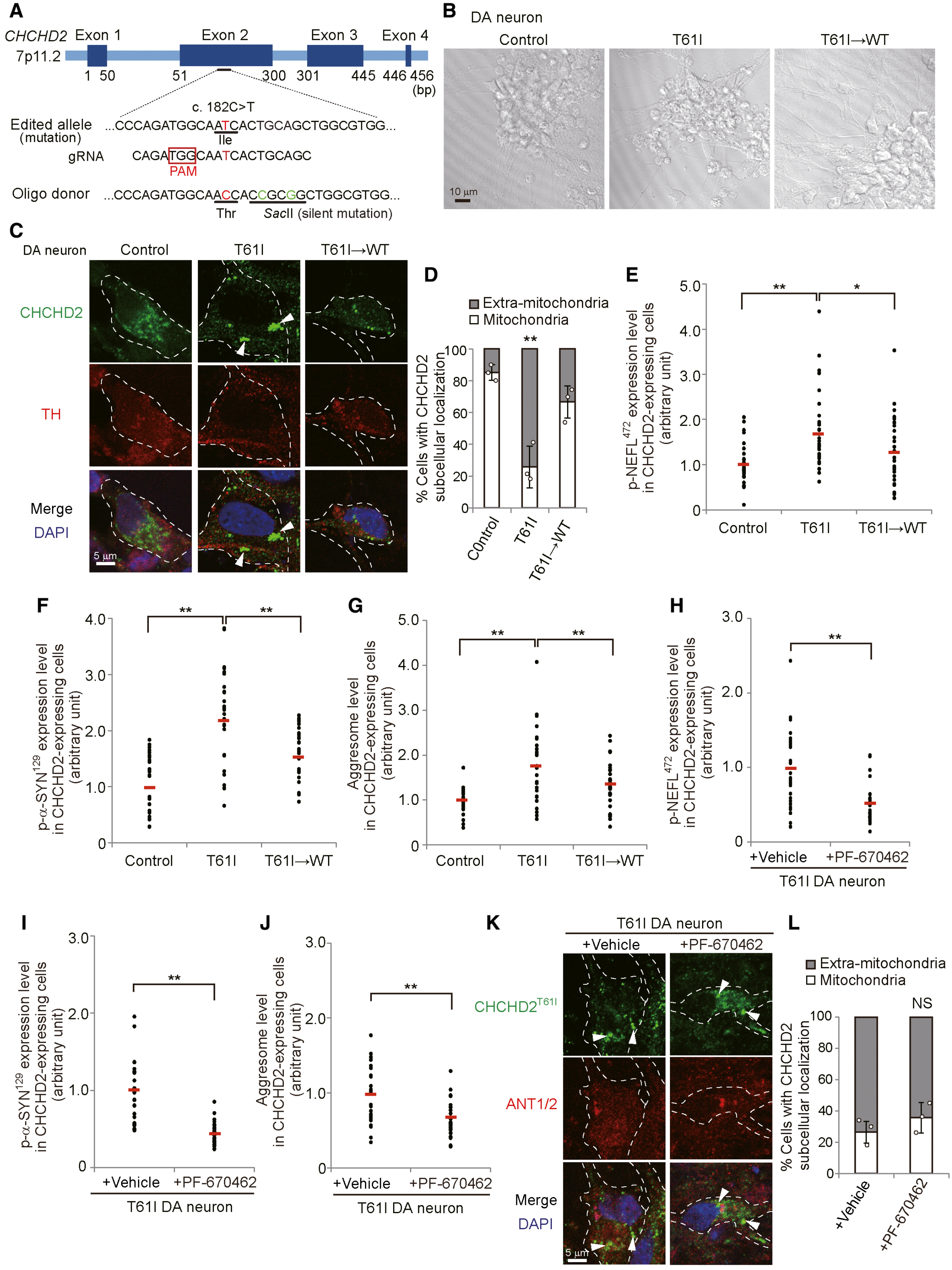
A
CHCHD2 Exon 1 Exon 2 Exon 3 Exon 4
7p11.2
1 50 51 300 301 445 446 456 (bp)
c. 182C>T
Edited allele (mutation) ...CCCAGATGGCAATCACTGCAGCTGGCGTGG...
Ile
gRNA CAGATGGCAATCACTGCAGC
PAM
Oligo donor ...CCCAGATGGCAACCACCGCGGCTGGCGTGG...
Thr SacII (silent mutation)
B
DA neuron
Control T61I T61I→WT
10 µm
C
DA neuron Control T61I T61I→WT
CHCHD2
TH
Merge
DAPI
5 µm
D
Extra-mitochondria
Mitochondria
% Cells with CHCHD2 subcellular localization
COntrol T61I T61I→WT
E
p-NEFL472 expression level in CHCHD2-expressing cells (arbitrary unit)
Control T61I T61I→WT
F
p-α-SYN129 expression level in CHCHD2-expressing cells (arbitrary unit)
Control T61I T61I→WT
G
Aggresome level in CHCHD2-expressing cells (arbitrary unit)
Control T61I T61I→WT
H
p-NEFL472 expression level in CHCHD2-expressing cells (arbitrary unit)
+Vehicle +PF-670462
T61I DA neuron
I
p-α-SYN129 expression level in CHCHD2-expressing cells (arbitrary unit)
+Vehicle +PF-670462
T61I DA neuron
J
Aggresome level in CHCHD2-expressing cells (arbitrary unit)
+Vehicle +PF-670462
T61I DA neuron
K
T61I DA neuron
+Vehicle +PF-670462
CHCHD2T61I
ANT1/2
Merge
DAPI
5 µm
L
Extra-mitochondria
Mitochondria
NS
% Cells with CHCHD2 subcellular localization
+Vehicle +PF-670462

Figure EV5.

Torii et al.

Involvement of casein kinase 1 epsilon/delta (Csnk1e/d) in the pathogenesis of familial Parkinson’ s disease caused by CHCHD2 gene mutations

Table of contents

Appendix Figure S1..2
Appendix Figure S2..4
Appendix Figure S3..6
Appendix Figure S4..8
Appendix Figure S5..10
Appendix Figure S6..12
Appendix Figure S7..13
Appendix Figure S8..15
Appendix Figure S9..16
Appendix Figure S10..18
Appendix Figure S11..19
Appendix Figure S12..21
Appendix Figure S13..22
Appendix Figure S14..24
Appendix Figure S15..25
Appendix Figure S16..27
Appendix Figure S17..28
Appendix Figure S18..30

Appendix Table S1...32
Appendix Table S2...34

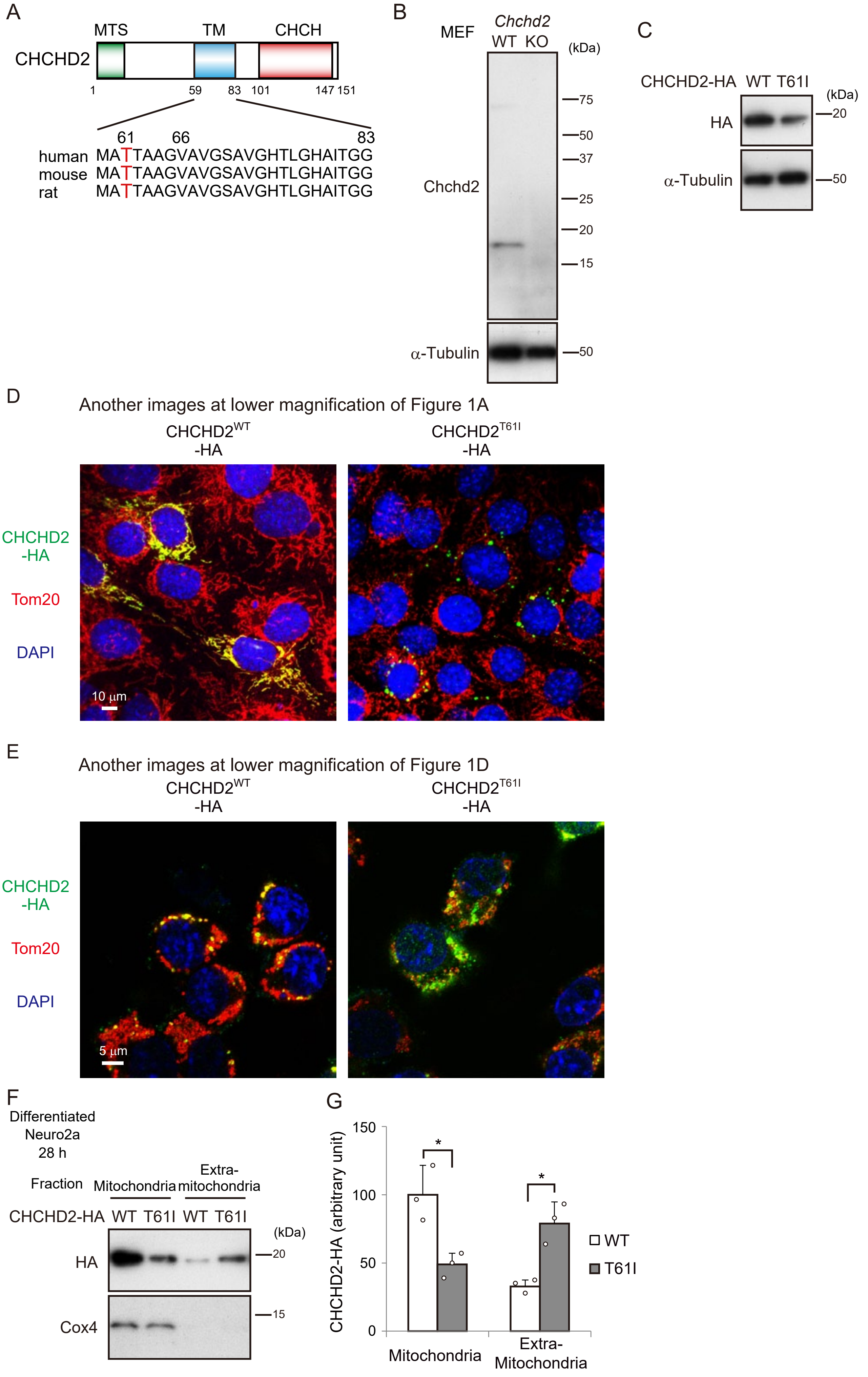
A
MTS
TM
CHCH
CHCHD2
1
59
83
101
147 151
61
66
83
human MATTAAGVAVGSAVGHTLGHAITGG
mouse MATTAAGVAVGSAVGHTLGHAITGG
rat MATTAAGVAVGSAVGHTLGHAITGG
B
MEF
Chchd2
WT KO
(kDa)
75
50
37
25
20
15
Chchd2
α-Tubulin
50
C
CHCHD2-HA WT T61I
(kDa)
HA
20
α-Tubulin
50
D
Another images at lower magnification of Figure 1A
CHCHD2WT -HA
CHCHD2T61I -HA
CHCHD2 -HA
Tom20
DAPI
10 μm
E
Another images at lower magnification of Figure 1D
CHCHD2WT -HA
CHCHD2T61I -HA
CHCHD2 -HA
Tom20
DAPI
5 μm
F
Differentiated Neuro2a 28 h
Fraction
Mitochondria
Extra-mitochondria
CHCHD2-HA WT T61I WT T61I
(kDa)
HA
20
Cox4
15
G
150
100
50
0
CHCHD2-HA (arbitrary unit)
*
*
WT
T61I
Mitochondria
Extra-Mitochondria

Appendix Fig. S1. Structure and expression of CHCHD2

(A) Structure of CHCHD2. MTS: mitochondrial targeting signal, TM: central α-helix (putative transmembrane domain), CHCH: coiled-coil-helix-coiled-coil-helix domain. Alignment of amino acid sequences of the TM of mammalian CHCHD2 (human, mouse, and rat). Thr^{61} is marked in red. **(B)** Lack of endogenous Chchd2 in *Chchd2*^{KO} MEFs. Cell lysates from *Chchd2*^{WT} and *Chchd2*^{KO} MEFs were subjected to western blotting to verify the expression of endogenous Chchd2. **(C)** Expression of Chchd2 and its mutant protein. *Chchd2*^{KO} cells were transfected with the *CHCHD2*^{WT}*-HA* and *CHCHD2*^{T61I}*-HA* plasmids. At 48 hr after transfection, cell lysates were subjected to western blotting. **(D)** Another representative images at lower magnification in Fig. 1A are shown. **(E)** Additional representative low-magnification images of the images in Fig. 1D are shown. **(F)** Neuro2a cells were transfected with the *CHCHD2*^{WT}*-HA* and *CHCHD2*^{T61I}*-HA* plasmids for 4 hr, and then cultured in medium containing 2% FBS and 10 μM retinoic acid. At 28 hr after transfection, cells were fractionated into mitochondria and non-mitochondria, and the expression of each protein was analyzed by western blotting. **(G)** A semiquantitative analysis of protein expression in **(F)** is shown. Data are shown as the mean ± SD ($n = 3$). Comparisons were performed using one-way ANOVA followed by the Tukey *post-hoc* test. *$p < 0.05$

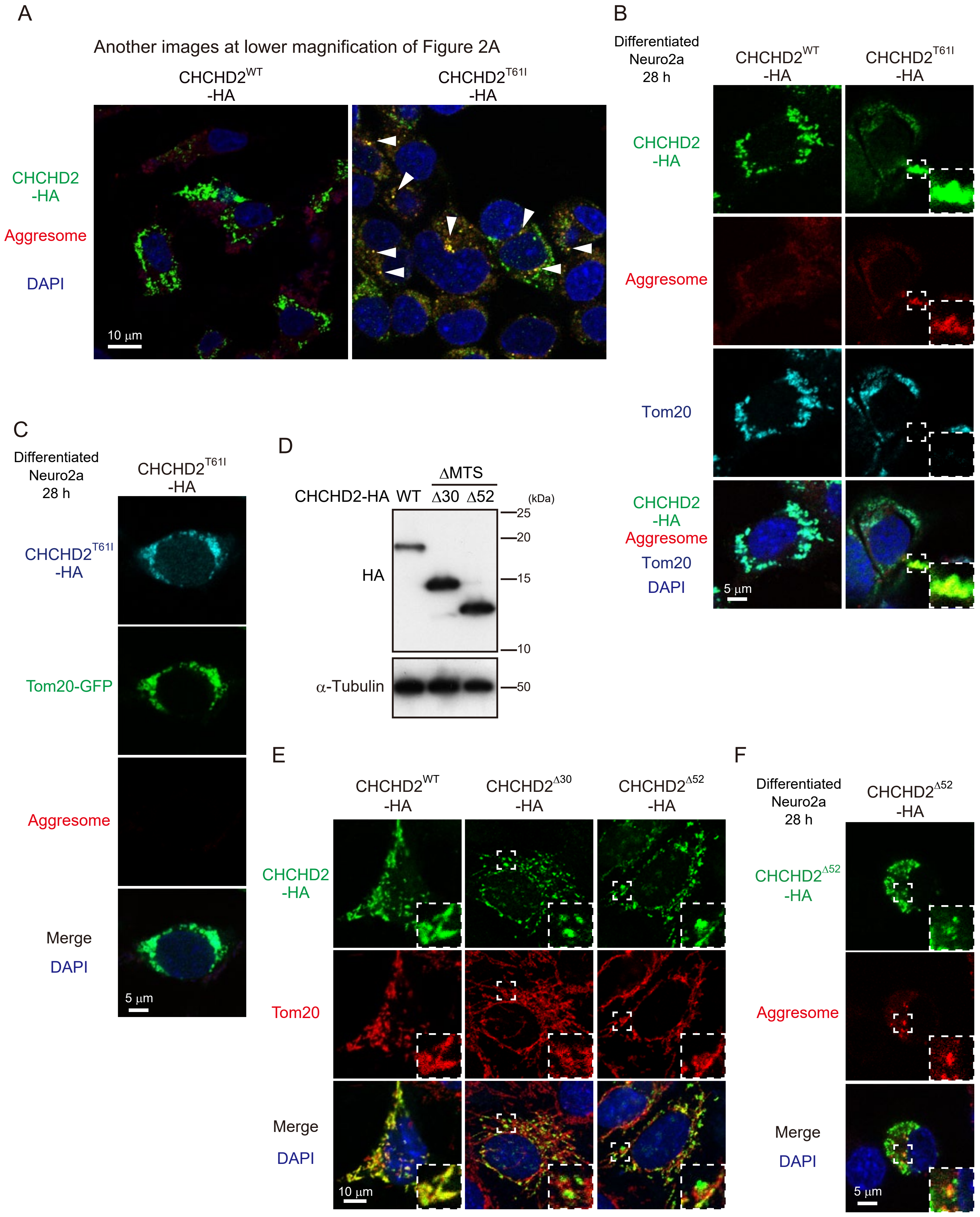
A
Another images at lower magnification of Figure 2A
CHCHD2WT -HA
CHCHD2T61I -HA
CHCHD2 -HA
Aggresome
DAPI
10 μm
B
Differentiated Neuro2a 28 h
CHCHD2WT -HA
CHCHD2T61I -HA
CHCHD2 -HA
Aggresome
Tom20
DAPI
5 μm
C
Differentiated Neuro2a 28 h
CHCHD2T61I -HA
Tom20-GFP
Aggresome
Merge
DAPI
5 μm
D
ΔMTS
CHCHD2-HA WT Δ30 Δ52
(kDa)
25
20
15
10
HA
α-Tubulin
50
E
CHCHD2WT -HA
CHCHD2Δ30 -HA
CHCHD2Δ52 -HA
CHCHD2 -HA
Tom20
Merge
DAPI
10 μm
F
Differentiated Neuro2a 28 h
CHCHD2Δ52 -HA
Aggresome
Merge
DAPI
5 μm

Appendix Fig. S2. Formation of aggresomes by extra-mitochondrial CHCHD2 mutant proteins

(A) Additional representative images at lower magnification of those in Fig. 2A are shown. Arrowheads indicate aggresomes containing CHCHD2^{T61I}-HA. **(B)** Neuro2a cells were transfected with *CHCHD^{IWT}-HA* and *CHCHD2^{T61I}-HA* plasmids for 4 hr, and then cultured in medium containing 2% FBS and 10 µM retinoic acid. At 28 hr after transfection, cells were stained with anti-HA and anti-Tom20 antibodies, and ProteoStat aggresome dye. Representative images are shown. The region of interest (ROI) indicates extramitochondrial aggresomes containing CHCHD2^{T61I}-HA, but not mitochondria (dashed squares). Magnified images of the areas are shown in the insets. **(C)** Neuro2a cells were transfected with *CHCHD2^{T61I}-HA* and *Tom20-GFP* plasmids for 4 hr, and then cultured in medium containing 2% FBS and 10 µM retinoic acid. At 28 hr after transfection, cells were stained with an anti-HA and anti-GFP antibodies, and ProteoStat aggresome dye. Representative images are shown. Intramitochondrial CHCHD2^{T61I}-HA did not generate aggresomes. **(D, E)** Extra-mitochondrial expression of CHCHD2$^{\Delta 30}$-HA and CHCHD2$^{\Delta 52}$-HA. The indicated CHCHD2 mutant proteins were expressed in *Chchd2^{KO}* cells by plasmid transfection. At 48 hr after transfection, cells were lysed and subjected to western blotting **(D)**, and were stained with anti-HA and anti-Tom20 antibodies and observed by confocal microscopy **(E)**. Magnified images of the areas within the dashed squares are shown in the insets. **(F)** Formation of aggresomes in Neuro2a cells expressing extra-mitochondrial CHCHD2$^{\Delta 52}$-HA. Neuro2a cells were transfected with the *CHCHD2^{T61I}-HA* plasmid for 4 hr, and then cultured in medium containing 2% FBS and 10 µM retinoic acid. At 28 hr after transfection, cells were stained with an anti-HA antibody and ProteoStat aggresome dye. Representative images are shown. Magnified images of the areas within the dashed squares are shown in the insets.

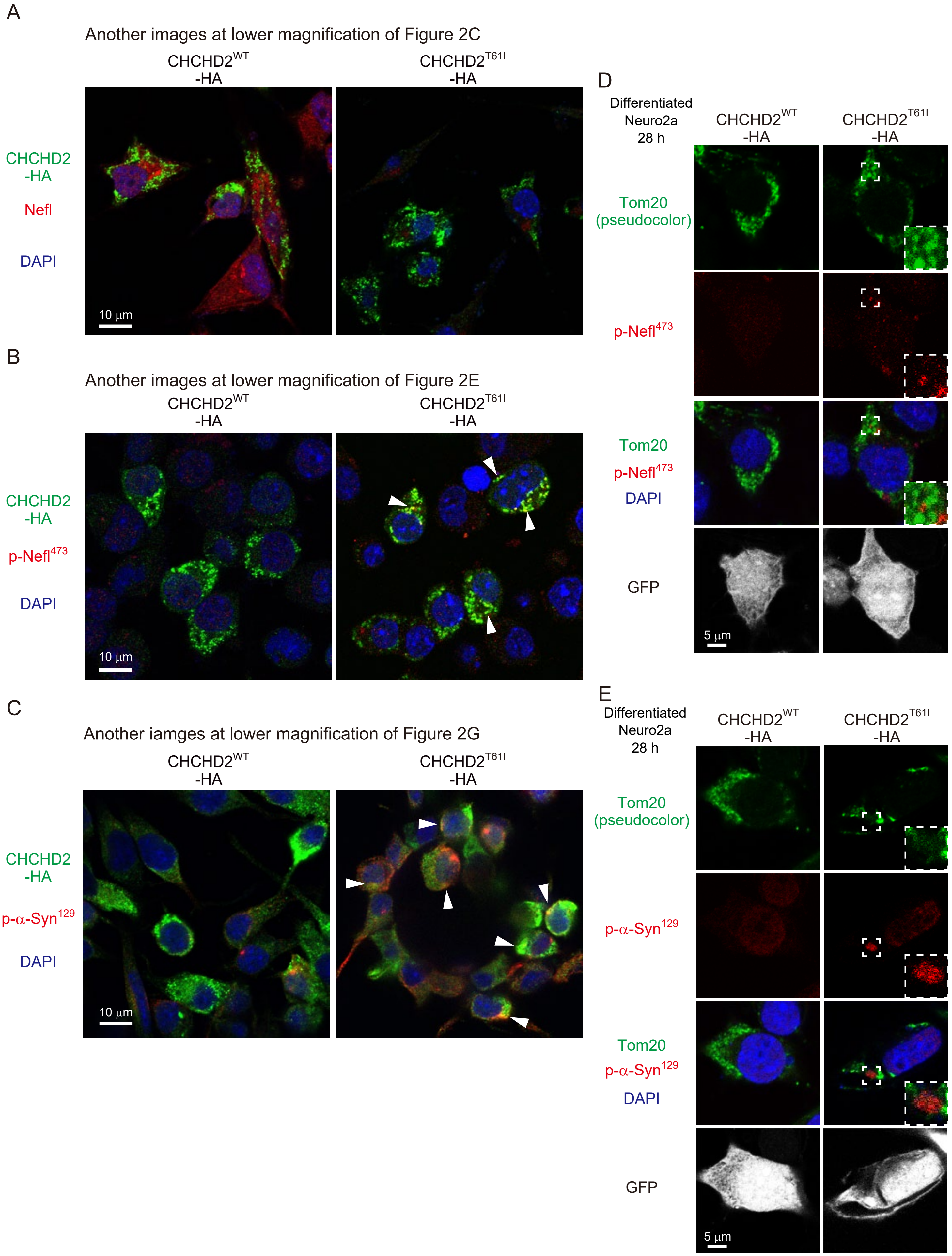
A
Another images at lower magnification of Figure 2C
CHCHD2^WT -HA
CHCHD2^T61I -HA
CHCHD2 -HA
Nefl
DAPI
10 µm
B
Another images at lower magnification of Figure 2E
CHCHD2^WT -HA
CHCHD2^T61I -HA
CHCHD2 -HA
p-Nefl^473
DAPI
10 µm
C
Another iamges at lower magnification of Figure 2G
CHCHD2^WT -HA
CHCHD2^T61I -HA
CHCHD2 -HA
p-α-Syn^129
DAPI
10 µm
D
Differentiated Neuro2a 28 h
CHCHD2^WT -HA
CHCHD2^T61I -HA
Tom20 (pseudocolor)
p-Nefl^473
Tom20
p-Nefl^473
DAPI
GFP
5 µm
E
Differentiated Neuro2a 28 h
CHCHD2^WT -HA
CHCHD2^T61I -HA
Tom20 (pseudocolor)
p-α-Syn^129
Tom20
p-α-Syn^129
DAPI
GFP
5 µm

Appendix Fig. S3. Generation of p-Nefl^{473} and p-α-Syn^{129} puncta in cells expressing extra-mitochondrial CHCHD2^{T61I}

(**A-C**) Additional representative lower magnification images of the images in Fig. 2C, E, and G are shown. **(D, E)** Neuro2a cells were transfected with *CHCHD^{WT}-HA*, *CHCHD2^{T61I}-HA*, and *pmax-GFP* plasmids for 4 hr, and then cultured in medium containing 2% FBS and 10 μM retinoic acid. At 28 hr after transfection, cells were stained with anti-Tom20 and anti-p-Nefl^{473} **(D)** or anti-p-α-Syn^{129} **(E)** antibodies. Extramitochondrial p-Nefl^{473} and p-α-Syn^{129} puncta (dashed squares) were observed only in *CHCHD2^{T61I}-HA* expressing cells. Magnified images of the areas are shown in the insets. GFP is a marker of successful transfection.

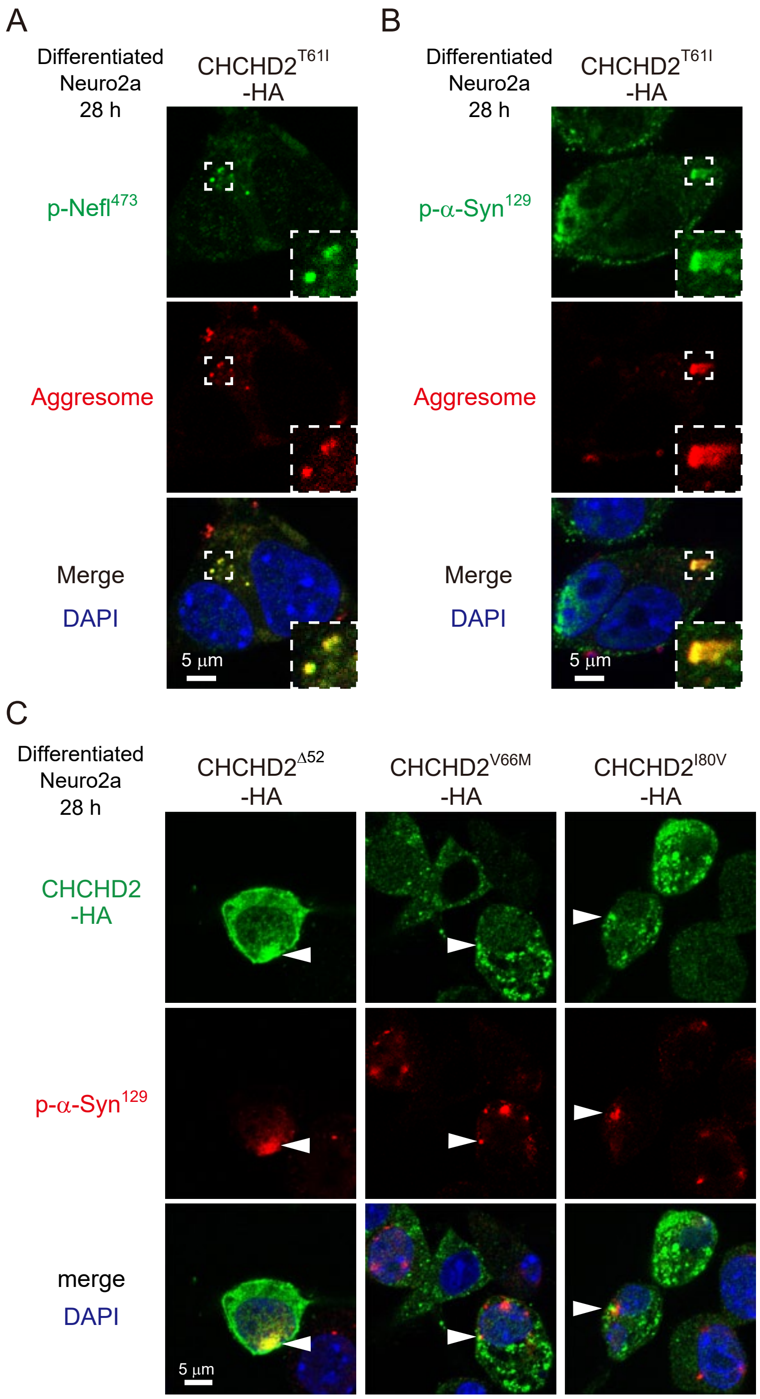
A
Differentiated Neuro2a 28 h
CHCHD2T61I -HA
p-Nefl473
Aggresome
Merge
DAPI
5 µm
B
Differentiated Neuro2a 28 h
CHCHD2T61I -HA
p-α-Syn129
Aggresome
Merge
DAPI
5 µm
C
Differentiated Neuro2a 28 h
CHCHD2Δ52 -HA
CHCHD2V66M -HA
CHCHD2I80V -HA
CHCHD2 -HA
p-α-Syn129
merge
DAPI
5 µm

Appendix Fig. S4. Generation of aggresome and p-α-Syn^{129} puncta in cells expressing extra-mitochondrial CHCHD2 mutant proteins

(A, B) Localization of p-Nefl^{473} and p-α-Syn^{129} in aggresomes formed in cells expressing CHCHD2^{T61I}-HA. Neuro2a cells were transfected with the *CHCHD2*^{T61I}*-HA* plasmid for 4 hr. At 28 hr after transfection, cells were stained with anti-p-Nefl^{473} **(A)** and anti-p-α-Syn^{129} **(B)** antibodies, and ProteoStat aggresome dye. Representative images are shown. Magnified images of the areas within the dashed squares are shown in the insets. **(C)** Neuro2a cells were transfected with the *CHCHD2*$^{\Delta 52}$*-HA*, *CHCHD2*^{V66M}*-HA*, and *CHCHD2*^{I80V}*-HA* plasmids for 4 hr, and then cultured in medium containing 2% FBS and 10 μM retinoic acid for neuronal differentiation. At 28 hr after transfection, cells were stained with anti-HA and anti-p-α-Syn^{129} antibodies. Arrowheads indicate colocalizing puncta.

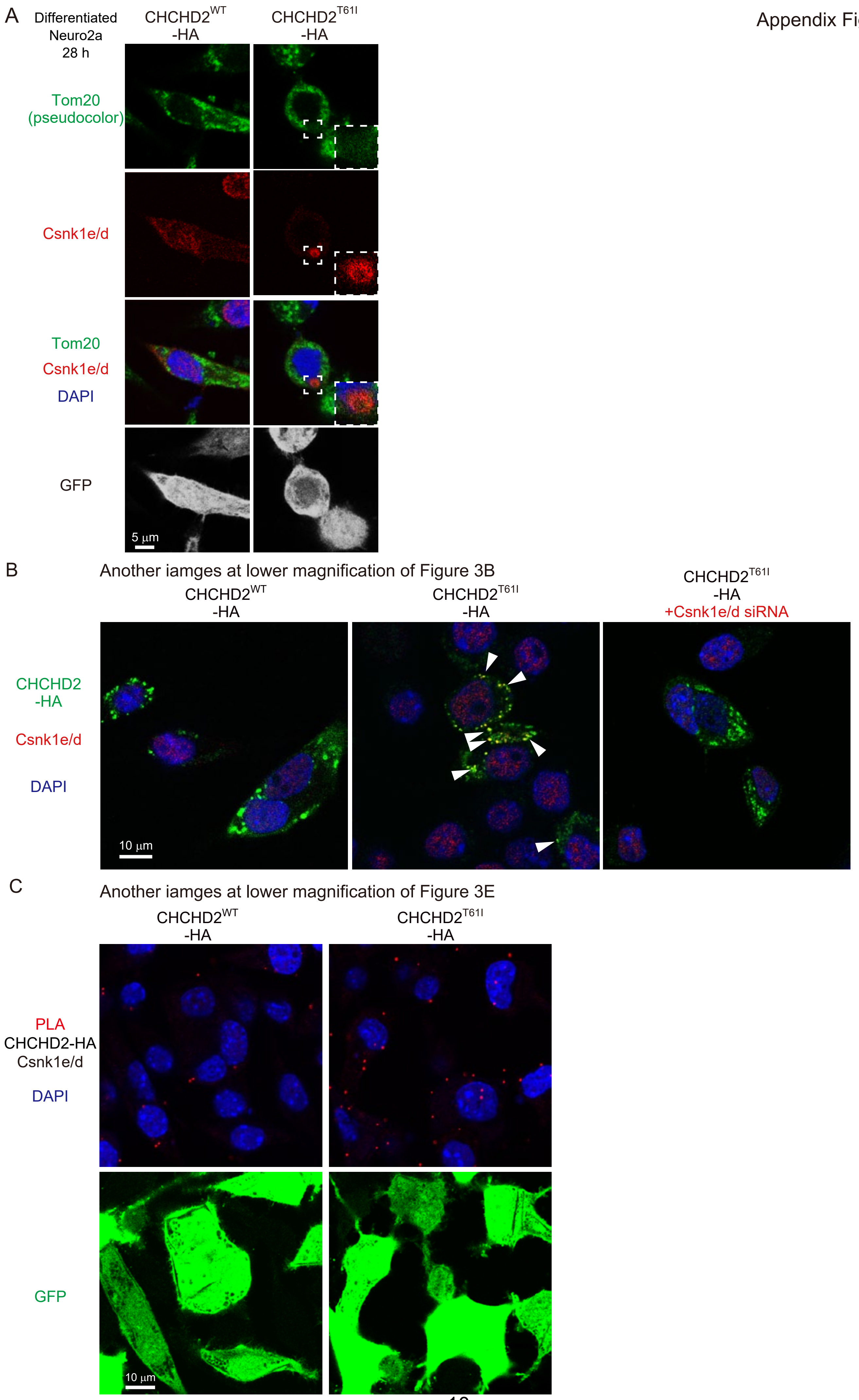
A
Differentiated Neuro2a 28 h
CHCHD2^{WT} -HA
CHCHD2^{T61I} -HA
Tom20 (pseudocolor)
Csnk1e/d
Tom20
Csnk1e/d
DAPI
GFP
5 µm
B
Another iamges at lower magnification of Figure 3B
CHCHD2^{WT} -HA
CHCHD2^{T61I} -HA
CHCHD2^{T61I} -HA
+Csnk1e/d siRNA
CHCHD2 -HA
Csnk1e/d
DAPI
10 µm
C
Another iamges at lower magnification of Figure 3E
CHCHD2^{WT} -HA
CHCHD2^{T61I} -HA
PLA
CHCHD2-HA
Csnk1e/d
DAPI
GFP
10 µm

Appendix Fig. S5 Interaction between CHCHD2^{T61I} and Csnk1e/d in outside of mitochondria

(A) Neuro2a cells were transfected with the *CHCHD2^{WT}-HA*, *CHCHD2^{T61I}-HA*, and *pmax-GFP* plasmids (to detect transfected cells) for 4 hr. At 28 hr after transfection, cells were stained with anti-Tom20 and anti-Csnk1e/d antibodies. Representative images are shown. Magnified images of the areas within the dashed squares are shown in the insets. **(B, C)** Additional representative lower magnification images of the images in Fig. 3B and E are shown. Arrows indicate colocalized puncta of CHCHD2^{T61I}-HA and Csnk1e/d.

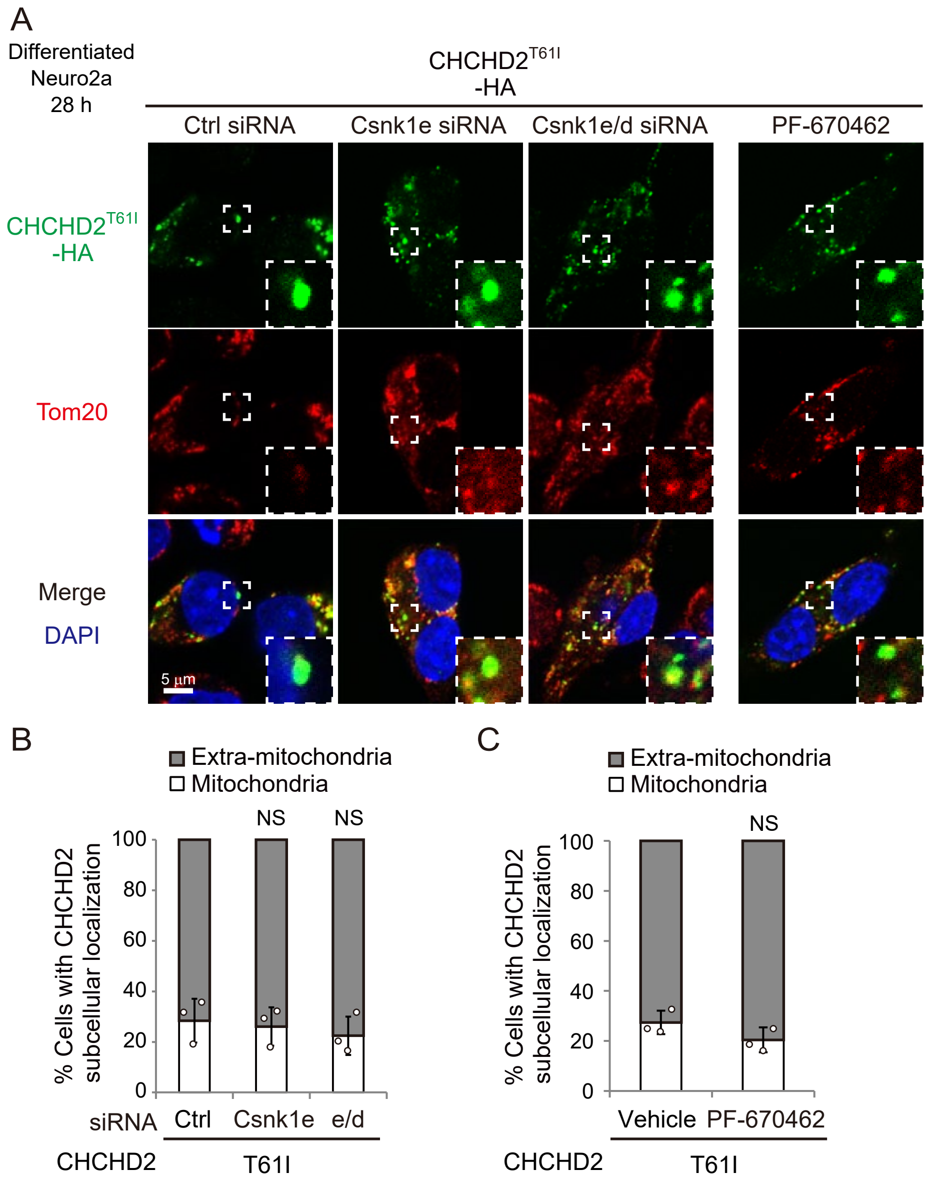

Appendix Fig. S6. No involvement of Csnk1e/d in the extra-mitochondrial localization of CHCHD2^{T61I}

Neuro2a cells were transfected with the *CHCHD2^{T61I}-HA* plasmid together with the indicated siRNAs, and were differentiated after 4 hr. At 28 hr after transfection, cells were fixed and stained with anti-HA and anti-Tom20 antibodies. Similar experiments were performed by the addition of PF-670462 (10 µM) instead of gene silencing. In **(A)**, representative images are shown. Magnified images of the areas within the dashed squares are shown in the insets. In **(B, C)**, the number of cells displaying mitochondrial CHCHD2^{T61I} and extra-mitochondrial CHCHD2^{T61I} puncta were quantified ($n \geq 100$ cells in each experiment). Data are shown as the mean ± SD ($n = 3$). Comparisons were performed using one-way ANOVA followed by the Tukey *post-hoc* test and the unpaired two-tailed Student *t*-test. NS: not significant

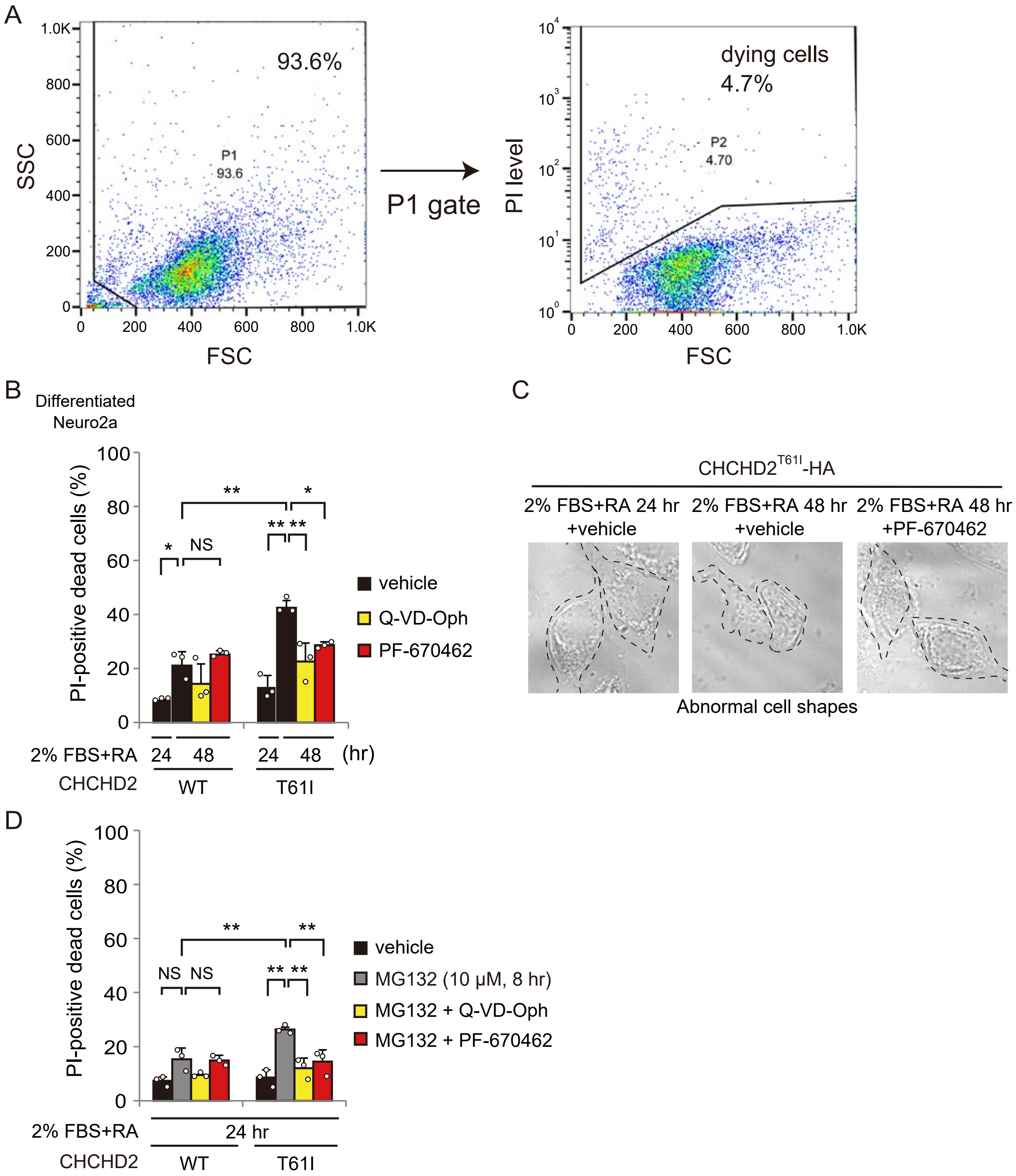
A
1.0K
800
600
400
200
0
SSC
P1
93.6
93.6%
0
200
400
600
800
1.0K
FSC
P1 gate
PI level
dying cells
4.7%
P2
4.70
FSC
B
Differentiated
Neuro2a
PI-positive dead cells (%)
vehicle
Q-VD-Oph
PF-670462
NS
2% FBS+RA
24
48
(hr)
CHCHD2
WT
T61I
C
CHCHD2T61I-HA
2% FBS+RA 24 hr
+vehicle
2% FBS+RA 48 hr
+vehicle
2% FBS+RA 48 hr
+PF-670462
Abnormal cell shapes
D
PI-positive dead cells (%)
vehicle
MG132 (10 µM, 8 hr)
MG132 + Q-VD-Oph
MG132 + PF-670462
NS
NS
2% FBS+RA
24 hr
CHCHD2
WT
T61I

Appendix Fig. S7. Induction of cell death in cells expressing CHCHD2^{T61I} and its inhibition by Q-VD-Oph and PF-670462

(A) Flow cytometry gating strategy. For the cell viability assay, cellular debris was removed using FSC/SSC (left panel). Then, PI-positive dead cells were detected using FSC/PE (PI level) (right panel). **(B, C)** Neuro2a cells stably expressing CHCHD2^{WT} or CHCHD2^{T61I} were seeded onto 12-well plates. After 12 hr, the culture medium was changed to medium containing 2% FBS and 10 µM retinoic acid for neuronal differentiation in the presence or absence of Q-VD-Oph (25 µM) or PF-670462 (10 µM). At the indicated times, cell death was analyzed by the PI uptake assay **(B)**. Cell morphology was also observed by differential interference contrast microscopy **(C)**. Dashed lines indicate cell shapes. **(D)** Neuro2a cells stably expressing CHCHD2^{WT} or CHCHD2^{T61I} were seeded onto 12-well plates, and the culture medium was changed to medium containing 2% FBS and 10 µM retinoic acid for neuronal differentiation. After 16 hr, MG132 (10 µM) was added together with or without Q-VD-Oph (25 µM) or PF-670462 (10 µM) for 8 hr, and cell death was analyzed by the PI uptake assay. Comparisons were performed using one-way ANOVA followed by the Tukey *post-hoc* test. NS: not significant; $*p < 0.05$; $**p < 0.01$

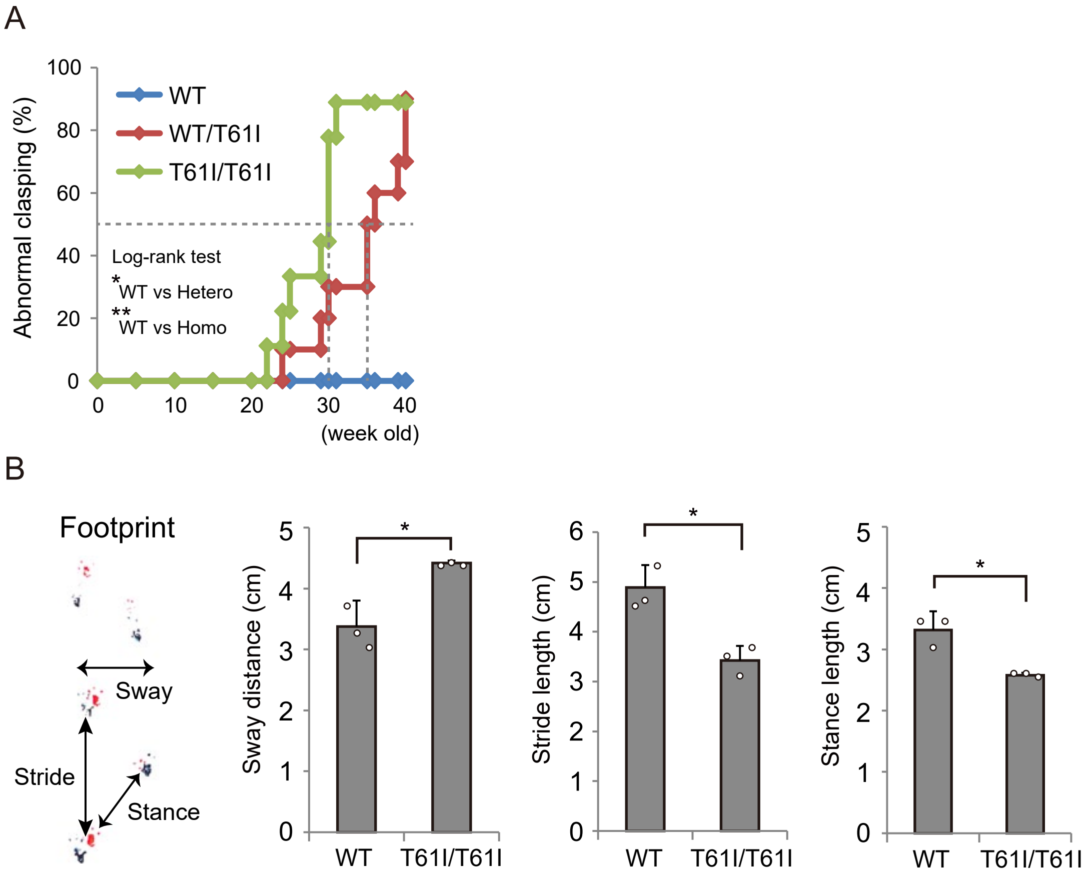

Appendix Fig. S8. Abnormal motor performance in Chchd2^{T61I} knock-in mice

(A) Quantitative analyses of the data in Fig. 5A are shown. Kaplan-Meier analysis of abnormal clasping of mice with the indicated genotypes. Comparisons were performed by the log-rank test (WT, n = 9; WT/T61I, n = 10; T61I/T61I, n = 9). **(B)** Sway, stride and stance length measured from the footprint analysis are shown with arrows (left). Quantitative analyses of the footprints are shown (right). Longer sway, shorter stride, and shorter stance indicate lower motor performance. Comparisons were performed using the unpaired two-tailed Student t-test. $*p < 0.05$; $**p < 0.01$

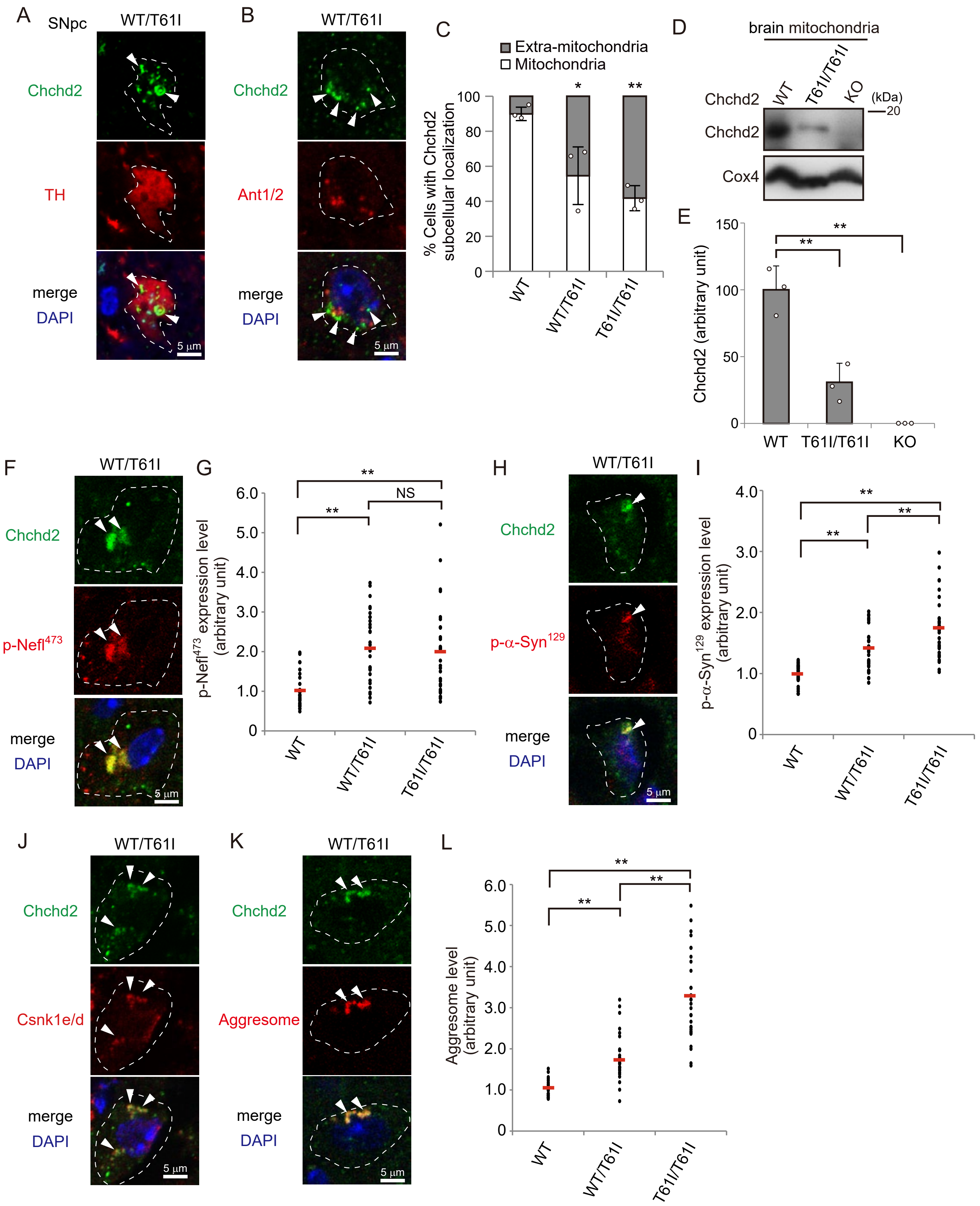
A
SNpc
WT/T61I
Chchd2
TH
merge
DAPI
5 µm
B
WT/T61I
Chchd2
Ant1/2
merge
DAPI
5 µm
C
Extra-mitochondria
Mitochondria
% Cells with Chchd2 subcellular localization
100
80
60
40
20
0
*
**
WT
WT/T61I
T61I/T61I
D
brain mitochondria
Chchd2
WT
T61I/T61I
KO
(kDa)
20
Chchd2
Cox4
E
Chchd2 (arbitrary unit)
150
100
50
0
**
**
WT
T61I/T61I
KO
F
WT/T61I
Chchd2
p-Nefl473
merge
DAPI
5 µm
G
p-Nefl473 expression level (arbitrary unit)
6.0
5.0
4.0
3.0
2.0
1.0
0
**
NS
**
WT
WT/T61I
T61I/T61I
H
WT/T61I
Chchd2
p-α-Syn129
merge
DAPI
5 µm
I
p-α-Syn129 expression level (arbitrary unit)
4.0
3.0
2.0
1.0
0
**
**
**
WT
WT/T61I
T61I/T61I
J
WT/T61I
Chchd2
Csnk1e/d
merge
DAPI
5 µm
K
WT/T61I
Chchd2
Aggresome
merge
DAPI
5 µm
L
Aggresome level (arbitrary unit)
6.0
5.0
4.0
3.0
2.0
1.0
0
**
**
**
WT
WT/T61I
T61I/T61I

Appendix Fig. S9. Generation of p-Nefl^{473} and p-α-Syn^{129} puncta, and aggresomes in Chchd2^{T61I} knock-in mice

(A–C) Cryosections of the midbrain of the indicated Chchd2^{T61I} knock-in mice were immunostained using antibodies for the indicated proteins. Dashed lines indicate cell shapes. Arrowheads indicate Chchd2 puncta in a TH-positive DA **(A)** and an extra-mitochondrial Chchd2$^{WT/T61I}$ punctum **(B)**. **(C)** Population of cells displaying mitochondrial CHCHD2 puncta and extra-mitochondrial CHCHD2 puncta in the SNpc of each mouse ($n \geq 100$ cells in each experiment). Data are shown as the mean ± SD ($n = 3$). **(D, E)** Isolated mitochondrial lysates from the whole brain of WT, *Chchd2$^{T61I/T61I}$*, and *Chchd2^{KO}* mice were subjected to western blotting. **(E)** A semiquantitative analysis of the protein expression in **(D)** is shown. Data are shown as the mean ± SD ($n = 3$). **(F-L)** The levels of p-Nefl^{473} **(F, G)**, p-α-Syn^{129} **(H, I)**, Csnk1e/d **(J)** puncta, and aggresomes **(K, L)** were analyzed by the average fluorescence intensity per cell ($n = 30$ cells in each experiment). Representative images are shown in **(F, H, J, K)**. Arrowheads indicate CHCHD2 puncta colocalizing with p-Nefl^{473} **(F)**, p-α-Syn^{129} **(H)**, Csnk1e/d **(J)**, and aggresomes **(K)**. In **(G, I, L)**, red bars indicate mean values. Comparisons were performed using one-way ANOVA followed by the Tukey post-hoc test. NS: not significant, $*p < 0.05$; $**p < 0.01$

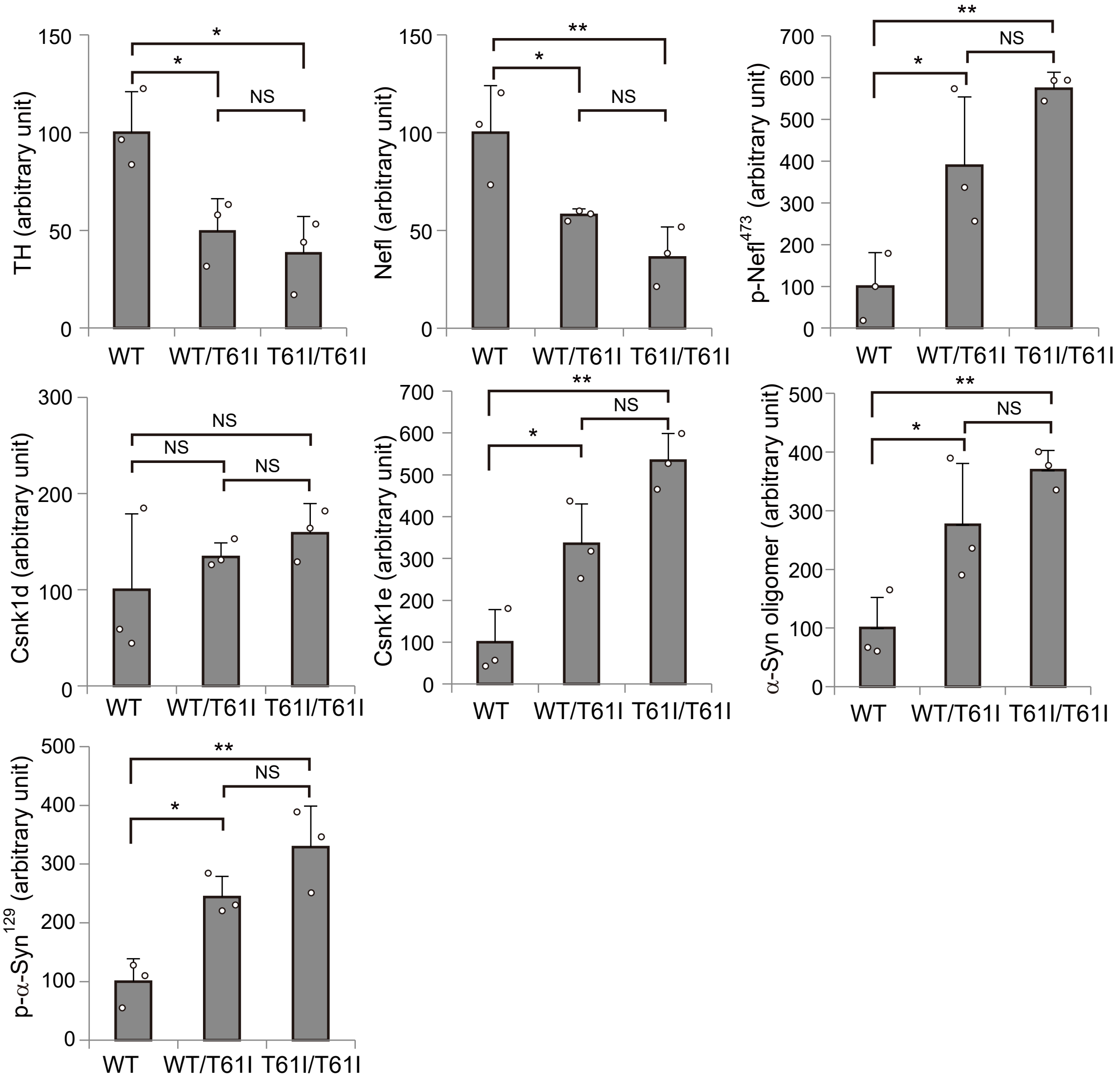

Appendix Fig. S10. Semiquantitative analyses of protein expression in the isolated midbrain and diencephalon

Expression levels of the indicated proteins from Fig. 5L are shown as the mean ± SD ($n = 3$). Comparisons were performed using one-way ANOVA followed by the Tukey post-hoc test. NS: not significant, *$p < 0.05$; **$p < 0.01$

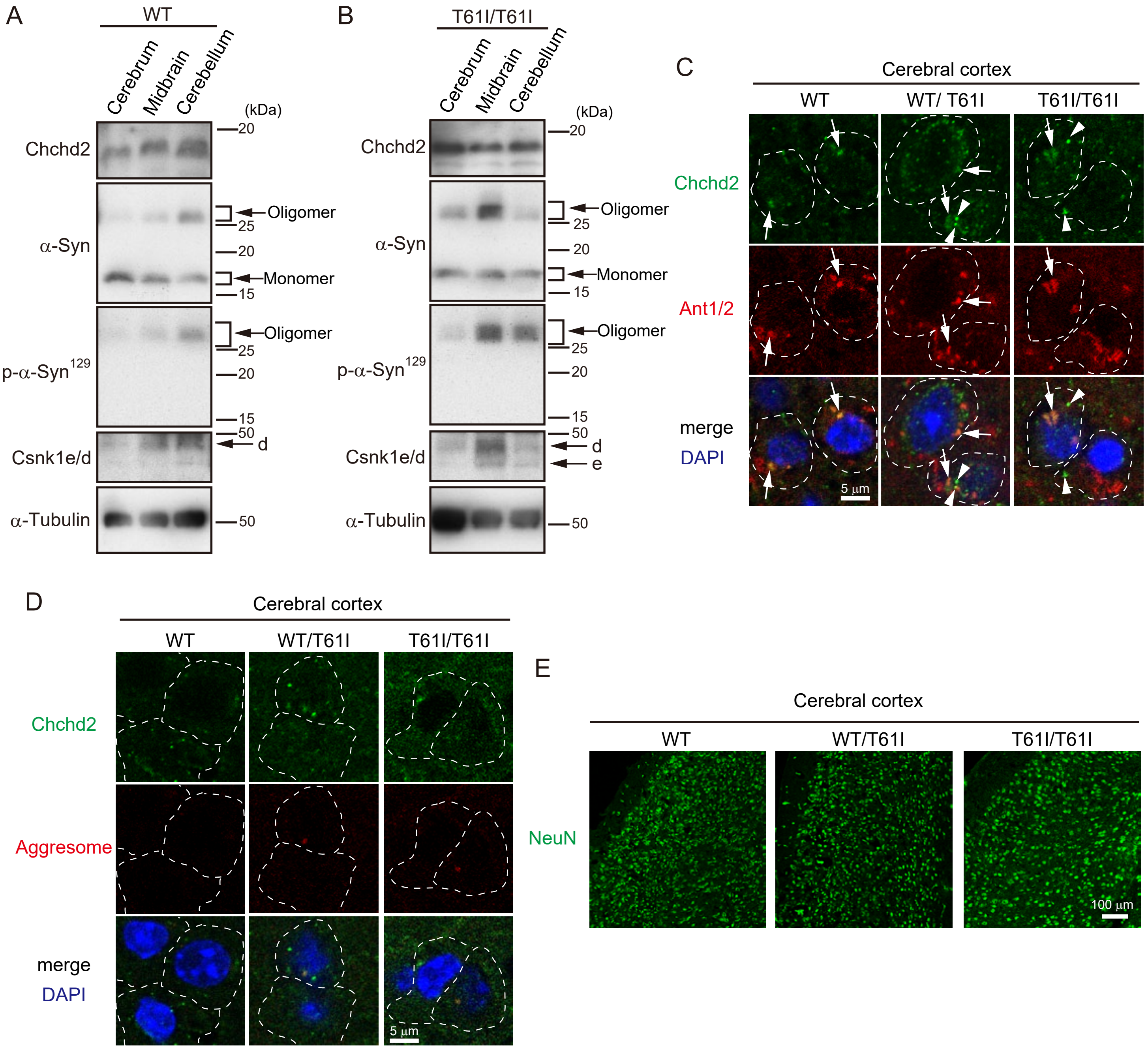
A
WT
Cerebrum
Midbrain
Cerebellum
(kDa)
Chchd2
α-Syn
p-α-Syn129
Csnk1e/d
α-Tubulin
Oligomer
Monomer
d
20
25
15
50
B
T61I/T61I
Cerebrum
Midbrain
Cerebellum
(kDa)
Chchd2
α-Syn
p-α-Syn129
Csnk1e/d
α-Tubulin
Oligomer
Monomer
d
e
C
Cerebral cortex
WT
WT/ T61I
T61I/T61I
Chchd2
Ant1/2
merge
DAPI
5 µm
D
Cerebral cortex
WT
WT/T61I
T61I/T61I
Chchd2
Aggresome
merge
DAPI
5 µm
E
Cerebral cortex
WT
WT/T61I
T61I/T61I
NeuN
100 µm

Appendix Fig. S11. Low level of p-α-Syn^{129} and absence of aggresome formation in the cerebral cortex and cerebellum of *CHCHD2^{T61I}* knock-in mice

(A, B) Lysates of the indicated brain regions from WT mice **(A)** and CHCHD2$^{T61I/T61I}$ knock-in mice **(B)** were prepared and subjected to western blotting. **(C)** Cryosections of the cerebral cortex of each mouse were immunostained with anti-Chchd2 and anti-Ant1/2 antibodies. Dashed lines indicate cell shape. Arrows and arrowheads indicate mitochondrial Chchd2 puncta and extra-mitochondrial Chchd2 puncta, respectively. **(D)** Cryosections of the cerebral cortex were immunostained with an anti-Chchd2 antibody and ProteoStat aggresome dye. Dashed lines indicate cell shapes. Aggresomes were not observed in these cells. **(E)** Cryosections of the cerebral cortex were immunostained with an anti-NeuN antibody to detect neuronal injury. No clear neuronal injury was detected in CHCHD2$^{T61I/T61I}$ and CHCHD2$^{WT/T61I}$ knock-in mice.

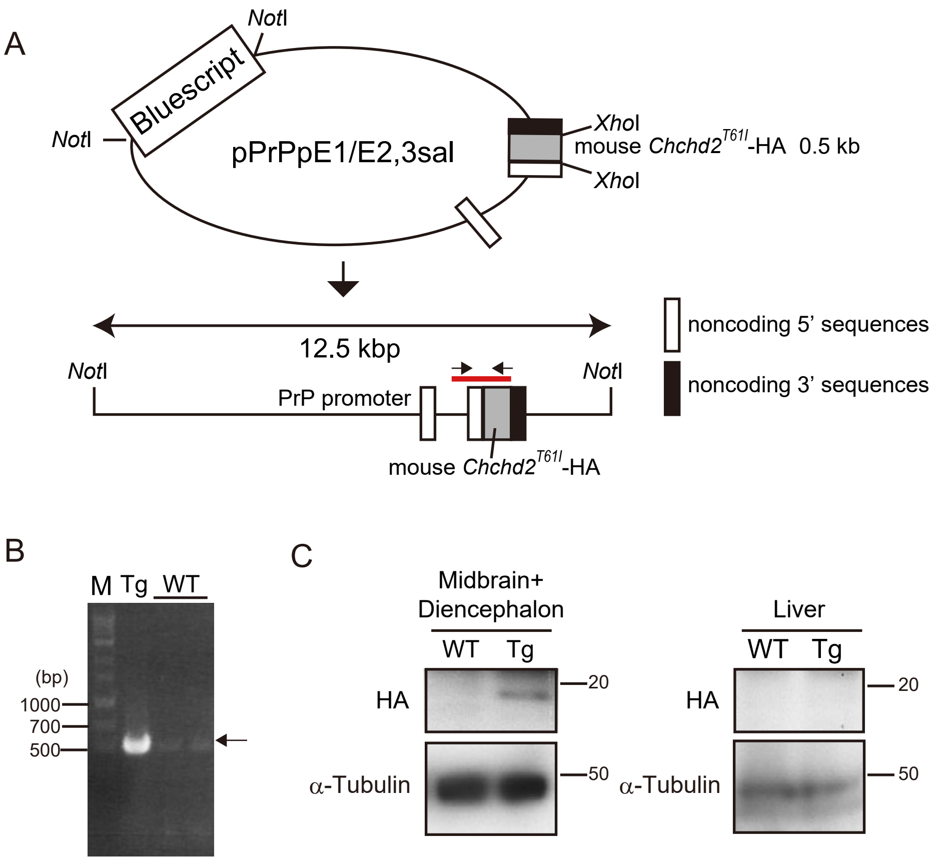

Appendix Fig. S12. Generation of Chchd2^{T61I}-HA Tg mice

(A) Schematic representation of the vector used to generate Chchcd2^{T61I}-HA Tg mice. The *Chchd2* ^{T61I}-HA fragment was inserted into the *Xho*I site in the pPrPpE1/E2,3sal vector. The pBluescript vector sequence was removed by digestion with *Not*I, resulting in a 12.5-kbp fragment (bottom). Small arrows indicate the position of the primers used for genotyping PCR and the red line indicates the PCR product (about 600 bp). **(B)** Genotyping was performed by genome amplification by PCR [product about 600 bp, a red line in **(A)**] from tail genomic DNA. The primers used are listed in Appendix Table S1. M indicates the lane of DNA markers. **(C)** Lysates from the indicated tissues were obtained from Chchcd2^{T61I}-HA Tg mice and their littermate WT mice, and were subjected to western blotting. Chchcd2^{T61I}-HA was expressed in the midbrain and diencephalon, but not in the liver.

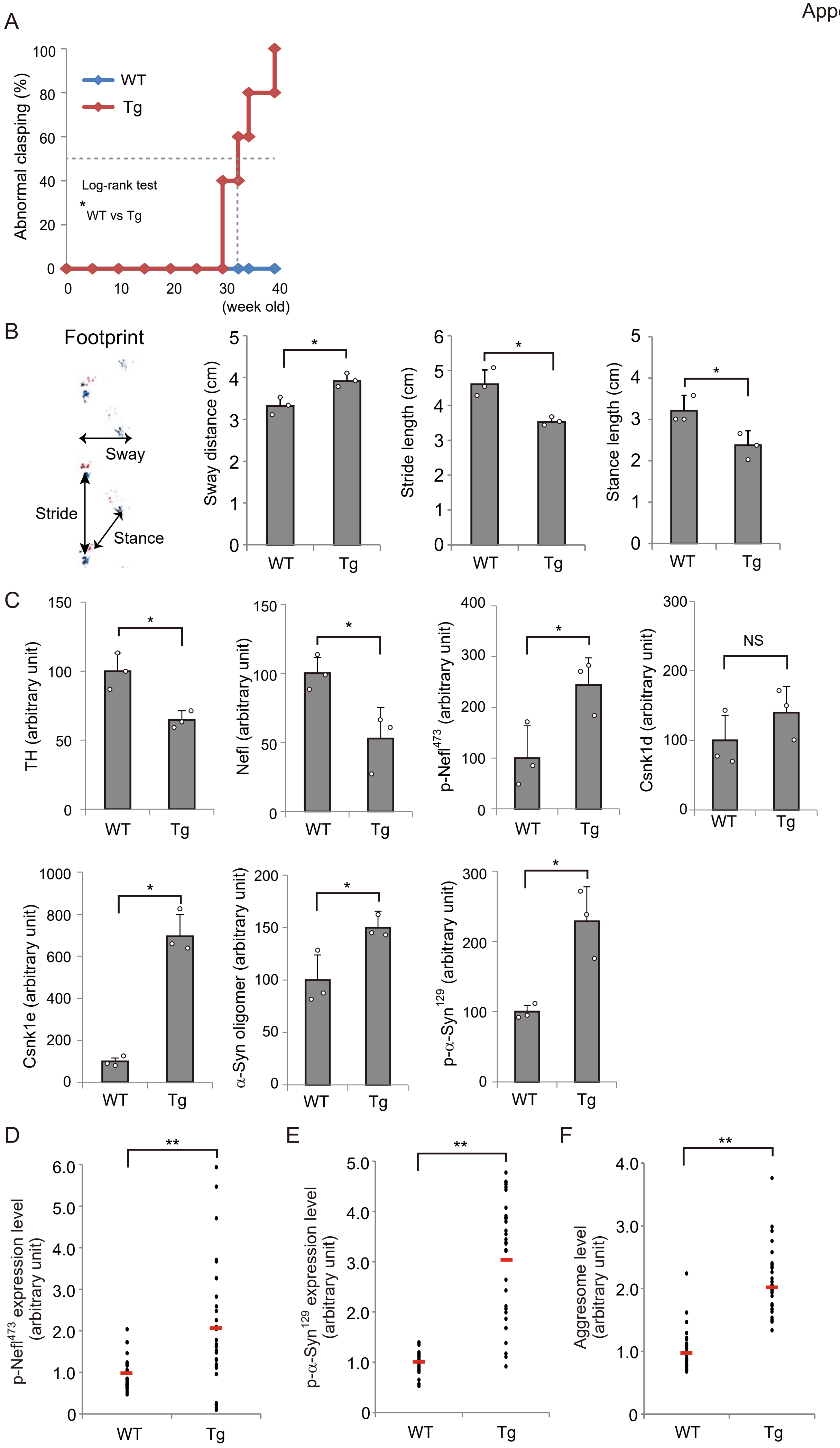
A
Abnormal clasping (%)
WT
Tg
Log-rank test
* WT vs Tg
(week old)
B
Footprint
Sway
Stride
Stance
Sway distance (cm)
Stride length (cm)
Stance length (cm)
WT
Tg
C
TH (arbitrary unit)
Nefl (arbitrary unit)
p-Nefl473 (arbitrary unit)
Csnk1d (arbitrary unit)
NS
Csnk1e (arbitrary unit)
α-Syn oligomer (arbitrary unit)
p-α-Syn129 (arbitrary unit)
D
p-Nefl473 expression level (arbitrary unit)
E
p-α-Syn129 expression level (arbitrary unit)
F
Aggresome level (arbitrary unit)

Appendix Fig. S13. Reduced motor performance and upregulation of p-Nefl^{473}, p-α-Syn^{129}, and aggresomes in the SNpc of Chchd2^{T61I}-HA Tg mice

(A) Quantitative analyses of the data in Fig. EV4A are shown. Kaplan-Meier analysis of the abnormal clasping of mice with the indicated genotypes. Comparisons were performed by the log-rank test (WT, n = 5; Chchd2^{T61I}-HA Tg, n = 5). **(B)** Sway, stride and stance length measured from footprint analysis are shown with arrows (left). Quantitative analyses of the footprints in Fig. EV4C are shown (right). Longer sway, shorter stride, and shorter stance indicate lower motor performance. **(C)** Semiquantitative analyses of protein expression in Fig. EV4B. **(D-F)** The amount of p-Nefl^{473} **(D)**, p-α-Syn^{129} **(E)**, and aggresomes **(F)** in the SNpc of Chchd2^{T61I}-HA Tg mice was quantified by the average fluorescence intensity per cell (n = 30 cells in each experiment). Representative images are shown in Fig. EV4I–K. Red bars indicate mean values. In **(B-F)**, comparisons were performed by the unpaired two-tailed Student t-test. $*p < 0.05$; $**p < 0.01$. NS: not significant

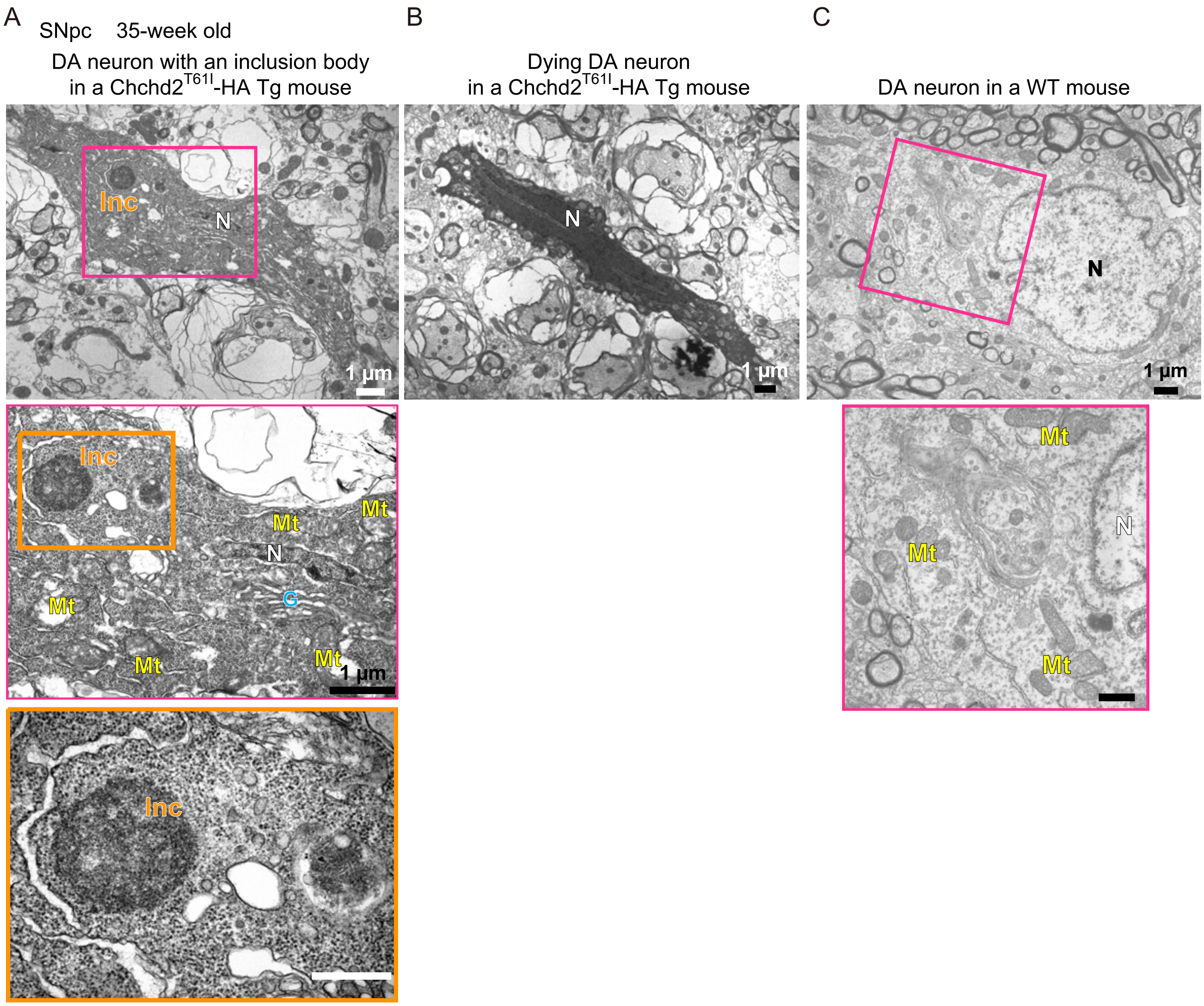

Appendix Fig. S14. Abnormal inclusion bodies and cell death in DA neurons of Chchd2^{T61I}-HA Tg mice observed by EM

(A, B) Sections of DA neurons in the SNpc of CHCHD2^{T61I}-HA Tg mice. **(A)** The DA neuron contained an inclusion body (Inc), and showed an abnormal nuclear shape (N), swollen mitochondria (Mt), and swollen Golgi (G). A magnified image of the area within the pink square of the top panel is shown in the middle panel, and that of the orange square in the middle panel is shown in the bottom panel. The inclusion body did not have a clear membrane. A low magnification image is shown in Fig. EV4M. **(B)** A dying DA neuron is shown. **(C)** A normal DA neuron in the SNpc of a WT mouse. A magnified image of the area within the pink square of the top panel is shown in the bottom panel.

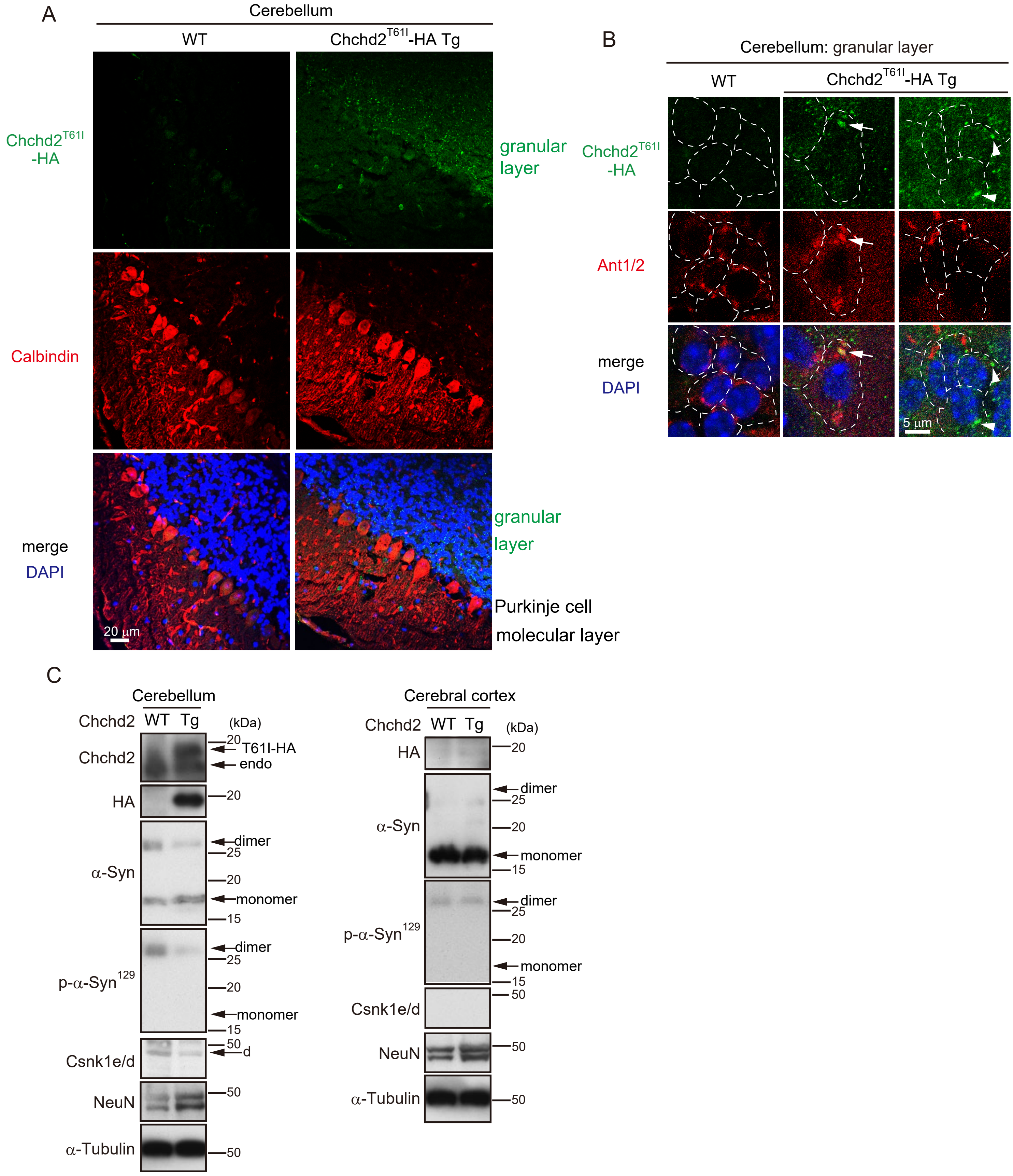
A
Cerebellum
WT
Chchd2T61I-HA Tg
Chchd2T61I
-HA
granular
layer
Calbindin
merge
DAPI
20 µm
granular
layer
Purkinje cell
molecular layer
B
Cerebellum: granular layer
WT
Chchd2T61I-HA Tg
Chchd2T61I
-HA
Ant1/2
merge
DAPI
5 µm
C
Cerebellum
Chchd2
WT
Tg
(kDa)
Chchd2
20
T61I-HA
endo
HA
20
α-Syn
dimer
25
20
monomer
15
p-α-Syn129
dimer
25
20
monomer
15
Csnk1e/d
50
d
NeuN
50
α-Tubulin
50
Cerebral cortex
Chchd2
WT
Tg
(kDa)
HA
20
α-Syn
dimer
25
20
monomer
15
p-α-Syn129
dimer
25
20
monomer
15
Csnk1e/d
50
NeuN
50
α-Tubulin
50

Appendix Fig. S15. No upregulation of p-α-Syn^{129} in the cerebellum and cerebral cortex of Chchd2^{T61I}-HA Tg mice

(A) Cryosections of the cerebellum of WT and Chchd2^{T61I}-HA Tg mice were immunostained with anti-HA and anti-calbindin antibodies. Chchd2^{T61I}-HA was expressed in the granular layer of the cerebellum. **(B)** Cryosections of the granular layer of the cerebellum in WT and Chchd2^{T61I}-HA Tg mice were immunostained with anti-HA and anti-Ant1/2 antibodies. Dashed lines indicate the cell shapes. Arrows and arrowheads indicate mitochondrial Chchd2^{T61I}-HA and extra-mitochondrial Chchd2^{T61I}-HA, respectively. **(C)** Lysates of the cerebellum (left) and cerebral cortex (right) were subjected to western blotting for the indicated proteins.

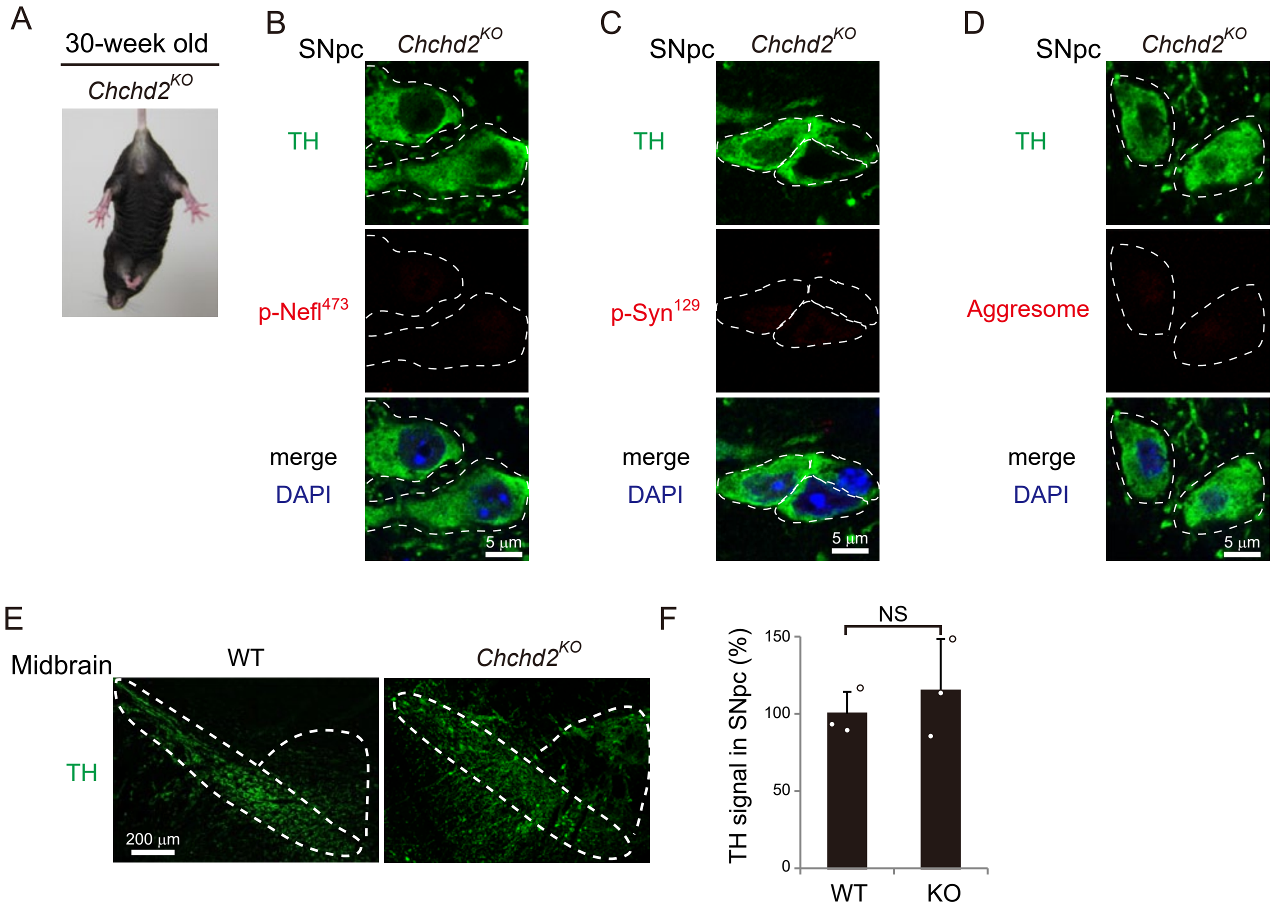

Appendix Fig. S16. Different neuropathology of dopaminergic neurons between *Chchd2*^{KO} mice and *CHCHD2*^{T61I} knock-in mice

(A) The limb-clasping reflex was not observed in *Chchd2*^{KO} mice at 30 weeks of age. **(B–D)** Cryosections of the SNpc in *Chchd2*^{KO} mice at 40 weeks of age were immunostained with anti-TH and anti-p-Nefl^{473} antibodies (**B**), the anti-p-α-Syn^{129} antibody **(C)**, and ProteoStat aggresome dye **(D)**. **(E, F)** Nine sections each from the brains of 40-weeks-old mice were immunostained for TH. In **(E)**, representative images of TH staining of the SNpc and VTA are shown. Dashed lines indicate the SNpc and VTA regions. In **(F)**, TH signals in the SNpc were counted. Data are shown as the mean ± SD (n = 3). Comparisons were performed using the unpaired two-tailed Student *t*-test. NS: not significant

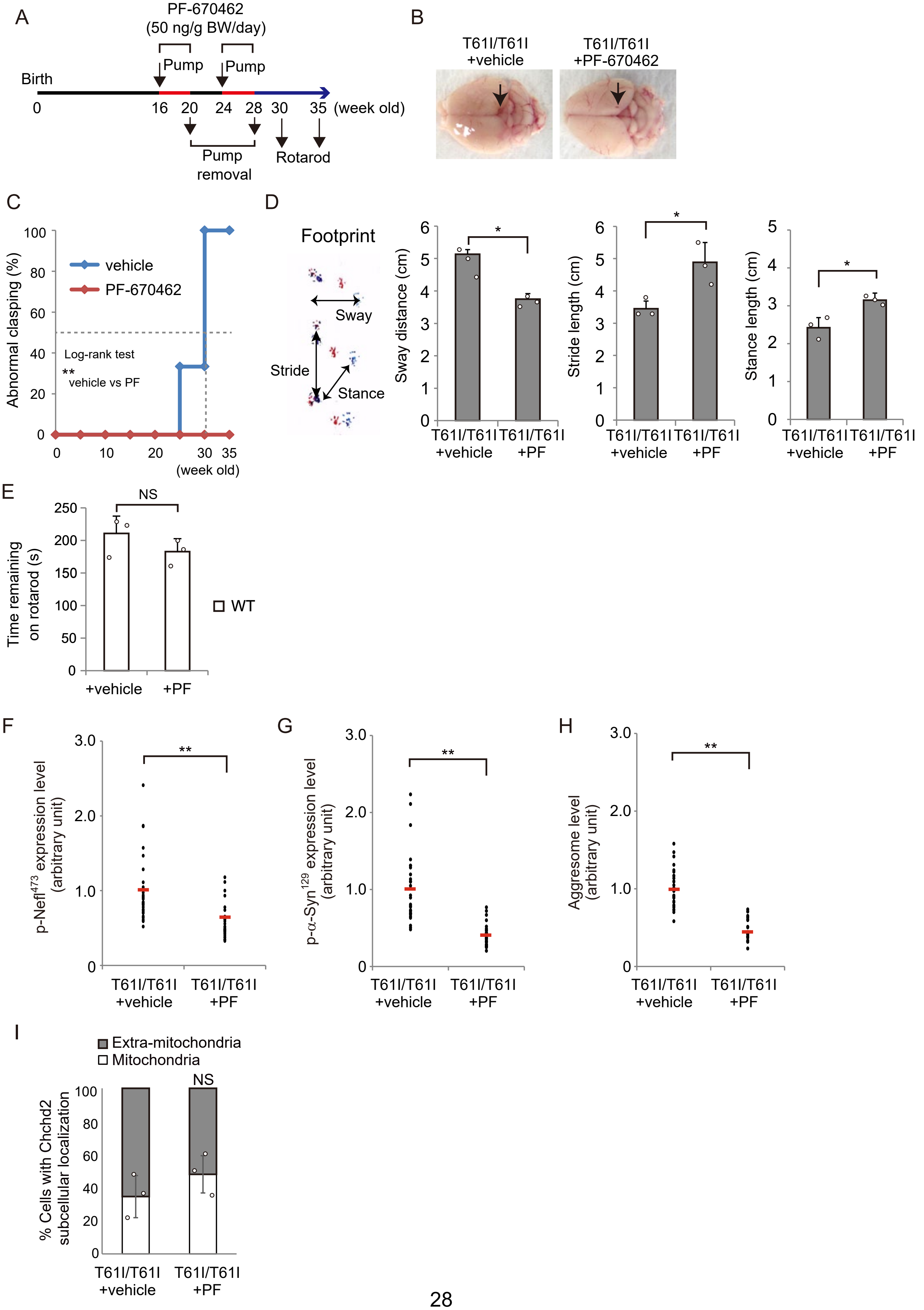
A
PF-670462
(50 ng/g BW/day)
Pump
Pump
Birth
0
16 20 24 28 30 35 (week old)
Pump
removal
Rotarod
B
T61I/T61I
+vehicle
T61I/T61I
+PF-670462
C
Abnormal clasping (%)
vehicle
PF-670462
Log-rank test
**
vehicle vs PF
(week old)
D
Footprint
Sway
Stride
Stance
Sway distance (cm)
Stride length (cm)
Stance length (cm)
T61I/T61I
+vehicle
T61I/T61I
+PF
E
NS
Time remaining
on rotarod (s)
WT
+vehicle
+PF
F
p-Nefl473 expression level
(arbitrary unit)
G
p-α-Syn129 expression level
(arbitrary unit)
H
Aggresome level
(arbitrary unit)
I
Extra-mitochondria
Mitochondria
NS
% Cells with Chchd2
subcellular localization

Appendix Fig. S17. Effects of PF-670462 on Chchd2$^{T61I/T61I}$ mice

(A) Schematic diagram of the time course of the osmotic pump experiment. PF-670462 or vehicle was continuously infused into the cerebral cortex just above the SNpc of Chchd2$^{T61I/T61I}$ mice from 16 weeks of age. Mice were analyzed at 30 and 35 weeks of age. **(B)** Representative brains after removal of the olfactory bulbs are shown. Arrows indicate the stereotaxic injection sites (see Material and Methods). **(C)** Quantitative analyses of the data in Fig. 6A are shown. Kaplan-Meier analysis of the abnormal clasping of mice with the indicated genotypes. Comparisons were performed by the log-rank test (vehicle, $n = 3$; PF-670462, $n = 4$). **(D)** Sway, stride, and stance length measured from the footprint analysis are indicated by arrows (left). Quantitative analyses of the footprint analysis data in Fig. 6B are shown (right). Longer sway, shorter stride, and shorter stance indicate lower motor performance. **(E)** No effect of PF-670462 on the motor performance of WT mice. PF-670462 (50 ng/g BW/day) or 5% DMSO was continuously infused into the cerebral cortex of WT mice from 16 weeks of age for 4 weeks. Then, the time that the indicated mice remained on the rotarod was measured. Data are shown as the mean ± SD ($n = 3$ mice). **(F-H)** Quantitative data of Fig. 6F-H ($n = 30$ cells in each experiment). Red bars indicate mean values. **(I)** Quantitative data of Fig. 6I. Population of cells displaying mitochondrial CHCHD2 puncta and extra-mitochondrial CHCHD2 puncta in the SNpc of each mouse ($n \geq 100$ cells in each experiment). Data are shown as the mean ± SD ($n = 3$). In **(D-I)**, comparisons were performed using the unpaired two-tailed Student *t*-tests. *$p < 0.05$; **$p < 0.01$, NS: not significant

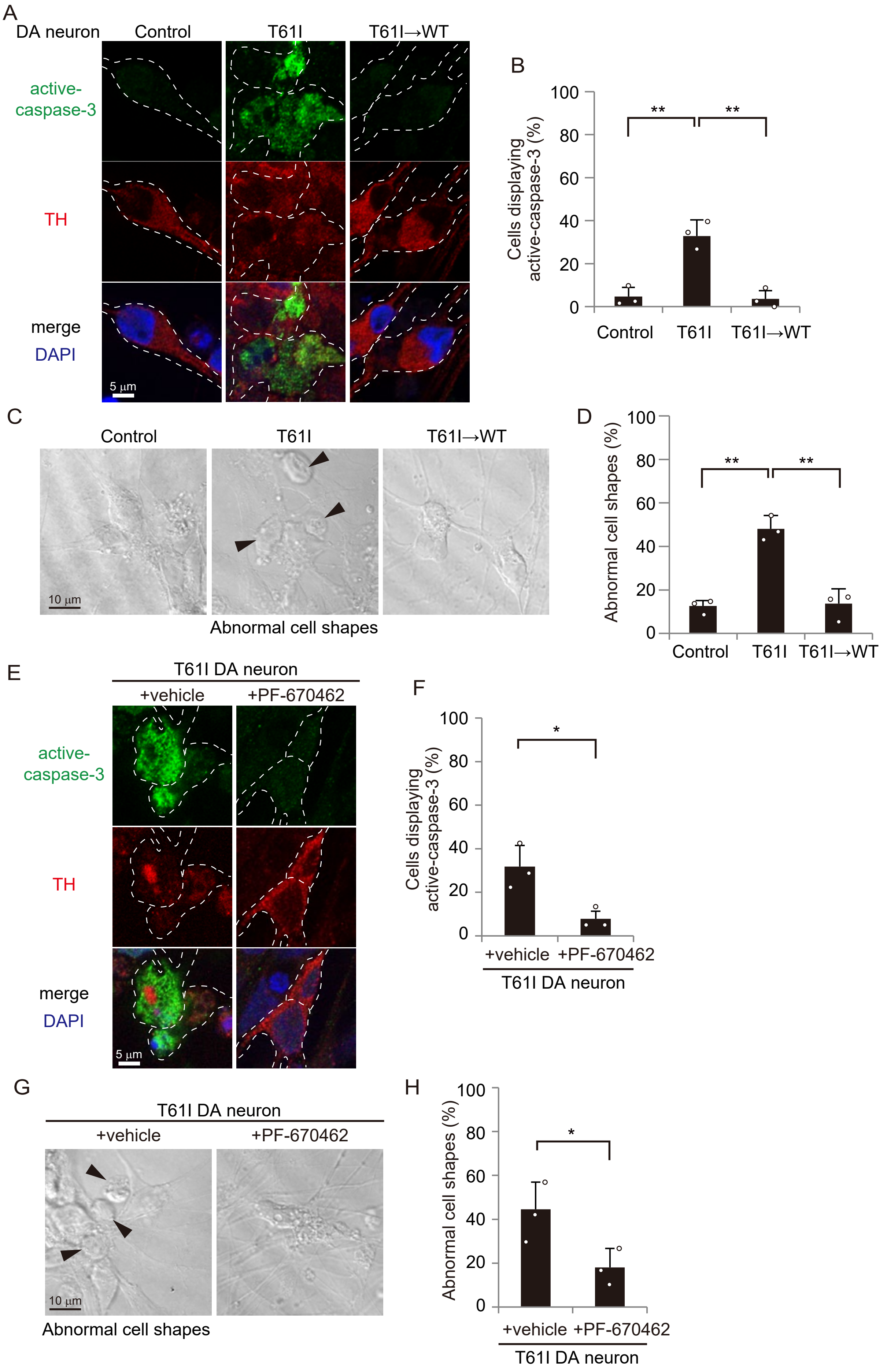
A
DA neuron
Control
T61I
T61I→WT
active-caspase-3
TH
merge
DAPI
5 µm
B
Cells displaying active-caspase-3 (%)
100
80
60
40
20
0
**
**
Control
T61I
T61I→WT
C
Control
T61I
T61I→WT
10 µm
Abnormal cell shapes
D
Abnormal cell shapes (%)
100
80
60
40
20
0
**
**
Control
T61I
T61I→WT
E
T61I DA neuron
+vehicle
+PF-670462
active-caspase-3
TH
merge
DAPI
5 µm
F
Cells displaying active-caspase-3 (%)
100
80
60
40
20
0
*
+vehicle
+PF-670462
T61I DA neuron
G
T61I DA neuron
+vehicle
+PF-670462
10 µm
Abnormal cell shapes
H
Abnormal cell shapes (%)
100
80
60
40
20
0
*
+vehicle
+PF-670462
T61I DA neuron

Appendix Fig. S18. Presence of dying dopaminergic neurons generated from patient-derived iPSCs

(A, B) The various types of DA neurons generated from iPSCs were cultured for a few days after differentiation. Then, cells were fixed and stained with anti-active caspase-3 and anti-TH antibodies. In **(A)**, representative images are shown. Dotted lines indicate the cell shape. In **(B)**, quantification of cells with active caspase-3 signals is shown ($n \geq 100$ cells in each experiment). Data are shown as the mean ± SD ($n = 3$ experiments). **(C, D)** The various types of DA neurons generated from iPSCs were cultured for a few days. Then, cells were fixed and observed by differential interference contrast microscopy. Representative images are shown in **(C)**. Arrowheads indicate cells with abnormal round shapes and shortened neurites. In **(D)**, the population of cells with abnormal round shapes was calculated ($n \geq 100$ cells in each experiment). Data are shown as the mean ± SD ($n = 3$). **(E–H)** Similar experiments to **(A–D)** were performed in the presence or absence of PF-670462 for 20 hr. Comparisons were performed using one-way ANOVA followed by the Tukey post-hoc test **(B, D)** and the unpaired two-tailed Student *t*-test **(F, H)**. $^{*}p < 0.05$; $^{**}p < 0.01$

Appendix Table S1. Used primers

Names	Sequences
CHCHD2-P2L-Fw	ATTCGCCACCATGCTGCGTGGAAGCCGAAG
CHCHD2-P2L-Rv	CTTCGGCTTCCACGCAGCATGGTGGCGAAT
CHCHD2-S5R-Fw	ATGCCGCGTGGAAGGCGAAGCCGCACCTCC
CHCHD2-S5R-Rv	GGAGGTGCGGCTTCGCCTTCCACGCGGCAT
CHCHD2-A32T-Fw	GGCCAGCACCAGTCACTCAGCCACCAGCAG
CHCHD2-A32T-Rv	CTGCTGGTGGCTGAGTGACTGGTGCTGGCC
CHCHD2-V66M-Fw	CCACTGCAGCTGGCATGGCTGTGGGCTCTG
CHCHD2-V66M-Rv	CAGAGCCCACAGCCATGCCAGCTGCAGTGG
CHCHD2-I80V-Fw	CATTGGGTCACGCCGTTACTGGGGGCTTCA
CHCHD2-I80V-Rv	TGAAGCCCCCAGTAACGGCGTGACCCAATG
CHCHD2-S85R-Fw	ACTGGGGGCTTCAGAGGAGGAAGTAATGCT
CHCHD2-S85R-Rv	AGCATTACTTCCTCCTCTGAAGCCCCCAGT
human CHCHD2-delta30-Fw	CGCGAATTCGCCACCATGGTCGCTCAGCCAC CA
human CHCHD2-delta52-Fw	CGCGAATTCGCCACCATGCCAGGTCTGATGG CC
human CHCHD2-Rv	CAGCCTCGAGGGCCAATCCGTTTGCAAGTC
CHCHD2 (T61I) transgenic XhoI PCR-Fw	CAGCCTCGAGATGCCGCGTGGAAGCCGAAG
CHCHD2 (T61I) transgenic XhoI PCR-Rv	CAGCCTCGAGTTACATTAAACCATTTGCAA
mouse CHCHD2-T61I-Fw	TGATGGCCCAGATGGCTATCACCGCGGCCG
mouse CHCHD2-T61I-Rw	CGGCCGCGGTGATAGCCATCTGGGCCATCA
pCMV6-cHA-Fw	GGCCGTACCCCTACGACGTGCCCGACTACGC CT
pCMV6-cHA-Rv	AGGCGTAGTCGGGCACGTCGTAGGGGTAC
CHCHD2 (T61I) transgenic Genotyping 1	TGCTCCATTTTGCGTGACTC
CHCHD2 (T61I) transgenic Genotyping 2 (pCMV6-cHA-Rv)	AGGCGTAGTCGGGCACGTCGTAGGGGTAC
CHCHD2 (T61I) Knock-in 5' arm cloning-Fw	GAAGACGAGCCTTGGTAAATATGAGC
CHCHD2 (T61I) Knock-in 5' arm cloning-Rv	ACAAGGGACTATCTTGATGACAGATG
CHCHD2 (T61I) Knock-in 5' arm Hind III-Fw	GATCAAGCTTACAGAATTTTCTTTCTCATA
CHCHD2 (T61I) Knock-in 5' arm Sal I-Fw	GATCGTCGACAAATGGTAGACATCTTGGAC
CHCHD2 (T61I) Knock-in exon2-4 arm cloning-Fw	TGTGGTAAACGATCTATTGAGCTACC
CHCHD2 (T61I) Knock-in exon2-4 arm cloning-Rv	AGGATAGGCCATTTACACAGATCTGG
CHCHD2 (T61I) Knock-in exon2-4 arm Sal I-Fw	GATCGTCGACTGTGGTAAACGATCTATTGAG

CHCHD2 (T61I) Knock-in exon2-4 arm Mlu I-Rv	GATCACGCGTGAATACTAGACAAGCCTGCTG
CHCHD2 (T61I) Knock-in 3' arm cloning- Xba I-Mlu I-Fw	GATCTCTAGAACGCGTACCAGGCTTGAGTCCT GTC
CHCHD2 (T61I) Knock-in 3' arm cloning-Xba I-Rv	GATCTCTAGAGGTAGATCTTTTGAGTTTGAGT G
CHCHD2 (T61I) Knock-in Genotyping for 9,000 bp-Fw	GTGGGCATCTCTGCATTTGAGCAG
CHCHD2 (T61I) Knock-in Genotyping for 9,000 bp-Rw	CAAATGGCTCAGAGGAGACAAAGC
CHCHD2 (T61I) Knock-in Genotyping 5' long-Fw	GCATCTCTGCATTTGAGCAG
CHCHD2 (T61I) Knock-in Genotyping 5' long-Rv	GGTGATATTAGGAATGCAGTCC
CHCHD2 (T61I) Knock-in Genotyping 3' long-Fw	GCCACCACACTAGCTTTGTTG
CHCHD2 (T61I) Knock-in Genotyping 3' long-Rv	GAACTACATCAGGGGTGACAC

Appendix Table S2. Antibody list

name	clone	Campany	Cat. Number	method	dilution
HA-Tag	F-7	Santa Cruz	sc-7392	WB, IF	1:200, 1:100
Anti-HA-tag pAb		MBL	561	IF	1:100
COX IV 3E11		Cell Signaling	4850	WB	1:500
α-Tubulin	DM1A	Sigma-Aldrich	T9026	WB	1:3,000
Tom20		Santa Cruz	sc-11415	IF	1:200
Anti-Tyrosine Hydroxylase Antibody		Merck Millipore	AB152	WB, IF	1:500, 1:100
Anti-Tyrosine Hydroxylase Antibody	LNC1	Merck Millipore	AB318	IF	1:100
CHCHD2 Antibody		Proteintech	19424-1-AP	WB, IF	1:200, 1:100
Anti-ANT1/2	5F51B B5AG7	Abcam	Ab110322	IF	1:50
Anti-68kDa		Abcam	ab24520	IF	1:100
Neurofilament-L	C28E1	Cell Signaling Tec.	2837T	WB	1:200
Anti-phospho-NFL (NEFL) (Ser473) Antibody mouse	4F8	Merck Millipore	MABN2431	WB, IF	1:200, 1:200
Synuclein	AB_39 8107	BD	610787	WB	1:500
Anti-phosphorylated a-Synuclein pSyn	#64	Wako FUJFILM	015-25191	IF	1:200
casein kinase I epsilon	A-2	Santa Cruz	sc-373912	WB, IF	1:200, 1:100
Anti-Caspase-3,Cleaved(Asp175)	# 269518	R&D systems	MAB835	IF	1:200
Anti-Calbindin D-28k	300	Swant	CB300	IF	1:400
Anti-Fox3 (NeuN)	1B7	BioLegend	834501	IF	1:100
