# Supplementary figures and images for: Involvement of casein kinase 1 epsilon/delta (Csnk1e/d) in the pathogenesis of familial Parkinson's disease caused by CHCHD2
Source: EMBO Mol Med. 2023 Aug 14;15(9):e17451. doi: 10.15252/emmm.202317451 (PMC10493588; doi:10.15252/emmm.202317451)

Source data for Fig. 1A

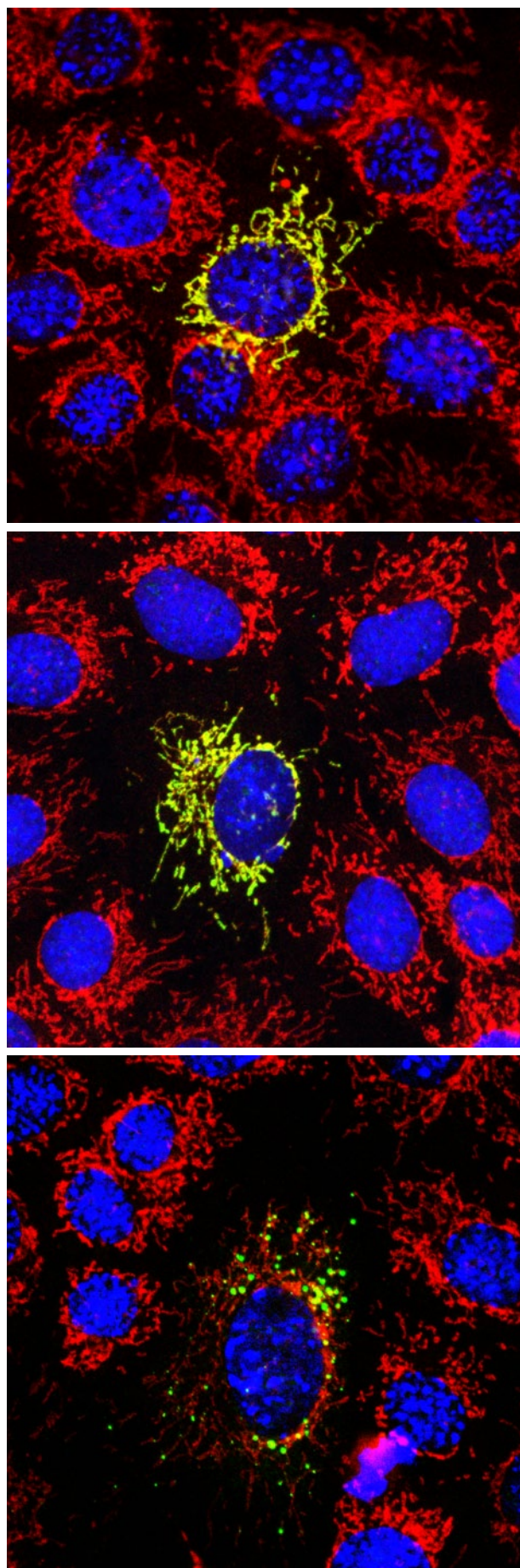

Supplement: Supplementary file 4 — Source Data for Figure 1 [file EMMM-15-e17451-s011.zip › Fig 1/1A/1A.pdf]

Source data for Fig. 1D

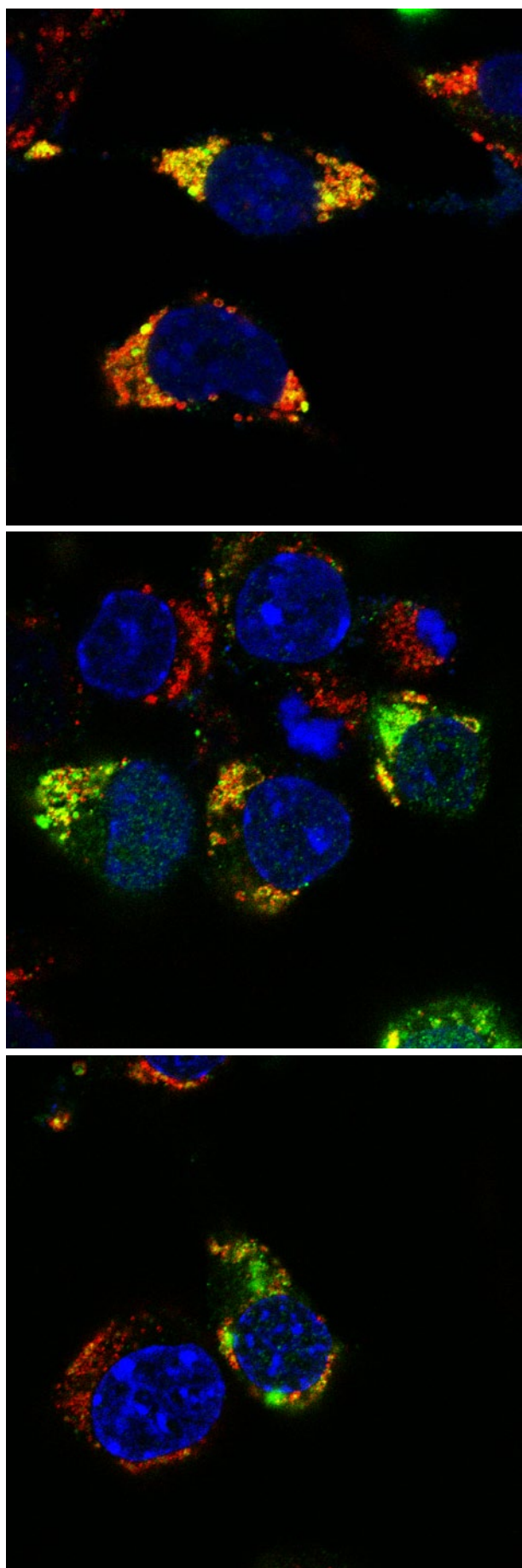

Supplement: Supplementary file 4 — Source Data for Figure 1 [file EMMM-15-e17451-s011.zip › Fig 1/1D/1D.pdf]

Source data for Fig. 1F

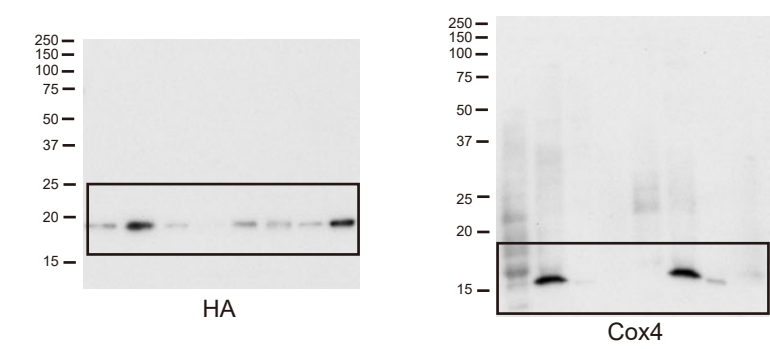

Supplement: Supplementary file 4 — Source Data for Figure 1 [file EMMM-15-e17451-s011.zip › Fig 1/1F/1F.pdf]

Source data for Fig. 2A

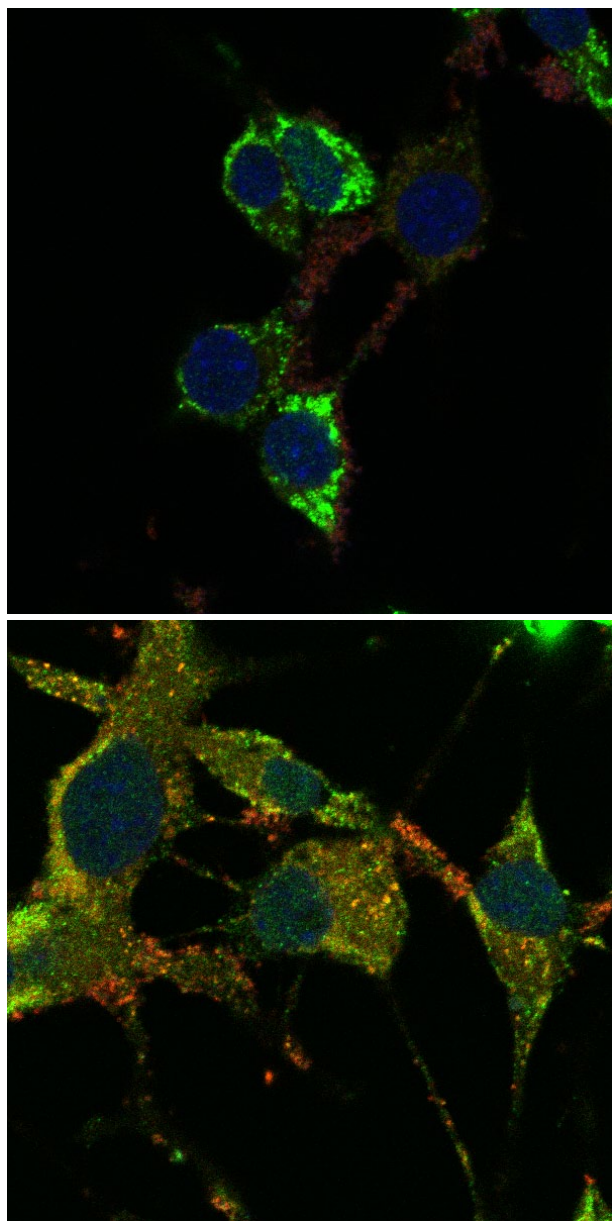

Supplement: Supplementary file 5 — Source Data for Figure 2 [file EMMM-15-e17451-s004.zip › Fig 2/2A/2A.pdf]

Source data for Fig. 2C

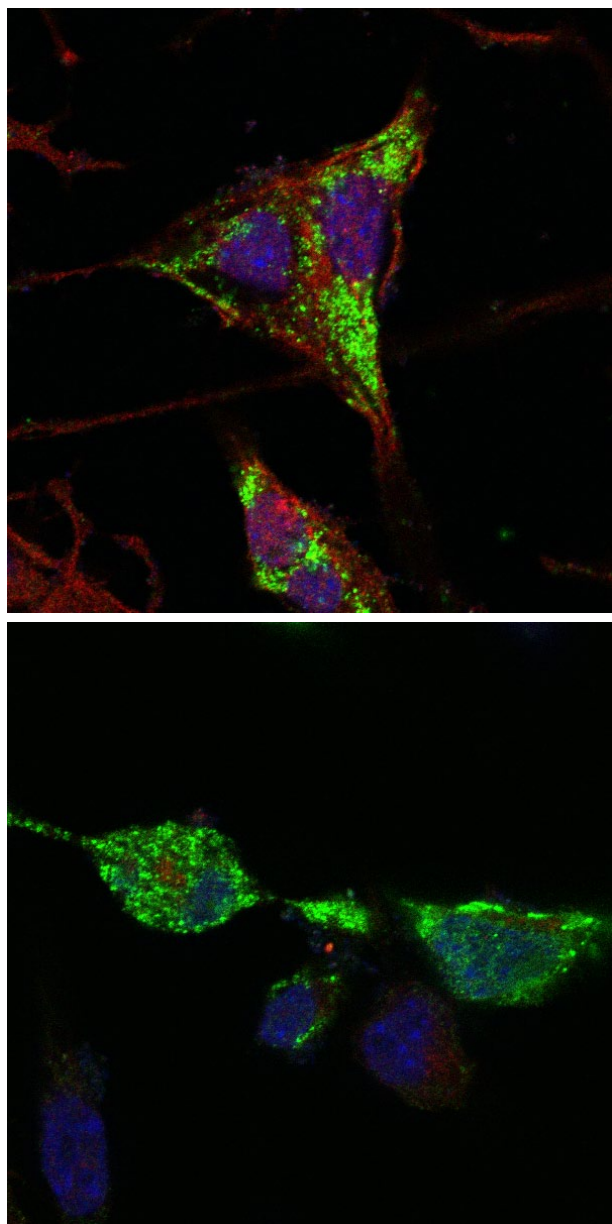

Supplement: Supplementary file 5 — Source Data for Figure 2 [file EMMM-15-e17451-s004.zip › Fig 2/2C/2C.pdf]

Source data for Fig. 2E

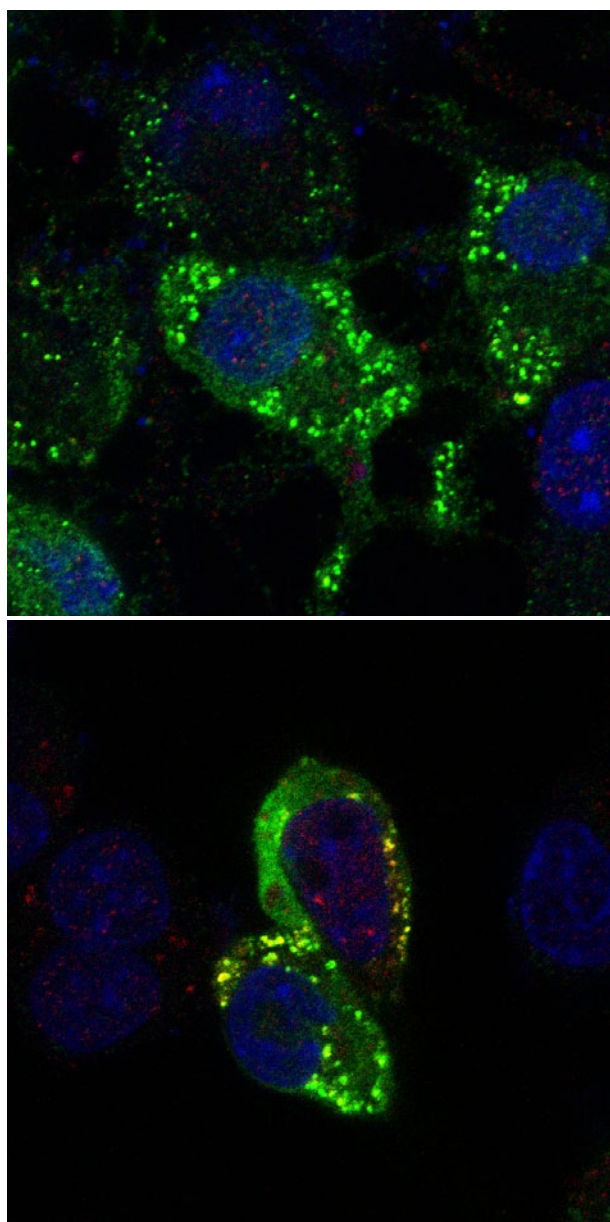

Supplement: Supplementary file 5 — Source Data for Figure 2 [file EMMM-15-e17451-s004.zip › Fig 2/2E/2E.pdf]

Source data for Fig. 2G

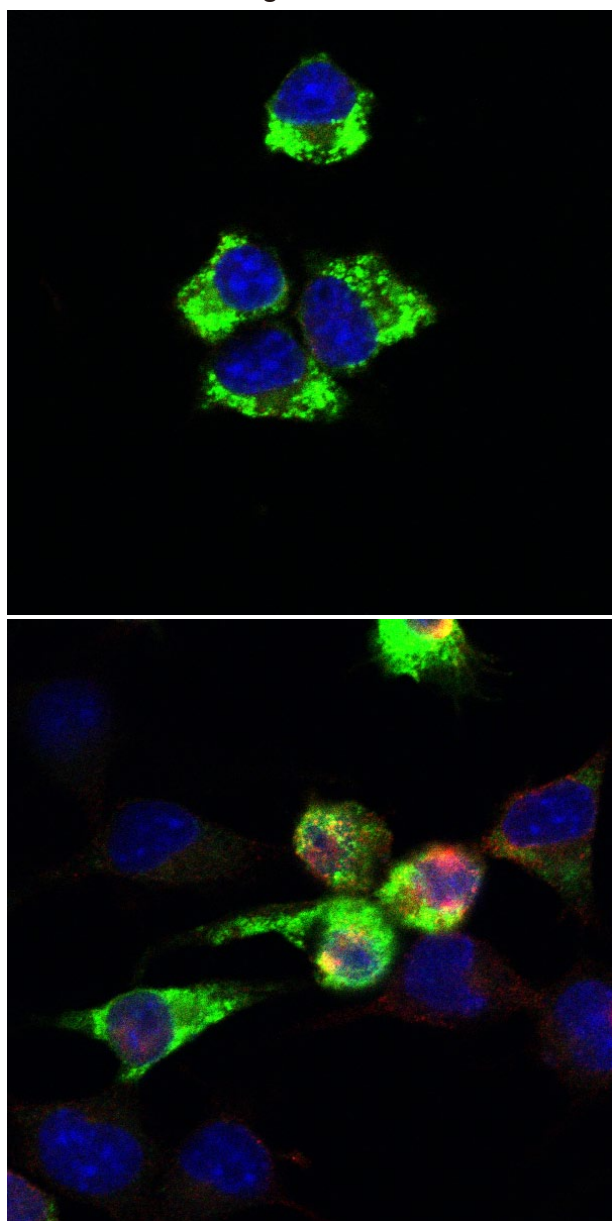

Supplement: Supplementary file 5 — Source Data for Figure 2 [file EMMM-15-e17451-s004.zip › Fig 2/2G/2G.pdf]

Source data for Fig. 2I

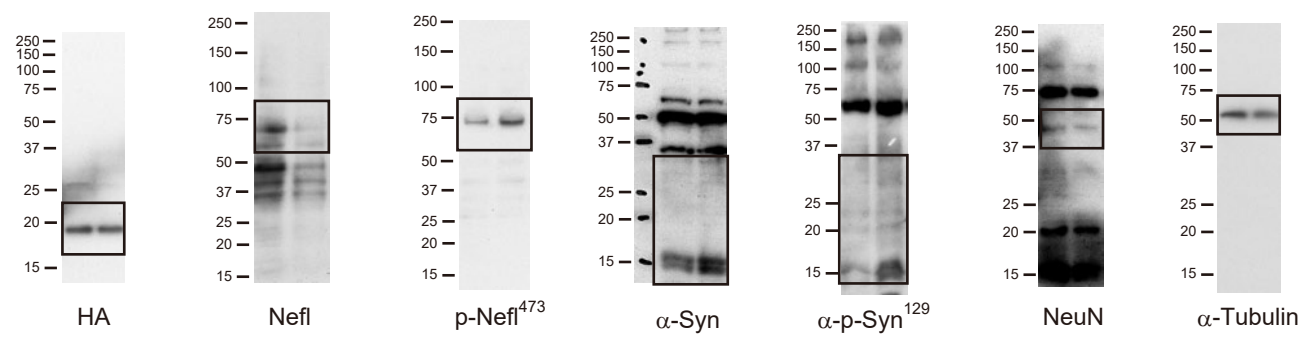

Supplement: Supplementary file 5 — Source Data for Figure 2 [file EMMM-15-e17451-s004.zip › Fig 2/2I/2I.pdf]

Source data for Fig. 3B

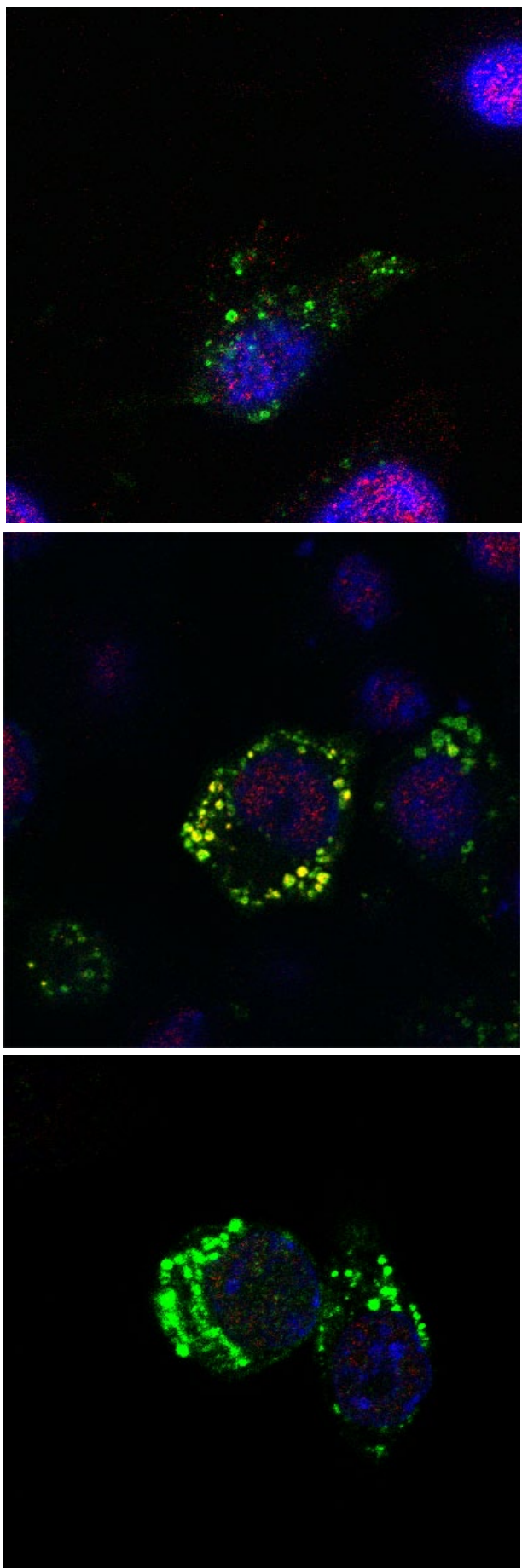

Supplement: Supplementary file 6 — Source Data for Figure 3 [file EMMM-15-e17451-s010.zip › Fig 3/3B/3B.pdf]

Source data for Fig. 3C

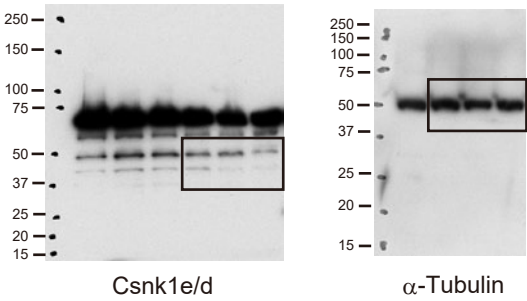

Supplement: Supplementary file 6 — Source Data for Figure 3 [file EMMM-15-e17451-s010.zip › Fig 3/3C/3C.pdf]

Source data for Fig. 3E

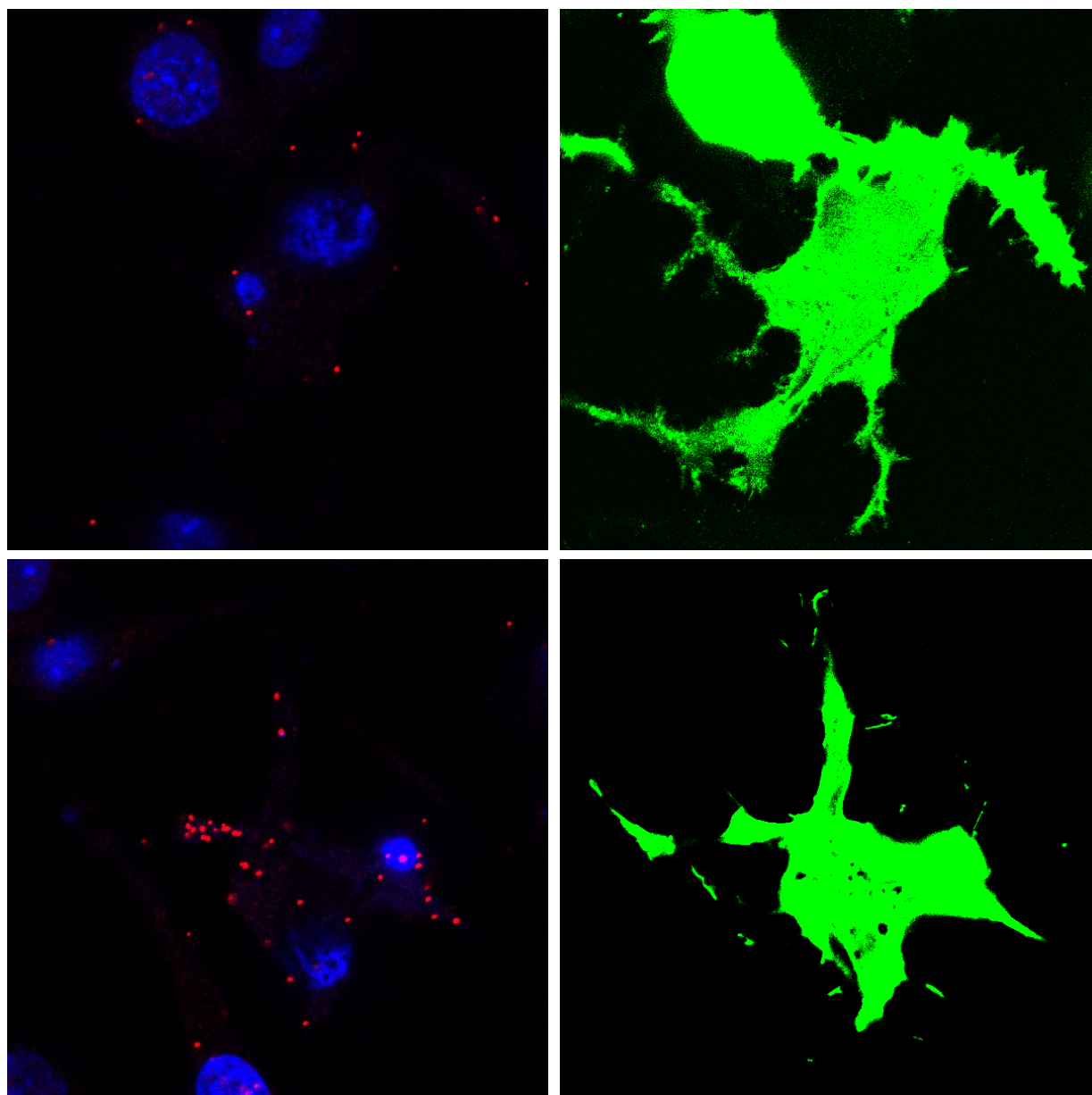

Supplement: Supplementary file 6 — Source Data for Figure 3 [file EMMM-15-e17451-s010.zip › Fig 3/3E/3E.pdf]

Source data for Fig. 4B

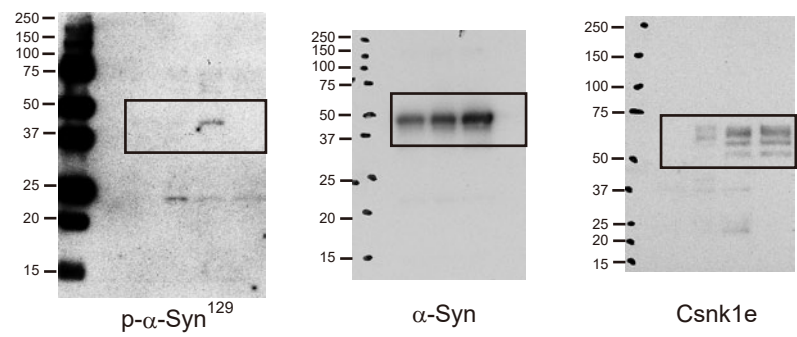

Supplement: Supplementary file 7 — Source Data for Figure 4 [file EMMM-15-e17451-s002.zip › Fig 4/4B/4B.pdf]

Source data for Fig. 4D

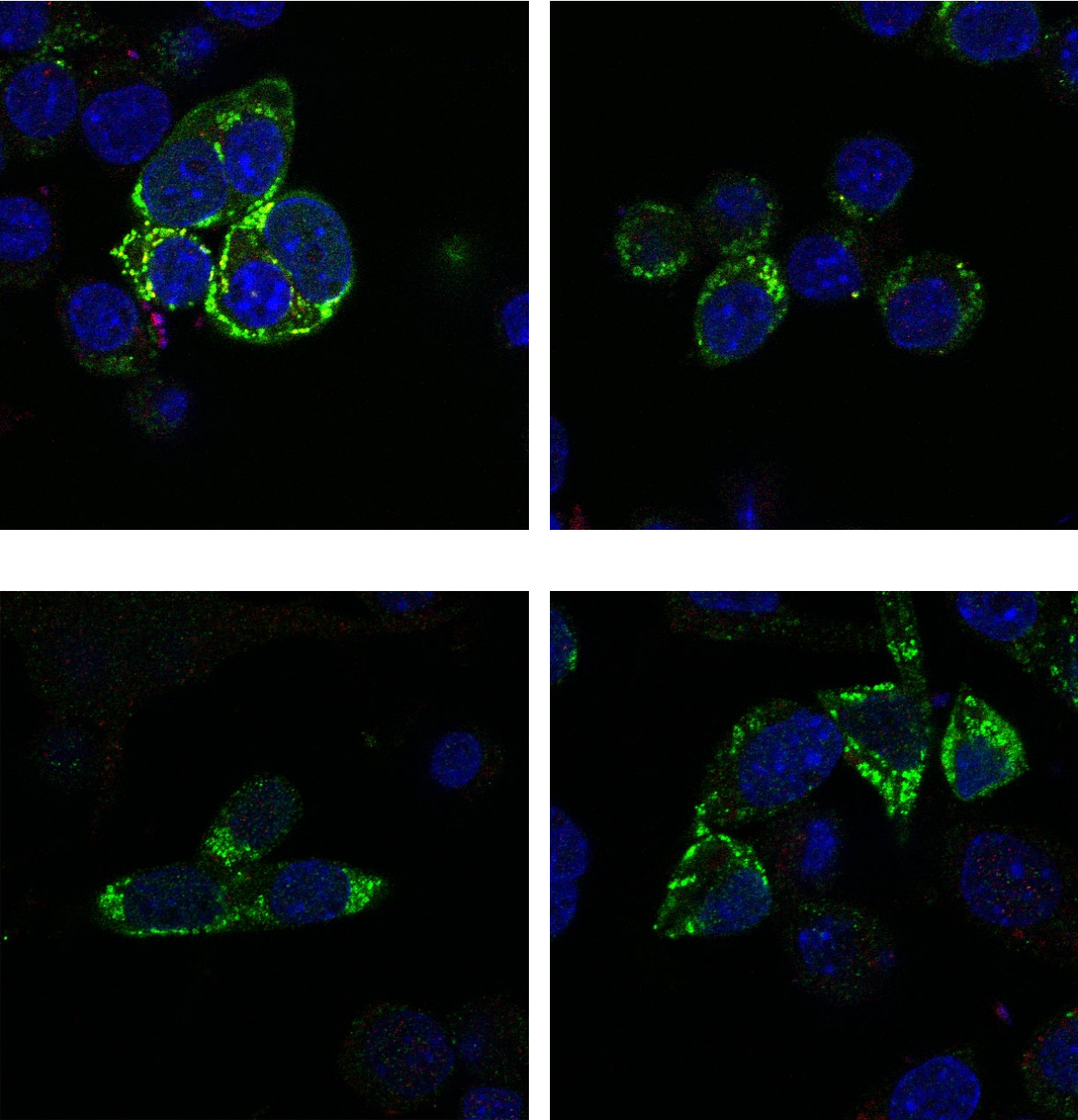

Supplement: Supplementary file 7 — Source Data for Figure 4 [file EMMM-15-e17451-s002.zip › Fig 4/4D/4D.pdf]

Source data for Fig. 4F

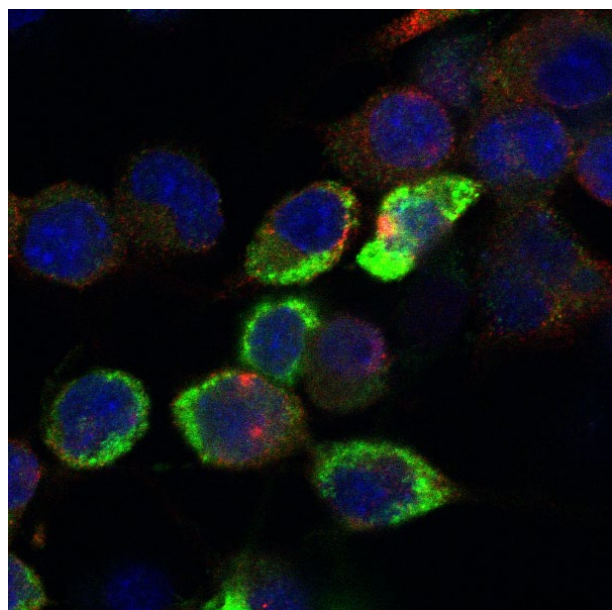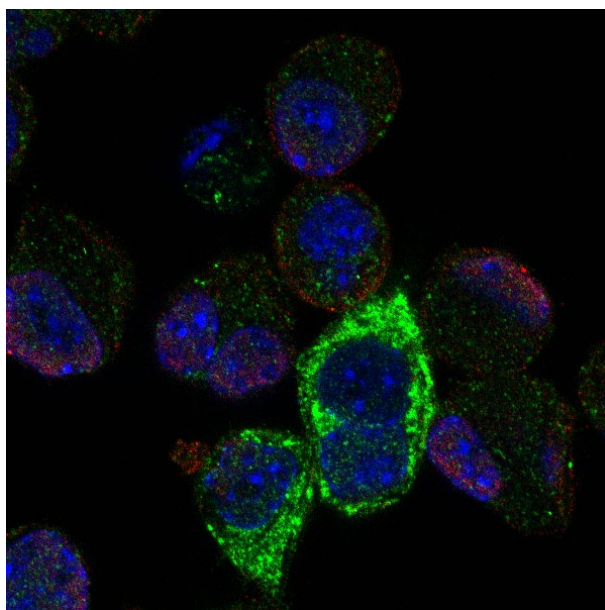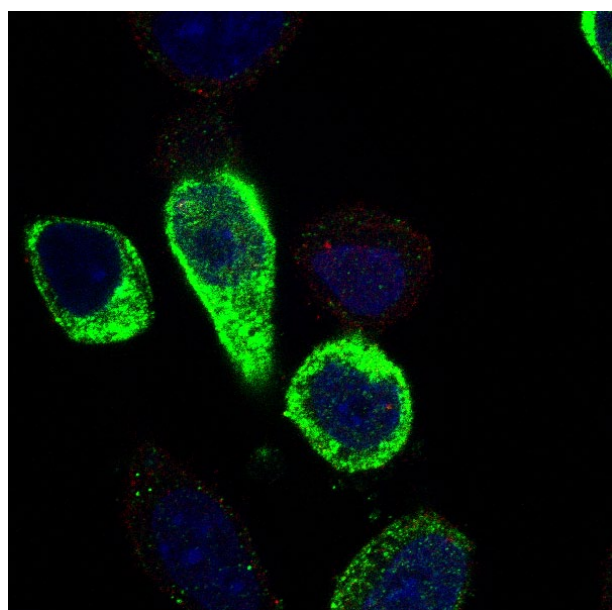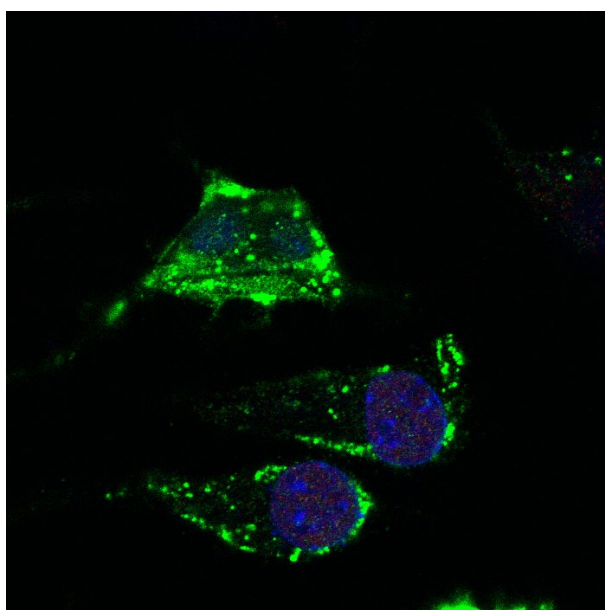

Supplement: Supplementary file 7 — Source Data for Figure 4 [file EMMM-15-e17451-s002.zip › Fig 4/4F/4F.pdf]

Source data for Fig. 4H

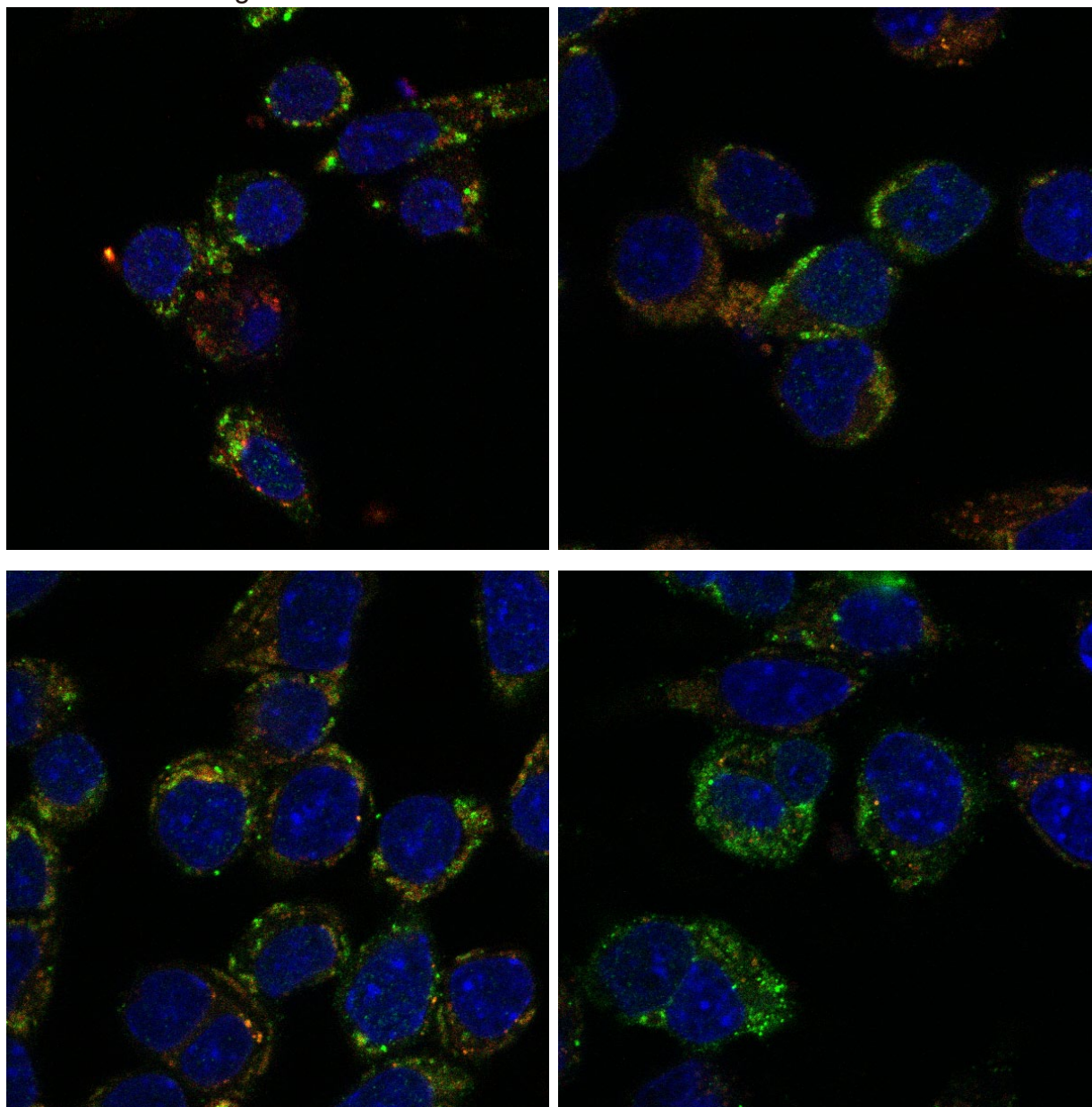

Supplement: Supplementary file 7 — Source Data for Figure 4 [file EMMM-15-e17451-s002.zip › Fig 4/4H/4H.pdf]

Source data for Fig. 5B

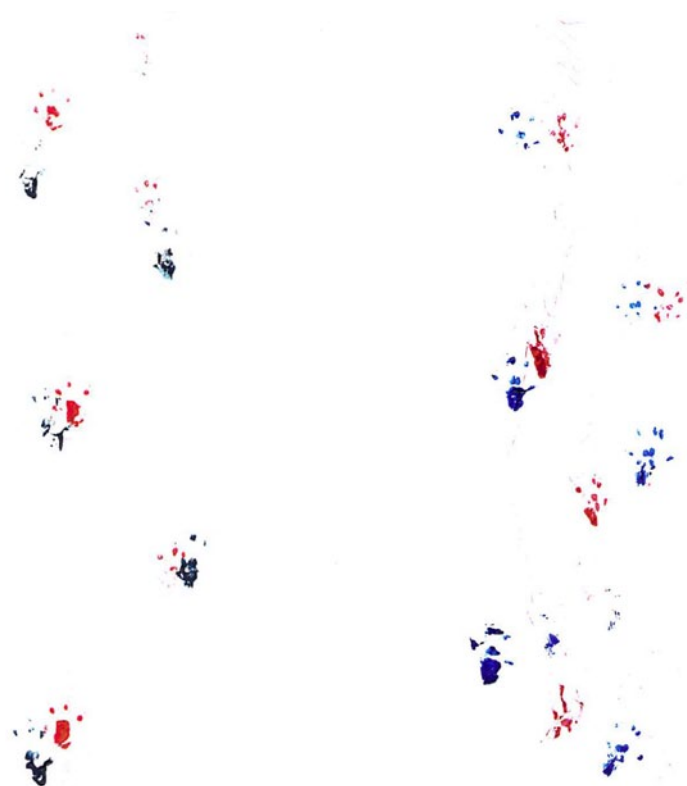

Supplement: Supplementary file 8 — Source Data for Figure 5 [file EMMM-15-e17451-s007.zip › Fig 5/5B/5B.pdf]

Source data for Fig. 5D

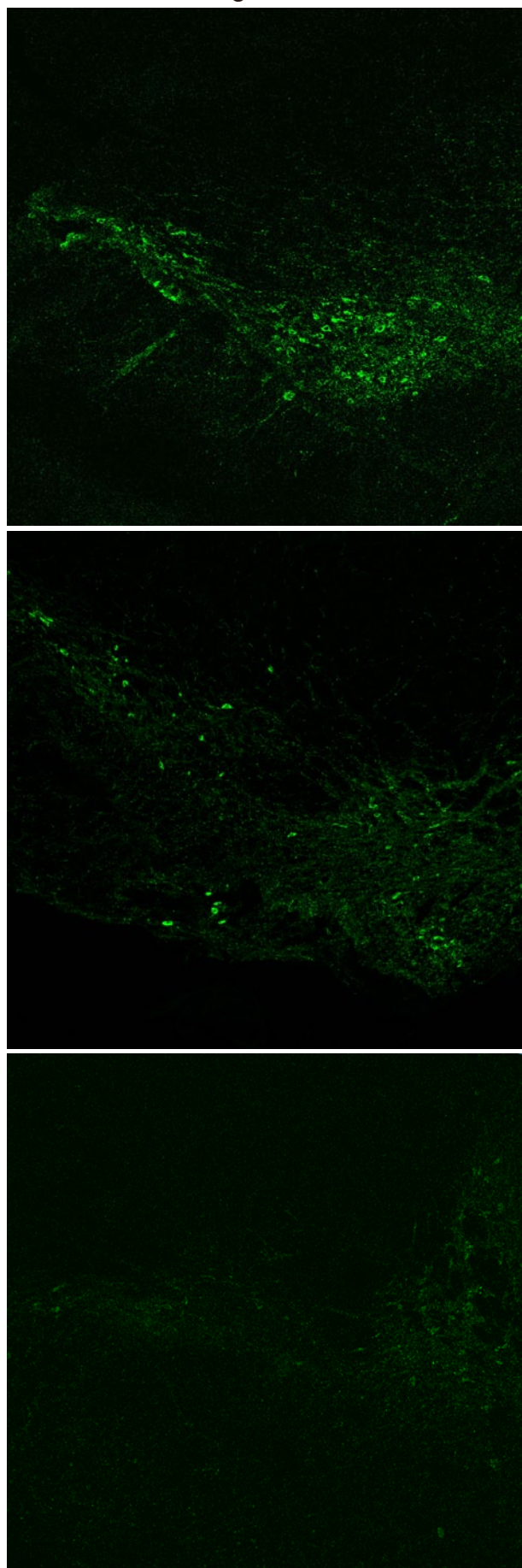

Supplement: Supplementary file 8 — Source Data for Figure 5 [file EMMM-15-e17451-s007.zip › Fig 5/5D/5D.pdf]

Source data for Fig. 5F

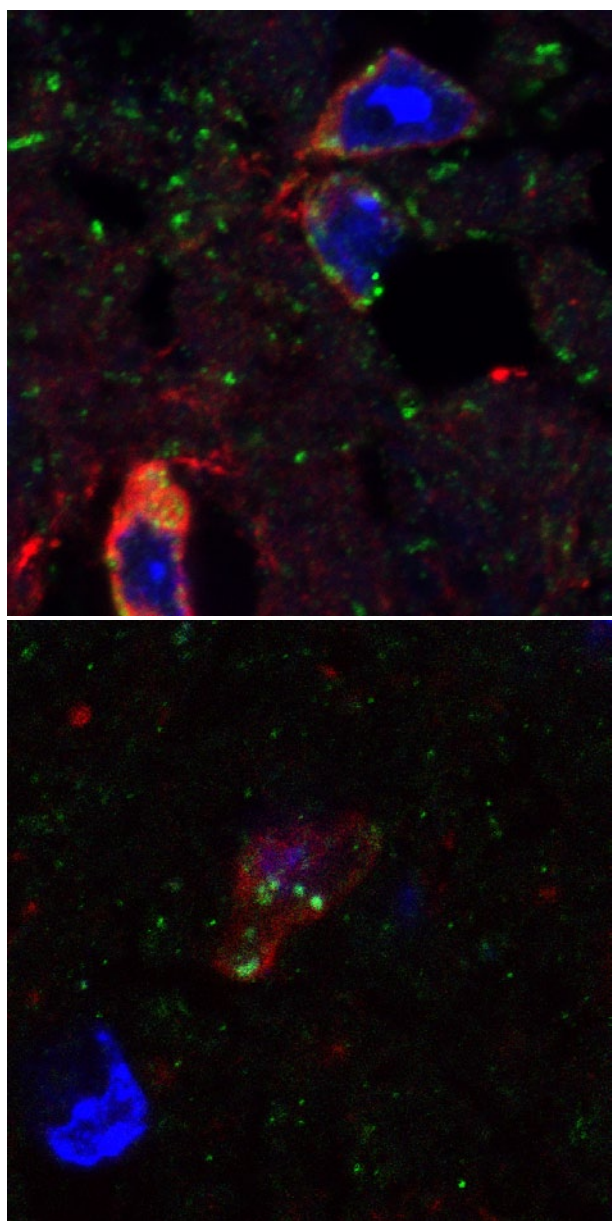

Supplement: Supplementary file 8 — Source Data for Figure 5 [file EMMM-15-e17451-s007.zip › Fig 5/5F/5F.pdf]

Source data for Fig. 5G

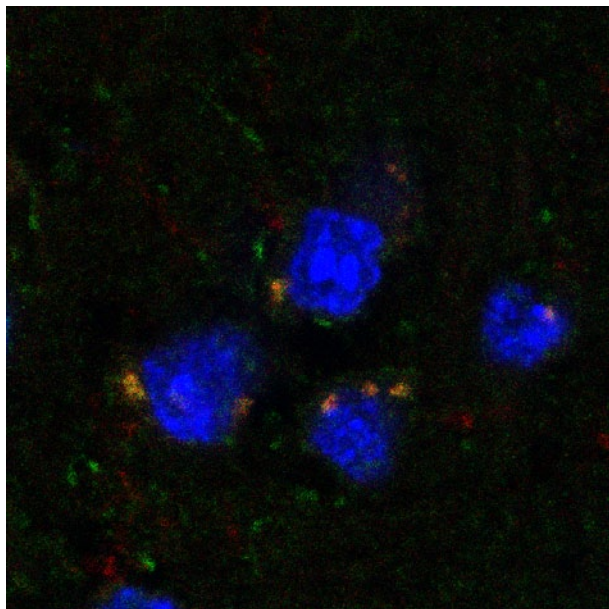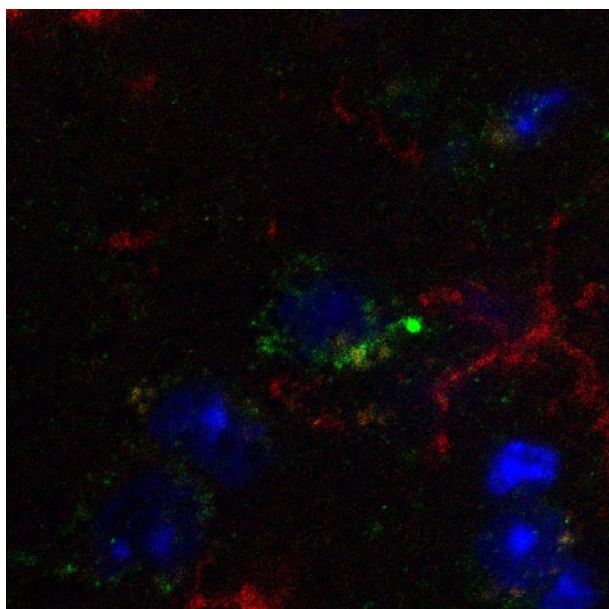

Supplement: Supplementary file 8 — Source Data for Figure 5 [file EMMM-15-e17451-s007.zip › Fig 5/5G/5G.pdf]

Source data for Fig. 5H

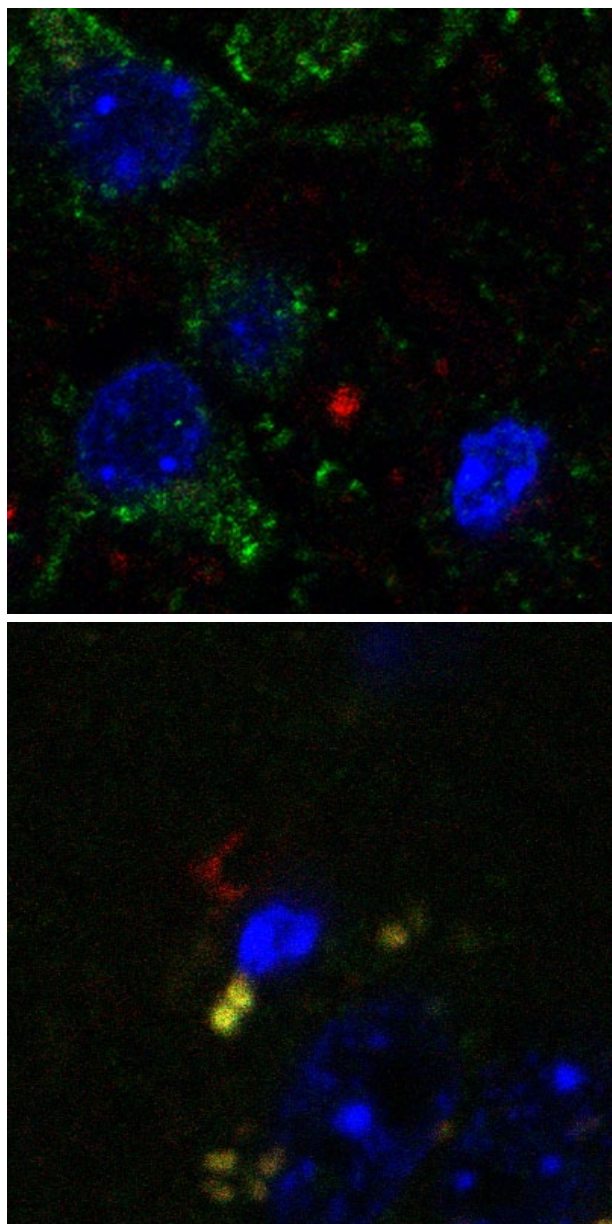

Supplement: Supplementary file 8 — Source Data for Figure 5 [file EMMM-15-e17451-s007.zip › Fig 5/5H/5H.pdf]

Source data for Fig. 5I

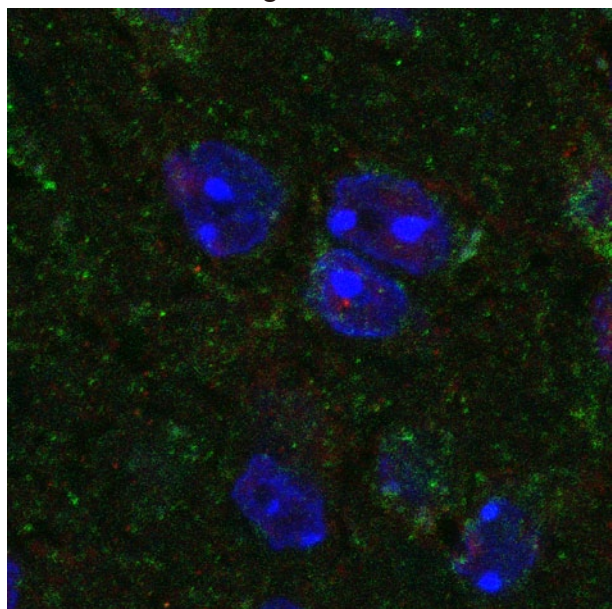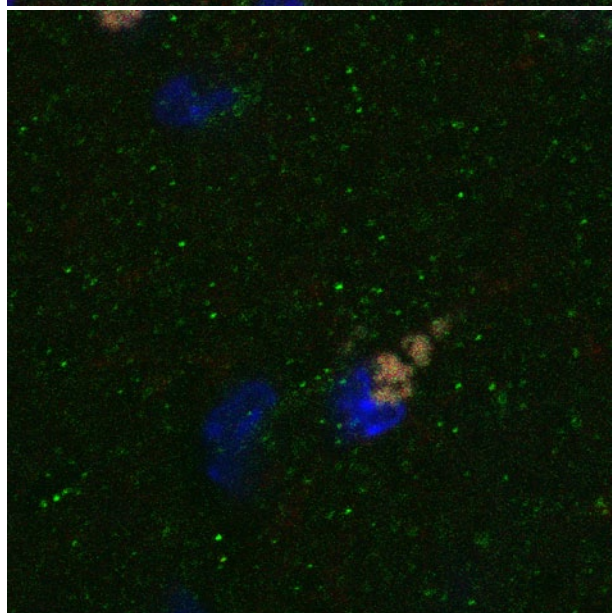

Supplement: Supplementary file 8 — Source Data for Figure 5 [file EMMM-15-e17451-s007.zip › Fig 5/5I/5I.pdf]

Source data for Fig. 5J

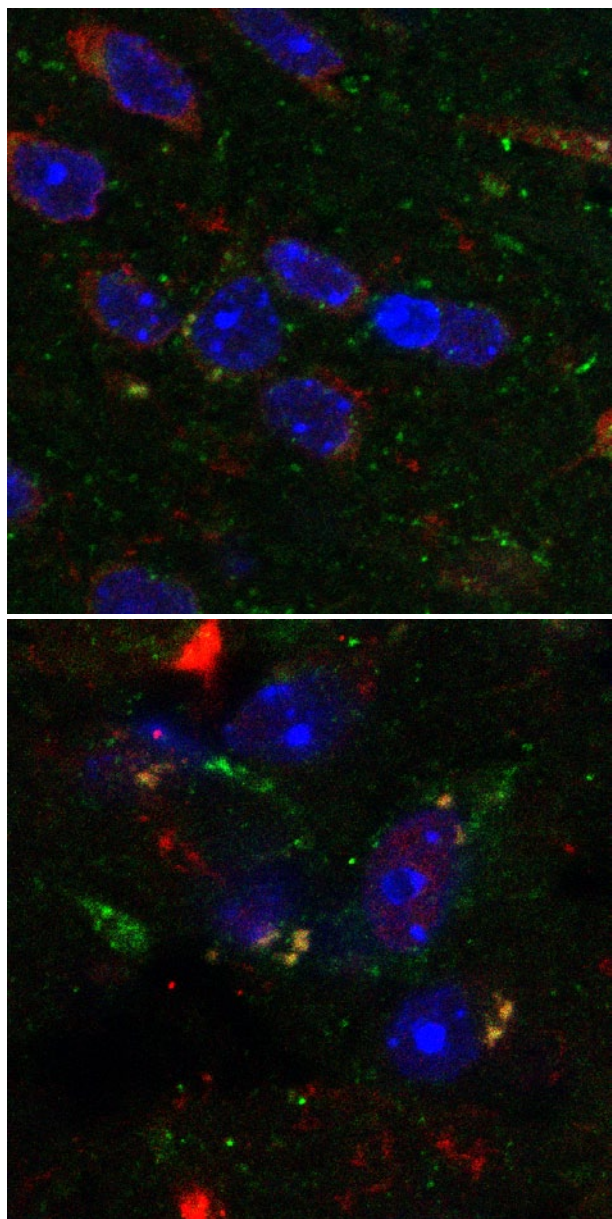

Supplement: Supplementary file 8 — Source Data for Figure 5 [file EMMM-15-e17451-s007.zip › Fig 5/5J/5J.pdf]

Source data for Fig. 5K

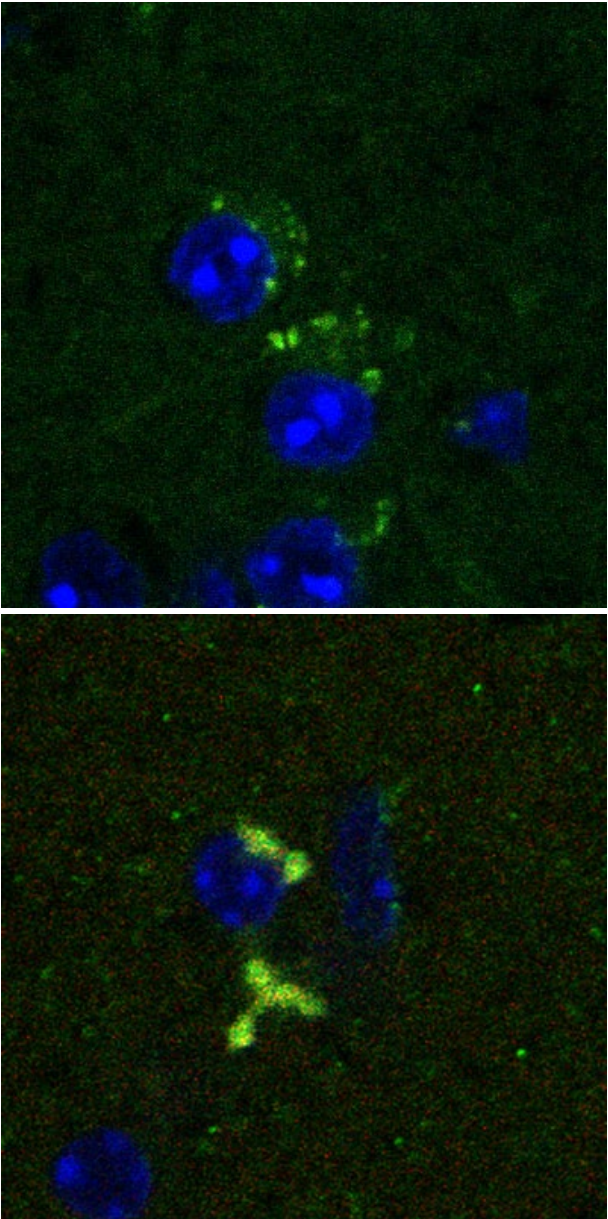

Supplement: Supplementary file 8 — Source Data for Figure 5 [file EMMM-15-e17451-s007.zip › Fig 5/5K/5K.pdf]

Source data for Fig. 5L

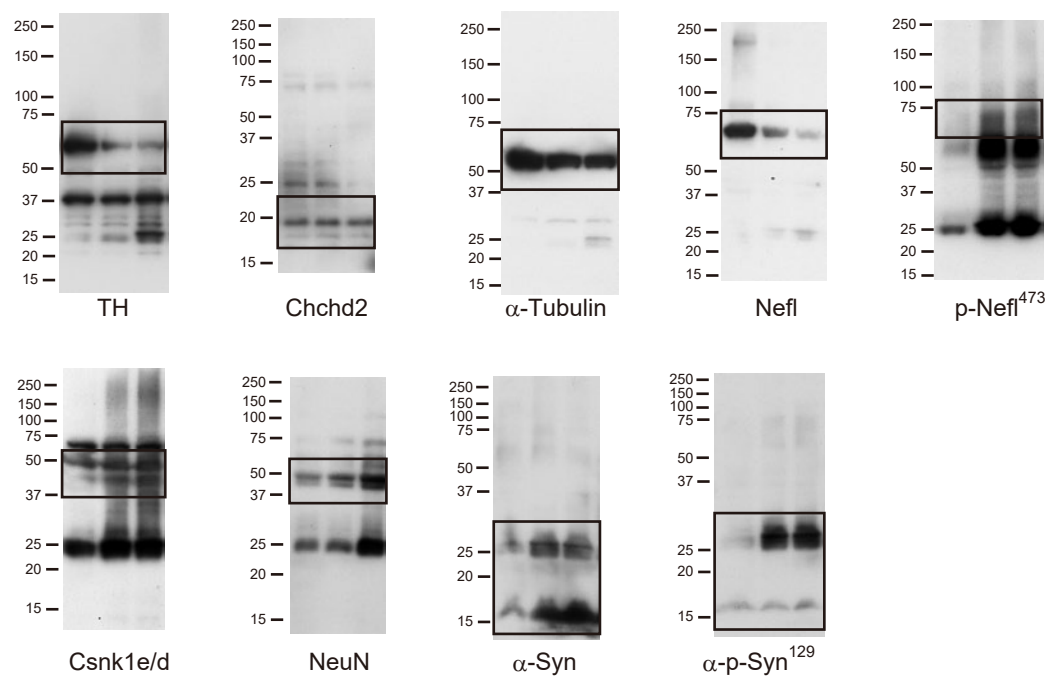

Supplement: Supplementary file 8 — Source Data for Figure 5 [file EMMM-15-e17451-s007.zip › Fig 5/5L/5L.pdf]

Source data for Fig. 5M

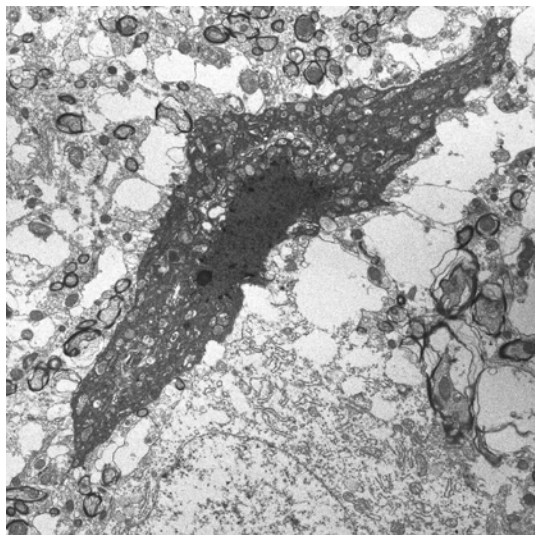

Supplement: Supplementary file 8 — Source Data for Figure 5 [file EMMM-15-e17451-s007.zip › Fig 5/5M/5M.pdf]

Source data for Fig. 6B

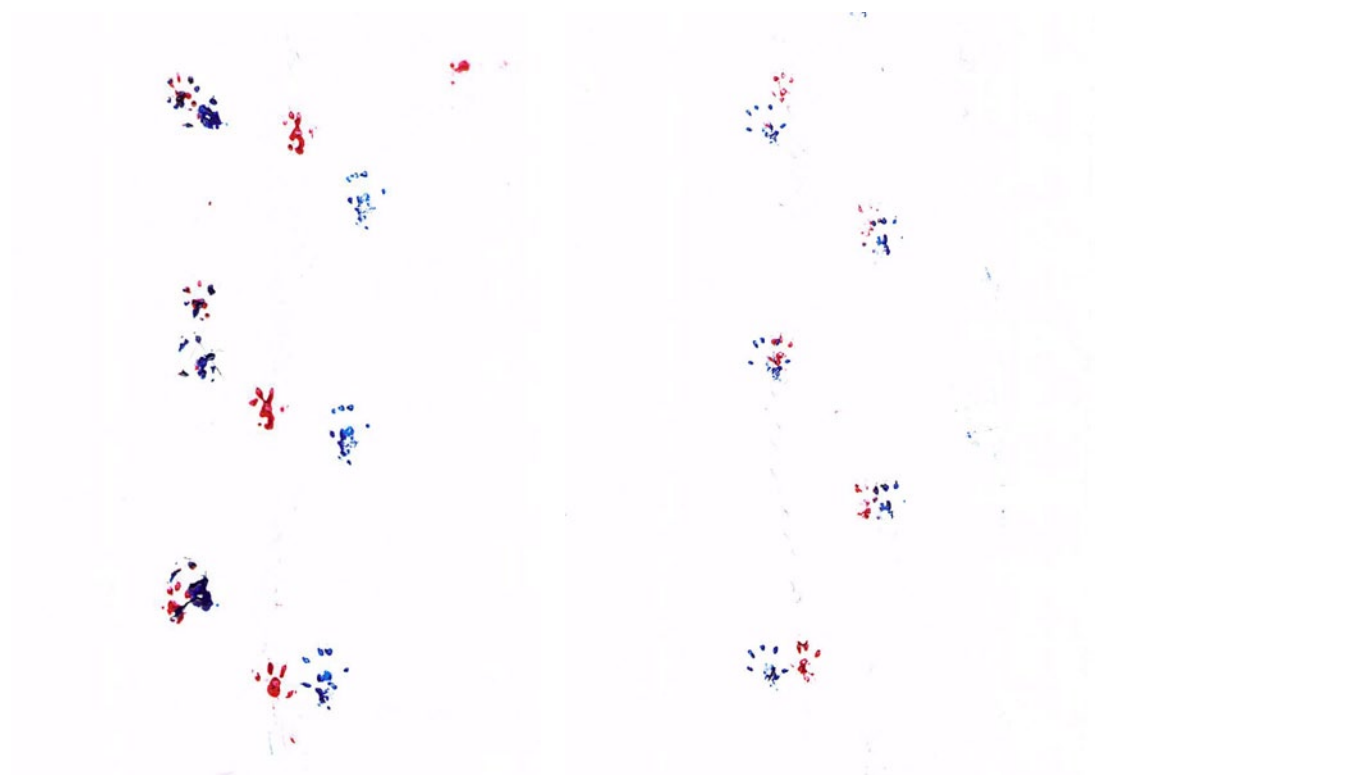

Supplement: Supplementary file 9 — Source Data for Figure 6 [file EMMM-15-e17451-s008.zip › Fig 6/6B/6B.pdf]

Source data for Fig. 6D

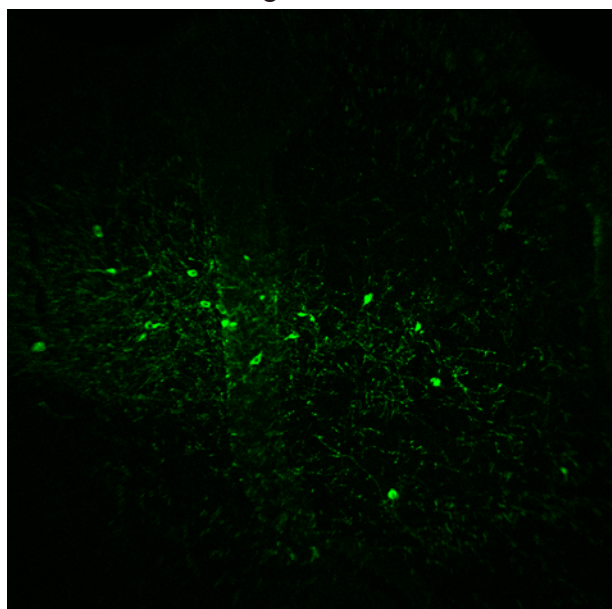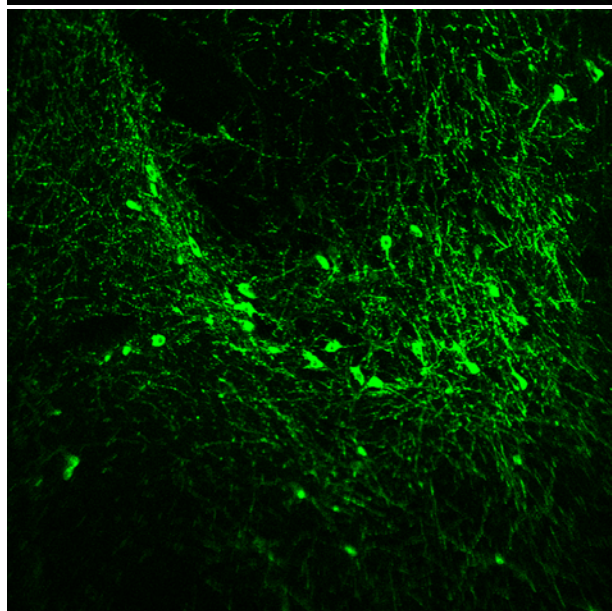

Supplement: Supplementary file 9 — Source Data for Figure 6 [file EMMM-15-e17451-s008.zip › Fig 6/6D/6D.pdf]

Source data for Fig. 6F

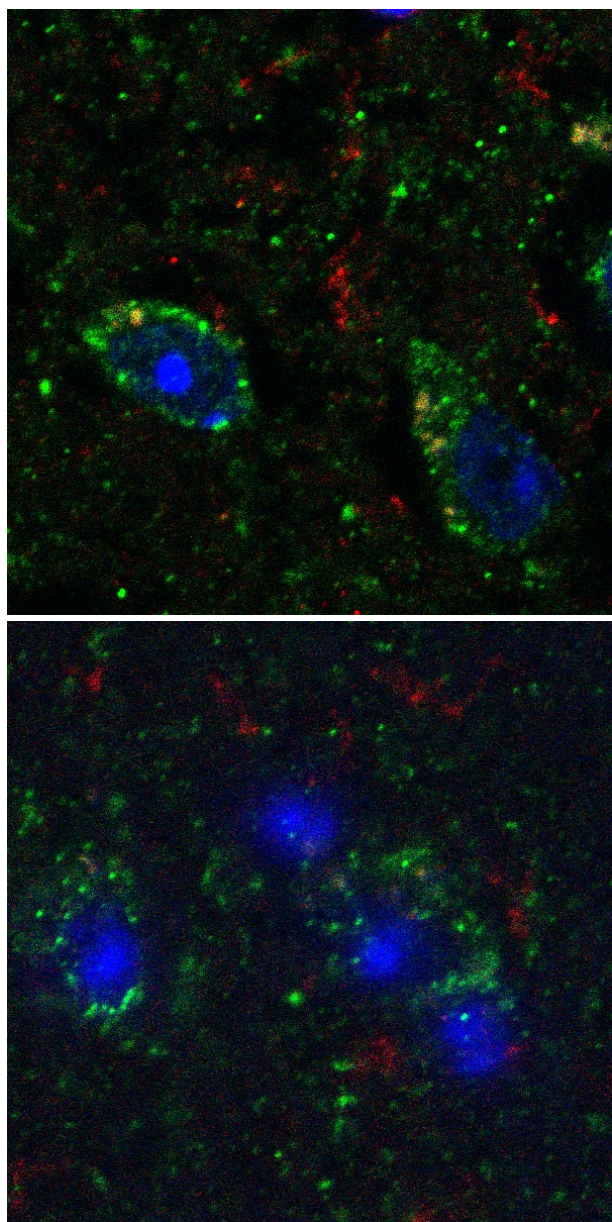

Supplement: Supplementary file 9 — Source Data for Figure 6 [file EMMM-15-e17451-s008.zip › Fig 6/6F/6F.pdf]

Source data for Fig. 6G

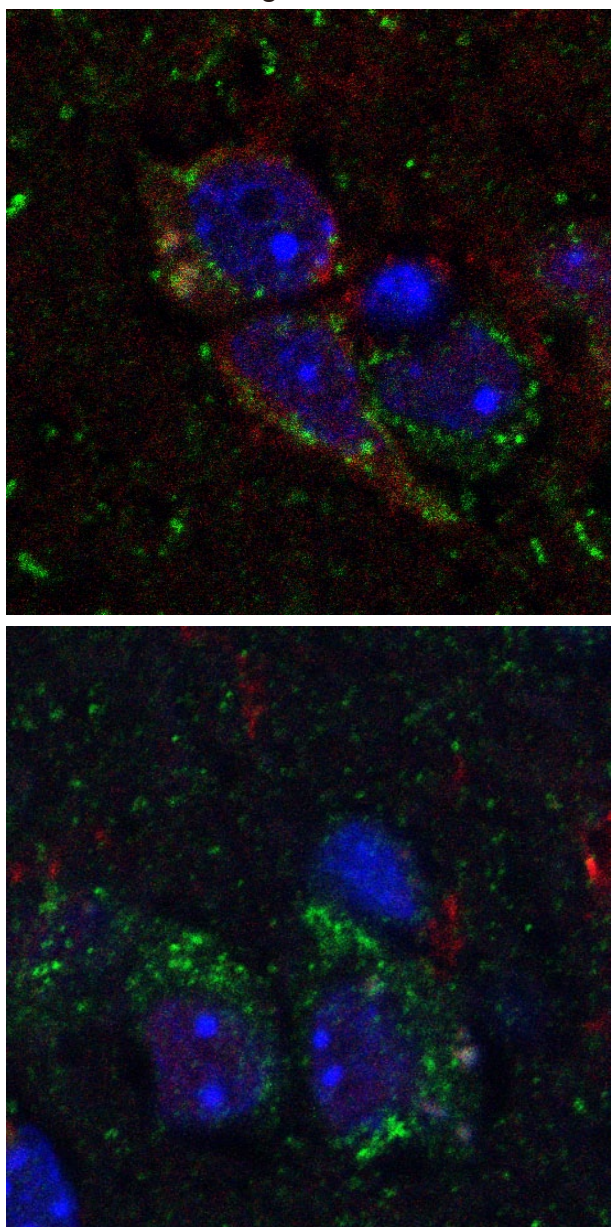

Supplement: Supplementary file 9 — Source Data for Figure 6 [file EMMM-15-e17451-s008.zip › Fig 6/6G/6G.pdf]

Source data for Fig. 6H

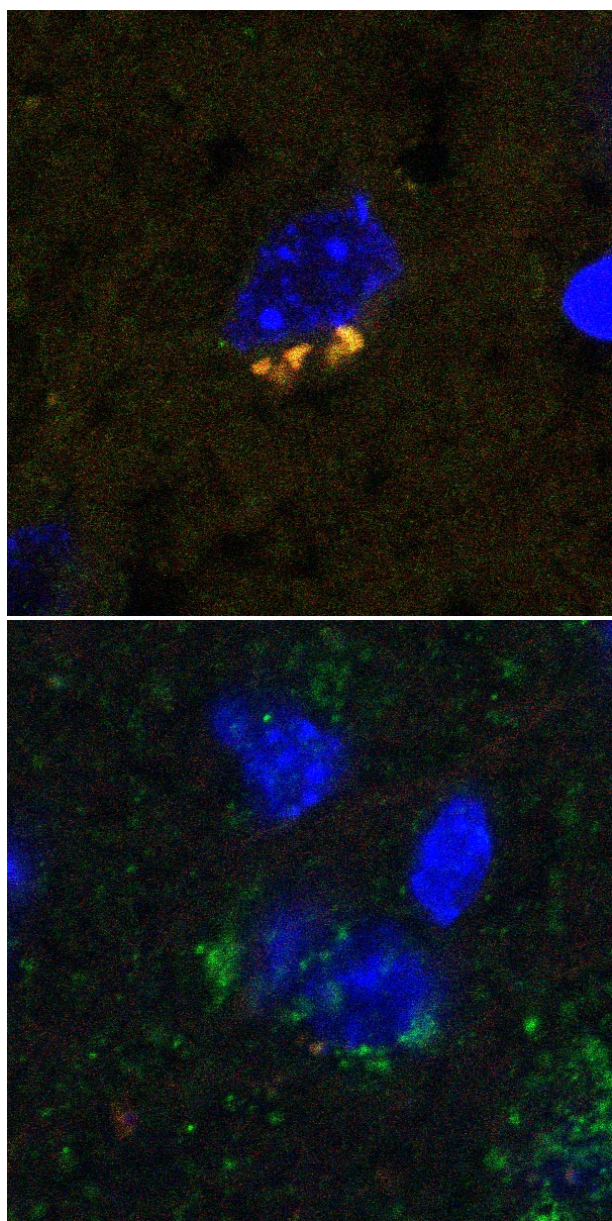

Supplement: Supplementary file 9 — Source Data for Figure 6 [file EMMM-15-e17451-s008.zip › Fig 6/6H/6H.pdf]

Source data for Fig. 6l

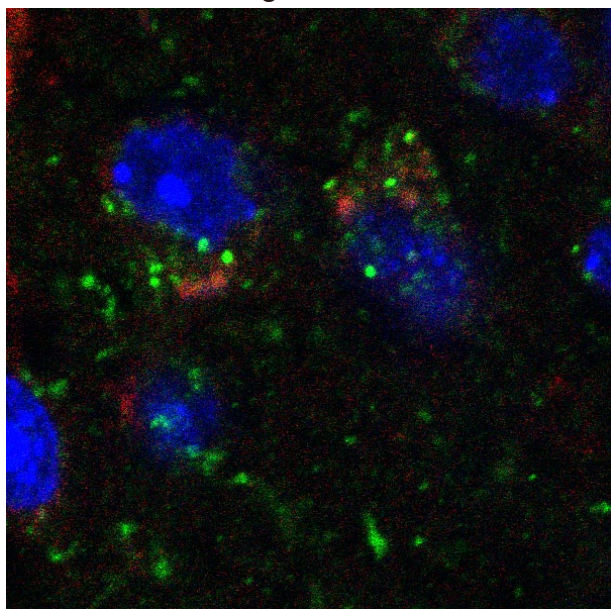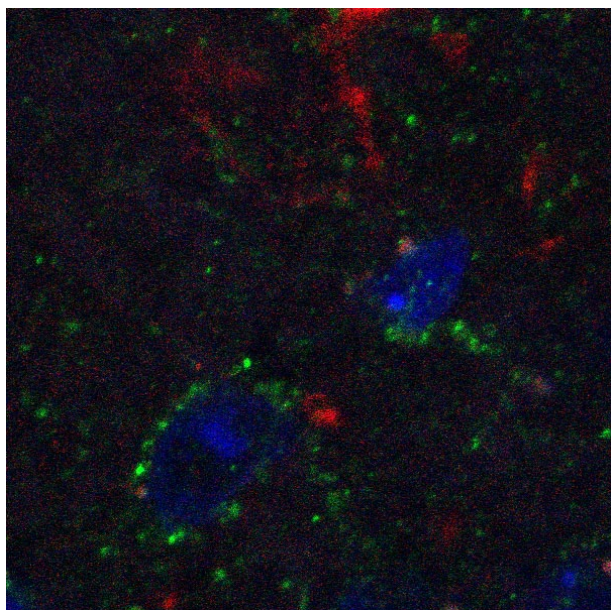

Supplement: Supplementary file 9 — Source Data for Figure 6 [file EMMM-15-e17451-s008.zip › Fig 6/6I/6I.pdf]

Source data for Fig. 6J

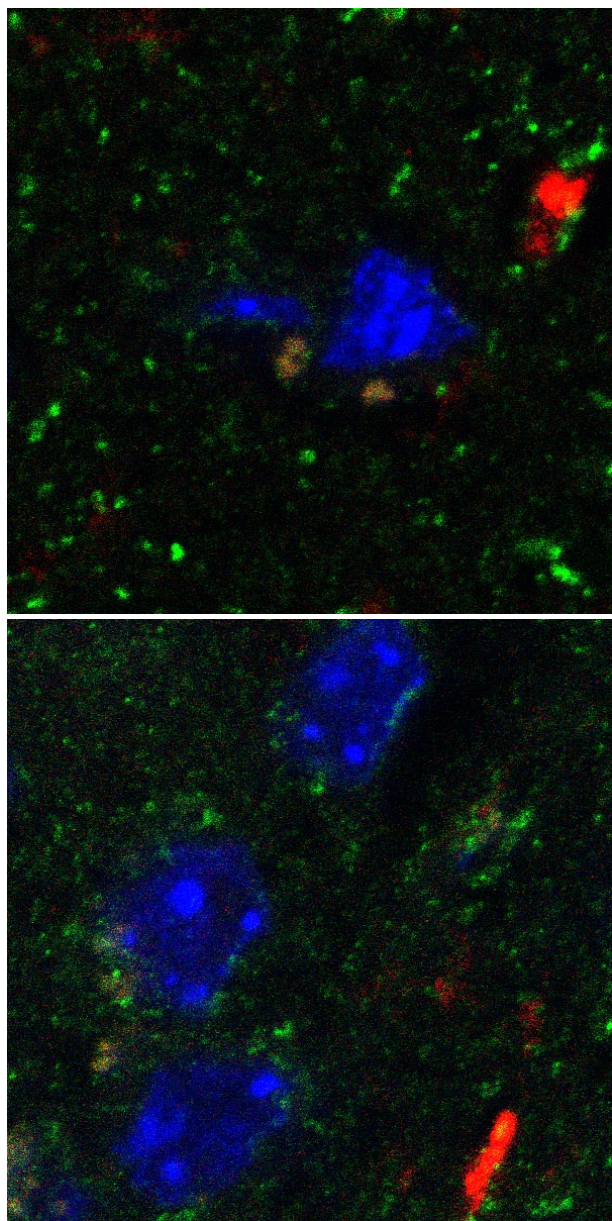

Supplement: Supplementary file 9 — Source Data for Figure 6 [file EMMM-15-e17451-s008.zip › Fig 6/6J/6J.pdf]

Source data for Fig. 7B

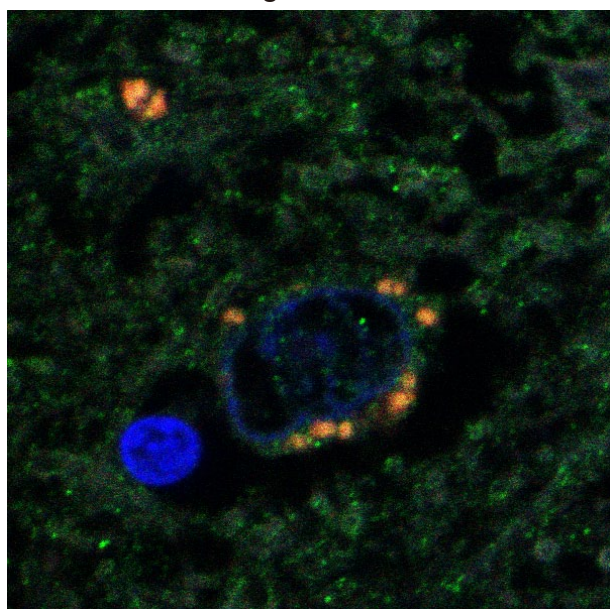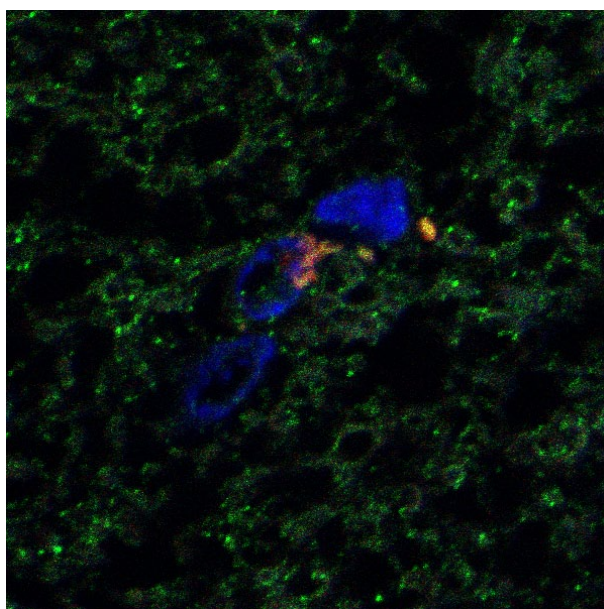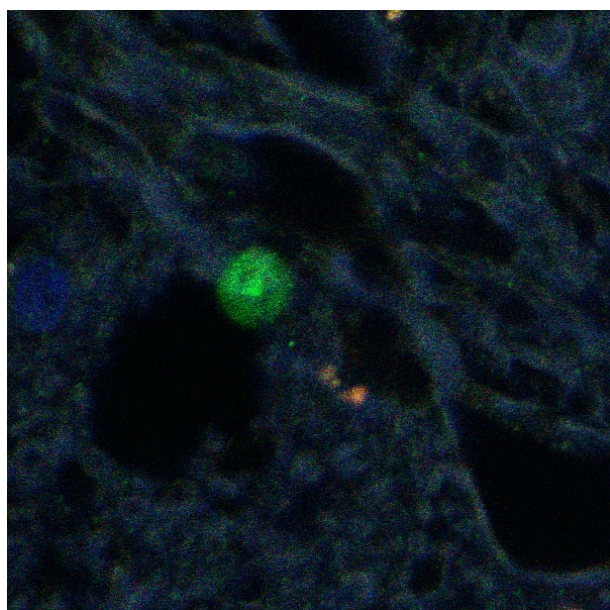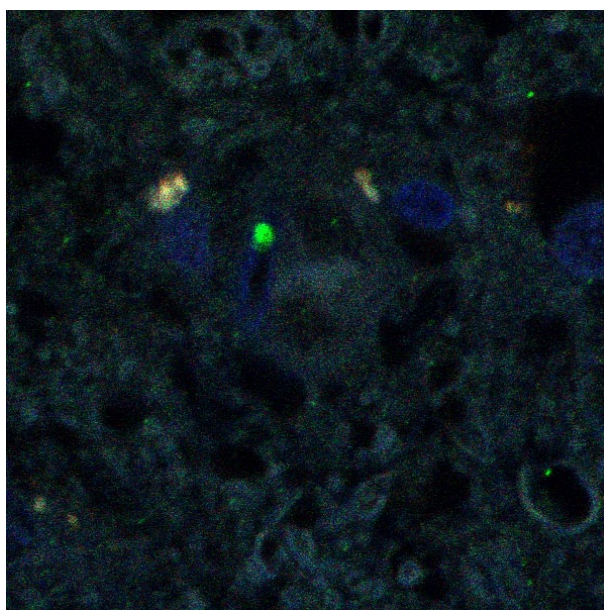

Supplement: Supplementary file 10 — Source Data for Figure 7 [file EMMM-15-e17451-s003.zip › Fig 7/7B/7B.pdf]

Source data for Fig. 7C

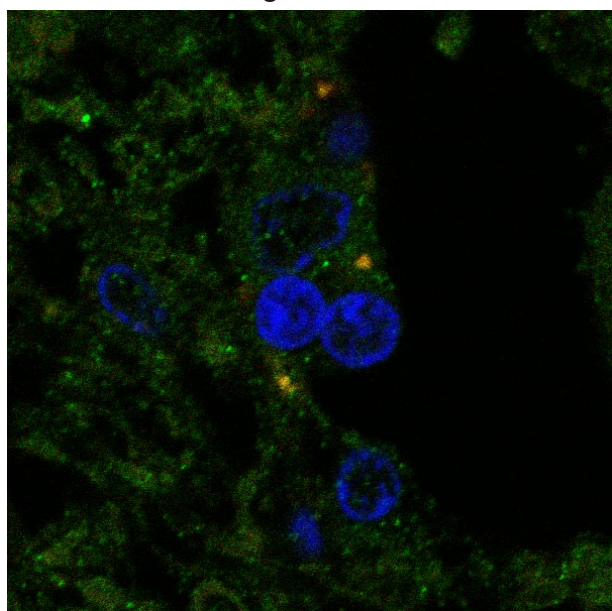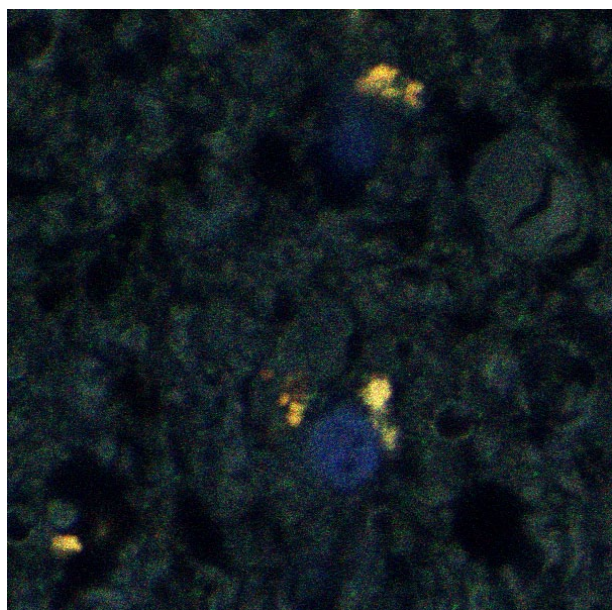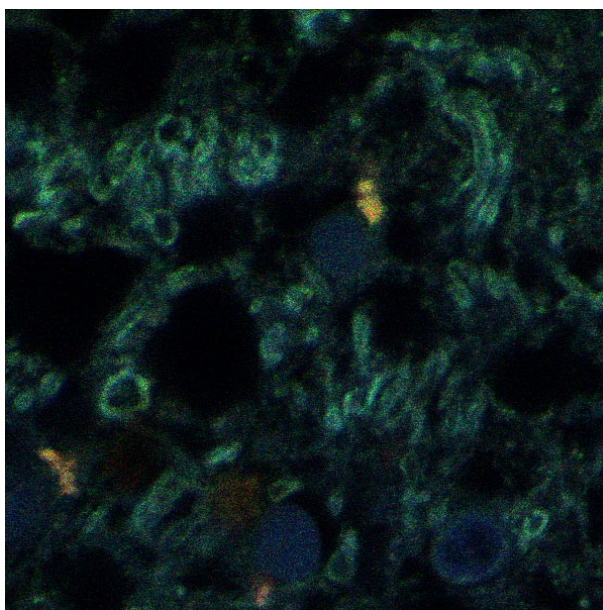

Supplement: Supplementary file 10 — Source Data for Figure 7 [file EMMM-15-e17451-s003.zip › Fig 7/7C/7C.pdf]

Source data for Fig. 7D

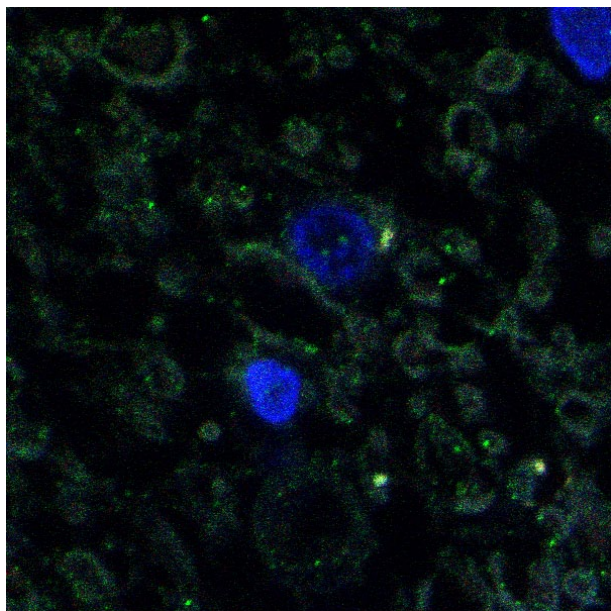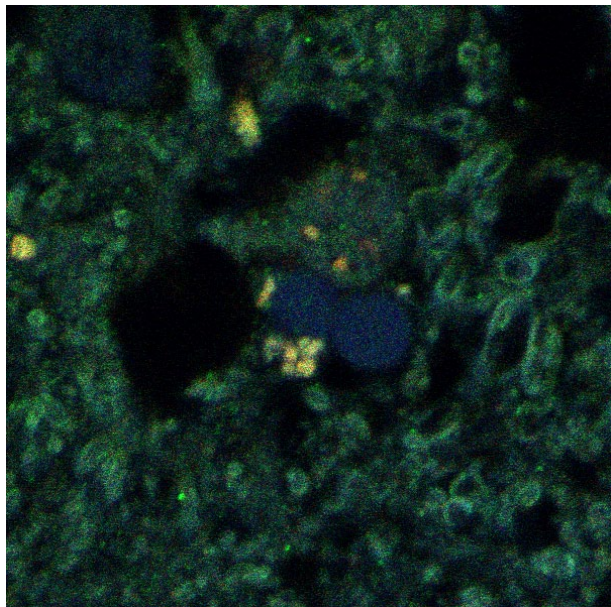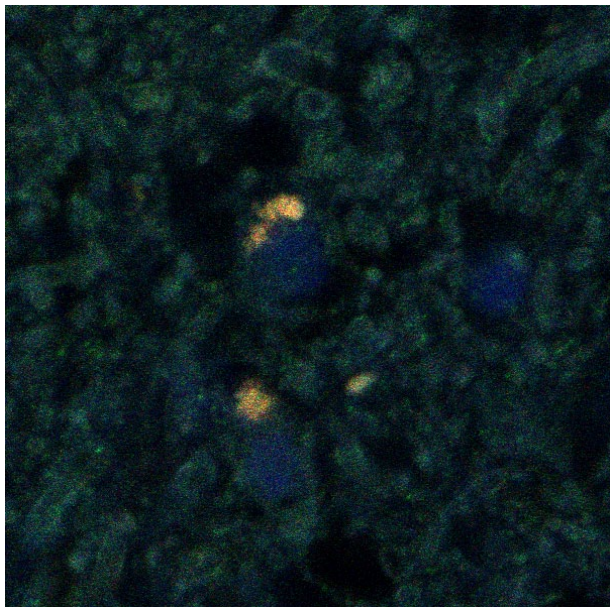

Supplement: Supplementary file 10 — Source Data for Figure 7 [file EMMM-15-e17451-s003.zip › Fig 7/7D/7D.pdf]

Source data for Fig. 7F

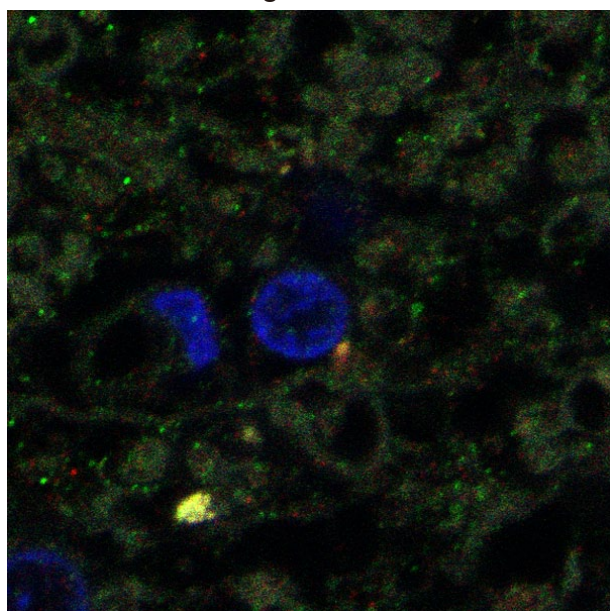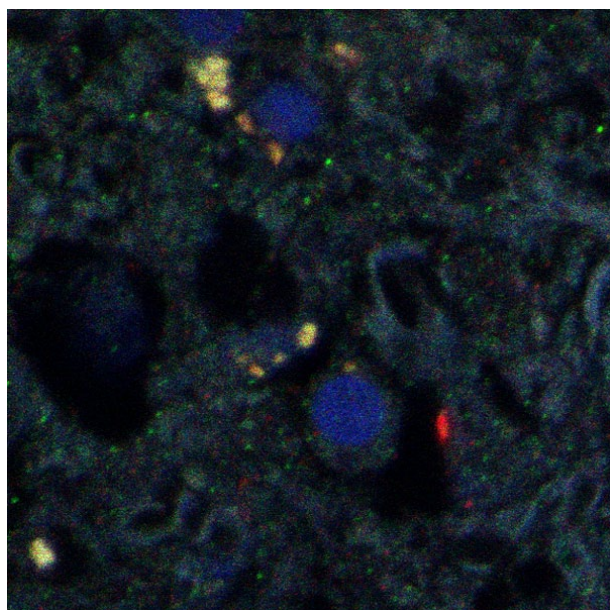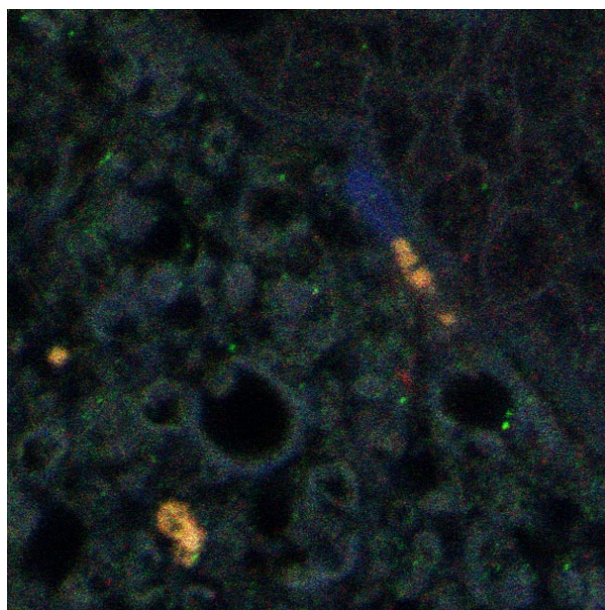

Supplement: Supplementary file 10 — Source Data for Figure 7 [file EMMM-15-e17451-s003.zip › Fig 7/7F/7F.pdf]

Source data for Fig. 8A

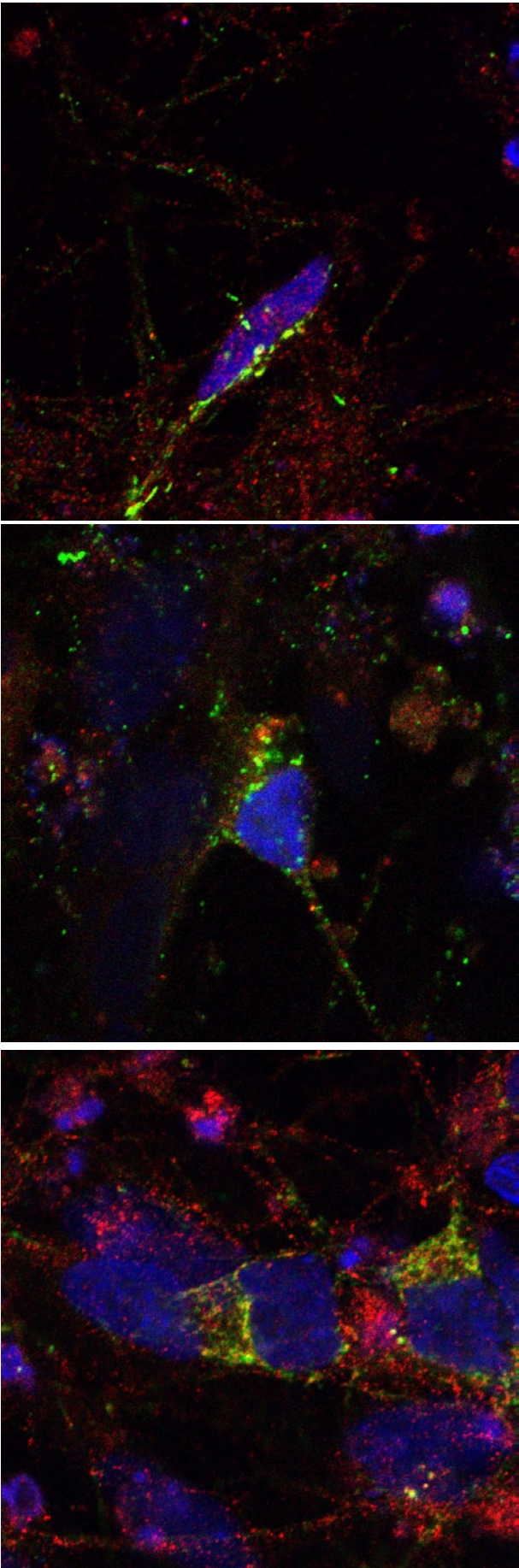

Supplement: Supplementary file 11 — Source Data for Figure 8 [file EMMM-15-e17451-s012.zip › Fig 8/8A/8A.pdf]

Source data for Fig. 8B

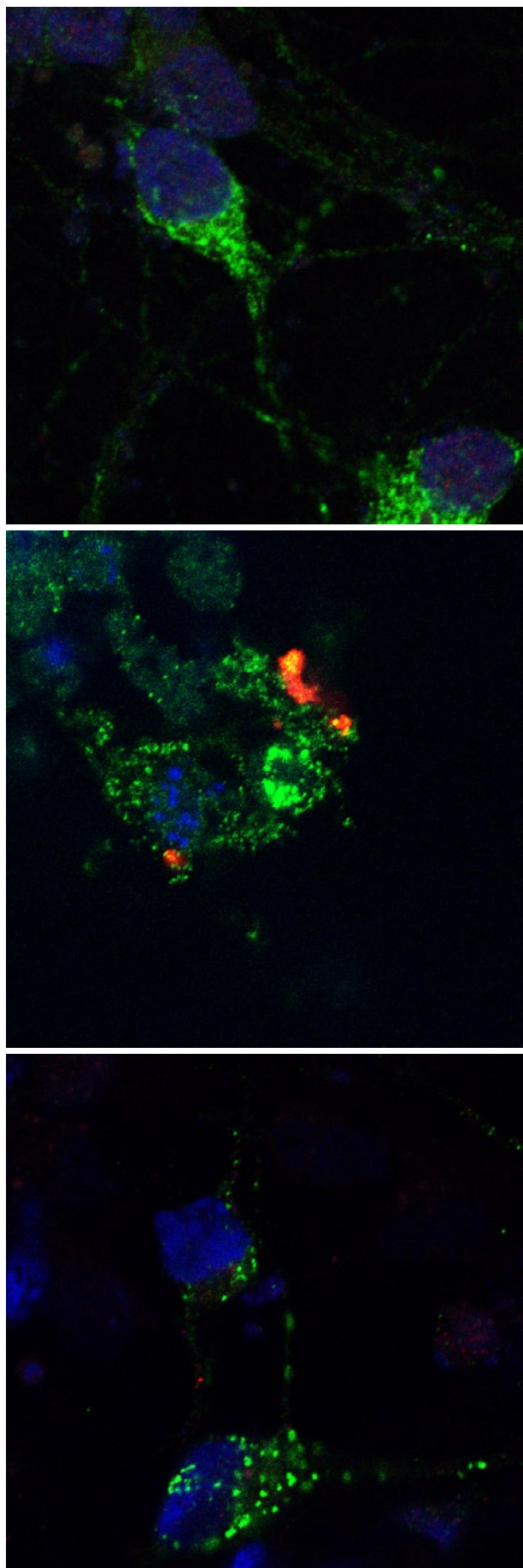

Supplement: Supplementary file 11 — Source Data for Figure 8 [file EMMM-15-e17451-s012.zip › Fig 8/8B/8B.pdf]

Source data for Fig. 8C

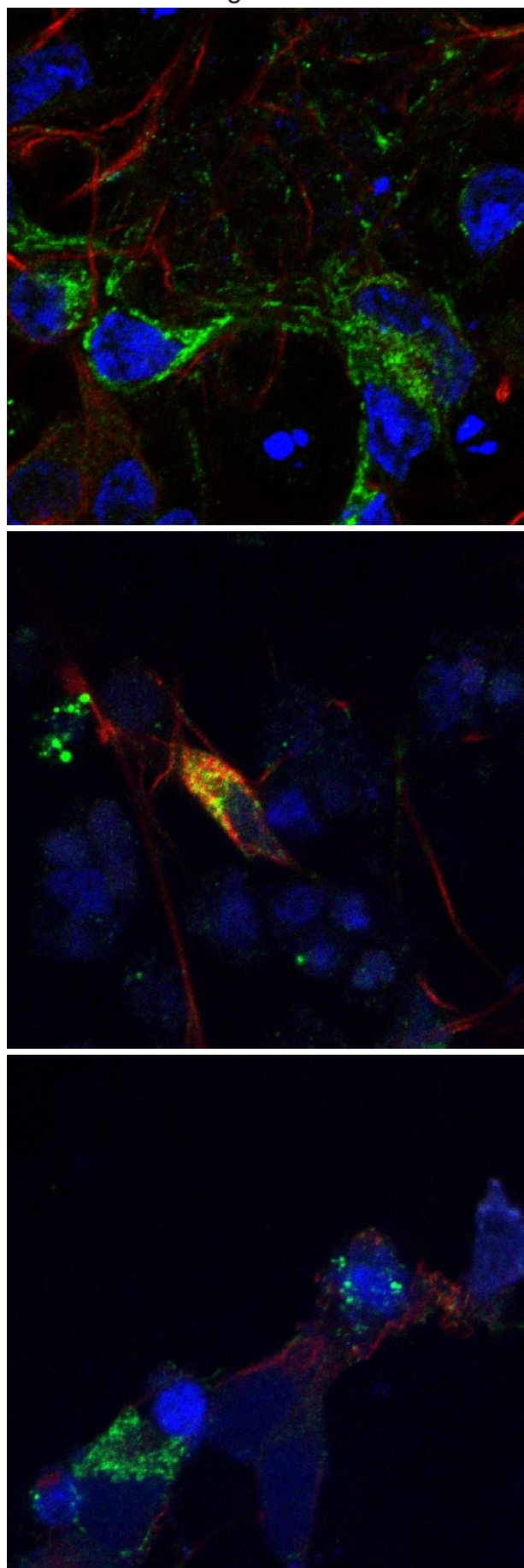

Supplement: Supplementary file 11 — Source Data for Figure 8 [file EMMM-15-e17451-s012.zip › Fig 8/8C/8C.pdf]

Source data for Fig. 8D

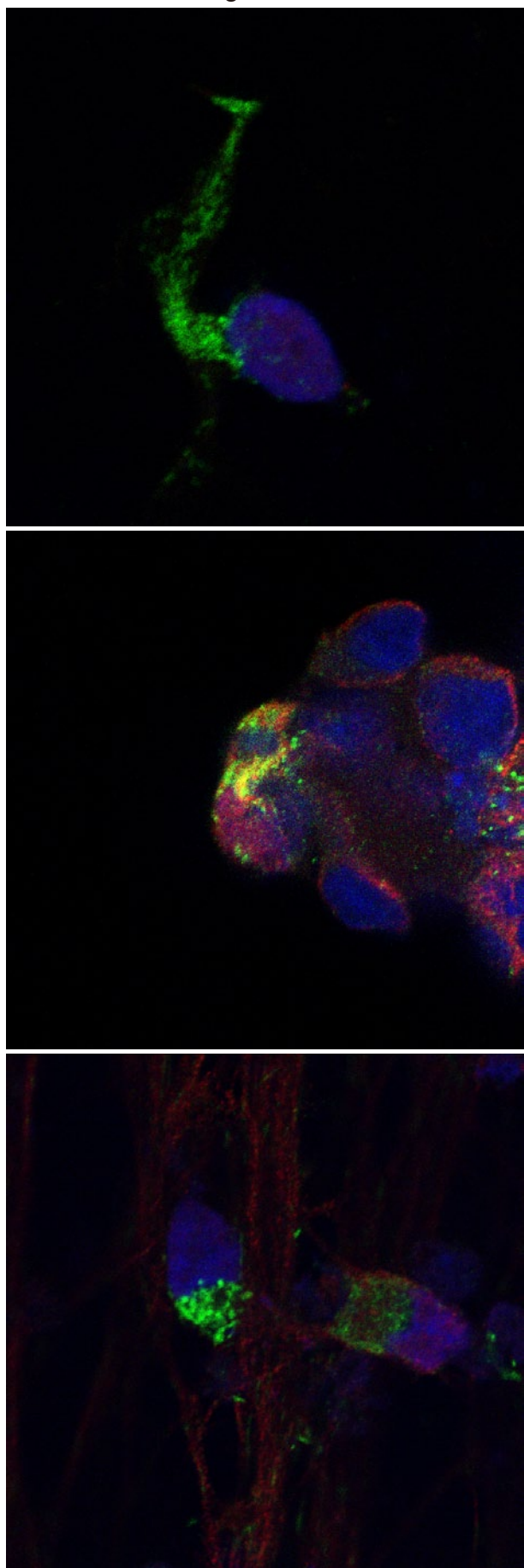

Supplement: Supplementary file 11 — Source Data for Figure 8 [file EMMM-15-e17451-s012.zip › Fig 8/8D/8D.pdf]

Source data for Fig. 8E

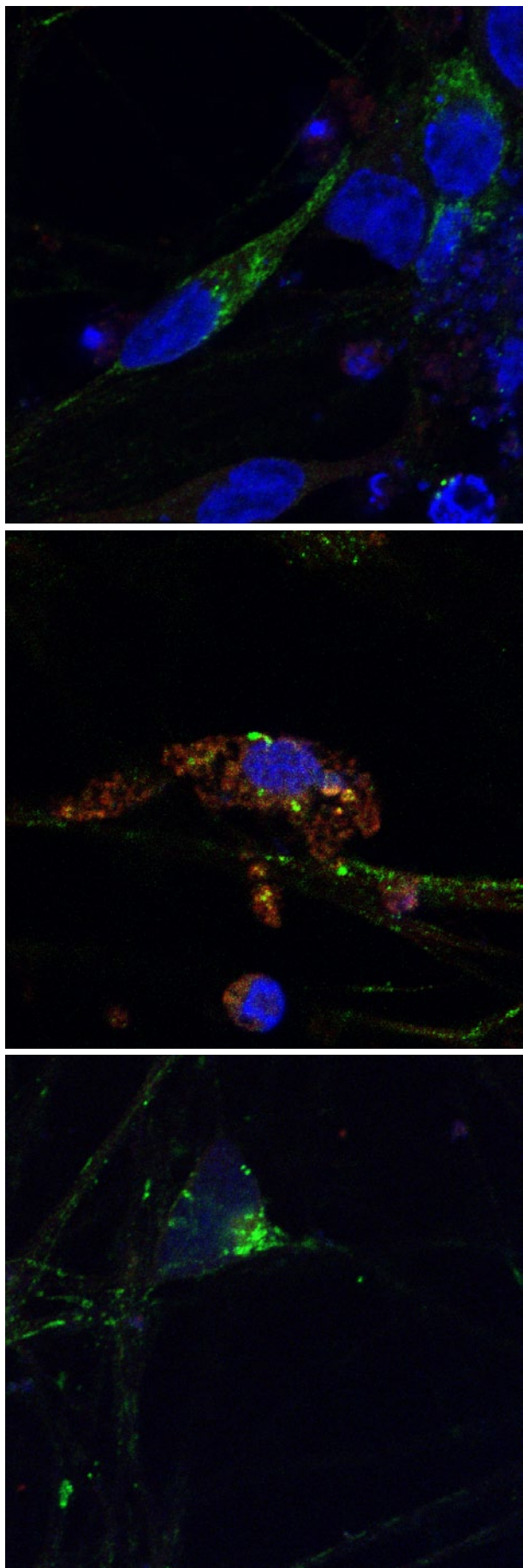

Supplement: Supplementary file 11 — Source Data for Figure 8 [file EMMM-15-e17451-s012.zip › Fig 8/8E/8E.pdf]

Source data for Fig. 8F

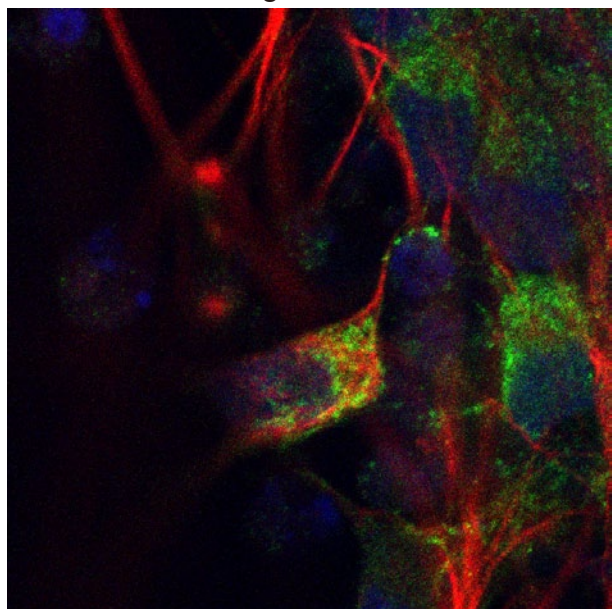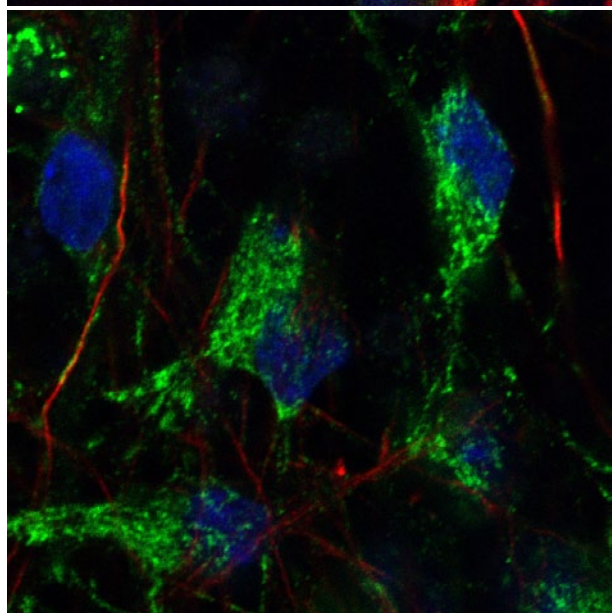

Supplement: Supplementary file 11 — Source Data for Figure 8 [file EMMM-15-e17451-s012.zip › Fig 8/8F/8F.pdf]

Source data for Fig. 8G

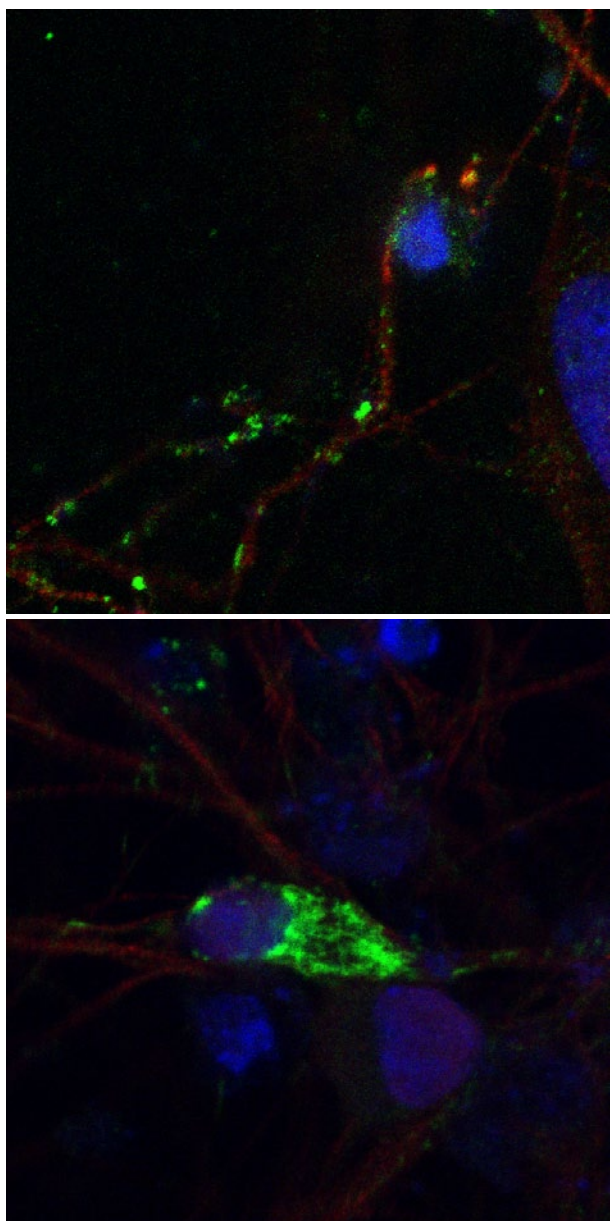

Supplement: Supplementary file 11 — Source Data for Figure 8 [file EMMM-15-e17451-s012.zip › Fig 8/8G/8G.pdf]

Source data for Fig. 8H

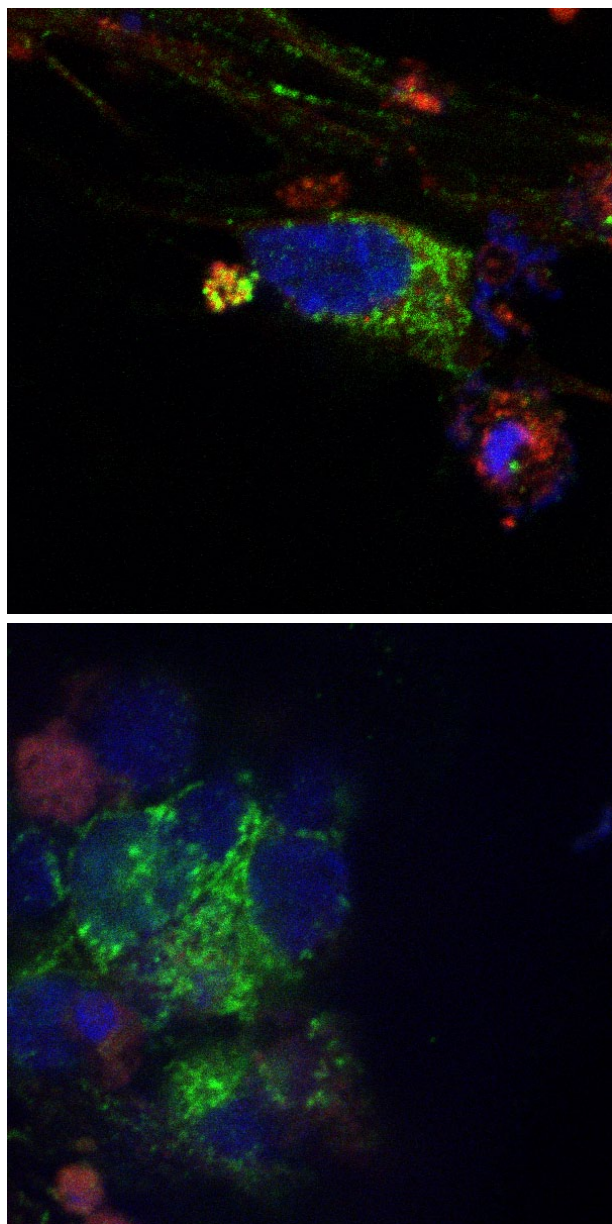

Supplement: Supplementary file 11 — Source Data for Figure 8 [file EMMM-15-e17451-s012.zip › Fig 8/8H/8H.pdf]
