# Supplementary material for: Involvement of casein kinase 1 epsilon/delta (Csnk1e/d) in the pathogenesis of familial Parkinson's disease caused by CHCHD2
Source: EMBO Mol Med. 2023 Aug 14;15(9):e17451. doi: 10.15252/emmm.202317451 (PMC10493588; doi:10.15252/emmm.202317451)
Supplement: Supplementary file 12 [file EMMM-15-e17451-s005.pdf]

EMBO Molecular Medicine

Involvement of Csnk1e/d in the pathogenesis of Parkinson's disease caused by CHCHD2 gene mutations

Satoru Torii, Kei-Ichi Ishikawa, Shigeto Sato, Hajime Sakurai, Shinya Honda, Masaya Ono, Wado Akamatsu, Nobutaka Hattori, Satoko Arakawa, Daisuke Taniguchi, Yuuichi Hiraoka and Shigeomi Shimizu
DOI: 10.15252/emmm.202317451

Corresponding author(s): Shigeomi Shimizu (shimizu.pcb@mri.tmd.ac.jp)

Review Timeline:

Submission Date:	18th Jan 23
Editorial Decision:	18th Feb 23
Revision Received:	30th May 23
Editorial Decision:	26th Jun 23
Revision Received:	4th Jul 23
Accepted:	5th Jul 23

Editor: Kelly Anderson

Transaction Report:

(Note: With the exception of the correction of typographical or spelling errors that could be a source of ambiguity, letters and reports are not edited. Depending on transfer agreements, referee reports obtained elsewhere may or may not be included in this compilation. Referee reports are anonymous unless the Referee chooses to sign their reports.)

18th Feb 2023

Dear Prof. Shimizu,

Thank you for the submission of your manuscript to EMBO Molecular Medicine. We have now received feedback from the two reviewers who agreed to evaluate your manuscript. As you will see from the reports below, the referees acknowledge the interest of the study and are overall supporting publication of your work pending appropriate revisions.

Addressing the reviewers' concerns in full will be necessary for further considering the manuscript in our journal, and acceptance of the manuscript will entail a second round of review. EMBO Molecular Medicine encourages a single round of revision only and therefore, acceptance or rejection of the manuscript will depend on the completeness of your responses included in the next, final version of the manuscript. For this reason, and to save you from any frustrations in the end, I would strongly advise against returning an incomplete revision.

Revised manuscripts should be submitted within three months of a request for revision; they will otherwise be treated as new submissions, except under exceptional circumstances in which a short extension is obtained from the editor.

I look forward to seeing a revised form of your manuscript as soon as possible.

Yours sincerely,

Kelly

Kelly M Anderson, PhD
Scientific Editor
EMBO Molecular Medicine

*****

When submitting your revised manuscript, please carefully review the instructions that follow below. We perform an initial quality control of all revised manuscripts before re-review; failure to include requested items will delay the evaluation of your revision.

We require:

1) A .docx formatted version of the manuscript text (including legends for main figures, EV figures and tables). Please make sure that the changes are highlighted to be clearly visible.

2) Individual production quality figure files as .eps, .tif, .jpg (one file per figure). For guidance, download the 'Figure Guide PDF': (https://www.embopress.org/page/journal/17574684/authorguide#figureformat).

3) A .docx formatted letter INCLUDING the reviewers' reports and your detailed point-by-point responses to their comments. As part of the EMBO Press transparent editorial process, the point-by-point response is part of the Review Process File (RPF), which will be published alongside your paper.

4) A complete author checklist, which you can download from our author guidelines (https://www.embopress.org/page/journal/17574684/authorguide#submissionofrevisions). Please insert information in the checklist that is also reflected in the manuscript. The completed author checklist will also be part of the RPF.

5) Please note that all corresponding authors are required to supply an ORCID ID for their name upon submission of a revised manuscript.

6) It is mandatory to include a 'Data Availability' section after the Materials and Methods. Before submitting your revision, primary datasets produced in this study need to be deposited in an appropriate public database, and the accession numbers and database listed under 'Data Availability'. Please remember to provide a reviewer password if the datasets are not yet public (see https://www.embopress.org/page/journal/17574684/authorguide#dataavailability).

In case you have no data that requires deposition in a public database, please state so in this section. Note that the Data Availability Section is restricted to new primary data that are part of this study.

7) For data quantification: please specify the name of the statistical test used to generate error bars and P values, the number

(n) of independent experiments (specify technical or biological replicates) underlying each data point and the test used to calculate p-values in each figure legend. The figure legends should contain a basic description of n, P and the test applied. Graphs must include a description of the bars and the error bars (s.d., s.e.m.). See also 'Figure Legend' guidelines: https://www.embopress.org/page/journal/17574684/authorguide#figureformat

8) At EMBO Press we ask authors to provide source data for the main manuscript figures. Our source data coordinator will contact you to discuss which figure panels we would need source data for and will also provide you with helpful tips on how to upload and organize the files.

9) Our journal encourages inclusion of *data citations in the reference list* to directly cite datasets that were re-used and obtained from public databases. Data citations in the article text are distinct from normal bibliographical citations and should directly link to the database records from which the data can be accessed. In the main text, data citations are formatted as follows: "Data ref: Smith et al, 2001" or "Data ref: NCBI Sequence Read Archive PRJNA342805, 2017". In the Reference list, data citations must be labeled with "[DATASET]". A data reference must provide the database name, accession number/identifiers and a resolvable link to the landing page from which the data can be accessed at the end of the reference. Further instructions are available at .

10) We replaced Supplementary Information with Expanded View (EV) Figures and Tables that are collapsible/expandable online. A maximum of 5 EV Figures can be typeset. EV Figures should be cited as 'Figure EV1, Figure EV2" etc... in the text and their respective legends should be included in the main text after the legends of regular figures.

- For the figures that you do NOT wish to display as Expanded View figures, they should be bundled together with their legends in a single PDF file called *Appendix*, which should start with a short Table of Content. Appendix figures should be referred to in the main text as: "Appendix Figure S1, Appendix Figure S2" etc.

- Additional Tables/Datasets should be labeled and referred to as Table EV1, Dataset EV1, etc. Legends have to be provided in a separate tab in case of .xls files. Alternatively, the legend can be supplied as a separate text file (README) and zipped together with the Table/Dataset file.

See detailed instructions here:

.

11) The paper explained: EMBO Molecular Medicine articles are accompanied by a summary of the articles to emphasize the major findings in the paper and their medical implications for the non-specialist reader. Please provide a draft summary of your article highlighting

- the medical issue you are addressing,

- the results obtained and

- their clinical impact.

This may be edited to ensure that readers understand the significance and context of the research. Please refer to any of our published articles for an example.

12) For more information: There is space at the end of each article to list relevant web links for further consultation by our readers. Could you identify some relevant ones and provide such information as well? Some examples are patient associations, relevant databases, OMIM/proteins/genes links, author's websites, etc...

13) Author contributions: You will be asked to provide CRediT (Contributor Role Taxonomy) terms in the submission system. These replace a narrative author contribution section in the manuscript.

14) A Conflict of Interest statement should be provided in the main text.

15) Every published paper now includes a 'Synopsis' to further enhance discoverability. Synopses are displayed on the journal webpage and are freely accessible to all readers. They include a short stand first (maximum of 300 characters, including space) as well as 2-5 one-sentences bullet points that summarizes the paper. Please write the bullet points to summarize the key NEW findings. They should be designed to be complementary to the abstract - i.e. not repeat the same text. We encourage inclusion of key acronyms and quantitative information (maximum of 30 words / bullet point). Please use the passive voice. Please attach these in a separate file or send them by email, we will incorporate them accordingly.

Please also suggest a striking image or visual abstract to illustrate your article as a PNG file 550 px wide x 300-600 px high.

EMBO Molecular Medicine has a "scooping protection" policy, whereby similar findings that are published by others during review or revision are not a criterion for rejection. Should you decide to submit a revised version, I do ask that you get in touch after three months if you have not completed it, to update us on the status.

Please note: When submitting your revision you will be prompted to enter your funding and payment information. This will allow Wiley to send you a quote for the article processing charge (APC) in case of acceptance. This quote takes into account any reduction or fee waivers that you may be eligible for. Authors do not need to pay any fees before their manuscript is accepted and transferred to the publisher.

EMBO Press participates in many Publish and Read agreements that allow authors to publish Open Access with reduced/no publication charges. Check your eligibility: https://authorservices.wiley.com/author-resources/Journal-Authors/open-access/affiliation-policies-payments/index.html

***** Reviewer's comments *****

Referee #1 (Remarks for Author):

In this interesting manuscript Torii et al. present the involvement of casein kinase epsilon/delta in CHCHD2-linked Parkinson's disease. They use an impressive number of cell and animal models as well patient tissue to demonstrate that mutant T61I CHCHD2 mislocalizes in cytosol and recruits there the Csnk1e/d, which phosphorylates neurofilament light and α-Synuclein, forming aggresomes. In addition they show in a mutant mouse model that Csnk1e/d inhibitor improved the disease phenotype. These are important findings for understanding the mechanism of CHCHD2-linked Parkinson's disease and for possibilities to stop the disease progression. Similar findings in a number of models is convincing, even when the depth of analysis of some models is a bit limited.

- Is it necessary to include the transgenic mouse data in main Figures when knock-in mouse data is also available? Perhaps move to Supplement.
-Western blots have n=1 and no quantification.
-Limb-clasping reflexes and footprint patterns have no quantification. Were the abnormalities seen in all mice at the same age? How many mice?
-Is there an effect with PF-670462 on WT mouse Rotarod performance?

Minor comments:
-title: Why CK1ε/δ in the title, whereas Csnk1e/d throughout the manuscript?
-abstract: PD results from the loss of dopaminergic neurons
-results, first paragraph: It could be explained more clearly why these data suggested that CHCHD2 mutant is first imported into mitochondria and subsequently removed from there. Some clarification is given in discussion but would be important to add here. Has it been shown that when mitochondrial import is blocked, CHCHD2 with the MTS can be detected on Western blot?
-results: "Neurofilament light chain (Nefl, also called Nfl/Nf68) is the basic subunit of neurofilament" is not correct, it is a subunit of neurofilament, but neurofilament can also form without Nefl.
-results: This sentence "Loss of Chchd2T61I from mitochondria was confirmed in mitochondria purified from Chchd2T61I-HA Tg mouse brains" is in the paragraph of the knock-in mice. Should it really read the Tg mice?
-results: the abnormal cell shapes of the iPSC neurons is not very convincing, they resemble death cell aggregates. Did the mutant cells differentiate normally? Was that quantified somehow? When did the abnormal cells appear?
-discussion: "This is because T61I localizes to the cytoplasm even in Bax/Bak double-deficient cells, which do not form apoptotic pores, and in cyclosporin A-treated cells, which do not form permeability transition pores." What is the reference for this?

Referee #2 (Comments on Novelty/Model System for Author):

They use several models, including a TG and a KI mouse model, cell cultures and they also validate findings in a brain of a patient.

Referee #2 (Remarks for Author):

Satoru Torii and colleagues investigated the involvement of CK1ε/δ in the pathogenesis of Parkinson's Disease caused by CHCHD2 gene mutations. The authors have deployed various models (MEF cells, Neuro2a cells, iPSCs, transgenic and knock-in homozygous and heterozygous mice, patient validations) to demonstrate that CHCHD2 T61I mutation causes CHCHD2 protein mislocalization in aggresomes, as well as CK1e/d recruitment which then phosphorylates Nefl and a-synuclein. CK1e/d inhibition reverses aggresome formation, phosphorylation of Nefl and a-synuclein and improves the dopaminergic neuron loss in

vivo and in vitro. CK1e/d siRNA has also been shown to improve the pathological observations caused by CHCHD2-T61I mutation. Overall this is a very nice and excellent paper with a lot of data and providing a convincing mechanism of disease.

Here are a few comments:
1. It would be useful to show the extend of CHCHD2-T61I mislocalization , NEFL and a-synuclein phosphorylation, or CK1e/d and aggresome accumulation by including representative images at lower magnification in the ICC figures and provide quantifications.
2. It appears in figure 1D that CHCHD2 T6lI-HA still partially colocalizes with Tom20(+) mitochondria. Since the authors claim that CHCHD2 T6lI localizes within aggresomes it would be good to demonstrate that mitochondria do not co-localize with aggresomes, CK1ε/δ, phospho-α-syn and phospho-Nefl.
3. Why do the authors think that the pathological observations are specific for dopaminergic neurons? Minimally they could check whether CHCHD2, CK1e/d, a-syn and Nefl RNA or protein levels are higher in the dopaminergic neurons relative to eg cortical neurons (based on publicly available datasets).
4. There are studies that have already reported a role for CK1 in PD that should be cited in this paper:
Reviewed in:
Dzamko N, Zhou J, Huang Y, Halliday GM. Parkinson's disease-implicated kinases in the brain; insights into disease pathogenesis. Front Mol Neurosci. 2014 Jun 24;7:57. doi: 10.3389/fnmol.2014.00057. PMID: 25009465; PMCID: PMC4068290.
5. Other comments:
page 8, row 10 there is a typo: its written "In vivo" instead of "In vitro" but the figure is about neuro2a cells

Point-by-point Responses

Responses to comment by Editor

Thank you for the submission of your manuscript to EMBO Molecular Medicine. We have now received feedback from the two reviewers who agreed to evaluate your manuscript. As you will see from the reports below, the referees acknowledge the interest of the study and are overall supporting publication of your work pending appropriate revisions.
Addressing the reviewers' concerns in full will be necessary for further considering the manuscript in our journal, and acceptance of the manuscript will entail a second round of review. EMBO Molecular Medicine encourages a single round of revision only and therefore, acceptance or rejection of the manuscript will depend on the completeness of your responses included in the next, final version of the manuscript. For this reason, and to save you from any frustrations in the end, I would strongly advise against returning an incomplete revision.

Response:
We greatly appreciate this high evaluation of our manuscript, as well as the kind comments. We have addressed all the reviewers' comments in full, as described below.

Responses to comment by Referee #1

Comment: In this interesting manuscript Torii et al. present the involvement of casein kinase epsilon/delta in CHCHD2-linked Parkinson's disease. They use an impressive number of cell and animal models as well patient tissue to demonstrate that mutant T61I CHCHD2 mislocalizes in cytosol and recruits there the Csnk1e/d, which phosphorylates neurofilament light and α-Synuclein, forming aggresomes. In addition they show in a mutant mouse model that Csnk1e/d inhibitor improved the disease phenotype. These are important findings for understanding the mechanism of CHCHD2-linked Parkinson's disease and for possibilities to stop the disease progression. Similar findings in a number of models is convincing, even when the depth of analysis of some models is a bit limited.

Response:
We greatly appreciate this high evaluation of our manuscript.

Comment #1: Is it necessary to include the transgenic mouse data in main Figures when knock-in mouse data is also available? Perhaps move to Supplement.

Response:
We agree with this comment. In accordance with this suggestion, the figure regarding the transgenic mouse data was moved to Figure EV4 and Appendix Figure S12-15 in the revised manuscript.

Comment #2: Western blots have n=1 and no quantification.

Response:
Thank you very much for this comment. In accordance with this suggestion, we showed quantified data and performed statistical analyses (see Figs. 1G, 2J, 3D, 4C, Appendix Fig. S1G, S9E, S10 and S13C).

Comment #3: **Limb-clasping reflexes and footprint patterns have no quantification. Were the abnormalities seen in all mice at the same age? How many mice?**

Response:
In accordance with this suggestion, we showed quantified data and performed statistical analyses (Appendix Fig. S8A, B for knock-in mice, Appendix Fig. S13A, B for transgenic mice, and Appendix Fig. S17C, D for knock-in mice treated with PF-670462). The limb-clasping reflexes was seen in 9 mice among the 10 hetero knock-in mice, and 8 mice among the 9 homo knock-in mice before at least 40-weeks old.

Comment #4: Is there an effect with PF-670462 on WT mouse Rotarod performance?

Response:
In response to this comment, we analyzed the effect of PF-670462 on WT mice, and showed the results in Appendix Fig. S17E. There was no effect of PF-670462 on the rotarod performance of WT mice.

Minor comments #1: title: Why CK1ε/δ in the title, whereas Csnk1e/d throughout the manuscript?

Response:

In accordance with this comment, we changed our manuscript title to **"Involvement of casein kinase 1 epsilon/delta (Csnk1e/d) in the pathogenesis of familial Parkinson's disease caused by *CHCHD2* gene mutations"**.

Minor comments #2: abstract: PD results from the loss of dopaminergic neurons

Response:

We appreciate this kind comment. In accordance with this suggestion, we corrected this sentence to the following.

> "Parkinson's disease (PD) is a common neurodegenerative disorder that results from the loss of dopaminergic neurons." (page 2 lines2–3).

Minor comments #3: results, first paragraph: It could be explained more clearly why these data suggested that CHCHD2 mutant is first imported into mitochondria and subsequently removed from there. Some clarification is given in discussion but would be important to add here. Has it been shown that when mitochondrial import is blocked, CHCHD2 with the MTS can be detected on Western blot?

Response:

Thank you very much for pointing this out. We felt that we did not have sufficient data on this matter, so we conducted additional experiments.

The presence of mitochondrial import can often be determined by the height of the band on western blot, because once in the mitochondria, the MTS is cleaved. If not, a protein with an MTS is expressed in the cytoplasm. In this study, in addition to comparing the band height of the T61I mutant with that of the P2L mutant, which does not undergo mitochondrial import, we also compared the band height when mitochondrial import inhibitors were added, as suggested by the Reviewer. As mitochondrial import inhibitors, we used antimycin A and MitoBlock-10 (a compound that inhibits Tim44 function) to the cells expressing CHCHD2-HA. Both inhibitors successfully blocked mitochondrial import, and CHCHD2 was observed at the 20-kD height despite usually being observed at 18-kD height (Fig. EV1A, B), validating the usefulness of this assay.

Regarding the T61I mutant, the band height was 18 kD, which is the same as wild-type CHCHD2. Therefore, we concluded that $CHCHD2^{T61I}$ is first imported into

mitochondria, where its N-terminus is cleaved, and is subsequently removed from mitochondria. These data were added to Fig. EV1 and explained in the revised manuscript, as follows.

"There are two possible mechanisms that may result in increased cytoplasmic localization of CHCHD2^{T61I} despite the presence of an N-terminal MTS: it can either enter the mitochondria and then be released into the cytoplasm, or it can be localized in the cytoplasm without entering the mitochondria. The height of the protein band on western blotting would be able to determine which is correct. In fact, when cells expressing CHCHD2^{WT} were treated with compounds that inhibit mitochondrial import (antimycin A and MitoBlock-10), CHCHD2^{WT} did not enter the mitochondria and an abnormal 20-kD band appeared instead of the normal 18-kD band (Fig. EV1A, B). On the other hand, for CHCHD2^{T61I} we observed a 18-kD band (Fig. EV1B), indicating that CHCHD2^{T61I} is first imported into mitochondria and then is subsequently removed from mitochondria." (page 5 lines13–23).

Fig EV1

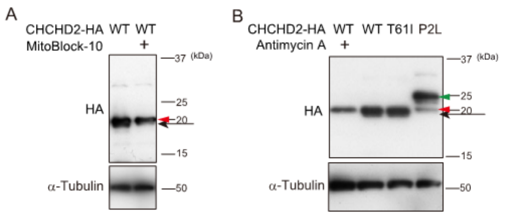

Effect of mitochondrial protein import inhibitors on western blot band sizes of CHCHD2 mutant proteins.

Minor comments #4: results: "Neurofilament light chain (Nefl, also called Nfl/Nf68) is the basic subunit of neurofilament" is not correct, it is a subunit of neurofilament, but neurofilament can also form without Nefl.

Response:

We appreciate this important comment. In accordance with the comment, we modified this sentence, as follows.

"Neurofilament light chain (Nefl, also called Nfl/Nf68) is a subunit of neurofilament, which is an abundant cytoskeletal protein in axons." (page 7, lines 5–6)

Minor comments #5: results: This sentence "Loss of Chchd2T61I from mitochondria was confirmed in mitochondria purified from Chchd2T61I-HA Tg mouse brains" is in the paragraph of the knock-in mice. Should it really read the Tg mice?

Response:

As pointed out by the Reviewer, our original description was wrong. In the revised manuscript, we replaced this description as follows.

> "Loss of Chchd2^{T61I} from mitochondria was confirmed in mitochondria purified from Chchd2^{T61I} knock-in mouse brains (Appendix Fig.S9D, E)." (page 10, lines 13–14)

Minor comments #6: results: the abnormal cell shapes of the iPSC neurons is not very convincing, they resemble death cell aggregates. Did the mutant cells differentiate normally? Was that quantified somehow? When did the abnormal cells appear?

Response:
We agree with this comment. To avoid confusion, we replaced the old figure with a new one, which indicates abnormal cell shapes, but not death aggregates, of the iPSC neurons (Appendix Fig. S18C, G in the revised manuscript).

As pointed out by the Reviewer, whether mutant cells differentiate normally is an important question. WT, CHCHD2^{T61I} patient-derived, and its isogenic control iPS cells were equivalently differentiated to dopaminergic cells, as quantitatively assessed by βIII-tubulin and TH neuronal markers. This point was already described in our previous paper (ref. 10 "Mutations in CHCHD2 cause alpha-synuclein aggregation" Hum Mol Genet, 28, 3895-3911). Only in the case of CHCHD2^{T61I} patient-derived iPS cells, abnormal cells appeared 15 to 20 days after induction of differentiation when differentiated neurons were sufficiently induced. We described these points in the revised manuscript, as follows.

> " We generated TH-expressing dopaminergic neurons from these iPS cells (Fig. EV5B, C) according to the established procedure described previously[10], by which these neurons are equivalently differentiated as assessed using the neuronal markers βIII-tubulin and TH[10]." (page 14 lines 18–21)

> "Abnormal cells appeared 15 to 20 days after the induction of differentiation only in patient-derived dopaminergic neurons." (page15 lines 5–7)

Minor comments #7: discussion: "This is because T61I localizes to the cytoplasm even in Bax/Bak double-deficient cells, which do not form apoptotic pores, and in cyclosporin A-treated cells, which do not form permeability transition pores." What is the reference for this?

Response:

As suggested by the Reviewer, we added the following new reference.

"23. Tsujimoto, Y. & Shimizu, S. (2007) Role of the mitochondrial membrane permeability transition in cell death. Apoptosis 12, 835-40."

Responses to comment by Referee #2

Comment: Satoru Torii and colleagues investigated the involvement of CK1ε/δ in the pathogenesis of Parkinson's Disease caused by CHCHD2 gene mutations. The authors have deployed various models (MEF cells, Neuro2a cells, iPSCs, transgenic and knock-in homozygous and heterozygous mice, patient validations) to demonstrate that CHCHD2 T61I mutation causes CHCHD2 protein mislocalization in aggresomes, as well as CK1e/d recruitment which then phosphorylates Nefl and a-synuclein. CK1e/d inhibition reverses aggresome formation, phosphorylation of Nefl and a-synuclein and improves the dopaminergic neuron loss in vivo and in vitro. CK1e/d siRNA has also been shown to improve the pathological observations caused by CHCHD2-T61I mutation. Overall this is a very nice and excellent paper with a lot of data and providing a convincing mechanism of disease.

Response:

We greatly appreciate this high evaluation of our manuscript.

Comment #1: It would be useful to show the extend of CHCHD2-T61I mislocalization, NEFL and a-synuclein phosphorylation, or CK1e/d and aggresome accumulation by including representative images at lower magnification in the ICC figures and provide quantifications.

Response:

We appreciate this constructive comment. In accordance with this suggestion, we newly added source image data to the main Figures, as well as and another low magnification images as supplementary images, and provided quantification data, as follows.

Low magnification images: Fig. S1D (Fig. 1A), Fig. S1E (Fig. 1D), Fig. S2A (Fig. 2A), Fig. S3A (Fig. 2C), Fig. S3B (Fig. 2E), Fig. S3C (Fig. 2G), Fig. S5B (Fig. 3B), Fig. S5C (Fig. 3E)

Quantification data: Fig. 1B, C, E, Fig. 2B, D, F, H, Fig. 4E, G, I, Fig. 7E, G, Fig. EV5E–J, Appendix Fig. S9G, I, L, Appendix Fig. S13D–F, Appendix Fig. S17F–H

Comment #2: It appears in figure 1D that CHCHD2 T6Il-HA still partially colocalizes with Tom20(+) mitochondria. Since the authors claim that CHCHD2 T6Il localizes within aggresomes it would be good to demonstrate that mitochondria do not co-localize with aggresomes, CK1ε/δ, phospho-α-syn and phospho-Nefl.

Response:

We appreciate this constructive comment. In accordance with this comment, we performed an immunostaining assay and confirmed that mitochondria do not colocalize with aggresomes, phospho-Nefl, phospho-α-Synuclein, and CK1ε/δ (Csnk1e/d). These data are shown in Appendix Fig. S2B, S3D, S3E, and S5A.

Comment #3: Why do the authors think that the pathological observations are specific for dopaminergic neurons? Minimally they could check whether CHCHD2, CK1e/d, a-syn and Nefl RNA or protein levels are higher in the dopaminergic neurons relative to eg cortical neurons (based on publicly available datasets).

Response:

This is an important point to be addressed. We therefore checked the expression of these proteins in "The Human Protein Atlas, https://www.proteinatlas.org/". According to this dataset, the RNA expression levels of CHCHD2, CK1e, α-Synuclein, and Nefl are not higher in the midbrain relative to the cerebral cortex and cerebellum.

In Chchd2^{T61I} knock-in mice, we observed equivalent Chchd2^{T61I} expression levels in the cerebral cortex and cerebellum as in the midbrain, but less Chchd2^{T61I} mislocalization from mitochondria was observed in the cerebral cortex than in the midbrain. Therefore, we think that any protein that regulates CHCHD2 localization is important. Alternatively, protein levels of Csnk1e/d in pathogenic conditions might be important. We hence described these points in the revised manuscript, as follows.

> "In this study, we found that the CHCHD2^{T61I} mutation has a greater effect in the SNpc than in the cortex or cerebellum. mRNA expression levels did not differ significantly among these regions according to the database[24,25], and indeed no differences in protein expression levels were observed in CHCHD2^{T61I} knock-in

mice (Appendix Fig. S11B). One reason for this specificity is that CHCHD2^{T61I} tends to mislocalize more specifically to the cytoplasm in neurons of the SNpc (Figs. 5G, EV4H, and Appendix Figs. S11C, S15B). This might be because the amount and type of proteins that bind and keep CHCHD2^{T61I} in the mitochondria may differ among brain regions. In addition, Csnk1e might be specifically upregulated by extramitochondrial CHCHD2^{T61I} in the SNpc (Fig. 5L, EV4B, and Appendix Fig. S11B, S15C). This increase in Csnk1e is expected to accelerate the recruitment of phospho-NEFL^{472} and phospho-α-Synuclein^{129}, rendering dopaminergic neurons to be more prone to abnormalities. These two factors may be responsible for the specific damage to dopaminergic neurons by CHCHD2^{T61I}." (page 17 lines 10–23)

Comment #4: There are studies that have already reported a role for CK1 in PD that should be cited in this paper:
Reviewed in: Dzamko N, Zhou J, Huang Y, Halliday GM. Parkinson's disease-implicated kinases in the brain; insights into disease pathogenesis. Front Mol Neurosci. 2014 Jun 24;7:57. doi: 10.3389/fnmol.2014.00057. PMID: 25009465; PMCID: PMC4068290.

Response:
Thank you very much for this information. We added the following new reference.

> "16, Dzamko, N., Zhou, J., Huang, Y. & Halliday, G.M. (2014) Parkinson's disease-implicated kinases in the brain; insights into disease pathogenesis. Front Mol Neurosci 7, 57."

Comment #5: page 8, row 10 there is a typo: its written "In vivo" instead of "In vitro" but the figure is about neuro2a cells

Response:
We appreciate this comment. We corrected this label.

26th Jun 2023

Dear Prof. Shimizu,

Congratulations on a great revision! Overall, the referees have been positive. However, one referee has a few minor editorial concerns that should be addressed in a revised version. When you submit your revised version, please also take care of the following editorial items and add this also to your point-by-point response:

1. For EV figures, please upload as Figures and not as Expanded View.

2. For the data availability section, we do not allow Data Available upon request statements. Rather, we require mass spec identified proteins (or any type of sequencing data) to be deposited in repositories and made available to readers. Please see our website for more information.

3. Please enter the appropriate funding information into eJP online.

4. Please remove the author contribution section from the manuscript file.

5. Please review our new policy on conflict of interests on the EMBO author guide and update the title of this section to: Disclosure and competing interests statement

6. There is space at the end of each article to list relevant web links for further consultation by our readers. Could you identify some relevant ones and provide such information as well? Some examples are patient associations, relevant databases, OMIM/proteins/genes links, author's websites, etc. This section is called "For more information" and can be useful for readers, but is not required.

7. For references, these should be in alphabetic order and only the first 10 author names should be displayed. In cases where there are more than 10 authors, add et al. after the 10th author name.

8. For the table of contents in the appendix file, please add page numbers.

9. Source data: The source data for each main figures needs to be provided in one zipped folder and uploaded separately. Please also provide source data for Fig. 5B, 6K-M, and 9A-h. Also the source data for EV figures should be zipped up on one folder.

10. Please resize the synopsis image to 550 pixels wide x 200-400 pixels high

11. All figures should be referred to in the manuscript and in sequential order. Please correct the following that are missing: 2D, 2F, 5J, 7E-G, 8D, 8E, 8G, 8H, EV3D. Appendix Fig3AB, Appendix Fig. 11, individual panels of Fig. 2, 3, 5, and 8 should be called out sequentially. The appendix tables should be referred to as Appendix Table S1, etc. in the main text. You have also referred to Supplementary Table 1 and 2, but there are no additional tables, please correct.

12. Legends for the Appendix figures should be removed from the main ms file.

13. Please reorder the sections in the ms file to: abstract, intro, results, discussion, materials & methods, data availability, acknowledgments, disclosure statement and competing interests, references, main figure legends, expanded figure legends.

Thank you for the opportunity to consider your work for publication. I look forward to your revision.

Kind regards,
Kelly

Kelly M Anderson, PhD
Scientific Editor
EMBO Molecular Medicine

*** Instructions to submit your revised manuscript ***

*** PLEASE NOTE *** As part of the EMBO Publications transparent editorial process initiative (see our Editorial at https://www.embopress.org/doi/pdf/10.1002/emmm.201000094), EMBO Molecular Medicine will publish online a Review

Process File to accompany accepted manuscripts.

In the event of acceptance, this file will be published in conjunction with your paper and will include the anonymous referee reports, your point-by-point response and all pertinent correspondence relating to the manuscript. If you do NOT want this file to be published, please inform the editorial office at contact@embomolmed.org.

When submitting your revised manuscript, please include:

1) a .docx formatted version of the manuscript text (including Figure legends and tables)

2) Separate figure files*

3) supplemental information as Expanded View and/or Appendix. Please carefully check the authors guidelines for formatting Expanded view and Appendix figures and tables at https://www.embopress.org/page/journal/17574684/authorguide#expandedview

4) a letter INCLUDING the reviewer's reports and your detailed responses to their comments (as Word file).

5) The paper explained: EMBO Molecular Medicine articles are accompanied by a summary of the articles to emphasize the major findings in the paper and their medical implications for the non-specialist reader. Please provide a draft summary of your article highlighting
- the medical issue you are addressing,
- the results obtained and
- their clinical impact.
This may be edited to ensure that readers understand the significance and context of the research.
Please refer to any of our published articles for an example.

6) For more information: There is space at the end of each article to list relevant web links for further consultation by our readers. Could you identify some relevant ones and provide such information as well? Some examples are patient associations, relevant databases, OMIM/proteins/genes links, author's websites, etc...

7) Author contributions: the contribution of every author must be detailed in a separate section.

8) EMBO Molecular Medicine now requires a complete author checklist (https://www.embopress.org/page/journal/17574684/authorguide) to be submitted with all revised manuscripts. Please use the checklist as guideline for the sort of information we need WITHIN the manuscript. The checklist should only be filled with page numbers were the information can be found. This is particularly important for animal reporting, antibody dilutions (missing) and exact values and n that should be indicted instead of a range.

9) Every published paper now includes a 'Synopsis' to further enhance discoverability. Synopses are displayed on the journal webpage and are freely accessible to all readers. They include a short stand first (maximum of 300 characters, including space) as well as 2-5 one sentence bullet points that summarise the paper. Please write the bullet points to summarise the key NEW findings. They should be designed to be complementary to the abstract - i.e. not repeat the same text. We encourage inclusion of key acronyms and quantitative information (maximum of 30 words / bullet point). Please use the passive voice. Please attach these in a separate file or send them by email, we will incorporate them accordingly.

You are also welcome to suggest a striking image or visual abstract to illustrate your article. If you do please provide a jpeg file 550 px-wide x 400-px high.

10) A Conflict of Interest statement should be provided in the main text

11) Please note that we now mandate that all corresponding authors list an ORCID digital identifier. This takes <90 seconds to complete. We encourage all authors to supply an ORCID identifier, which will be linked to their name for unambiguous name identification.

Currently, our records indicate that the ORCID for your account is 0000-0002-8437-0791.

Please click the link below to modify this ORCID:

Link Not Available

12) The system will prompt you to fill in your funding and payment information. This will allow Wiley to send you a quote for the article processing charge (APC) in case of acceptance. This quote takes into account any reduction or fee waivers that you may be eligible for. Authors do not need to pay any fees before their manuscript is accepted and transferred to our publisher.

*Additional important information regarding Figures

Each figure should be given in a separate file and should have the following resolution:
Graphs 800-1,200 DPI
Photos 400-800 DPI
Colour (only CMYK) 300-400 DPI"

Figures are not edited by the production team. All lettering should be the same size and style; figure panels should be indicated by capital letters (A, B, C etc). Gridlines are not allowed except for log plots. Figures should be numbered in the order of their appearance in the text with Arabic numerals. Each Figure must have a separate legend and a caption is needed for each panel.

*Additional important information regarding figures and illustrations can be found at https://bit.ly/EMBOPressFigurePreparationGuideline. See also figure legend preparation guidelines: https://www.embopress.org/page/journal/17574684/authorguide#figureformat

The system will prompt you to fill in your funding and payment information. This will allow Wiley to send you a quote for the article processing charge (APC) in case of acceptance. This quote takes into account any reduction or fee waivers that you may be eligible for. Authors do not need to pay any fees before their manuscript is accepted and transferred to our publisher.

***** Reviewer's comments *****

Referee #1 (Remarks for Author):

The authors have addressed my comments in an excellent manner.

Please note two typos in Fig 1G: organelles and cytoskeleton

Referee #2 (Remarks for Author):

The authors did a very good job. Here are some absolutely minor comments:

Page 4: CK1d is a paralogue of CK1e, not an orthologue

Page 6: CHCHD2 T61I localization doesn't look cytoplasmic as indicated in page 6 but rather vesicular

Page 7: CHCHD2 T61I is not completely absent from mitochondria, but its greatly reduced

Page 63 (figure 1G): typo - cytoskeleton instead of cytockelton

Page 23 (methods): "infusion of the CK1d/e inhibitor to the brain with an osmotic pump that is placed subcutaneously at the back of the mice at 16 weeks (4 months) of age. The osmotic pump was replaced every 28 days". According to providers of the used inhibitor (Medchem and selleckchem), the inhibitor half life is about a day and recommended storage after dissolving is at -20 to -80. Can the authors specify how they maintained the inhibitor active throughout 28 days at body temperature?

Point-by-point Responses

Responses to comment by Editor

Congratulations on a great revision! Overall, the referees have been positive. However, one referee has a few minor editorial concerns that should be addressed in a revised version. When you submit your revised version, please also take care of the following editorial items and add this also to your point-by-point response:

Response:

We greatly appreciate this high evaluation of our manuscript, as well as the kind comments. We have addressed all the editorial concerns and reviewers' comments in full, as described below.

Editor's comment #1: For EV figures, please upload as Figures and not as Expanded View.

Response:

Thank you very much for this comment. We uploaded EV figures as Figures.

Editor's comment #2. For the data availability section, we do not allow Data Available upon request statements. Rather, we require mass spec identified proteins (or any type of sequencing data) to be deposited in repositories and made available to readers. Please see our website for more information.

Response:

We made new source excel data, in which identified proteins from mass spectrometry in Fig. 3A is included. We updated the Data availability section and corrected the sentence to the following.

> "The data of identified proteins from mass spectrometry (Fig. 3A) is included within the source data." (page 30 lines 7-8).

Editor's comment #3. Please enter the appropriate funding information into eJP online.

Response:
Thank you very much for this comment. We entered the appropriate funding information.

Editor's comment #4. Please remove the author contribution section from the manuscript file.

Response:
In accordance with this comment, we removed the author contribution section.

Editor's comment #5. Please review our new policy on conflict of interests on the EMBO author guide and update the title of this section to: Disclosure and competing interests statement

Response:
In accordance with this comment, we updated the title of the section to: Disclosure and competing interests statement.

Editor's comment #6. There is space at the end of each article to list relevant web links for further consultation by our readers. Could you identify some relevant ones and provide such information as well? Some examples are patient associations, relevant databases, OMIM/proteins/genes links, author's websites, etc. This section is called "For more information" and can be useful for readers, but is not required.

Response:
Thank you very much for this comment. We don't have additional information.

Editor's comment #7. For references, these should be in alphabetic order and only the first 10 author names should be displayed. In cases where there are more than 10 authors, add et al. after the 10th author name.

Response:
In accordance with this comment, we updated the reference section and corrected the format of references in main text.

Editor's comment #8. For the table of contents in the appendix file, please add page numbers.

Response:
We added the page numbers in the table of contents in the appendix file.

Editor's comment #9. Source data: The source data for each main figures needs to be provided in one zipped folder and uploaded separately. Please also provide source data for Fig. 5B, 6K-M, and 9A-h. Also the source data for EV figures should be zipped up on one folder.

Response:
We provided source data for Fig. 5B and 6B (images of footprint data). Our previous manuscript did not have Fig. 6K-M, and 9A-h. We here provided source data for new Fig. 5K-M (old Fig. 6K-M) and new Fig. 8A-h (old 9A-h) in revised manuscript. We provided each source data as each zipped folder.

Editor's comment #10. Please resize the synopsis image to 550 pixels wide x 200-400 pixels high

Response:
We resized the synopsis image to 550 pixels wide x 400 pixels high.

Editor's comment #11. All figures should be referred to in the manuscript and in sequential order. Please correct the following that are missing: 2D, 2F, 5J, 7E-G, 8D, 8E, 8G, 8H, EV3D. Appendix Fig3AB, Appendix Fig. 11, individual panels of Fig. 2, 3, 5, and 8 should be called out sequentially. The appendix tables should be referred to as Appendix Table S1, etc. in the main text. You have also referred to Supplementary Table 1 and 2, but there are no additional tables, please correct.

Response:
We corrected the manuscript. These panels in Figures were called out sequentially. We checked main text and corrected "Appendix Table S1" in Material and Methods. We removed the word "Supplementary" in legends of Fig. EV2 and Appendix Fig. S12.

Editor's comment #12. Legends for the Appendix figures should be removed from

the main ms file.

Response:
In accordance with this comment, we removed legends for the Appendix figures.

Editor's comment #13. Please reorder the sections in the ms file to: abstract, intro, results, discussion, materials & methods, data availability, acknowledgments, disclosure statement and competing interests, references, main figure legends, expanded figure legends.

Response:
In accordance with this comment, we reordered the sections in the manuscript.

<u>Responses to Reviewers' comments</u>

<u>Responses to comment by Referee #1</u>
Comment #1: authors have addressed my comments in an excellent manner.

Response:
We greatly appreciate this high evaluation of our manuscript.

Comment #2: Please note two typos in Fig 1G: organelles and cytoskeleton

Response:
We appreciate this kind comment. We have corrected the typo according to the reviewers' suggestion.

<u>Responses to comment by Referee #2</u>
Comment #1: The authors did a very good job. Here are some absolutely minor comments:

Response:
We greatly appreciate this high evaluation of our manuscript.

Comment #2: Page 4: CK1d is a paralogue of CK1e, not an orthologue

Response:
We appreciate this kind comment. We corrected related sentences to the following.

> "We found that Chchd2^{T61I} is mislocalized to outside of mitochondria, where casein kinase 1 epsilon (Csnk1e) and its <u>paralogue</u> casein kinase 1 delta (Csnk1d) were recruited. " (page 4 lines 16-18).
> "Csnk1d is a close <u>paralogue</u> of Csnk1e, and they share substrates and perform the same biological roles in some cases (Bibian *et al*, 2013; Hanna-Addams *et al*, 2020). " (page 9 lines 7-9).

Comment #3: Page 6: CHCHD2 T61I localization doesn't look cytoplasmic as indicated in page 6 but rather vesicular

Response:
We appreciate this kind comment. In accordance with this comment, we avoided use the word “cytoplasmic” and corrected this sentence to the following.

> “There are two possible mechanisms that may result in increased extra-mitochondrial localization of CHCHD2^{T61I} despite the presence of an N-terminal MTS: it can either enter the mitochondria and then be released into the cytoplasm, or it can be localized in the cytoplasm without entering the mitochondria.” (page 6 lines 12-16).

Comment #4: Page 7: CHCHD2 T61I is not completely absent from mitochondria, but its greatly reduced

Response:
Thank you very much for this comment. Based on this comment, we corrected the sentence in Page 7 to the following.

> “Consistently, cell fractionation analysis showed that most of the CHCHD2^{WT} was recovered in the mitochondria-containing organellar fraction, whereas the amount of CHCHD2^{T61I} in this fraction was greatly reduced and was mostly recovered in the insoluble fraction (Fig. 1F and G).” (page 7 lines 7-10).

Comment #5: Page 63 (figure 1G): typo - cytoskeleton instead of cytockelton

Response:
We appreciate this kind comment. In accordance with the comment, we modified Fig. 1.

Comment #6: Page 23 (methods): "infusion of the CK1d/e inhibitor to the brain with an osmotic pump that is placed subcutaneously at the back of the mice at 16 weeks (4 months) of age. The osmotic pump was replaced every 28 days". According to providers of the used inhibitor (Medchem and selleckchem), the inhibitor half life is about a day and recommended storage after dissolving is at -20 to -80. Can the authors specify how they maintained the inhibitor active throughout 28 days at body temperature?

Response:
Thank you very much for pointing this out. PF-670462 is a selective Csnk1e and 1d

inhibitor (IC50 values are 7.7 and 14 nM respectively). We used 10 μM PF-670462 in cultured cells. In osmotic pump experiments of mice, 0.5 mM PF-670462 was used (50 ng/g body weight/day). This concentration (0.5 mM) was defined as when all PF-670462 in the pump was dissolved in mice (the concentration is 5 μM in 20 ml), assuming the total weight of the mouse (for example, 20 g) as water. As this concentration (0.5 mM) is a high concentration, we consider that the activity is maintained for some time. Also, even if the drug is not permanently effective, the initial administration may be sufficient. To reduce the number of pump changes, which are stressful for mice, a 28-day pump must be used in this study. In view of the above, we decided to conduct our mouse experiments under these conditions in the present study.

5th Jul 2023

Dear Prof. Shimizu,

Please find enclosed the final reports on your manuscript. We are pleased to inform you that your manuscript is accepted for publication and is now being sent to our publisher to be included in the next available issue of EMBO Molecular Medicine.

We would like to remind you that as part of the EMBO Publications transparent editorial process initiative, EMBO Molecular Medicine will publish a Review Process File online to accompany accepted manuscripts. If you do NOT want the file to be published or would like to exclude figures, please immediately inform the editorial office via e-mail.

Please read below for additional IMPORTANT information regarding your article, its publication and the production process.

Congratulations on your interesting work,

Kelly

Kelly M Anderson, PhD
Scientific Editor
EMBO Molecular Medicine

Follow us on Twitter @EmboMolMed @Kelly_EMBOJ
Sign up for eTOCs at embopress.org/alertsfeeds

*** *** *** IMPORTANT INFORMATION *** *** ***

SPEED OF PUBLICATION
The journal aims for rapid publication of papers, using using the advance online publication "Early View" to expedite the process: A properly copy-edited and formatted version will be published as "Early View" after the proofs have been corrected. Please help the Editors and publisher avoid delays by providing e-mail address(es), telephone and fax numbers at which author(s) can be contacted.

Should you be planning a Press Release on your article, please get in contact with embomolmed@wiley.com as early as possible, in order to coordinate publication and release dates.

LICENSE AND PAYMENT:

All articles published in EMBO Molecular Medicine are fully open access: immediately and freely available to read, download and share.

EMBO Molecular Medicine charges an article processing charge (APC) to cover the publication costs. You, as the corresponding author for this manuscript, should have already received a quote with the article processing fee separately. Please let us know in case this quote has not been received.

Once your article is at Wiley for editorial production you will receive an email from Wiley's Author Services system, which will ask you to log in and will present you with the publication license form for completion. Within the same system the publication fee can be paid by credit card, an invoice, pro forma invoice or purchase order can be requested.

Payment of the publication charge and the signed Open Access Agreement form must be received before the article can be published online.

PROOFS

You will receive the proofs by e-mail approximately 2 weeks after all relevant files have been sent o our Production Office. Please return them within 48 hours and if there should be any problems, please contact the production office at embopressproduction@wiley.com.

Please inform us if there is likely to be any difficulty in reaching you at the above address at that time. Failure to meet our

deadlines may result in a delay of publication.

All further communications concerning your paper proofs should quote reference number EMM-2023-17451-V3 and be directed to the production office at embopressproduction@wiley.com.

Thank you,

Kelly M Anderson, PhD
Scientific Editor
EMBO Molecular Medicine

EMBO Press Author Checklist

Corresponding Author Name: Shigeomi Shimizu, Satoru Torii
Journal Submitted to: EMBO Molecular Medicine
Manuscript Number: EMM-2023-17451

USEFUL LINKS FOR COMPLETING THIS FORM
The EMBO Journal - Author Guidelines
EMBO Reports - Author Guidelines
Molecular Systems Biology - Author Guidelines
EMBO Molecular Medicine - Author Guidelines

Reporting Checklist for Life Science Articles (updated January
This checklist is adapted from Materials Design Analysis Reporting (MDAR) Checklist for Authors. MDAR establishes a minimum set of requirements in transparent reporting in the life sciences (see Statement of Task: 10.31222/osf.io/9sm4x). Please follow the journal's guidelines in preparing your manuscript.
Please note that a copy of this checklist will be published alongside your article.

Abridged guidelines for figures
1. Data
The data shown in figures should satisfy the following conditions:
- the data were obtained and processed according to the field's best practice and are presented to reflect the results of the experiments in an accurate and unbiased manner.
- ideally, figure panels should include only measurements that are directly comparable to each other and obtained with the same assay.
- plots include clearly labeled error bars for independent experiments and sample sizes. Unless justified, error bars should not be shown for technical
- if n<5, the individual data points from each experiment should be plotted. Any statistical test employed should be justified.
- Source Data should be included to report the data underlying figures according to the guidelines set out in the authorship guidelines on Data

2. Captions
Each figure caption should contain the following information, for each panel where they are relevant:
- a specification of the experimental system investigated (eg cell line, species name).
- the assay(s) and method(s) used to carry out the reported observations and measurements.
- an explicit mention of the biological and chemical entity(ies) that are being measured.
- an explicit mention of the biological and chemical entity(ies) that are altered/varied/perturbed in a controlled manner.
- the exact sample size (n) for each experimental group/condition, given as a number, not a range;
- a description of the sample collection allowing the reader to understand whether the samples represent technical or biological replicates (including how many animals, litters, cultures, etc.).
- a statement of how many times the experiment shown was independently replicated in the laboratory.
- definitions of statistical methods and measures:
  - common tests, such as t-test (please specify whether paired vs. unpaired), simple χ2 tests, Wilcoxon and Mann-Whitney tests, can be unambiguously identified by name only, but more complex techniques should be described in the methods section;
  - are tests one-sided or two-sided?
  - are there adjustments for multiple comparisons?
  - exact statistical test results, e.g., P values = x but not P values < x;
  - definition of 'center values' as median or average;
  - definition of error bars as s.d. or s.e.m.

Please complete ALL of the questions below.
Select "Not Applicable" only when the requested information is not relevant for your study.

Materials

Newly Created Materials	Information included in the manuscript?	In which section is the information available? (Reagents and Tools Table, Materials and Methods, Figures, Data Availability Section)
New materials and reagents need to be available; do any restrictions apply?	Not Applicable	

Antibodies	Information included in the manuscript?	In which section is the information available? (Reagents and Tools Table, Materials and Methods, Figures, Data Availability Section)
For **antibodies** provide the following information:<br>- Commercial antibodies: RRID (if possible) or supplier name, catalogue number and or/clone number<br>- Non-commercial: RRID or citation	Yes	Appendix Table S2

DNA and RNA sequences	Information included in the manuscript?	In which section is the information available? (Reagents and Tools Table, Materials and Methods, Figures, Data Availability Section)
**Short novel DNA or RNA including primers, probes**: provide the sequences.	Yes	Materials and Methods, and Appendix Table S1

Cell materials	Information included in the manuscript?	In which section is the information available? (Reagents and Tools Table, Materials and Methods, Figures, Data Availability Section)
**Cell lines:** Provide species information, strain. Provide accession number in repository **OR** supplier name, catalog number, clone number, and/**OR** RRID.	Yes	Materials and Methods
**Primary cultures:** Provide species, strain, sex of origin, genetic modification status.	Yes	Materials and Methods
Report if the cell lines were recently **authenticated** (e.g., by STR profiling) and tested for mycoplasma contamination.	Not Applicable	

Experimental animals	Information included in the manuscript?	In which section is the information available? (Reagents and Tools Table, Materials and Methods, Figures, Data Availability Section)
**Laboratory animals or Model organisms:** Provide species, strain, sex, age, genetic modification status. Provide accession number in repository **OR** supplier name, catalog number, clone number, **OR** RRID.	Yes	Materials and Methods
**Animal observed in or captured from the field:** Provide species, sex, and age where possible.	Not Applicable	
Please detail **housing and husbandry conditions**.	Yes	Materials and Methods

Plants and microbes	Information included in the manuscript?	In which section is the information available? (Reagents and Tools Table, Materials and Methods, Figures, Data Availability Section)
**Plants:** provide species and strain, ecotype and cultivar where relevant, unique accession number if available, and source (including location for collected wild specimens).	Not Applicable	
**Microbes:** provide species and strain, unique accession number if available, and source.	Not Applicable	

Human research participants	Information included in the manuscript?	In which section is the information available? (Reagents and Tools Table, Materials and Methods, Figures, Data Availability Section)
If collected and within the bounds of privacy constraints report on age, sex and gender or ethnicity for all study participants.	Yes	Materials and Methods

Core facilities	Information included in the manuscript?	In which section is the information available? (Reagents and Tools Table, Materials and Methods, Figures, Data Availability Section)
If your work benefited from core facilities, was their service mentioned in the acknowledgments section?	Not Applicable	

Design

Study protocol	Information included in the manuscript?	In which section is the information available? (Reagents and Tools Table, Materials and Methods, Figures, Data Availability Section)
If study protocol has been **pre-registered, provide DOI in the manuscript**. For clinical trials, provide the trial registration number **OR** cite DOI.	Not Applicable	
Report the **clinical trial registration number** (at ClinicalTrials.gov or equivalent), where applicable.	Not Applicable	

Laboratory protocol	Information included in the manuscript?	In which section is the information available? (Reagents and Tools Table, Materials and Methods, Figures, Data Availability Section)
Provide DOI OR other citation details if **external detailed step-by-step protocols** are available.	Not Applicable	

Experimental study design and statistics	Information included in the manuscript?	In which section is the information available? (Reagents and Tools Table, Materials and Methods, Figures, Data Availability Section)
Include a statement about **sample size** estimate even if no statistical methods were used.	Yes	Materials and Methods and Figure legends
Were any steps taken to minimize the effects of subjective bias when allocating animals/samples to treatment (e.g. **randomization procedure**)? If yes, have they been described?	Not Applicable	
Include a statement about **blinding** even if no blinding was done.	Not Applicable	
Describe **inclusion/exclusion criteria** if samples or animals were excluded from the analysis. Were the criteria pre-established?<br><br>If sample or data points were omitted from analysis, report if this was due to attrition or intentional exclusion and provide justification.	Not Applicable	
For every figure, are **statistical tests** justified as appropriate? Do the data meet the assumptions of the tests (e.g., normal distribution)? Describe any methods used to assess it. Is there an estimate of variation within each group of data? Is the variance similar between the groups that are being statistically compared?	Not Applicable	

Sample definition and in-laboratory replication	Information included in the manuscript?	In which section is the information available? (Reagents and Tools Table, Materials and Methods, Figures, Data Availability Section)
In the figure legends: state number of times the experiment was **replicated** in laboratory.	Not Applicable	
In the figure legends: define whether data describe **technical or biological replicates**.	Yes	Figure legends

Ethics

Ethics	Information included in the manuscript?	In which section is the information available? (Reagents and Tools Table, Materials and Methods, Figures, Data Availability Section)
Studies involving **human participants**: State details of **authority granting ethics approval** (IRB or equivalent committee(s), provide reference number for approval.	Yes	Material and Methods
Studies involving **human participants**: Include a statement confirming that **informed consent** was obtained from all subjects and that the experiments conformed to the principles set out in the WMA Declaration of Helsinki and the Department of Health and Human Services Belmont Report.	Yes	Material and Methods
Studies involving **human participants:** For publication of **patient photos**, include a statement confirming that consent to publish was obtained.	Not Applicable	
Studies involving experimental **animals**: State details of **authority granting ethics approva**l (IRB or equivalent committee(s), provide reference number for approval. Include a statement of compliance with ethical regulations.	Yes	Material and Methods
Studies involving **specimen and field samples:** State if relevant **permits** obtained, provide details of authority approving study; if none were required, explain why.	Not Applicable	

Dual Use Research of Concern (DURC)	Information included in the manuscript?	In which section is the information available? (Reagents and Tools Table, Materials and Methods, Figures, Data Availability Section)
Could your study fall under dual use research restrictions? Please check biosecurity documents and list of **select agents and toxins** (CDC): https://www.selectagents.gov/sat/list.htm	Not Applicable	
If you used a select agent, is the security level of the lab appropriate and reported in the manuscript?	Not Applicable	
If a study is subject to dual use research of concern regulations, is the name of the **authority granting approval and reference number** for the regulatory approval provided in the manuscript?	Not Applicable	

Reporting

The MDAR framework recommends adoption of discipline-specific guidelines, established and endorsed through community initiatives. Journals have their own policy about requiring specific guidelines and recommendations to complement MDAR.

Adherence to community standards	Information included in the manuscript?	In which section is the information available? (Reagents and Tools Table, Materials and Methods, Figures, Data Availability Section)
State if relevant guidelines or checklists (e.g., **ICMJE, MIBBI, ARRIVE, PRISMA**) have been followed or provided.	Not Applicable	
For **tumor marker prognostic studies,** we recommend that you follow the **REMARK** reporting guidelines (see link list at top right). See author guidelines, under 'Reporting Guidelines'. Please confirm you have followed these guidelines.	Not Applicable	
For **phase II and III randomized controlled trials**, please refer to the **CONSORT** flow diagram (see link list at top right) and submit the CONSORT checklist (see link list at top right) with your submission. See author guidelines, under 'Reporting Guidelines'. Please confirm you have submitted this list.	Not Applicable	

Data Availability

Data availability	Information included in the manuscript?	In which section is the information available? (Reagents and Tools Table, Materials and Methods, Figures, Data Availability Section)
Have **primary datasets** been deposited according to the journal's guidelines (see 'Data Deposition' section) and the respective accession numbers provided in the Data Availability Section?	Not Applicable	
Were **human clinical and genomic datasets** deposited in a public access-controlled repository in accordance to ethical obligations to the patients and to the applicable consent agreement?	Not Applicable	
Are **computational models** that are central and integral to a study available without restrictions in a machine-readable form? Were the relevant accession numbers or links provided?	Not Applicable	
If publicly available data were reused, provide the respective **data citations in the reference list**.	Not Applicable	
